# Safety and Efficacy of Combined Tixagevimab and Cilgavimab Administered Intramuscularly or Intravenously in Nonhospitalized Patients With COVID-19: 2 Randomized Clinical Trials

**DOI:** 10.1001/jamanetworkopen.2023.10039

**Published:** 2023-04-26

**Authors:** Rachel A. Bender Ignacio, Kara W. Chew, Carlee Moser, Judith S. Currier, Joseph J. Eron, Arzhang Cyrus Javan, Mark J. Giganti, Evgenia Aga, Michael Gibbs, Hervé Tchouakam Kouekam, Eva Johnsson, Mark T. Esser, Keila Hoover, Gene Neytman, Matthew Newell, Eric S. Daar, William Fischer, Courtney V. Fletcher, Jonathan Z. Li, Alexander L. Greninger, Robert W. Coombs, Michael D. Hughes, Davey Smith, David Alain Wohl

**Affiliations:** 1 Division of Allergy and Infectious Diseases, Department of Medicine, University of Washington, Seattle; 2 Vaccine and Infectious Diseases Division, Fred Hutchinson Cancer Center, Seattle, Washington; 3 Division of Infectious Diseases, Department of Medicine, David Geffen School of Medicine, UCLA (University of California, Los Angeles); 4 Department of Biostatistics, Harvard T. H. Chan School of Public Health, Boston, Massachusetts; 5 Division of Infectious Diseases, Department of Medicine, The University of North Carolina School of Medicine, Chapel Hill; 6 National Institute of Allergy and Infectious Diseases, National Institutes of Health, Bethesda, Maryland; 7 Vaccines & Immune Therapies, BioPharmaceuticals R&D, AstraZeneca, Cambridge, United Kingdom; 8 Vaccines & Immune Therapies, BioPharmaceuticals R&D, AstraZeneca, Toronto, Ontario, Canada; 9 Vaccines & Immune Therapies, BioPharmaceuticals R&D, AstraZeneca, Gothenburg, Sweden; 10 Vaccines & Immune Therapies, BioPharmaceuticals R&D, AstraZeneca, Gaithersburg, Maryland; 11 Miami Clinical Research and Baptist Health South Florida, Miami; 12 Quantum Clinical Trials, Miami Beach, Florida; 13 Division of HIV Medicine, Lundquist Institute, Harbor-UCLA Medical Center, Los Angeles, California; 14 Division of Pulmonary Diseases and Critical Care Medicine, The University of North Carolina School of Medicine, Chapel Hill; 15 UNMC Center for Drug Discovery, University of Nebraska Medical Center, Omaha; 16 Division of Infectious Diseases, Department of Medicine, Harvard Medical School, Boston, Massachusetts; 17 Department of Laboratory Medicine and Pathology, University of Washington Medical Center, Seattle; 18 Division of Infectious Diseases and Global Public Health, Department of Medicine, University of California, San Diego

## Abstract

**Importance:**

Development of effective, scalable therapeutics for SARS-CoV-2 is a priority.

**Objective:**

To test the efficacy of combined tixagevimab and cilgavimab monoclonal antibodies for early COVID-19 treatment.

**Design, Setting, and Participants:**

Two phase 2 randomized blinded placebo-controlled clinical trials within the Accelerating COVID-19 Therapeutic Interventions and Vaccines (ACTIV)–2/A5401 platform were performed at US ambulatory sites. Nonhospitalized adults 18 years or older within 10 days of positive SARS-CoV-2 test and symptom onset were eligible and were enrolled from February 1 to May 31, 2021.

**Interventions:**

Tixagevimab-cilgavimab, 300 mg (150 mg of each component) given intravenously (IV) or 600 mg (300 mg of each component) given intramuscularly (IM) in the lateral thigh, or pooled placebo.

**Main Outcomes and Measures:**

Coprimary outcomes were time to symptom improvement through 28 days; nasopharyngeal SARS-CoV-2 RNA below the lower limit of quantification (LLOQ) on days 3, 7, or 14; and treatment-emergent grade 3 or higher adverse events through 28 days.

**Results:**

A total of 229 participants were randomized for the IM study and 119 were randomized for the IV study. The primary modified intention-to-treat population included 223 participants who initiated IM tixagevimab-cilgavimab (n = 106) or placebo treatment (n = 117) (median age, 39 [IQR, 30-48] years; 113 [50.7%] were men) and 114 who initiated IV tixagevimab-cilgavimab (n = 58) or placebo treatment (n = 56) (median age, 44 [IQR, 35-54] years; 67 [58.8%] were women). Enrollment in the IV study was stopped early based on a decision to focus on IM product development. Participants were enrolled at a median of 6 (IQR, 4-7) days from COVID-19 symptom onset. Significant differences in time to symptom improvement were not observed for IM tixagevimab-cilgavimab vs placebo or IV tixagevimab-cilgavimab vs placebo. A greater proportion in the IM tixagevimab-cilgavimab arm (69 of 86 [80.2%]) than placebo (62 of 96 [64.6%]) had nasopharyngeal SARS-CoV-2 RNA below LLOQ at day 7 (adjusted risk ratio, 1.33 [95% CI, 1.12-1.57]) but not days 3 and 14; the joint test across time points favored treatment (*P* = .003). Differences in the proportion below LLOQ were not observed for IV tixagevimab-cilgavimab vs placebo at any of the specified time points. There were no safety signals with either administration route.

**Conclusions:**

In these 2 phase 2 randomized clinical trials, IM or IV tixagevimab-cilgavimab was safe but did not change time to symptom improvement. Antiviral activity was more evident in the larger IM trial.

**Trial Registration:**

ClinicalTrials.gov Identifier: NCT04518410

## Key Points

**Question:**

Do tixagevimab and cilgavimab, 2 long-acting anti–SARS-CoV-2 monoclonal antibodies given in combination, improve symptoms and viral shedding when administered intramuscularly in the thigh or intravenously in persons with early COVID-19?

**Findings:**

In these 2 phase 2 randomized clinical trials within the Accelerating COVID-19 Therapeutic Interventions and Vaccines (ACTIV)–2/A5401 platform, tixagevimab-cilgavimab given intramuscularly or intravenously was safe but did not shorten symptom duration. Fewer participants who received intramuscular treatment vs placebo had quantifiable SARS-CoV-2 RNA in nasopharyngeal swabs at day 7.

**Meaning:**

Tixagevimab-cilgavimab produced modest antiviral effects, suggesting potential clinical activity for treatment of SARS-CoV-2 infection, but had no effect on symptom duration.

## Teaser

These 2 phase 2 randomized clinical trials evaluated the safety and efficacy of single-dose combination tixagevimab and cilgavimab administered intramuscularly and intravenously, each compared with placebo, for treatment of symptomatic nonhospitalized adults with early COVID-19.

## Introduction

The COVID-19 pandemic caused by SARS-CoV-2 continues, and therapies that prevent hospitalization and/or death have received US Food and Drug Administration emergency use authorization, including monoclonal antibodies (mAbs) and direct-acting antivirals.^1,2,3,4,5,6,7^ Given 1-time dosing, safety in those with liver or kidney disease, and lack of drug-drug interactions, mAbs have been relied on to treat persons at risk for severe COVID-19, especially those for whom currently available oral agents are contraindicated. To date, all mAbs authorized for COVID-19 treatment have been delivered intravenously (IV); alternative administration routes for mAbs, including intramuscular (IM) injection, could improve treatment access.

AZD7442 is a combination of 2 anti–SARS-CoV-2 mAbs, tixagevimab (AZD8895) and cilgavimab (AZD1061), both derived from persons recovered from COVID-19. Tixagevimab-cilgavimab administered by IM gluteal injection previously received a US Food and Drug Administration emergency use authorization for preexposure prophylaxis.^8,9^ These mAbs bind unique, nonoverlapping epitopes at the human angiotensin-converting enzyme 2 interface of the receptor-binding domain of the SARS-CoV-2 spike protein. Modifications in Fc regions were designed to extend their half-life to approximately 90 days and reduce risk of antibody-dependent enhancement.^10,11,12,13,14,15^ The combination mAb demonstrated in vitro neutralization of SARS-CoV-2, with reduced activity against several Omicron subvariants.^16,17,18,19^ We herein evaluated the safety and efficacy of single-dose combination tixagevimab-cilgavimab administered IM and IV, each compared with placebo, for treatment of symptomatic nonhospitalized adults with early COVID-19 within the Accelerating COVID-19 Therapeutic Interventions and Vaccines (ACTIV)–2 platform trial.

## Methods

### Trial Design and Oversight

ACTIV-2/A5401 is a multicenter, controlled platform randomized clinical trial designed to evaluate investigational agents for treatment of nonhospitalized adults with mild-to-moderate COVID-19. The trial protocol is provided in Supplement 1. The protocol was approved by a central institutional review board, with additional local approval as required by sites. Participants provided written informed consent. The study followed the Consolidated Standards of Reporting Trials (CONSORT) reporting guideline.

Tixagevimab-cilgavimab administered IV and IM were studied as separate agents, and each group was enrolled in parallel and compared with placebo. Participants were randomized in 2 steps to an investigational agent group (eg, IM tixagevimab-cilgavimab, IV tixagevimab-cilgavimab, or another agent in the platform), and then to blinded active agent or placebo for that agent, within the assigned group. For each investigational agent, a control arm was constructed by pooling all participants who were eligible for that agent group and randomized to placebo for that agent or any other concurrently enrolling agent. Randomization was stratified by time from symptom onset (≤5 or >5 days) and risk of disease progression (lower or higher). Details on trial design, including randomization scheme, are given in eMethods 1 in Supplement 2.

Each phase 2 evaluation was powered on the primary virologic outcome. The target sample size for each phase 2 evaluation was 220 (110 each for active agent and placebo), affording at least 82% power to detect a 20% absolute increase in proportion with viral RNA below the lower limit of quantification vs placebo, with 2-sided 5% type I error.

### Participants and Procedures

Eligible participants were nonhospitalized adults 18 years or older with symptomatic SARS-CoV-2 infection documented by positive antigen or nucleic acid testing results and symptom onset within 10 days of entry; early in enrollment, eligibility was further restricted to 8 days of symptoms as more data on the kinetics of SARS-CoV-2 became available.^20^ Eligibility for IV tixagevimab-cilgavimab was restricted to participants at higher risk of progression to severe COVID-19 based on protocol-defined age and comorbidities criteria; IM-eligible participants could be at lower or higher risk. Persons needing hospitalization or who had received investigational SARS-CoV-2 treatments or convalescent plasma were excluded. Enrollment into both tixagevimab-cilgavimab groups was limited to US sites (eMethods 2 in Supplement 2).

Participants self-reported their race and ethnicity (Asian, Black, Hispanic or Latino, White, or other race or ethnicity [including American Indian or Alaska Native, multiple races or ethnicities, or other race or ethnicity]) and gender (cisgender, transgender spectrum, or not reported). These data were collected in accordance with reporting requirements for clinical trials and to ensure representation among the study participants.

On day 0, participants randomized to the IM group received 600 mg of tixagevimab-cilgavimab (300 mg in 3 mL of each component, 1 component delivered to each lateral thigh [IM T-C arm]), or equivalent volumes of IM saline placebo. Participants in the IV group received 300 mg of tixagevimab-cilgavimab (150 mg of each component admixed [IV T-C arm]) or saline placebo infused over approximately 15 minutes. Visits were conducted at days 3, 7, 14, and 28 for clinical assessments and staff-collected nasopharyngeal swabs. Participants self-collected anterior nasal swabs daily through day 14 and completed a daily symptom diary through day 28.^20^

### Study Objectives and Outcome Measures

The 3 primary outcomes were (1) time to symptom improvement through 28 days; (2) SARS-CoV-2 RNA below the lower limit of quantification (LLOQ; 2.0 log_10_ copies/mL) on nasopharyngeal samples at days 3, 7, or 14; and (3) new grade 3 or higher treatment-emergent adverse events (TEAEs) through day 28. Time to symptom improvement was defined as days from entry to the first of 2 consecutive days when all 13 targeted symptoms scored as moderate or severe at study entry were scored as mild or absent, and all symptoms scored as mild or absent at entry were scored as absent. Key secondary outcomes through day 28 included time to symptom resolution (time to the first of 2 consecutive days when all targeted symptoms were reported absent), progression of 1 or more targeted symptoms to a worse severity than at entry, time to self-reported return to usual health, time-averaged total daily symptom score, the composite of all-cause hospitalization or death, quantitative nasopharyngeal SARS-CoV-2 RNA levels, and SARS-CoV-2 RNA below the LLOQ on anterior nasal swabs through day 14. Virologic methods were previously published.^20^

### Statistical Analysis

Analyses were restricted to the modified intention-to-treat population, defined as all randomized participants who initiated the study intervention. Time to symptom improvement, symptom resolution, and return to usual health were compared between arms using the Gehan-Wilcoxon test for censored data. Time-averaged total daily symptom score was compared using a 2-sided Wilcoxon rank sum test.

The proportion of participants with nasopharyngeal and anterior nasal SARS-CoV-2 RNA less than the LLOQ was compared between arms across study visits using Poisson regression with robust variance adjusted for entry log_10_ transformed SARS-CoV-2 RNA level, summarized with risk ratios (RRs) and 95% CIs and joint Wald test across the time points. Quantitative nasopharyngeal SARS-CoV-2 RNA levels at each postentry visit were compared between arms using Wilcoxon rank sum tests. Post hoc analysis was conducted to examine changes in nasopharyngeal RNA from day 0 among participants with quantifiable day 0 nasopharyngeal RNA using linear regression models for censored data, adjusting for baseline RNA level.

The proportion of participants experiencing a new grade 3 or greater TEAE through day 28 and the proportion with symptom progression were compared between arms using log-binomial regression and summarized with an RR and *P* value based on the Wald test. The proportion of hospitalizations and/or deaths through day 28 was compared between arms using a Fisher exact test.

All comparisons used a 2-sided 5% type I error without adjustment for multiple comparisons; *P* < .05 indicated statistical significance. Statistical analyses were conducted with SAS software, version 9.4 (SAS Institute Inc). The ACTIV-2 statistical analysis plan is available in Supplement 1.

## Results

### Study Population

Between February 1 and May 31, 2021, 228 participants were randomized to the IM T-C arm or pooled placebo; 223 (106 to active treatment and 117 to placebo) initiated study intervention and were included in the modified intention-to-treat analysis (CONSORT diagram in eFigure, A, in Supplement 2). Of the 119 participants randomized to the IV T-C arm or pooled placebo, 114 (58 to active treatment and 56 to placebo) initiated study intervention and were included in the modified intention-to-treat analysis (CONSORT diagram in eFigure, B, in Supplement 2). Baseline characteristics are described in Table 1. The median age was 39 (IQR, 30-48) years in the IM trial, with 12 of 223 (5.4%) 60 years or older (110 [49.3%] women and 113 [50.7%] men). In terms of race and ethnicity, 3 participants in the IM trial (1.3%) were Asian, 26 (11.7%) were Black, 99 (44.4%) were Hispanic or Latino, 181 (81.2%) were White, and 12 (5.4%) were of other race or ethnicity (including American Indian or Alaska Native, multiple races or ethnicities, or other race or ethnicity). Participants in the IV trial had a median age of 44 (IQR, 35-44) years, with 17 of 114 (14.9%) 60 years or older (67 [58.8%] women and 47 [41.2%] men). For self-reported race and ethnicity, 1 participant in the IV trial (0.9%) was Asian, 22 (19.3%) were Black, 54 (47.4%) were Hispanic or Latino, 89 (78.1%) were White, and 2 (1.8%) were of other race or ethnicity. Only 13 participants in the IM trial (5.8%) and 1 in the IV trial (0.9%) reported receiving at least 1 COVID-19 vaccine dose prior to entry. Overall, 100 participants in the IM trial (44.8%) and 45 in the IV trial (39.5%) enrolled within 5 days of symptom onset. Enrollment into the IV trial was stopped early following a decision by the manufacturer to focus on development of IM administration of tixagevimab-cilgavimab due to evolving standards of COVID-19 treatment; there were no safety concerns.

**Table 1.** Demographics of Participants Receiving IM or IV Tixagevimab-Cilgavimab and Their Respective Pooled Placebo Arms

Characteristic	Tixagevimab-cilgavimab trial^a^					
	IM administration			IV administration		
	T-C (n = 106)	Placebo (n = 117)	All (n = 223)	T-C (n = 58)	Placebo (n = 56)	All (n = 114)
Age, median (IQR), y	40 (32-48)	38 (29-48)	39 (30-48)	43 (33-51)	46 (35-58)	44 (35-54)
Sex						
Women	52 (49.1)	58 (49.6)	110 (49.3)	34 (58.6)	33 (58.9)	67 (58.8)
Men	54 (50.9)	59 (50.4)	113 (50.7)	24 (41.4)	23 (41.1)	47 (41.2)
Gender^b^						
Cisgender	105 (99.1)	116 (99.1)	221 (99.1)	58 (100)	56 (100)	114 (100)
Transgender spectrum	0	1 (0.9)	1 (0.4)	0	0	0
Not reported	1 (0.9)	0	1 (0.4)	0	0	0
Race^b^						
Asian	2 (1.9)	1 (0.9)	3 (1.3)	1 (1.7)	0	1 (0.9)
Black	8 (7.5)	18 (15.4)	26 (11.7)	10 (17.2)	12 (21.4)	22 (19.3)
White	88 (83.0)	93 (79.5)	181 (81.2)	47 (81.0)	42 (75.0)	89 (78.1)
Other^c^	7 (6.6)	5 (4.3)	12 (5.4)	0	2 (3.6)	2 (1.8)
Missing	1 (0.9)	0	1 (0.4)	0	0	0
Ethnicity^b^						
Hispanic or Latino	52 (49.1)	47 (40.2)	99 (44.4)	27 (46.6)	27 (48.2)	54 (47.4)
Not Hispanic or Latino	54 (50.9)	70 (59.8)	124 (55.6)	31 (53.4)	29 (51.8)	60 (52.6)
BMI, median (IQR)	28 (25-33)	28 (25-32)	28 (25-32)	34 (26-37)	31 (27-36)	31 (27-37)
Duration of symptoms prior to entry, d						
Median (IQR)	6 (4-7)	6 (4-7)	6 (4-7)	6 (3-7)	6 (4-7)	6 (4-7)
≤5	47 (44.3)	53 (45.3)	100 (44.8)	21 (36.2)	24 (42.9)	45 (39.5)
COVID-19 progression risk						
Higher	33 (31.1)	33 (28.2)	66 (29.6)	58 (100)	56 (100)	114 (100)
Lower	73 (68.9)	84 (71.8)	157 (70.4)	0	0	0
History of SARS-CoV-2 vaccination	9 (8.5)	4 (3.4)	13 (6)	1 (1.7)^d^	0	1 (0.9)

Abbreviations: BMI, body mass index (calculated as weight in kilograms divided by height in meters squared); IM, intramuscularly; IV, intravenously; T-C, study group receiving active treatment with tixagevimab-cilgavimab.

^a^ Unless otherwise indicated, data are expressed as No. (%) of participants. Percentages have been rounded and may not total 100. All participants were from sites in the US. IM administration consists of 600 mg (300 mg of each component) of monoclonal antibodies or 600 mg of placebo; IV administration, 300 mg (150 mg of each component) of monoclonal antibodies or 300 mg of placebo.

^b^ Self-identified by participants.

^c^ Includes American Indian or Alaska Native, multiple races or ethnicities, and other race or ethnicity.

^d^ For most study enrollment into both IM and IV groups, persons who had received SARS-CoV-2 vaccines were considered lower risk. The single IV participant who was vaccinated enrolled under Protocol V3.0, when having received a vaccine was not included in risk stratification.

Variant analysis was available for 202 participants in either the IV or IM trials: 115 (56.9%) had the Alpha variant, 23 (11.4%) had the Iota variant, 1 (0.5%) had the Delta variant, and 63 (31.2%) had a mix of other variants (eTable 7 in Supplement 2). In the IM trial, 211 (94.6%) remained on study through day 28, as did 108 (94.7%) in the IV trial.

### Clinical Outcomes

#### IM Tixagevimab-Cilgavimab Trial

**Figure 1.**
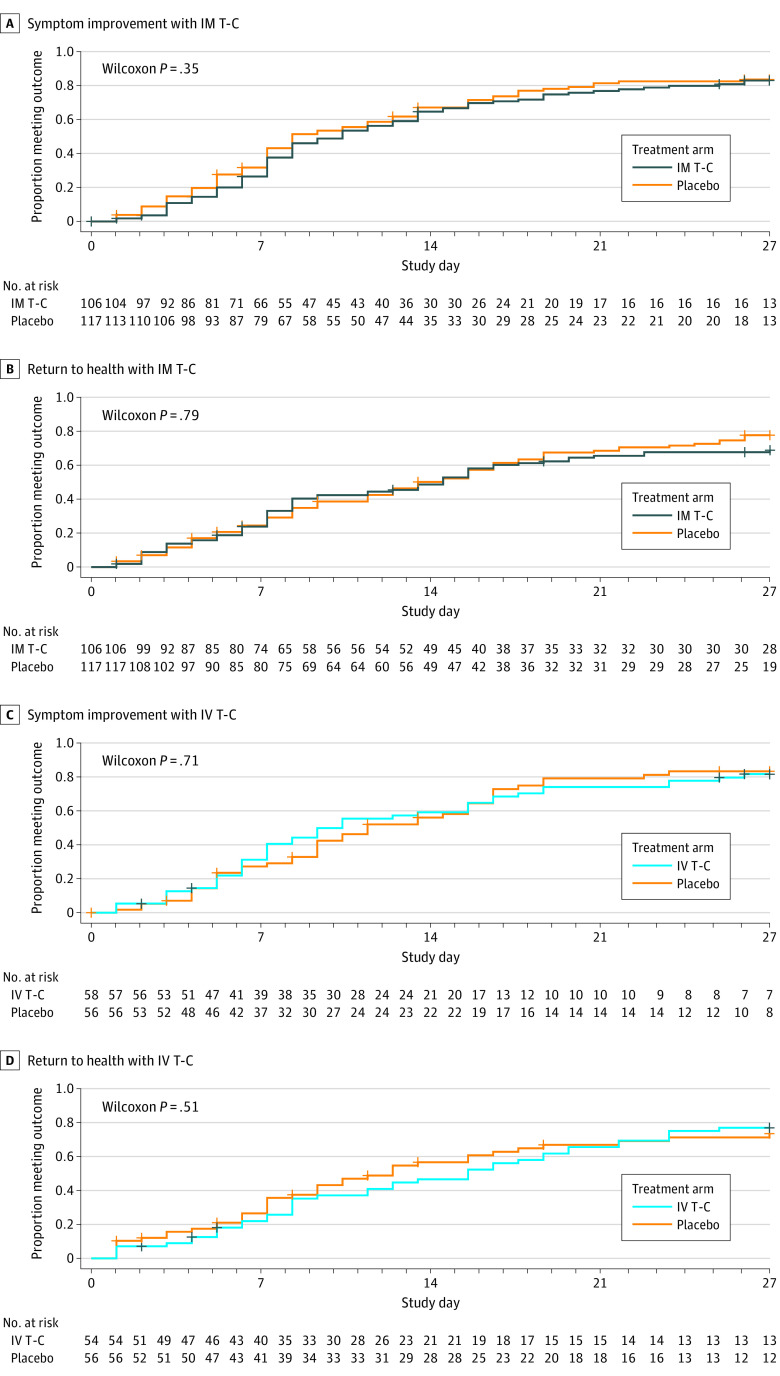
Time to Symptom Improvement and Return to Usual Health Participants include those treated with a tixagevimab-cilgavimab (T-C) combination administered as 600 mg intramuscularly (IM) or placebo or as 300 mg intravenously (IV) or placebo.

Time to symptom improvement was not significantly different between arms, with a median of 8 (95% CI, 7-12) days for IM T-C and 10 (95% CI, 8-13) days for placebo (*P* = .35) (Figure 1A and eTable 1 in Supplement 2). The effects were similar for participants enrolled within 5 days vs more than 5 days after symptom onset (eTable 2 in Supplement 2). Median time to return to health was 14 (95% CI, 8-16) days for the IM T-C arm and 13 (95% CI, 11-16) days for the placebo arm (*P* = .79) (Figure 1B), and there was no difference in total daily symptom scores through day 28 (median area under the curve, 2.2 [IQR, 1.1-4.3] for T-C vs 2.2 [IQR, 1.1-4.1] for placebo; *P* = .87) (eTable 1 in Supplement 2). Four persons (3.8%) were hospitalized in the IM T-C arm and 7 (6.0%) in the placebo arm in the first 28 days (*P* = .54); there were no deaths in either arm (Table 2).

**Table 2.** Adverse Events in IM and IV T-C Groups

Adverse event	Tixagevimab-cilgavimab trial^a^							
	IM administration				IV administration			
	T-C (n = 106)	Placebo (n = 117)	Risk ratio (95% CI)	*P* value^b^	T-C (n = 58)	Placebo (n = 56)	Risk ratio (95% CI)	*P* value^b^
Treatment emergent								
Grade 3 or higher through day 28^c^	9 (8.5)	7 (6.0)	1.42 (0.55-3.68)	.47	3 (5.2)	7 (12.5)	0.41 (0.11-1.52)	.18
Grade 2 or higher through day 28	30 (28.3)	25 (21.4)	1.33 (0.84-2.01)	.23	20 (34.5)	15 (26.8)	1.29 (0.74-2.24)	.38
Serious^d^								
All through day 28	4 (3.8)	7 (6.0)	NA	NA	0	4 (7.1)	NA	NA
COVID-19 pneumonia	4 (3.8)	6 (5.1)	NA	NA	0	3 (5.4)	NA	NA
Gastroenteritis	0	1 (0.)	NA	NA	0	0	NA	NA
Pneumonia bacteria	0	0	NA	NA	0	1 (1.8)	NA	NA
Acute respiratory failure	0	1 (0.9)	NA	NA	0	1 (1.8)	NA	NA
Study drug–related	7 (6.6)	11 (9.4)	NA	NA	4 (6.9)	6 (1.7)	NA	NA
Special interest through day 28	1 (0.9)	1 (0.9)	NA	NA	2 (3.4)	1 (1.8)	NA	NA
Hypersensitivity	1 (0.9)	0	NA	NA	0	0	NA	NA
IRR	0	1 (0.9)	NA	NA	2 (3.4)	1 (1.8)	NA	NA

Abbreviations: IM, intramuscularly; IRR, infusion related reaction; IV, intravenously; NA, not applicable; T-C, study group receiving active treatment with tixagevimab-cilgavimab.

^a^ Unless otherwise indicated, data are expressed as No. (%) of participants. IM administration consists of 600 mg (300 mg of each component) of monoclonal antibody or 600 mg of placebo; IV administration, 300 mg (150 mg of each component) of monoclonal antibody or 300 mg of placebo.

^b^ Calculated using Wald test from Poisson regression with robust variance and log-link; risk ratio comparing T-C group vs placebo.

^c^ Indicates primary outcome.

^d^ All serious adverse events were hospitalizations. There were no deaths in either study. None of the hospitalizations were considered to be study drug related and were primarily progression of COVID-19 respiratory failure.

#### IV Tixagevimab-Cilgavimab Trial

Time to symptom improvement was similar between IV trial arms with a median of 11 (95% CI, 9-15) days for IV T-C and 10 (95% CI, 7-15) days for placebo (*P* = .71) (Figure 1C and eTable 1 in Supplement 2). The median time to return to health was 12 (95% CI, 8-16) days for the IV T-C arm and 15 (95% CI, 9-19) days for the placebo arm (*P* = .51) (Figure 1D). There was no between-arm difference in time-averaged total daily symptom scores through day 28 (median area under the curve, 2.6 [IQR, 1.2-4.2] for T-C vs 2.2 [IQR, 1.3-5.2] for placebo; *P* > .99) (eTable 1 in Supplement 2). There were no hospitalizations in the IV T-C arm and 4 (7.1%) in the placebo arm during the first 28 days (*P* = .06), and no deaths in either arm (Table 2).

### Virologic Outcomes

#### IM Tixagevimab-Cilgavimab Trial

**Figure 2.**
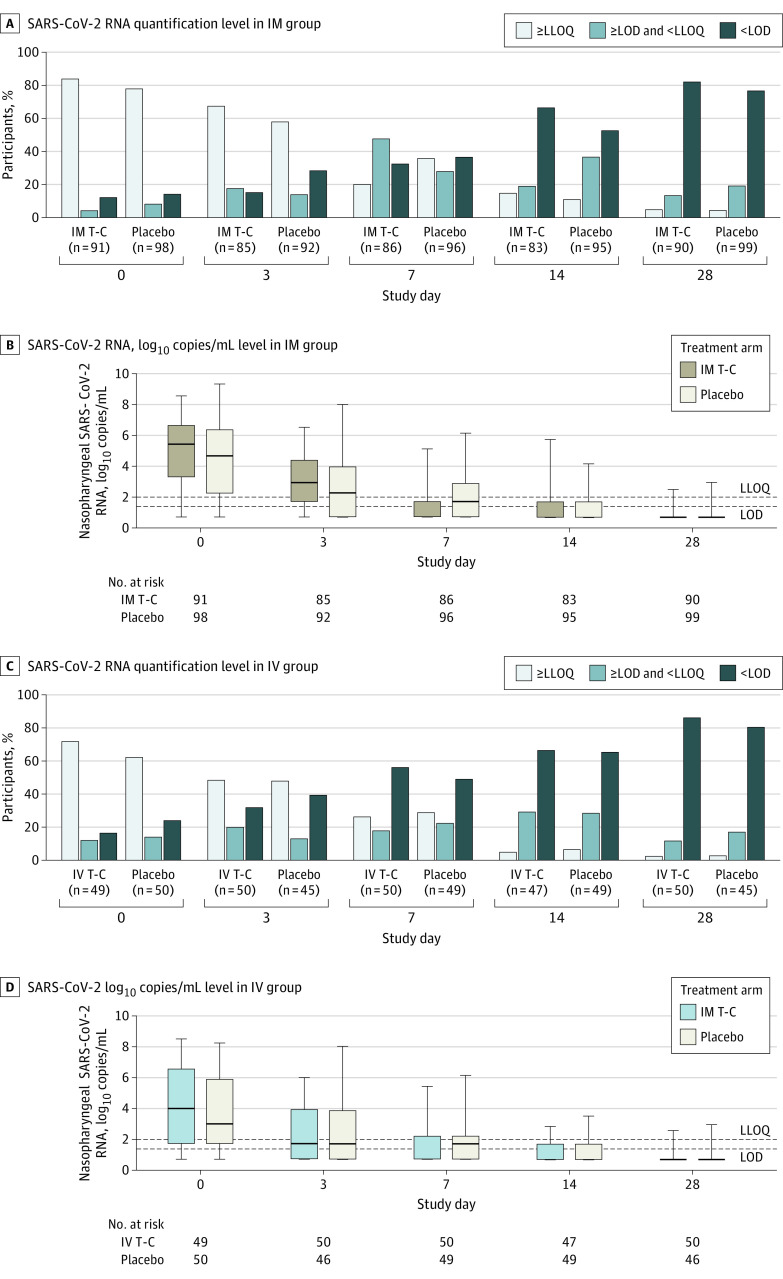
Virologic Outcomes of Tixagevimab-Cilgavimab (T-C) Treatment Proportion of participants with SARS-CoV-2 RNA on nasopharyngeal swabs below the limit of detection (LOD [1.4 log_10_ copies/mL]), at or above the LOD but below the lower limit of quantitation (LLOQ [2.0 log_10_ copies/mL]), or with quantifiable SARS-CoV-2 RNA levels (≥LLOQ) on days 0, 3, 7, 14, and 28 by site-collected nasopharyngeal swab are shown for T-C treatment groups receiving 600 mg intramuscularly (IM) and 300 mg intravenously (IV). SARS-CoV-2 log_10_ copies/mL levels in nasopharyngeal swabs for the T-C and placebo arms are shown at each of the same days in the IM and IV groups. B and D, Middle horizontal lines represent medians; outer horizontal lines of the boxes, IQRs; and whiskers, ranges.

The proportion of participants with unquantifiable nasopharyngeal SARS-CoV-2 RNA differed significantly across days 3, 7, and 14 between the IM T-C and placebo arms after adjusting for pretreatment (day 0) values (joint *P* = .003) (Table 3). This primarily reflected a higher proportion in the IM T-C arm (69 of 86 [80.2%]) than the placebo arm (62 of 96 [64.6%]) with SARS-CoV-2 RNA below LLOQ at day 7 (adjusted RR, 1.33 [95% CI, 1.12-1.57]). At day 3 the proportion with SARS-CoV-2 RNA below LLOQ was 28 of 85 participants (32.9%) in the IM T-C arm vs 39 of 92 (42.4%) in the placebo arm (adjusted RR, 0.75 [95% CI, 0.52-1.10]), and at day 14 it was 71 of 83 (85.5%) in the IM T-C arm vs 85 of 95 (89.5%) in the placebo arm (adjusted RR, 1.03 [95% CI, 0.90-1.18]) (Figure 2A). The difference in quantifiable virus at day 7 was observed in those enrolled both within and more than 5 days since symptom onset, although the estimated effect was greater in those enrolled within 5 days (eTable 3 in Supplement 2). Nasopharyngeal SARS-CoV-2 RNA levels did not differ between arms at any individual time point (Table 3), and in a post hoc analysis the change in log_10_ RNA from baseline to day 3 was not different between arms (Figure 2B and eTable 4 in Supplement 2); later visits were not evaluated due to high levels of unquantifiable RNA. The proportion of participants with unquantifiable anterior nasal SARS-CoV-2 RNA across days 1 to 14 was different between the IM T-C and placebo arms (*P* = .01), with higher proportions below the LLOQ in the T-C arm on days 5 and 7 (eTable 5 in Supplement 2).

**Table 3.** Primary and Secondary Virologic Outcomes Among Persons Randomized to IM or IV T-C or Placebo

Outcome	Tixagevimab-cilgavimab trial								
	IM administration^a^					IV administration^a^			
	T-C (n = 106)	Placebo (n = 117)	aRR (95% CI)^b^	Overall *P* value^c^	T-C (n = 58)		Placebo (n = 56)	aRR (95% CI)^b^	Overall *P* value^c^
**Primary virology: proportion with nasopharyngeal SARS-CoV-2 RNA <LLOQ**									
Day 0									
No. (%)	15 (17)	22 (22)	NA	NA	14 (29.2)		19 (38.0)	NA	NA
No. missing	16	19			10		6		
Day 3				.003					.49
No. (%)	28 (32.9)	39 (42.4)	0.75 (0.52 to 1.10)		26 (52.0)		24 (52.2)	0.93 (0.69 to 1.26)	
No. missing	21	25			8		10		
Day 7									
No. (%)	69 (80.2)	62 (64.6)	1.33 (1.12 to 1.57)		37 (74.0)		35 (71.4)	1.12 (0.93 to 1.35)	
No. missing	20	21			8		7		
Day 14									
No. (%)	71 (85.5)	85 (89.5)	1.03 (0.90 to 1.18)		45 (95.7)		46 (93.9)	1.10 (0.94 to 1.29)	
No. missing	23	22			11		7		
**Secondary virology: quantitative nasopharyngeal SARS-CoV-2 RNA, log_10_ copies/mL**									
Day 0									
Median (IQR)	5.43 (3.31 to 6.63)	4.67 (2.24 to 6.35)	NA	NA^d^	3.91 (<LLOQ to 6.54)		3.00 (<LLOQ to 5.87)	NA	NA^d^
No. missing	16	19			10		6		
Day 3									
Median (IQR)	2.94 (<LLOQ to 4.38)	2.26 (<LOD to 3.95)	NA	.17^d^	<LLOQ (<LOD to 3.94)		<LLOQ (<LOD to 3.89)	NA	.96^d^
No. missing	21	25			8		10		
Day 7									
Median (IQR)	<LLOQ (<LOD to <LLOQ)	<LLOQ (<LOD to 2.88)	NA	.31^d^	<LOD (<LOD to 2.20)		<LLOQ (<LOD to 2.21)	NA	.46^d^
No. missing	20	21			8		7		
Day 14									
Median (IQR)	<LOD (<LOD to <LLOQ)	<LOD (<LOD to <LLOQ)	NA	.16^d^	<LOD (<LOD to <LLOQ)		<LOD (<LOD to <LLOQ)	NA	.90^d^
No. missing	23	22			11		7		

Abbreviations: aRR, adjusted risk ratio; LLOQ, lower limit of quantification (2.0 log_10_ copies/mL); LOD, limit of detection (1.4 log_10_ copies/mL); NA, not applicable; T-C, study group receiving active treatment with tixagevimab-cilgavimab.

^a^ IM administration consists of 600 mg (300 mg of each component) of monoclonal antibodies or 600 mg of placebo; IV administration, 300 mg (150 mg of each component) of monoclonal antibodies or 300 mg of placebo.

^b^ Compares proportion of participants with SARS-CoV-2 RNA below LLOQ for tixagevimab-cilgavimab vs placebo, using a modified Poisson regression model adjusted for baseline log_10_ SARS-CoV-2 RNA level, and an independent working correlation structure with robust SEs for repeated measurements. Corresponding 95% CI for the risk ratio from the generalized estimating equation fit.

^c^ Calculated using the 2-sided Wald test.

^d^ Calculated using the 2-sided Wilcoxon rank sum test.

#### IV Tixagevimab-Cilgavimab Trial

There were no significant differences in proportions with quantifiable nasopharyngeal SARS-CoV-2 RNA or quantitative levels at days 3, 7, or 14 (Table 3 and Figure 2C and D). The post hoc analysis for change in log_10_ RNA from baseline to day 3 showed faster declines for the IV T-C compared with placebo arms (mean difference, −0.98 log_10_ copies/mL [95% CI, −1.81 to −0.13 log_10_ copies/mL]; *P* = .02) (eTable 4 in Supplement 2). Later visits were not evaluated due to high levels of unquantifiable RNA. Although there was no overall between-arm difference in the proportion with unquantifiable anterior nasal SARS-CoV-2 RNA across days 1 to 14, the IV T-C arm had a higher proportion with levels below LLOQ on days 5 and 6 (eTable 5 in Supplement 2).

### Safety

#### IM Tixagevimab-Cilgavimab Trial

Grade 3 or higher TEAEs were reported in 9 participants (8.5%) in the IM T-C arm and 7 (6.0%) in the placebo arm (RR, 1.42 [95% CI, 0.55-3.68]; *P* = .47). Grade 2 or higher TEAEs were reported in 30 participants (28.3%) and 25 (21.4%) in the IM and placebo arms, respectively (Table 2 and eTable 6 in Supplement 2). There were 11 serious adverse effects across both arms, but none were considered related to treatment. The arms had similar numbers of study drug–related adverse effects (7 [6.6%] vs 11 [9.4%]), and 1 participant in each arm had an adverse effect of special interest—a grade 2 hypersensitivity-type reaction in a tixagevimab-cilgavimab recipient and a grade 1 infusion-related reaction in a pooled placebo recipient; neither of these were serious. There were no moderate or severe injection site reactions.

#### IV Tixagevimab-Cilgavimab Trial

Grade 3 or higher TEAEs occurred in 3 participants (5.2%) in the IV T-C arm and 7 (12.5%) in the placebo arm (RR, 0.41 [95% CI, 0.11-1.52]; *P* = .18); grade 2 or higher TEAEs occurred in 20 (34.5%) and 15 (26.8%) participants, respectively. A similar number of study drug–related adverse events occurred in each arm (4 [6.9%] vs 6 [10.7%]), none of which were serious; there were 4 unrelated serious adverse events in the placebo arm. There were 2 infusion-related reactions in the IV T-C arm vs 1 in the placebo arm; all participants completed their infusion.

## Discussion

In these 2 phase 2 randomized clinical trials conducted prior to the emergence of the SARS-CoV-2 Delta and Omicron variants in a predominantly unvaccinated population with a median symptom duration of 6 days at the time of treatment, a single 600-mg IM dose of tixagevimab-cilgavimab to the thigh was safe but did not lead to improvement in symptom outcomes among adult outpatients with mild-to-moderate COVID-19 compared with placebo. Within the smaller group administered a 300-mg IV dose of tixagevimab-cilgavimab or placebo, there was a similar absence of benefit on symptoms. However, an antiviral effect of tixagevimab-cilgavimab is supported by the primary virologic analysis in the IM group and in secondary and post hoc analyses of both groups. There were fewer hospitalizations in both IM and IV T-C arms compared with placebo arms, but the study had limited power to evaluate effects.^21^

Intramuscular tixagevimab-cilgavimab administered in the vastus lateralis muscle of the thigh was safe and well-tolerated. Absorption of drugs administered to the thigh is expected to be quicker with less interperson variability compared with injection into gluteal musculature, especially in women and those with higher body mass index.^22^ Only gluteal IM administration was evaluated in prior trials of tixagevimab-cilgavimab and sotrovimab.^14,21,23,24^ Previously reported pharmacokinetic results from the ACTIV-2 study^25^ support thigh administration of this mAb combination, although the time to reach the expected effective concentration could be delayed; 300 mg IV and 600 mg IM administered in the thigh achieved equivalent concentrations by 72 hours. It is notable that given these considerations and greater convenience, studies of newer SARS-CoV-2 mAbs include thigh administration.^26^

The virologic findings from this study complement those from the placebo-controlled phase 3 TACKLE trial of gluteal IM administration of tixagevimab-cilgavimab,^21^ where a reduction through day 6 in nasopharyngeal SARS-CoV-2 RNA levels was observed compared with placebo. The larger TACKLE study, which recruited unvaccinated individuals a median of 5 days from symptom onset, demonstrated a 50% reduction in progression to severe COVID-19 and death in those administered IM tixagevimab-cilgavimab vs placebo.^21^ In TACKLE, efficacy against COVID-19 progression decreased from 88% when administered within 3 days of symptom onset to 67% within 5 days of symptoms. More than 55% of both ACTIV-2 IM and IV groups enrolled more than 5 days after symptom onset, and 25% after more than 7 days.

We found a significant decline in SARS-CoV-2 RNA from baseline to day 3 in the post hoc analysis for IV but not IM tixagevimab-cilgavimab compared with placebo. It is possible the more rapid achievement of effective mAb concentrations with IV compared with IM administration could also provide greater protection from disease progression, depending on the timing of treatment. Drugs with demonstrated antiviral effect have consistently shown effect on hospitalizations, while clinical benefits have also been observed in the absence of effect on nasopharyngeal RNA.^3,27,28^ Drugs with antiviral effect have also failed to shorten symptoms in lower-risk populations.^29^ In studies comparing IV vs IM or subcutaneous administration of 2 other anti–SARS-CoV-2 mAbs, lower point estimates for hospitalizations were observed for IV administration, although outcomes were not statistically different from the alternate route of administration.^24,25,30^

### Limitations

While the randomized placebo-controlled design, standardized outcome measures, and diverse population are strengths of our study, there are limitations. Foremost, phase 2 studies within ACTIV-2 were powered for the primary virologic end point and were not designed to detect differences in rates of hospitalization or death. As age is a major risk factor for COVID-19 progression, the younger age of participants in this study could have contributed to lower hospitalization rates, further limiting evaluation of this end point. Although only higher-risk participants were eligible for the IV group, and the effect of the study agent on COVID-19 progression appeared stronger in that group, which was older than the IM group, the hospitalization rate across the IM and IV placebo arms was similar. Enrollment into the IV group was truncated, which reduced statistical power and our ability to detect signals in the primary analyses.

Enrollment into ACTIV-2 occurred prior to the widespread circulation of the Delta variant, and this mAb combination has reduced neutralization against Omicron subvariants.^19,31^ Doses for these trials were selected for the ancestral SARS-CoV-2 strain. However, short delays to reach effective concentration after IM administration could impact efficacy, and this could be compounded by the longer time from symptom onset to enrollment. The potential drawbacks of longer time to effective concentration with IM administration could be offset by the ease of administration, which could alleviate the access bottlenecks and inequities observed with IV COVID-19 treatments.^32,33^

## Conclusions

Results of these 2 randomized clinical trials indicate that tixagevimab-cilgavimab administered IM to the thigh was safe but neither IM nor IV treatment had significant effects on measured symptom outcomes. An antiviral effect of tixagevimab-cilgavimab was demonstrated in the primary virologic analysis for IM and other analyses in the truncated IV study. There were numerically fewer hospitalizations with receipt of tixagevimab-cilgavimab.

Therapeutic options for COVID-19 remain limited. At present, there are no mAbs available with activity against the circulating SARS-CoV-2 variants. Our findings support IM thigh administration of mAbs, a route that should be considered in development of mAbs for SARS-CoV-2 infection.

## Notes

Supplement 1.
Trial Protocol and Statistical Analysis Plan

Supplement 2. **eMethods 1.** Participant Enrollment and Study Analysis **eFigure.** CONSORT Diagram **eTable 1.** Clinical Outcomes Amongst Persons Randomized to Receive Tixagevimab-Cilgavimab, 600 mg IM or 300 mg IV, or Placebo **eTable 2.** Clinical Outcomes Stratified by Symptom Duration Prior to Randomization **eTable 3.** Number and Proportion of Participants With SARS-CoV-2 RNA Levels Below and at or above the LLOQ From NP Swabs by Duration of Symptoms Prior to Enrollment (≤ 5 or >5 d), Amongst Those in the Tixagevimab-Cilgavimab, 600 mg IM and 300 mg IV, Groups **eTable 4.** Change in NP RNA From Day 0 to Day 3 Among Those With NP RNA at Day 0 Greater Than LLOQ **eTable 5.** Number and Proportion of Participants With Unquantifiable SARS-CoV-2 RNA Levels From Participant Self-collected Anterior Nasal (AN) Swabs Amongst Persons in the Tixagevimab-Cilgavimab, 600 mg IM and 300 mg IV, Groups **eTable 6.** Number of Participants With Adverse Events Grade 2 or Higher Through Day 28 **eTable 7.** Variants Among Persons Randomized to Either IV or IM Tixagevimab-Cilgavimab or Placebo Groups **eMethods 2.** Site Acknowledgments

Supplement 3.
Nonauthor Collaborators

Supplement 4.
Data Sharing Statement
