# Supplementary material for: Safety and Efficacy of Combined Tixagevimab and Cilgavimab Administered Intramuscularly or Intravenously in Nonhospitalized Patients With COVID-19: 2 Randomized Clinical Trials
Source: JAMA Netw Open. 2023 Apr 26;6(4):e2310039. doi: 10.1001/jamanetworkopen.2023.10039 (PMC10134004; doi:10.1001/jamanetworkopen.2023.10039)
Supplement: Supplement 1. — Trial Protocol and Statistical Analysis Plan [file jamanetwopen-e2310039-s001.pdf]

Safety and Efficacy of Tixagevimab/Cilgavimab Administered Intramuscularly or Intravenously in Non-Hospitalized Patients with COVID-19: Two Randomized Phase 2 Trials

Section 1 **Final Protocol Version 5.0**

Section 2 **Enrollment Criteria Changes and High Risk Criteria Changes from Version 3.0 to Version 5.0**

Section 3 **Statistical Analysis Plan 7.0**

Section 1. Final Protocol Version 5.0

ACTIV-2/A5401

Adaptive Platform Treatment Trial for Outpatients with COVID-19 (Adapt Out COVID)

A Multicenter Trial of the AIDS Clinical Trials Group (ACTG)

Sponsored by:
National Institute of Allergy and Infectious Diseases

Industry Support Provided by:

CBER IND #27230
SAB Biotherapeutics

CDER IND #151193
AstraZeneca
Brii Biosciences
Bristol Myers Squibb
Lilly Research Laboratories, Eli Lilly and Company
Sagent Pharmaceuticals
Synairgen

Protocol Co-Chairs: Kara Chew, MD, MS
David (Davey) Smith, MD, MAS

Protocol Vice Chairs: Eric Daar, MD
David Wohl, MD

DAIDS Clinical Representative: Arzhang Cyrus Javan, MD, MPH, DTM&H

Clinical Trials Specialists: Lara A. Hosey, MA, CCRP
Jhoanna C. Roa, MD

FINAL Version 5.0
April 2, 2021

ACTG
AIDS CLINICAL TRIALS GROUP

ACTIV-2/A5401

Adaptive Platform Treatment Trial for Outpatients with COVID-19
(Adapt Out COVID)

SIGNATURE PAGE

I will conduct the study in accordance with the provisions of this protocol and all applicable protocol-related documents. I agree to conduct this study in compliance with United States (US) Health and Human Service regulations (45 CFR 46); applicable US Food and Drug Administration regulations; standards of the International Conference on Harmonization Guideline for Good Clinical Practice (E6); Institutional Review Board/Ethics Committee determinations; all applicable in-country, state, and local laws and regulations; and other applicable requirements (e.g., US National Institutes of Health, Division of AIDS) and institutional policies.

The following study agents are included in this version of the protocol. Sites are expected to participate in all available study agents.

Initial each agent below to confirm site participation. If not participating in an agent, mark that agent with an N/A.

____BAMLANIVIMAB INTRAVENOUS ADMINISTRATION

____BRII-196 and BRII-198 INTRAVENOUS ADMINISTRATION

____AZD7442 INTRAVENOUS ADMINISTRATION

____AZD7442 INTRAMUSCULAR ADMINISTRATION

____SNG001 INHALATION ADMINISTRATION

____CAMOSTAT ORAL ADMINISTRATION

____SAB-185 INTRAVENOUS ADMINISTRATION

____ **BMS-986414 and BMS-986413 SUBCUTANEOUS ADMINISTRATION**

Principal Investigator: ____
Print/Type

Signed: ___________________________________ Date: _____________
Name/Title

TABLE OF CONTENTS

Page

SIGNATURE PAGE 3

TABLE OF CONTENTS 4

SITES PARTICIPATING IN THE STUDY 7

PROTOCOL TEAM ROSTER 8

STUDY MANAGEMENT 13

GLOSSARY OF PROTOCOL-SPECIFIC TERMS 14

SCHEMA 15

1.0 STUDY OBJECTIVES 18
- 1.1 Co-Primary Objectives 18
- 1.2 Secondary Objectives 18
- 1.3 Exploratory Objectives 18

2.0 INTRODUCTION 19
- 2.1 Background 19
- 2.2 Rationale 21

3.0 STUDY DESIGN 22
- 3.1 Overview of Study Design 22
- 3.2 Infused Agents: Overview of Study Design for Graduation from Phase II to Phase III 24
- 3.3 Considerations Regarding the Use of Placebos and the Sharing of Placebo Groups for Evaluating Multiple Investigational Agents 27

4.0 SELECTION AND ENROLLMENT OF PARTICIPANTS 29
- 4.1 General Eligibility Criteria 29
- 4.2 Study Enrollment Procedures 31
- 4.3 Co-enrollment Guidelines 32

5.0 INVESTIGATIONAL AGENT 32
- 5.1 Regimen, Administration, and Duration 33
- 5.2 Formulation, Storage, and Preparation 33
- 5.3 Supply, Distribution, and Accountability 33
- 5.4 Concomitant Medications 33

6.0 CLINICAL AND LABORATORY EVALUATIONS 34
- 6.1 Schedule of Evaluations 34
- 6.2 Timing of Evaluations 39
- 6.3 Instructions for Evaluations 39

7.0 ADVERSE EVENTS AND STUDY MONITORING 46
- 7.1 Definitions of Adverse Events 47
- 7.2 Eliciting and Documenting Adverse Events 47
- 7.3 Recording Adverse Events 48
- 7.4 Follow-up of Participants Reporting Adverse Events 50
- 7.5 Study Monitoring 50

8.0 CLINICAL MANAGEMENT ISSUES 51
- 8.1 Toxicity 51
- 8.2 Management of Side Effects 51
- 8.3 Pregnancy 52
- 8.4 Breastfeeding 52

CONTENTS (Cont'd) Page

9.0 CRITERIA FOR DISCONTINUATION 52
9.1 Permanent and Premature Treatment Discontinuation 52
9.2 Premature Study Discontinuation 52

10.0 STATISTICAL CONSIDERATIONS 53
10.1 General Design Issues 53
10.2 Outcome Measures 53
10.3 Randomization and Stratification 56
10.4 Sample Size 57
10.5 Data and Safety Monitoring 60
10.6 Analyses 63
10.7 Unblinding 65

11.0 PHARMACOLOGY PLAN 65

12.0 DATA COLLECTION AND MONITORING 66
12.1 Data Quality Assurance 66
12.2 Records to Be Kept 66
12.3 Role of Data Management 66
12.4 Clinical Site Monitoring and Record Availability 67

13.0 PARTICIPANTS 67
13.1 Institutional Review Board (IRB) Review and Informed Consent 67
13.2 Ethical Conduct of Study 68
13.3 Participant Information and Consent 68
13.4 Participant Confidentiality 68
13.5 Study Discontinuation 68

14.0 PUBLICATION OF RESEARCH FINDINGS 68

15.0 BIOHAZARD CONTAINMENT 68

16.0 REFERENCES 70

APPENDIX I: SAMPLE INFORMED CONSENT – MAIN PROTOCOL 71

APPENDIX II: INVESTIGATIONAL AGENT BAMLANIVIMAB ..... **Error! Bookmark not defined.**

APPENDIX III: SAMPLE INFORMED CONSENT FOR STUDY DRUG BAMLANIVIMAB ... **Error! Bookmark not defined.**

APPENDIX IV: INVESTIGATIONAL AGENTS, BRII-196 + BRII 198 ..... **Error! Bookmark not defined.**

APPENDIX V: SAMPLE INFORMED CONSENT FOR STUDY DRUGS BRII-196 AND BRII-198 ..... **Error! Bookmark not defined.**

APPENDIX VI: INVESTIGATIONAL AGENT AZD7442 INTRAVENOUS ADMINISTRATION 85

APPENDIX VII: SAMPLE INFORMED CONSENT FOR STUDY DRUG AZD7442 ADMINISTERED VIA INTRAVENOUS INFUSION 105

APPENDIX VIII: INVESTIGATIONAL AGENT AZD7442 INTRAMUSCULAR ADMINISTRATION 110

APPENDIX IX: SAMPLE INFORMED CONSENT FOR STUDY DRUG AZD7442 ADMINISTERED AS AN INTRAMUSCULAR INJECTION 127

APPENDIX X: INVESTIGATIONAL AGENT INHALED INTERFERON-β1a (SNG001) ...**Error! Bookmark not**

CONTENTS (Cont'd) Page

defined.

APPENDIX XI: SAMPLE INFORMED CONSENT FOR STUDY DRUG SNG001 **Error! Bookmark not defined.**

APPENDIX XII: INVESTIGATIONAL AGENT CAMOSTAT............................... **Error! Bookmark not defined.**

APPENDIX XIII: SAMPLE INFORMED CONSENT FOR STUDY DRUG CAMOSTAT ....**Error! Bookmark not defined.**

APPENDIX XIV: SAB-185 ANTI-SARS-COV-2 HUMAN IMMUNOGLOBULIN INTRAVENOUS (TC BOVINE-DERIVED) .... **Error! Bookmark not defined.**

APPENDIX XV: SAMPLE INFORMED CONSENT FOR STUDY DRUG SAB-185...........**Error! Bookmark not defined.**

APPENDIX XVI: INVESTIGATIONAL AGENT BMS-986414 and BMS-986413...........**Error! Bookmark not defined.**

APPENDIX XVII: SAMPLE INFORMED CONSENT FOR STUDY DRUGS BMS-986414 and BMS-986413.. **Error! Bookmark not defined.**

APPENDIX XVI**II:** SIGNATURE PAGE – STUDY DRUGS.. 131

SITES PARTICIPATING IN THE STUDY

ACTIV-2/A5401 is a multicenter study open to select US- and non-US-based clinical research sites.

PROTOCOL TEAM ROSTER

Co-Chairs
Kara Chew, MD, MS
Clinical AIDS Research and Education
University of California, Los Angeles
911 Broxton Avenue, Suite 200
Los Angeles, CA 90024
Phone: 310-825-0796
Fax: 310-477-7657
E-mail: kchew@mednet.ucla.edu

David (Davey) Smith, MD, MAS
Antiviral Research Center
University of California, San Diego
220 Dickinson Street
San Diego, CA 92103
Phone: 619-543-7449
Fax: 619-298-0177
E-mail: d13smith@health.ucsd.edu

Vice Chairs
Eric Daar, MD
Harbor University of California Los Angeles Center
CRS, CDCRC Building
1124 West Carson Street
Torrance, CA 90502
Phone: 424-201-3000
Fax: 310-533-0447
E-mail: edaar@lundquist.org

David Wohl, MD
Chapel Hill CRS,
Suite 2100, Bioinformatics Building
130 Mason Farm Road
Chapel Hill, NC 27514
Phone: 919-843-2723
Fax: 919-966-8928
E-mail: wohl@med.unc.edu

DAIDS Clinical Representative
Arzhang Javan, MD, MPH, DTM&H
TRP, DAIDS, NIAID, NIH
5601 Fishers Lane
Room 9E40, MSC 9830
Rockville, MD 20852
Phone: 301-761-7710
Mobile: 240-475-6683
E-mail: arzhang.javan@nih.gov

Clinical Trials Specialists
Lara Hosey, MA
ACTG Network Coordinating Center
Social & Scientific Systems, a DLH Company
8757 Georgia Avenue, 12th Floor
Silver Spring, MD 20910
Phone: 301-628-3395
Fax: 301-628-3302
E-mail: lara.hosey@dlhcorp.com

Jhoanna C. Roa, MD
ACTG Network Coordinating Center
Social & Scientific Systems, a DLH Company
8757 Georgia Avenue, 12th Floor
Silver Spring, MD 20910-3714
Phone: 301-628-3196
Fax: 301-628-3302
E-mail: jhoanna.roa@dlhcorp.com

Statisticians
Mark Giganti, PhD
Center for Biostatistics in AIDS Research
Harvard T.H. Chan School of Public Health
FXB Building, Room 603
Boston, MA 02115
Phone: 617-432-3233
Fax: 617-432-3163
E-mail: mgiganti@sdac.harvard.edu

Statisticians (Cont'd)
Michael Hughes, PhD
Statistical and Data Analysis Center
Harvard T.H. Chan School of Public Health
Building 2, Room 439A, 655 Huntington Ave
Boston, MA 02115-6017
Phone: 617-432-3161
Fax: 617-432-2832
E-mail: mhughes@sdac.harvard.edu

Carlee Moser, PhD
Center for Biostatistics in AIDS Research
Harvard T.H. Chan School of Public Health
FXB Building, Room 513
Boston, MA 02115
Phone: 617-432-2526
Fax: 617-432-2829
E-mail: cmoser@sdac.harvard.edu

Justin Ritz, MS
Center for Biostatistics in AIDS Research
Harvard T.H. Chan School of Public Health FXB
Building, Room 510
Boston, MA 02115
Phone: 617-432-3034
Fax: 617-432-3163
E-mail: jritz@sdac.harvard.edu

DAIDS Pharmacists
Kelly Parsons, PharmD
Pharmaceutical Affairs Branch
DAIDS, OCSO, PAB
5601 Fishers Lane, 9E15
Rockville, MD 20852
Phone: 240-669-5721
E-mail: kelly.parsons@nih.gov

Justine Beck, Pharm.D
NIAID/NIH/DHHS/PAB
5601 Fishers Lane
Room 9D39
Bethesda, MD 20892
Phone: 301-761-5288
Fax: 240-627-3112
E-mail: justine.beck@nih.gov

Immunologist
Scott Sieg, PhD
Case Western Reserve University
Biomedical Research Building
Room 1020
2109 Adelbert Road
Cleveland, OH 44106-4984
Phone: 216-368-6594
Fax: 216-368-5415
E-mail: sfs2@case.edu

Virologist
Jonathan Li, MD, MMSc
Brigham and Women's Hospital Therapeutics
(BWHT) CRS
Harvard Medical School
Department of Infectious Diseases
65 Landsdowne Street, Room 421
Boston, MA 02139
Phone: 617-768-8476
Fax: 617-768-8738
E-mail: jli@bwh.harvard.edu

Pharmacologist
Courtney Fletcher, PharmD
University of Nebraska Medical Center
College of Pharmacy
986000 Nebraska Medical Center
Omaha, NE 68198-6000
Phone: 402-559-4333
Email: cfletcher@unmc.edu

Critical Care Specialist
William Fischer, MD
Division of Pulmonary and Critical Care Medicine
University of NC School of Medicine
Phone: 410-908-7779
Email: WFischer@med.unc.edu

Investigators
Judith Currier, MD, MSc
Clinical AIDS Research and Education (CARE) Center CRS
911 Broxton Avenue, Suite 200
Los Angeles, CA 90024
Phone: 310-825-9283
Fax: 310-477-7657
E-mail: jscurrier@mednet.ucla.edu

Joseph Eron, MD
University of North Carolina Global HIV Prevention and Treatment CTU
Bioinformatics Building
130 Mason Farm Road, Suite 210
Chapel Hill, NC 27599-7215
Phone: 919-843-2722
Fax: 919-966-6714
E-mail: jeron@med.unc.edu

Teresa Evering, MD, MS
Weill Cornell Medicine
Uptown CRS
1300 York Avenue
New York, NY 10065
E-mail: evering@med.cornell.edu

Rachel Bender Ignacio, MD, MPH
University of Washington AIDS Clinical Trials Unit CRS
325 9th Ave
Harborview Medical Center
P.O. Box 359929
Seattle, WA 98104
Phone: 206-940-5522
E-mail: rbi13@uw.edu

Prasanna Jagannathan, MD
Division of Infectious Diseases and Geographic Medicine
Stanford University
240 Pasteur Drive
Biomedical Innovations Building
3rd Floor, Room 3456
Phone: 650-724-5343
Email: prasj@stanford.edu
Investigators (Cont'd)
Nikolaus Jilg, MD, PhD
Massachusetts General Hospital/
Harvard Medical School
55 Fruit Street
Boston, MA 02114
Phone: 617-416-7955
Fax: 617-726-7653
E-mail: njilg@partners.org

Alan Landay, PhD
Division of Geriatrics, Gerontology and Palliative Medicine
Rush Medical College
1735 West Harrison Street, Suite 306 POB1
Chicago, IL 60612
Phone: 312-942-2849
E-mail: alan_landay@rush.edu

Upinder Singh, MD
Departments of Internal Medicine and Microbiology and Immunology
Stanford University School of Medicine
Lane Building, Suite 134, Rm 121
300 Pasteur Drive
Stanford, CA 94305-5107
Phone: (650) 723-4045
Fax: (650) 724-3892
E-mail: usingh@stanford.edu

Babafemi Taiwo, MBBS, MD
Northwestern University CRS
Feinberg School of Medicine
Division of Infectious Diseases
645 North Michigan Avenue, Suite 900
Chicago, IL 60611
Phone: 312-695-4994
Fax: 312-695-5088
E-mail: b-taiwo@northwestern.edu

TEAM ROSTER (Cont'd)

Field Representatives
Joan Gottesman, BSN, RN, CCRP
Vanderbilt Therapeutics (VT) CRS
One Hundred Oaks
719 Thompson Lane, Suite 47183
Nashville, TN 37204
Phone: 615-936-7143
Fax: 615-936-2644
E-mail: joan.gottesman@vumc.org

Matthew Newell, BSN, RN, CCRN
Chapel Hill CRS
Campus Box 7215
Bioinformatics Suite 2100
130 Mason Farm Road
Chapel Hill, NC 27599-7215
Phone: 919-843-6929
Fax: 919-966-8928
E-mail: matthew_newell@med.unc.edu

Laboratory Technologists
Joan Dragavon, MLM
University of Washington AIDS Clinical Trials Unit,
Retrovirology Laboratory
Ninth & Jefferson Building, Room 342.7
908 Jefferson Street
Seattle, WA 98104
Phone: 206-897-5210
E-mail: dragavon@uw.edu

Cheryl Jennings, BS
Northwestern University CRS
Clinical Retrovirology Research Laboratory
1181 Jelke Building
1750 West Harrison Street
Chicago, IL 60612
Phone: 312-942-5954
Fax: 312-942-6787
E-mail: cheryl_jennings@rush.edu

Community Scientific Subcommittee (CSS)
Representative
Jan Kosmyna, MIS, RN, CCRP
Case CRS, 10041 Brookside Circle
North Royalton, OH 44133
E-mail: jankosmyna@gmail.com

International Site Specialists
Morgan Gapara, MPH
ACTG Network Coordinating Center
Social & Scientific Systems, a DLH Company
4505 Emperor Boulevard, Suite 400
Durham, NC 27703
Phone: 919-287-4503
Fax: 919-941-9349
E-mail: morgan.gapara@dlhcorp.com

Akbar Shahkolahi, PhD
ACTG Network Coordinating Center
Social & Scientific Systems, a DLH Company
8757 Georgia Avenue, 12th Floor
Silver Spring, MD 20910
Phone: 301-628-3318
Fax: 301-628-3302
E-mail: akbar.shahkolahi@dlhcorp.com

Industry Representatives
Robert A Gasser, Jr., MD
Kelly Services, a Service Provider to AstraZeneca
One MedImmune Way
Gaithersburg, MD 20878
Phone 703-242-6259
E-mail: robert.gasserjr@astrazeneca.com

David Margolis, MD, MPH
Brii Biosciences
WeWork One City Center
110 Corcoran St., Durham, NC 27701
Phone: 303-520-1579
E-mail: david.margolis@briibio.com

Industry Representatives (Cont’d)
Edgar Charles, MD
Bristol Myers Squibb
Route 206 & Province Line Road
Lawrenceville, NJ 08543
Phone: 609-252-3759
E-mail: Edgar.Charles@bms.com

Paul Klekotka, MD, PhD
Lilly Research Laboratories
Lilly Biotechnology Center

10290 Campus Point Drive
San Diego, CA 92121
Phone: 619-346-8191
E-mail: klekotka_paul@lilly.com

Rick Finnegan
SAB Biotherapeutics
245 First Street, Suite 1836
Cambridge, MA 02142
Phone: 978-500-3354
E-mail: rfinnegan@sabbiotherapeutics.com

Jake Miles, MBA
SAB Biotherapeutics
2301 E 60th St N
Sioux Falls, SD 57104
Phone: 605-679-6988
E-mail: jmiles@sabbiotherapeutics.com

Bob Szurgot
Sagent
1901 N. Roselle Rd, Suite 450 Schaumburg,
IL 60195
Phone: 847-908-1616
E-mail: bszurgot@SagentPharma.com

Industry Representatives (Cont'd)

Mark J. Main, PhD
Synairgen Research Ltd
Mailpoint 810, Level F
South Block
Southampton General Hospital
Tremona Road
Southampton
SO16 6YD
UK
Phone: +44 7768 415004
E- mail: Mark.Main@synairgen.com

Laboratory Specialist

William A. Murtaugh, MPH
ACTG Laboratory Center
University of California Los Angeles
11075 Santa Monica Boulevard, Suite #200
Los Angeles, CA 90025
Phone: 310-794-9894
E-mail: wmurtaugh@milabcentral.org

STUDY MANAGEMENT

All general questions concerning this protocol and safety and risk management inquiries must be submitted through the electronic Protocol Inquiry Platform (ePIP) system. For urgent ePIPs, following entry into ePIP, contact the following PPD 24/7 global coverage hotline:

24-Hour Study Protocol Queries and Pharmacovigilance Hotline	Telephone Number
North America	1 888 483 7729
Latin America	55 11 4504 4801
Europe, Middle East, and Africa (EMEA) and Asia Pacific (APAC)	44 122 337 4240

Protocol E-mail Group

This protocol will have an email group to allow the study team to communicate directly with staff at participating sites.

Each site must identify the staff members who need to receive study-related information, including announcement of conference calls, and ensure that they are added to the protocol email group, as soon as possible by contacting FSTRF User Support at actg.user.support@fstrf.org. Please note that there is no limit to the number of individuals who can be included in this group. At a minimum, we recommend that the following staff members be included: CRS Leader, Investigator of Record, CRS Coordinator, Pharmacist, Data Manager, and laboratory staff members.

Protocol-Specific Web Page

Additional information about management of the protocol can be found on the protocol-specific web page (PSWP).

GLOSSARY OF PROTOCOL-SPECIFIC TERMS

ACTIV	Accelerating COVID-19 Therapeutic Interventions and Vaccines
AE	adverse event
AESI	adverse event of special interest
AUC	area under the curve
CDMS	Clinical Data Management System
CLIA	Clinical Laboratory Improvement Amendments
COVID-19	coronavirus disease 2019
CRS	clinical research site
DSMB	Data and Safety Monitoring Board
FDA	US Food and Drug Administration
ICU	intensive care unit
IRT	Interactive Response Technology
LPC	lab processing chart
mAb	monoclonal antibody
NP	nasopharyngeal
PBMC	peripheral blood mononuclear cells
SAE	serious adverse event
SAP	statistical analysis plan
SARS-CoV	Severe Acute Respiratory Syndrome coronavirus
SARS-CoV-2	Severe Acute Respiratory Syndrome coronavirus 2
SOE	Schedule of Evaluations
TOC	Trial Oversight Committee

SCHEMA

ACTIV-2 / A5401

Adaptive Platform Treatment Trial for Outpatients with COVID-19
(Adapt Out COVID)

DESIGN

Adapt Out COVID is a master protocol to evaluate the safety and efficacy of investigational agents for the treatment of symptomatic non-hospitalized adults with COVID-19.

The trial is a randomized, blinded, controlled adaptive platform that allows agents to be added and dropped during the course of the study for efficient testing of new agents against placebo within the same trial infrastructure. When two or more new agents are being tested concurrently, the same placebo will be used, if feasible.

The protocol will be amended when information becomes available from within or outside of the trial indicating that further randomization to a placebo is inappropriate.

Version 3 of the protocol will introduce agents that do not require an intravenous infusion (non-infused agents). Thus, the trial will include both infused and non-infused agents. For infused agents, enrollment will be restricted to participants at higher risk of progression to severe COVID-19. Non-infused agents will be open to participants at both “higher” and “lower” risk of progression to severe COVID-19.

For infused agents, the study begins with a phase II evaluation, followed by a transition into a larger phase III evaluation for promising agents. The phase III evaluation is a continuation of the phase II trial for agents that meet study-defined criteria for further evaluation and for which sufficient investigational agent is available. An infused agent may also enter directly into phase III evaluation based on Trial Oversight Committee (TOC) assessments.

For non-infused agents, the same phase II study will be undertaken as for infused agents. However, the design of the phase III evaluation for non-infused agents will be developed in a subsequent version of the protocol. Once developed, non-infused agents may also enter directly into phase III based on TOC assessments.

REGIMEN

Investigational agents will be selected by the TOC for phase II evaluation based on the presence of in vitro data demonstrating promise as anti-SARS-CoV-2 therapeutics in pre-clinical testing and for which there are suitable pharmacokinetics and safety data from phase I testing or through clinical or research testing for a different indication, and agent availability.

DURATION

28 days of intensive follow-up, followed by limited follow-up through 24 weeks. Study visits may be required after week 24, depending on the agent. Details are listed in the agent-specific protocol appendix and consents.

STRATIFICATION

Randomization in both phase II and phase III will be stratified by time from symptom onset (≤5 days versus >5 days). Randomization for non-infused agents will also be stratified by risk of progression to severe COVID-19 (“higher” versus “lower”).

POPULATION

Outpatient adults (≥18 years) with a documented positive SARS-CoV-2 molecular (nucleic acid) or antigen test from a sample collected ≤240 hours (10 days) prior to study entry and with ≤8 days of symptoms of COVID-19 at study entry, plus the presence of select symptoms within 24 hours prior to study entry.

Participants eligible for infused agents will have at least one of the following factors for "higher" risk of progression to severe COVID-19:

- age 60 years and older and no history of SARS-CoV-2 vaccination
- any age with at least one of the following conditions (self-report is acceptable) and no history of SARS-CoV-2 vaccination:
    1. current smoker (cigarette smoking within the past 30 days) AND history of at least 100 lifetime cigarettes
    2. exogenous or endogenous immunosuppression defined as any of the following:
        - HIV infection with CD4 count <200 cells/mm^3
        - receiving corticosteroids equivalent to prednisone ≥20mg daily for at least 14 consecutive days within 30 days prior to study entry
        - treatment with biologics (e.g., infliximab, abalizumab, ustekinumab, etc.), immunomodulators (e.g., methotrexate, 6MP, azathioprine, etc.), or cancer chemotherapy within 90 days prior to study entry
    3. chronic lung disease or asthma requiring daily prescribed therapy
    4. obesity (body mass index [BMI] >35; may be based on self-report of height and weight)
    5. hypertension, with at least one medication recommended or prescribed
    6. cardiovascular disease defined as history of any of the following: myocardial infarction, stroke, transient ischemic attack, heart failure, angina with prescribed nitroglycerin, coronary artery bypass grafts, percutaneous coronary intervention (PCI), carotid endarterectomy, and aortic bypass
    7. diabetes mellitus
    8. chronic kidney disease requiring hemodialysis or peritoneal dialysis
    9. history of cirrhosis
    10. active cancer, other than localized skin cancer

For non-infused agents, participants may be at "higher" or "lower" risk for progression to severe COVID-19.

SCHEMA (Cont'd)

SAMPLE SIZE

Approximately 110 participants per investigational agent (and 110 on placebo) in the phase II evaluation. For infused agents, approximately 421 participants per investigational agent (and 421 on placebo), in the phase III evaluation (including those enrolled in phase II). The sample size for Phase III for non-infused agents will be included in a subsequent version of the protocol.

OUTCOME MEASURES

The primary outcome measures in the phase II evaluation will be duration of symptoms, SARS-CoV-2 RNA below lower limit of quantification by nasopharyngeal (NP) swabs, and safety.

For infused agents, determination of whether an agent in phase II will continue to be evaluated in phase III will be made after the last participant randomized in phase II to that agent or placebo completes their day 28 visit. The fully powered phase III trial will evaluate the efficacy of each selected investigational infused agent compared to placebo to prevent hospitalization and death in non-hospitalized adults with COVID-19.

A subsequent version of the protocol will include a new phase III evaluation of non-infused agents in a broad outpatient population with COVID-19, which will, with the primary outcome, likely be based on a symptom duration outcome measure.

1.0STUDY OBJECTIVES

1.1 Co-Primary Objectives

1.1.1 Phases II and III: To evaluate safety of the investigational agent.

1.1.2 Phase II: To determine efficacy of the investigational agent to reduce the duration of COVID-19 symptoms through study day 28.

1.1.3 Phase II: To determine the efficacy of the investigational agent to increase the proportion of participants with nasopharyngeal (NP) SARS-CoV-2 RNA below the lower limit of quantification (LLoQ) at study days 3, 7, 14, and 28.

1.1.4 Phase III for infused agents only: To determine if the investigational agent will prevent the composite endpoint of either hospitalization or death through study day 28. Hospitalization is defined as ≥24 hours of acute care, in a hospital or similar acute care facility, including Emergency Rooms or temporary facilities instituted to address medical needs of those with severe COVID-19 during the COVID-19 pandemic.

1.2 Secondary Objectives

1.2.1 Phases II and III: To determine whether the investigational agent reduces a COVID-19 Severity Ranking scale based on COVID-19-associated symptom burden (severity and duration), hospitalization, and death, through study day 28.

1.2.2 Phase II and III: To determine whether the investigational agent reduces the progression of COVID-19-associated symptoms.

1.2.3 Phases II and III: To determine if the investigational agent reduces levels of SARS-CoV-2 RNA in nasal swabs.

1.2.4 Phase II: To determine the pharmacokinetics of the investigational agent.

1.2.5 Phase II: To evaluate differences in SARS-CoV-2 RNA levels in NP swabs between the investigational agent versus placebo and among subgroups of the population and risk groups defined by age and comorbidities.

1.2.6 Phase II: To determine efficacy of the investigational agent to obtain pulse oximetry measurement of ≥96% through day 28.

1.2.7 Phase III: To evaluate differences in symptom duration between the investigational agent versus placebo among subgroups of the population, and risk groups defined by age and comorbidities.

1.2.8 Phase III: To determine if the investigational agent will prevent the composite endpoint of either hospitalization or death through study week 24.

1.3 Exploratory Objectives

1.3.1 Phases II and III: To explore the impact of the investigational agent on participant-reported rates of SARS-CoV-2 positivity of household contacts.

1.3.2 Phases II and III: To explore if baseline and follow-up hematology, chemistry, coagulation, viral, and inflammatory biomarkers are associated with clinical and virologic outcomes in relation to investigational agent use.

1.3.3 Phases II and III: To explore possible predictors of outcomes across the study population, notably sex, time from symptom onset to start of investigational agent, race/ethnicity, and risk groups defined by age and comorbidities.

1.3.4 Phases II and III: To explore if the investigational agent changes the hospital course once a participant requires hospitalization.

1.3.5 Phases II and III: To explore and develop a model for the interrelationships between virologic outcomes, clinical symptoms, hospitalization, and death in each study group.

1.3.6 Phases II and III: To explore the relationship between exposure to the investigational agent and SARS-CoV-2 innate, humoral or cellular response, including anti-drug antibodies, as appropriate per investigational agent.

1.3.7 Phases II and III: To explore baseline and emergent viral resistance to the investigational agent.

1.3.8 Phases II and III: To explore the association between viral genotypes and phenotypes, and clinical outcomes and response to agents.

1.3.9 Phases II and III: To explore the association between host genetics and clinical outcomes and response to agents.

1.3.10 Phases II and III: To explore relationships between dose and concentration of investigational agent with virology, symptoms, and oxygenation.

1.3.11 Phase II: To explore the impact of investigational agents on levels of SARS-CoV-2 RNA in the blood.

1.3.12 Phase II: To explore if levels of SARS-CoV-2 RNA in self-collected nasal swabs correlate with levels of SARS-CoV-2 RNA in site-collected NP swabs.

2.0INTRODUCTION

2.1 Background

Virology

Coronaviruses (CoVs) are positive-sense, single-stranded, enveloped RNA viruses, many of which are commonly found in humans and cause mild symptoms. Over the past two decades, emerging pathogenic CoVs capable of causing life-threatening disease in humans and animals have been identified, namely, severe acute respiratory syndrome
coronavirus (SARS-CoV) in 2002-2003 and Middle East Respiratory Syndrome coronavirus (MERS-CoV) in 2012 [1].

New Threat

A novel pneumonia caused by a previously unknown betacoronavirus emerged in Wuhan, China, in December 2019. The virus is closely related to SARS-CoV-1, which caused an outbreak in 2003, and has

been named SARS-CoV-2. The human disease caused by SARS-CoV-2 is called COVID-19.

During the current SARS-CoV-2 outbreak, the incidence of known cases has rapidly increased such that, on January 5, 2020, there were 59 confirmed cases, 278 cases on January 20, 2118 cases on January 26, and more than 80,000 cases and 2700 deaths as of February 25, 2020, according to various international health reporting agencies. As a result, on January 30, 2020, the International Health Regulations Emergency Committee of the World Health Organization (WHO) declared the COVID-19 outbreak a Public Health Emergency of International Concern. On January 31, 2020, the US Department of Health and Human Services declared a public health emergency in the United States. Despite quarantine measures, SARS-CoV-2 has spread to over 188 counties, infecting millions worldwide and killing hundreds of thousands [2]. Outbreak forecasting and modeling suggest that these numbers will continue to rise [3]. Global efforts to evaluate novel antivirals and therapeutic interventions to treat COVID-19 have intensified. There is currently no vaccine to prevent SARS-CoV-2 infection nor any therapeutic agent to treat COVID-19. Therefore, there is an urgent public health need for rapid development of novel interventions.

Disease Course
Once infection occurs, the clinical course is variable. Recent data suggest that fewer than 2.5% of infected persons will show symptoms within 2.2 days (CI, 1.8 to 2.9 days) of exposure, and symptom onset will occur within 11.5 days (CI, 8.2 to 15.6 days) for 97.5% of infected persons [4]. In most (~80%) cases, COVID-19 presents as a mild-to-moderately severe, self-limited acute respiratory illness with fever, cough, and shortness of breath. It remains unclear exactly what the rate of progression of COVID-19 is and what the predictors are for complications, including pneumonia, acute respiratory distress syndrome (ARDS), kidney failure, and death. It is clear that older age, male sex, and comorbidities including diabetes and hypertension increase the risk for worse outcomes [5, 6]. In a recent meta-analysis, the main clinical symptoms were fever (88.5%), cough (68.6%), myalgia or fatigue (35.8%), expectoration (28.2%), and dyspnea (21.9%). Minor symptoms included headache or dizziness (12.1%), diarrhea (4.8%), and nausea and vomiting (3.9%) [7]. Laboratory examinations showed that lymphocytopenia (64.5%), increase of C-reactive protein (CRP) (44.3%), increase of lactate dehydrogenase (LDH) (28.3%), and leukocytopenia (29.4%) were more common in those with COVID-19 [5, 8].

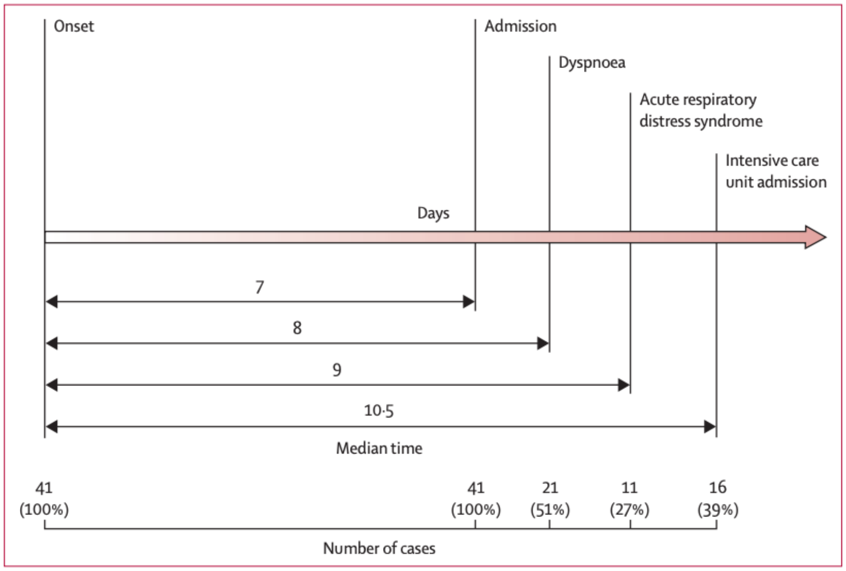

Figure 2.1-1: Timeline of COVID-19 Disease Progression. Onset refers to onset of symptoms.

Shedding
Viral infections jump from host to host through a variety of pathways. Coronaviruses do this through respiratory droplets. Understanding this shedding is important to understanding epidemic spread and how shedding relates to disease progression. Best evidence available now suggests that viral shedding, especially in upper respiratory secretions, is detectable around 2 days before symptoms develop and continues throughout the symptomatic phase. This shedding can be quite high during active disease and can continue for up to 37 days, with a quarter of persons still shedding at 3 weeks, as detected by NP swabs [7].

Biomedical Interventions
Two monoclonal antibody based agents have received emergency use authorization for treatment of COVID-19 in the outpatient setting for high risk persons [9,10]. Full approvals may come soon for these agents, and other agents, as efforts to combat the pandemic progresses. The adenosine analog, remdesivir, has shown clinical benefit for COVID-19 in hospitalized patients, and was approved by the FDA for use in patients requiring hospitalization [4, 5, 11]. Remdesivir must be given intravenously and has a short half-life, and thus is not optimal for an outpatient setting.

New agents are becoming available that may be useful for the treatment of non-hospitalized persons with COVID-19, including anti-SARS-CoV-2 monoclonal antibodies, viral enzyme inhibitors, small interfering RNAs, immune modulators, and other small molecules [12]. Before they can be clinically deployed, they will need to be evaluated quickly in ambulatory persons in a rigorous clinical trial, as will be achieved through ACTIV-2/A5401, the Adapt Out COVID Trial.

2.2 Rationale

There is an urgent need for a platform to rapidly evaluate therapies in the outpatient setting, to prevent disease progression, and reduce serious complications of COVID-19 and transmission [13]. ACTIV-2/A5401 is a phase II/III randomized, blinded, controlled adaptive platform trial to efficiently evaluate agents for the treatment of non-hospitalized persons with COVID-19. This will allow:

- comparison of multiple therapies with a common control group, when feasible, thus potentially requiring fewer participants than in independently conducted randomized controlled trials,
- continuous introduction of new promising agents as they become available,
- generation of separate effect size estimates for each therapy, and
- minimized downtime, with rapid movement of promising agents into phase III evaluation.

Additionally, the trial will facilitate the exploration of virologic endpoints as possible future primary endpoints in COVID-19 trials by assessing the correlation between changes in viral shedding and clinical outcomes.

Outcome Measures
Phase II evaluates the potential effect of an investigational agent on COVID-19-associated symptoms and on viral shedding. However, it is unknown a priori if an investigational agent that is effective in reducing symptom duration and/or viral shedding will have meaningful impact on the clinical outcome of hospitalization or death. Therefore, an investigational agent that has shown preliminary evidence of effects on viral shedding, clinical symptoms, and/or hospitalization/death and has an acceptable safety profile in phase II evaluation will be considered by the Trial Oversight Committee (TOC) for graduation to phase III evaluation (see section 3.0). The TOC is comprised of protocol, ACTG, and NIH Accelerating COVID-19 Therapeutic Interventions and Vaccines (ACTIV) group leadership.

The primary symptom endpoint in phase II and secondary endpoint for infused agents among participants at higher risk for severe COVID-19 in phase III relies on targeted symptoms that have been associated with COVID-19, and which are expected to be dynamic and improve with effective anti-SARS-CoV-2 therapy.

In clinical practice, non-infused agents may have much broader utility because of a simpler mode of administration and availability in more clinical settings. Such treatments would provide greater access to broader populations who have varied risk of severe COVID-19. Thus, a reduction in symptom duration may be an adequate measure for establishing effectiveness of a non-infused agent. Because of this, a subsequent version of the protocol will include a new phase III evaluation of non-infused agents in a broad outpatient population with COVID-19, which will, with the primary outcome, likely be based on a symptom duration outcome measure. The study team has started a discussion with the US Food and Drug Administration about what would be an appropriate phase III primary symptom duration outcome measure for non-infused agents in a broad outpatient population.

<u>Investigational Agents</u>
See appendices for rationale for each investigational agent.

<u>Multi-Site Design</u>
In any multi-site study, outcomes can potentially differ due to variation in site populations, stage of epidemic spread, diagnostic capability, and clinical management. It is expected that any differences between sites will be balanced between arms through randomization.

3.0STUDY DESIGN

3.1Overview of Study Design

Adapt Out COVID is a master protocol to evaluate the safety and efficacy of investigational agents for the treatment of symptomatic non-hospitalized adults with COVID-19. The trial is a randomized, blinded, controlled adaptive platform that allows investigational agents to be added and dropped during the course of the study for efficient testing of new agents against placebo within the same trial infrastructure [13]. This protocol will be amended to include information about each new agent to be evaluated, as well as the handling of any design issues in the context of the platform design.

Figure 3.0-1 provides a simplified overview of the current study design. The study includes a phase II evaluation for all investigational agents. For infused agents, the study also includes a transition into a larger phase III evaluation without a pause in enrollment provided that safety data are acceptable as determined by the study’s independent Data and Safety Monitoring Board (DSMB). Two analyses of phase II efficacy data will also be undertaken: one when virologic data is available through study day 7 for approximately 55 participants on the investigational agent (and approximately 55 in the placebo control group for evaluating that agent), and one when all phase II participants for that agent have data available through study Day 28. Enrollment in phase III will continue based on these analyses (and acceptable safety data) if there is adequate evidence of efficacy based on “graduation” criteria described below. For non-infused agents, the phase III evaluation will be developed in a subsequent version or amendment of the protocol. Hence, if enrollment to phase II for a non-infused investigational agent is completed before that protocol version is available, then enrollment for that agent will pause, pending release of the new protocol version.

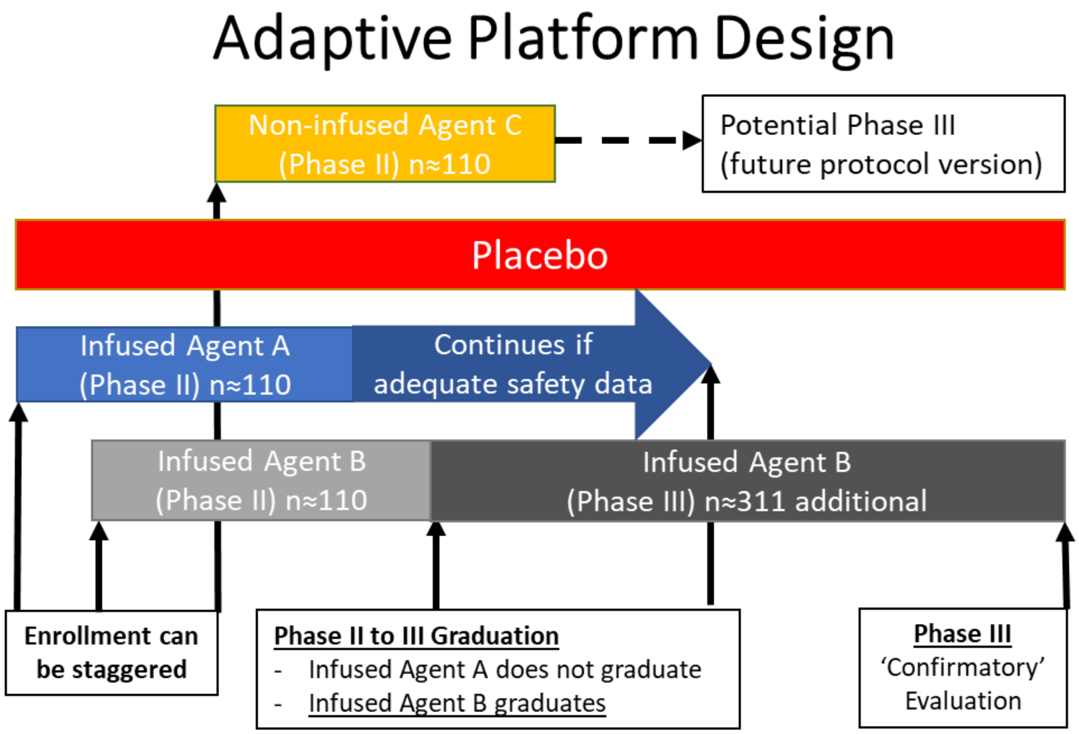

Figure 3.0-1: Adaptive platform trial that includes a phase II evaluation of both non-infused and infused investigational agents. For infused agents, the study includes the possibility of graduating to phase III evaluation. For non-infused agents, the phase III evaluation is pending and will be included in a future protocol version. Comparison of a given investigational agent is with concurrently randomized participants receiving placebo who could have been randomized to receive the agent (taking account of the fact that only participants who are at higher risk of severe COVID-19 can be randomized to an infused agent). If an infused agent graduates to phase III evaluation, the 110 participants from phase II plus the 311 additional participants enrolled in phase III (total 421 participants) will be used to address phase III objectives. For infused agents, comparison placebo recipients will be only those who are "higher risk" (i.e., all placebo recipients from infused agents or placebo recipients in the higher-risk stratum for non-infused agents); symptom duration strata will be balanced. For non-infused agents, the comparison placebo recipients will be balanced across risk and symptom duration strata.

Selection of Investigational Agents

The trial will rapidly assess various investigational agents that have shown substantial promise as anti-SARS-CoV-2 therapeutics in pre-clinical testing and for which there are suitable pharmacokinetics and safety data from phase I testing or through clinical or research testing for a different indication and agent availability. The TOC will choose which agents are evaluated by the trial and when a standard-of-care agent will replace a placebo [14]. Up to two dose levels of the same agent may be assessed. Based on TOC recommendations, an investigational agent can move directly into phase III testing without prior phase II evaluation in this trial. In this instance, for infused agents, the number enrolled in the phase III evaluation will be approximately 842 participants (421 on active and 421 on pooled placebo) versus 622 participants in phase III if a phase II evaluation had occurred in this Adapt Out COVID trial. The phase III design for non-infused agents, including sample size considerations, is forthcoming in a subsequent protocol version.

Phase II Period of Evaluation

In phase II, an investigational agent will be evaluated for safety, as well as for activity in reducing the duration of COVID-19 symptoms over 28 days, and SARS-CoV-2 RNA below lower limit of quantification in NP swabs as compared to control.

Phase II Early Termination

During the phase II evaluation, the DSMB will review interim safety results on a monthly basis (or as otherwise recommended by the DSMB). The DSMB may recommend early termination of randomization to a particular investigational agent if there are safety concerns.

3.2 Infused Agents: Overview of Study Design for Graduation from Phase II to Phase III

For infused investigational agents, the study is designed to allow both phase II and phase III evaluation of promising agents in a single trial (for non-infused agents, the phase III evaluation will be added in a future version of the protocol). Promising infused agents with limited product availability may only be evaluated in phase II, and a phase III evaluation may occur at a later time. Agents may also enter directly into the phase III evaluation, if sufficient safety and efficacy data are available from outside the trial with approval from the TOC.

For each infused agent, an interim analysis will be conducted when the 220 participants assigned to the agent or concurrent placebo in phase II evaluation have data available through to day 28 of follow-up. This interim analysis will be used to assess whether study-defined “graduation” criteria have been met so that the agent may graduate to phase III evaluation. The graduation criteria are described further below.

Figure 3.0-2 provides an overview of the graduation decision process. The DSMB will review the unblinded data and make a recommendation to NIAID (as trial sponsor) and hence to the TOC indicating whether or not graduation criteria have been met. The recommendation to continue further into the phase III evaluation will be made by the TOC in discussion with the company.

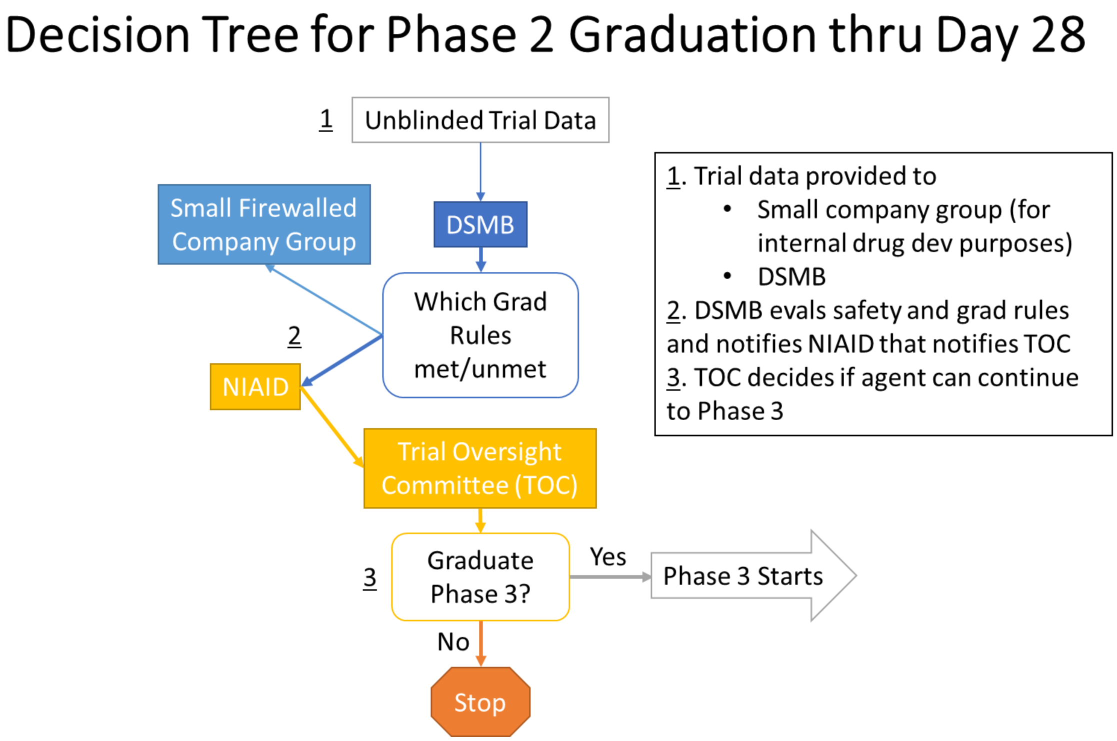

Figure 3.0-2: 1. Unblinded trial data will be provided to the Data and Safety Monitoring Board (DSMB) for interim analyses after Day 28 data have been generated to assess Phase II graduation rules. 2. Unblinded Day 28 data will also be provided to a small group of people from the company who owns the investigational agent. The small company group will not be allowed to share unblinded trial data outside of their group, per a clinical trial agreement. The rationale for sharing unblinded trial data to the small company group is to assist the company in choosing a dose of their investigational agent to move into phase III. The DSMB will provide recommendations based on graduation rules and safety to NIAID, as the trial sponsor, and then NIAID will report DSMB recommendations to the TOC. 3. In discussion with the company, the TOC, on behalf of the trial sponsor (NIAID), will then decide whether an investigational agent enters into phase III.

Phase II to Phase III Graduation Rules for Infused Agents

Graduation will be based on there being a desired level of evidence of an effect of an investigational agent versus placebo on one or more virologic and clinical outcome measures detailed below, as well as safety measures, as described below. The level of evidence required for the virology and clinical measures will be expressed in terms of Bayesian probability statements of the following form:

Probability (agent is better than placebo by at least X) is greater than 0.6 where X is defined below for each outcome measure.

The choice of 0.6 for this probability indicates that there are 3 to 2 odds of the agent being better than placebo for that parameter. As there is considerable uncertainty about the association between phase II outcomes and the phase III outcome of hospitalization or death, graduation will be considered if this probability statement is met for any one of the virology and symptom outcome measures listed below (i.e.,

it does not need to be met for all outcome measures listed).

Virology: The virology-based graduation guideline for an investigational agent to be eligible for phase III evaluation will be evidence of any one of the following:

1. Higher absolute proportion of participants testing below the lower limit of quantification (LLoQ) for SARS-CoV-2 in NP swabs by at least 20% at one or more of the scheduled in-person measurement times (e.g., 30% for placebo and 50% for investigational agent at day 7) as compared to placebo (i.e., X in the probability statement above is an absolute 20% increase for this outcome); or
2. A decrease in median SARS-CoV-2 RNA levels in NP swabs of at least 0.5 $\log_{10}$ copies/mL at one or more of the scheduled in-person measurement times through to day 7 as compared to placebo (i.e., X in the probability statement above is 0.5 $\log_{10}$ copies/mL) (measurements after day 7 are not considered as a majority of participants are expected to be below the LLoQ after day 7); or
3. A relative reduction in median area under the curve measure (AUC) of SARS-CoV-2 RNA levels in NP swab viral loads through study day 28 of at least 20%, as compared to placebo (i.e., X in the probability statement above is a relative 20% reduction).

The absolute difference of 20% in 1) and the 0.5 $\log_{10}$ copies/mL difference in 2) were surpassed in a comparison of interferon beta-1b, ribavirin, and lopinavir-ritonavir to lopinavir-ritonavir alone in a trial among hospitalized COVID-19 patients [8]. The threshold used in 3) also seems achievable based on the same trial though the AUC outcome was not formally evaluated in that trial.

Symptoms: The symptom-based graduation guideline for an investigational agent to be eligible for phase III evaluation will be a relative reduction of at least 20% in median duration of symptoms as compared to placebo (i.e., X in the probability statement above is a relative 20% reduction).

Hospitalization/Death: Although there will be very limited precision to compare an investigational agent to placebo in Phase II, graduation may also be considered based on hospitalization/death if the proportion of participants who are hospitalized or die by day 28 is lower by 33.3% (specifically, one-third) for an investigational agent versus placebo (i.e., X in the probability statement above is a relative reduction of 33.3% for this outcome).

Safety: Graduation to phase III will also depend on an acceptable safety profile, as determined by the DSMB. This decision will largely be based on differences in the frequency of Grade 3 and 4 AEs between participants receiving the investigational agent and those receiving placebo.

Other: The TOC may also consider other secondary outcomes (such as the dynamics of virologic measures and symptoms over time, or any evidence of viral rebound to suggest resistance) in the decision to graduate an investigational agent from phase II to phase III evaluation, as provided by the DSMB. In addition, based on TOC recommendations from review of existing data from outside of the study, an infused investigational agent may move directly into phase III evaluation without completing phase II evaluation through this trial.

The final decision to graduate an investigational agent to phase III will be determined when day 28 evaluations have been completed for all phase II participants. Prior to assessing graduation criteria, participants may be randomized into the phase III portion of the trial upon completion of phase II enrollment if the DSMB determines that the safety of the agent is adequate based on available phase II data. An additional interim analysis of phase II data will be undertaken when approximately 55 participants on an investigational agent (and approximately 55 in the placebo group for evaluating the agent) have viral shedding data in NP swabs through to day 7, which will also be used to determine enrollment into phase III. If graduation criteria for viral shedding at day 3 and/or day 7 are met in this interim analysis, and/or

graduation criteria are met for hospitalization/death based on all available data at the time of that interim analysis, then phase III enrollment will continue pending the day 28 graduation analysis including data from all phase II participants; otherwise, enrollment to the investigational agent will pause after phase II enrollment of 220 participants is complete (if this interim analysis occurs before phase II is fully enrolled), or as soon as possible (if phase III enrollment has already begun on the basis of safety data) pending the day 28 graduation analysis. This means that some participants may be enrolled into phase III before all evaluations have been completed for all participants in phase II, and thus, participants may be enrolled in phase III before a decision has been made by the TOC that an agent should graduate. For participants who are enrolled in phase III for an agent that does not graduate, they will be followed per the phase III SOE for the given investigational agent/placebo for safety and other evaluations (Table 6.1-2 and agent-specific appendix).

Phase III Period of Evaluation for Infused Agents

If it is decided that an infused agent graduates to phase III evaluation, then the study will continue for that agent using a continuation of the randomized design. Phase III will evaluate efficacy of the investigational agent to reduce the composite primary outcome of hospitalization or death over 28 days (i.e., from study day 0 through day 28) with additional follow-up to at least week 24 for clinical and immunologic parameters. To increase efficiency of the design, data collected during the phase II evaluation will contribute to the phase III evaluation. Throughout phase II and phase III, participants who do not start their randomized investigational agent or placebo will be replaced with new participants who are re-randomized.

Phase III Early Termination for Infused Agents

During the phase III evaluation, there will be reviews of both interim safety and efficacy results by an independent DSMB. The DSMB may recommend early termination of randomization to a particular investigational agent if there are safety concerns, if efficacy of the agent versus placebo has been established, or if it is unlikely that efficacy of the agent versus placebo would be established by continuing to planned maximal sample size. As a guideline for early termination of the comparison of an agent to placebo based on efficacy using concurrently randomized participants, an O’Brien and Fleming type stopping guideline will be used. Early termination for statistical and operational futility will also be considered.

3.3 Considerations Regarding the Use of Placebos and the Sharing of Placebo Groups for Evaluating Multiple Investigational Agents

The inclusion of a placebo arm, rather than an untreated open-label control group, is considered important for the integrity of the study to reduce the possibility of differential retention of participants randomized to an investigational agent versus to the control group, as well as to minimize subjective bias in completion of symptom diaries by participants.

Having exactly the same placebo for multiple investigational agents with different modes of administration is, however, not achievable. To speed evaluation of multiple investigational agents, the study uses a control group that includes participants who received placebos for different agents. The selection of participants in the placebo control group for evaluating a specific agent follows two key principles: (1) they must have been eligible to receive the specific agent of interest; and (2) they must have been concurrently randomized with the group of participants who received the specific agent of interest in the same phase (II or III) of evaluation. Of note, the first principle means that a participant at lower risk for severe COVID-19 cannot be part of the placebo control group for an infused agent, as only higher risk participants are eligible to received infused agents. For the second principle, the restriction to being in the same phase of evaluation is necessary because participants receiving a placebo under the phase III set of evaluations undergo a reduced set of evaluations compared with participants receiving a placebo under the phase II set of evaluations, and

therefore do not include all necessary evaluations for an agent in phase II. The randomization system is complex, but has been designed to fulfill these principles and, in doing so, also allows for a placebo control group that will have approximately the same sample size and characteristics (including by the randomization stratification factors) as the group of participants receiving a specific agent.

Figure 3.0-3 provides an illustration of how the randomization system works for the situation in which there are three agents in the same phase of evaluation including one infused agent (labeled A) for which only participants at higher risk for severe COVID-19 are eligible, and two non-infused agents (labeled B and C) for which any participant irrespective of their risk for severe COVID-19 is eligible. The figure shows how the randomization might occur for 300 participants, of whom 120 are at higher risk and 180 and are lower risk for severe COVID-19. The choice of 300 participants for this illustration is arbitrary; the ratio of higher to lower risk participants approximately reflects experience in this study as of November 2020. The system uses two randomizations within each risk group. The first randomization is to an “agent group” and is not blinded because it is not practical to blind mode of administration of an agent. The second randomization is within each agent group, and is to active agent or associated placebo and is double-blind. Of note, the ratio of the second randomization to active agent or placebo depends on the number of agents in the same phase of evaluation that a participant was eligible to receive. The choice of this ratio provides the mechanism for achieving similar sample sizes for the pooled placebo control and active agent for a given agent group.

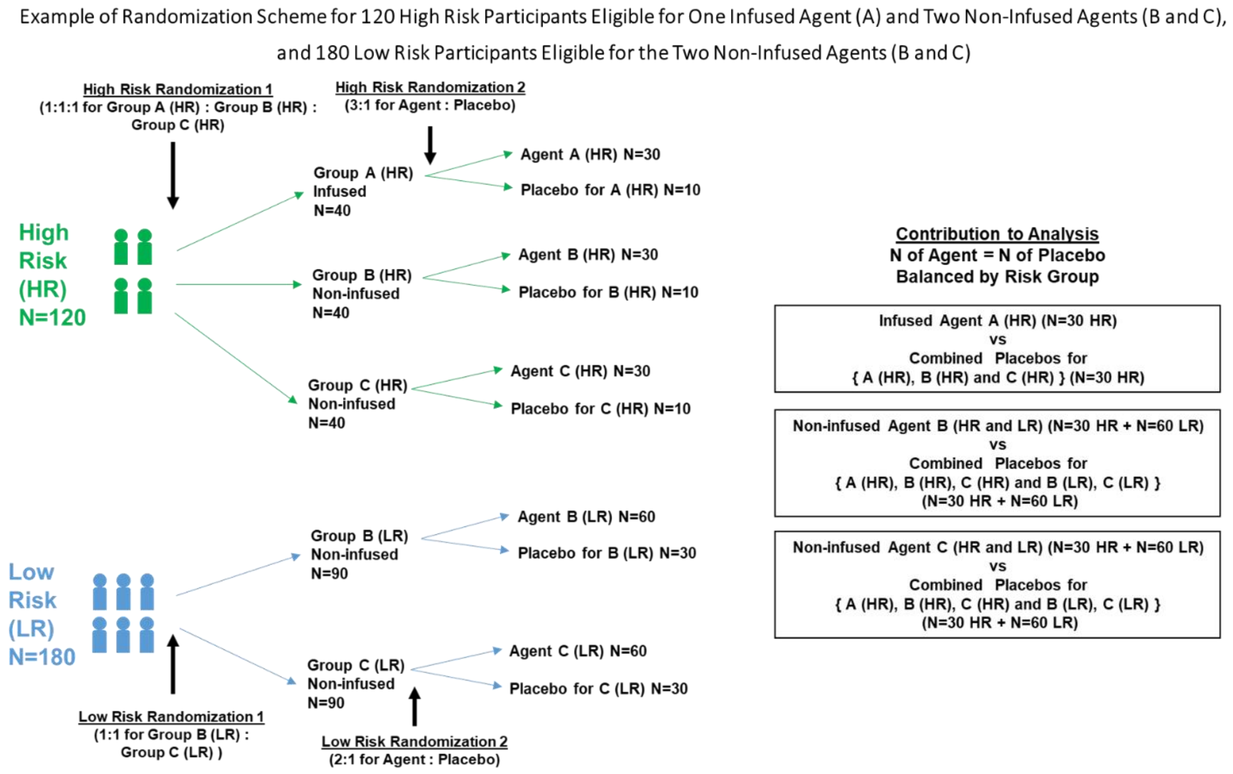

Figure 3.0-3: Illustrative example of the randomization system for agents A, B, and C in with a concurrent period of phase II evaluation. Participants at higher risk of severe COVID-19 are eligible to receive A, B, or C; whereas participants at lower risk of severe COVID-19 are eligible to receive only the non-infused agents, B and C. Participants will undergo two randomizations. Within each risk stratum, the first

randomization will be to each (agent) Group equally. The second randomization is to active agent or corresponding placebo within each Group. The ratio used in the second randomization is chosen to ensure that the number of participants receiving each active agent is approximately equal to the number assigned any of the placebos (i.e., combining the placebos into a single control group) in a given study phase. The right-hand side of the figure shows the construction of the placebo control group for evaluating each active agent, with the placebo control group and active agent group having the same sample sizes. In practice, the two group sizes might not be exactly equal dependent on random variation, the block size, and eligibility requirements.

The platform design also needs to be flexible with regard to potential differences in study population eligible for randomization to different agents, for example due to safety or polypharmacy issues. As an example, if some participants are eligible to receive Agent A but not Agent B, then the randomization is structured to allow randomization of these participants to Agent A or placebo only. In this case, these participants would not be considered as part of the placebo group for evaluating Agent B since their inclusion in this comparison could introduce bias.

The combining of placebo groups to construct the control placebo group for a given agent has the caveat that placebo effects might vary among the placebos for different investigational agents, for example, related to mode of administration. The study team considers that the risk of differential placebo effects on objective outcome measures such as the virologic outcome measures (key primary and secondary outcome measures in the phase II evaluation) is likely very low. It is also thought that the risk is very low for the phase III primary outcome measure of hospitalization/death, particularly as this outcome measure requires at least a 24-hour period of hospitalization—thus requiring a clinical decision that is unlikely to be determined by mode of administration of an investigational agent (as distinct from, for example, a participant-driven decision to go to an emergency room without a subsequent hospitalization of at least 24 hours). It is recognized that participants might possibly score symptoms of COVID (in participant symptom diaries) differentially according to mode of administration of an agent but the study team believes the risk is low. However, recognizing this possibility, a supportive analysis of the secondary symptom duration outcome in phase III will be undertaken using the investigational agent's own placebo (or a combined placebo group using placebos with the same mode of administration, e.g., by infusion). This analysis will be detailed in the Statistical Analysis Plan. Although such a supportive analysis will have reduced precision than the main analysis of symptom durations, it will be well-powered because of the large sample size needed in phase III to evaluate an investigational agent with respect to the hospitalization/death primary outcome.

Isolation Procedures

Given that SARS-CoV-2 is spread through respiratory secretions, each site must develop procedures to protect study staff and participants in other trials from infectious exposure. Each site will have a plan for appropriate protection by providing PPE, setting up isolation rooms, and providing special access points or contact with study participants, including the possibility for home or other non-clinic in-person visits. Each site will develop their own set of procedures for such participant contact. Guidance for the sites can be found in the Manual of Procedures (MOP).

4.0 SELECTION AND ENROLLMENT OF PARTICIPANTS

4.1 General Eligibility Criteria

4.1.1 Inclusion Criteria

4.1.1.1 Ability and willingness of participant (or legally authorized representative) to provide informed consent prior to initiation of any study procedures.

4.1.1.2 Individuals ≥18 years of age.

4.1.1.3 Documentation of laboratory-confirmed SARS-CoV-2 infection, as determined by a molecular (nucleic acid) or antigen test from any respiratory tract specimen (e.g., oropharyngeal, NP, or nasal swab, or saliva) collected ≤240 hours prior to study entry and conducted at any US clinic or laboratory that has a Clinical Laboratory Improvement Amendments (CLIA) certification or its equivalent or any non-US DAIDS-approved laboratory.

4.1.1.4 Participants must be expected to begin study treatment no more than 8 days from self-reported onset of COVID-19 related symptoms or measured fever, where the first day of symptoms is considered symptom day 0 and defined by the self-reported date of first reported sign/symptom from the following list:

- subjective fever or feeling feverish
- cough
- shortness of breath or difficulty breathing at rest or with activity
- sore throat
- body pain or muscle pain/aches
- fatigue
- headache
- chills
- nasal obstruction or congestion
- nasal discharge
- loss of taste or smell
- nausea or vomiting
- diarrhea
- documented temperature >38°C

4.1.1.5 One or more of the following signs/symptoms present within 24 hours prior to study entry:

- subjective fever or feeling feverish
- cough
- shortness of breath or difficulty breathing at rest or with activity
- sore throat
- body pain or muscle pain/aches
- fatigue
- headache
- chills
- nasal obstruction or congestion
- nasal discharge
- nausea or vomiting
- diarrhea
- documented temperature >38°C

4.1.1.6 Oxygenation saturation of ≥92% obtained at rest by study staff within 24 hours prior to study entry. For a potential participant who regularly receives chronic supplementary oxygen for an underlying lung condition their oxygen saturation should be measured while on their standard home oxygen supplementation level.

4.1.1.7 Agrees to not participate in another clinical trial for the treatment of COVID-19 or SARS-CoV-2 during the study period until reaching hospitalization or 28 days post-entry, whichever is earliest.

4.1.1.8 Additional inclusion criteria as appropriate for the investigational agent (see relevant appendix/appendices).

4.1.2 Exclusion Criteria

4.1.2.1 History of or current hospitalization for COVID-19.

4.1.2.2 For the current SARS-CoV-2 infection, any positive SARS-CoV-2 molecular (nucleic acid) or antigen tests from any respiratory tract specimen (e.g., oropharyngeal, NP, or nasal swab, or saliva) collected >240 hours prior to study entry.

4.1.2.3 Current need for hospitalization or immediate medical attention in the clinical opinion of the site investigator.

4.1.2.4 Use of any prohibited medication listed in section 5.4.1 within 30 days prior to study entry.

4.1.2.5 Receipt of convalescent COVID-19 plasma or other antibody-based anti-SARS-CoV-2 treatment or prophylaxis at any time prior to study entry.

4.1.2.6 Receipt of other available investigational treatments for SARS-CoV-2 at any time prior to study entry. This does not include drugs approved for other uses and taken for those uses.

4.1.2.7 Known allergy/sensitivity or any hypersensitivity to components of the investigational agent or placebo. See relevant appendix.

4.1.2.8 Any co-morbidity requiring surgery within 7 days prior to study entry, or that is considered life threatening in the opinion of the site investigator within 30 days prior to study entry.

4.1.2.9 Additional exclusion criteria as appropriate for the investigational agent (see relevant appendix/appendices).

4.2 Study Enrollment Procedures

All sites will be registered through the DAIDS Protocol Registration Office (DAIDS PRO) at the Regulatory Support Center (RSC) by PPD.

Prior to implementation of this protocol, and any subsequent full version amendments, each site must have the protocol and the protocol consent form(s) approved, as appropriate, by the institutional review board (IRB)/ethics committee (EC) and any other applicable regulatory entity (RE) responsible for oversight of the study.

Upon receiving final approval, PPD on the site’s behalf will submit all required protocol registration

documents to the DAIDS PRO at the RSC. The DAIDS PRO will review the submitted protocol registration packet to ensure that all of the required documents have been received.

Site-specific informed consent forms (ICFs) will be reviewed and approved by the DAIDS PRO, and sites and PPD will receive an Initial Registration Notification from the DAIDS PRO. A copy of the Initial Registration Notification should be retained in the site's regulatory files.

For amendments, sites will receive a notification letter from PPD with instructions to sites prior to implementation. Upon receiving final IRB/EC and any other applicable RE approvals for an amendment, sites should provide the necessary approvals to PPD.

PPD will submit amendment registration packets to the DAIDS PRO at the RSC on behalf of the sites. The DAIDS PRO will review the submitted protocol registration packet to ensure that all required documents have been received. For full version protocol amendments, sites must receive the initial registration notification for the amendment from the DAIDS PRO prior to implementing the amendment. Site-specific ICF(s) will be reviewed by the DAIDS PRO if the site ICF was not submitted as part of the prior registration.

Sites and PPD will receive an Amendment Registration Notification when the DAIDS PRO receives a complete registration packet. The first notification will be based on receipt of minimal document requirement, which allows sites to start the implementation of the amendment. A final notification will be sent to sites and PPD once the entire registration packet review has been completed. A copy of the Amendment Registration Notification should be retained in the site's regulatory files.

For additional information on the protocol registration process and specific documents required for initial and amendment registrations, refer to the current version of the DAIDS Protocol Registration Manual.

4.2.1Protocol Activation

PPD will be responsible for site activation for both ACTG and non-ACTG sites.

4.2.2Randomization

Participants who meet the enrollment criteria will be randomized to the study through the IRT (Interactive Response Technology) system.

4.3 Co-enrollment Guidelines

Co-enrollment in an observational study or the ACTG REPRIEVE study (ACTG 5332) is allowed and does not require permission from the A5401 protocol chairs, as long as ACTG network blood collection limits are not exceeded, that is, 450 mL over 8 weeks.

Co-enrollment in an interventional study following hospitalization for COVID-19 or after 28 days post-entry (Day 29 onward) for the treatment of COVID-19 or its complications is allowed.

For specific questions and approval for co-enrollment in other studies, sites should follow the directions described in the Study Management section.

5.0INVESTIGATIONAL AGENT

Study treatment is defined as any active investigational agent and an appropriate placebo identified by the

TOC for use in this study.

5.1 Regimen, Administration, and Duration

See relevant appendix/appendices for details of investigational agents.

5.2 Formulation, Storage, and Preparation

See relevant appendix/appendices for details of investigational agents.

5.3 Supply, Distribution, and Accountability

5.3.1Acquisition/Distribution

See relevant appendix/appendices for details of investigational agents.

5.3.2Accountability

See relevant appendix/appendices for details of investigational agents.

5.4 Concomitant Medications

Whenever a concomitant medication or investigational agent is initiated or a dose changed, investigators must review the concomitant medications and the relevant protocol appendix/appendices, as well as the most recent package insert, Investigator's
Brochure, or updated information from DAIDS to obtain the most current information on drug interactions, contraindications, and precautions.

Additional drug information may be found on the ACTG Precautionary and Prohibited Medications Database located at http://tprc.pharm.buffalo.edu/home/di_search/.

5.4.1 Prohibited Medications

Use of hydroxychloroquine (unless used chronically for autoimmune diseases), chloroquine (unless used for a parasitic infection), ivermectin (unless used for a parasitic infection), any antibody-based therapy for COVID-19, remdesivir, fluvoxamine (unless used chronically), and HIV protease inhibitors (unless used chronically for HIV infection) while on study, prior to hospitalization. In the event of hospitalization, these medications may be given unless otherwise specified in the agent-specific appendix/appendices.

See relevant appendix/appendices for additional prohibited medications, if applicable.

5.4.2 Precautionary Medications

See relevant appendix/appendices for precautionary medications, if applicable.

6.0 CLINICAL AND LABORATORY EVALUATIONS

See appendix/appendices for additions to the following clinical and laboratory evaluations.

6.1 Schedule of Evaluations

Table 6.1-1: Schedule of Evaluations Phase II

Phase II Evaluation	Screening	Study Entry/Day 0	Day 3	Day 7	Day 14	Day 28	Week 12	Week 24	Premature Study D/C (Before Day 28 Visit)	Premature Study D/C (After Day 28 Visit)
Visit Window			+/-1 day	+/-2 days		+4 days	-7/+14 days			
Documentation of SARS-CoV-2 Infection	X									
COVID-19 Symptom Screen	X	X								
Medical/Medication History	X	X								
Smoking Status		X								
Clinical Assessments	X	X	X	X	X	X	X	X	X	X
Collect/Update Secondary Contacts		X	X	X	X	X	X			
Vital Status Check		If Participant Cannot be Reached per section 6.3.8								
Investigational Agent Administered		Per Appendix for Investigational Agent								
Study Kit Dispensed		X								
Participant-Completed Study Diary		Every Day through Day 28								

Phase II Evaluation	Screening	Study Entry/Day 0	Day 3	Day 7	Day 14	Day 28	Week 12	Week 24	Premature Study D/C (Before Day 28 Visit)	Premature Study D/C (After Day 28 Visit)
Visit Window			+/-1 day	+/-2 days		+4 days	-7/+14 days			
Study Diary Reminder		Days 1- 28								
Staff Review of Study Diary		X	X	X	X	X			X	
Retrieval of Study Diary						X			X	
Post-Acute COVID-19 Assessment							X	X		X
Household Infection and Linkage Report		X				X	X	X	X	X
Self-Collected Anterior Nasal Swab		Every Day through Day 14				X			X	
Retrieval of Self-Collected Anterior Nasal Swabs			Follow Instructions in MOP						X	
Staff-Collected NP Swab		X	X	X	X	X			X	
Blood Plasma for SARS-CoV-2 RNA		X		X					X	
Inflammatory Markers		X		X		X		X		
Coagulation Markers		X		X		X		X		
Hematology	Per Appendix for Investigational Agent									
Chemistry	Per Appendix for Investigational Agent									
Pregnancy Testing	Per Appendix for Investigational Agent									

Phase II Evaluation	Screening	Study Entry/Day 0	Day 3	Day 7	Day 14	Day 28	Week 12	Week 24	Premature Study D/C (Before Day 28 Visit)	Premature Study D/C (After Day 28 Visit)
Visit Window			+/-1 day	+/-2 days		+4 days	-7/+14 days			
Pharmacokinetics	Per Appendix for Investigational Agent									
Stored Plasma		X		X		X		X	X	X
Stored Serum		X		X		X		X	X	X
Stored PBMCs (Selected Sites)		X		X		X		X	X	

Table 6.1-2: Schedule of Evaluations Phase III

Phase III Evaluation	Screening	Study Entry/Day 0	Day 3	Day 7	Day 14	Day 28	Week 12	Week 24	Premature Study D/C (Before Day 28)	Premature Study D/C (After Day 28)
Visit Window			+/-1 day	+/-2 days		+4 days	-7/+14 days			
Documentation of SARS-CoV-2 Infection	X									
COVID-19 Symptom Screen	X	X								
Post-Acute COVID-19 Assessment							X	X		X
Medical/Medication History	X	X								
Smoking Status		X								
Clinical Assessments	X	X	X	X	X	X	X	X	X	X
Collect/Update Secondary Contacts		X	X	X	X	X	X			
Vital Status Check		If Participant Cannot be Reached per section 6.3.8								
Investigational Agent Administered	Per Appendix for Investigational Agent									
Study Kit Dispensed		X								
Participant-Completed Study Diary		Every Day through Day 28								
Study Diary Reminder		Days 1- 28								
Staff Review of Study Diary		X	X	X	X	X			X	
Retrieval of Study Diary						X			X	

Phase III Evaluation	Screening	Study Entry/Day 0	Day 3	Day 7	Day 14	Day 28	Week 12	Week 24	Premature Study D/C (Before Day 28)	Premature Study D/C (After Day 28)
Visit Window			+/-1 day	+/-2 days		+4 days	-7/+14 days			
Household Infection and Linkage Report		X				X	X	X	X	X
Self-Collected Anterior Nasal Swab		X	X	X	X	X			X	
Retrieval of Self-Collected Anterior Nasal Swabs			Follow Instructions in MOP						X	
Blood Plasma for SARS-CoV-2 RNA		X							X	
Inflammatory Markers		X				X			X	
Coagulation Markers		X				X			X	
Hematology	Per Appendix for Investigational Agent									
Chemistry	Per Appendix for Investigational Agent									
Pregnancy Testing	Per Appendix for Investigational Agent									
Pharmacokinetics	Per Appendix for Investigational Agent									
Stored Plasma		X				X		X	X	X
Stored Serum		X				X		X	X	X

6.2 Timing of Evaluations

6.2.1 Screening Evaluations

Screening evaluations must occur prior to the participant starting any study medications, treatments, or interventions.

Screening and study entry visit evaluations may be combined unless not allowed per the relevant appendix/appendices. If feasible, screening evaluations may occur remotely.

Study entry visit evaluations must be done prior to administration of study agent.

6.2.2 Entry Evaluations

Entry evaluations must occur ≤48 hours after screening evaluations unless otherwise specified.

Participants must be expected to begin study treatment no more than 8 days from self-reported onset of COVID-19 related symptoms or measured fever as noted in section 4.1.1.4.

6.2.3 Post-Entry Evaluations

On-Treatment/Post-Treatment Evaluations
Evaluations should occur in the visit windows described in Tables 6.1-1 and 6.1-2.

Study Completion Evaluations
Participants will be evaluated at week 24 or later, depending on the agent-specific appendix.

6.2.4 Event-Driven Evaluations

See relevant appendix/appendices for details of any event-driven evaluations.

6.2.5 Discontinuation Evaluations

Evaluations for Randomized Participants Who Do Not Start Investigational Agent/Placebo
All eCRFs must be keyed for the period up to and including the entry visit. Participants who were randomized but do not start investigational agent or placebo will be prematurely discontinued from the study and will not be followed.

Premature Treatment Discontinuation Evaluations
Participants who discontinue investigational agent or placebo early should remain on study and all evaluations should be performed as outlined in Tables 6.1-1 and 6.1-2.

Premature Study Discontinuation Evaluations
Participants who discontinue study participation should have premature study discontinuation evaluations, as outlined in Tables 6.1-1 and 6.1-2 and the relevant appendix/appendices, prior to being taken off the study, unless the reason for premature study discontinuation was that they did not start investigational agent or placebo.

6.3 Instructions for Evaluations

Sites must follow PPD source document guidelines.

All evaluations below are for both Phase II and III unless otherwise noted.

All stated evaluations are to be recorded on the eCRF unless otherwise specified. Refer to section 7.0 for information on reporting of adverse events.

In the event of hospitalization, targeted physical examination, study diary entry and review, and specimen collection do not need to be completed during hospitalization but should be restarted after discharge. Other evaluations should be performed as feasible, including ascertainment of interventions, including medications received, and outcomes of interest/study endpoints.

Location of Study Visits
Sites should, in discussion with participants, determine the most appropriate place to conduct study visits, whether in-person or remote.

In person visits will take place at the clinic, at the participant's home, or at another non-clinic location if the site is able to accomplish all of the scheduled study visit evaluations.

Remote visits can take place over the phone or via telemedicine systems approved for use at the site.

6.3.1 Documentation of SARS-CoV-2 Infection

Section 4.1.1.3 specifies assay requirements for SARS-CoV-2 infection documentation. SARS-CoV-2 infection documentation is recorded on the eCRF. If a viral load level is available, it should be recorded as well.

See the MOP for further guidance.

6.3.2 COVID-19 Symptoms

COVID-19 Symptom Screen
Participants will be asked about their first symptoms related to COVID-19 and their current symptoms.

The time from symptom onset at anticipated study entry (≤5 days versus >5 days) should be recorded.

Post-Acute COVID-19 Assessment
Participants will be asked about potential COVID-19-related symptoms and diagnoses experienced after Day 28 using a standardized questionnaire (see MOP for additional information).

6.3.3 Medical History

At Screening and updated at Study Entry, a complete medical history for the preceding 120 days should be recorded. Additionally, the following diagnoses should be recorded regardless of when the diagnosis was made, except where noted:

- autoimmune disease
- pulmonary embolus

- deep venous thrombosis
- HIV infection
- cancer (exclusive of basal/squamous cell skin cancer)
- acute viral respiratory infection (influenza, parainfluenza, respiratory syncytial virus, rhinovirus) within the previous 14 days (if known by participant)
- chronic lung disease
- asthma requiring daily inhaled medication
- obesity (body mass index [BMI] >35; may be based on self-report of height and weight)
- hypertension
- cardiovascular disease
- diabetes
- chronic kidney disease
- history of cirrhosis
- exogenous or endogenous immunosuppression

The participant's risk category for COVID-19 progression ("higher" vs. "lower" risk) should be recorded. If participant meets the criteria for "higher" risk, all high risk criteria that are met should be recorded.

Any allergies to any medications and their formulations must also be documented.

See appendix/appendices for additional elements of the medical history that should be recorded.

6.3.4 Medication History

A medication history must be present, including start and stop dates. The table below lists the medications that must be included in the history at screening and updated at entry.

Table 6.3.4-1: Medication History

Medication/Category	Timeframe
All prescription drugs	Last 7 days
Corticosteroids, anabolic steroids	Last 30 days
Prescription drugs for high blood pressure	Last 3 months
Prescription drugs for diabetes and pre-diabetes	Last 3 months
Prescription drugs for lung disease	Last 3 months
Prescription drugs for heart disease	Last 3 months
Prescription drugs for autoimmune disease	Last 3 months
Cancer chemotherapy	Last 3 months
Antiretroviral therapy	Last 3 months
Immune-based therapy	Last 3 months
Blinded investigational agent	Last 12 months
CoV-related vaccines or treatments	Complete history
Hydroxychloroquine	Complete history
Antibiotics	Last 3 months
Anti-parasitics	Last 3 months
Alternative therapies	Last 3 months
Dietary supplements (including zinc and vitamins C and D)	Last 3 months

6.3.5 Smoking Status

A Smoking Status questionnaire will be completed as part of medical history and recorded on the eCRF.

6.3.6 Clinical Assessments

<u>Physical Examination</u>
Weight is measured only at screening.

At entry, perform physical exam, including cardiac exam, pulmonary exam, and vital signs (temperature, pulse, blood pressure, and resting peripheral oxygen saturation).

After entry, perform a targeted physical examination at the following visits: Phase II Day 3, Day 7, Day 14, Day 28, Week 24, and Premature Study D/C (before or after Day 28) and Phase III Day 28, Week 24, and Premature Study D/C (before or after Day 28). A targeted physical examination will also be performed at other visits not listed here if required for specific agents (see appendix/appendices). A targeted physical examination includes vital signs (temperature, pulse, blood pressure, and resting peripheral oxygen saturation) and examinations driven by any previously identified or new adverse event/targeted condition that the participant has experienced.

Supplemental oxygen use will be recorded at each visit at which vital signs are recorded.

At study entry, if peripheral oxygen saturation is <92% on usual supplemental oxygen requirements, the participant should be referred for emergency department evaluation and should not initiate investigational product.

Post-entry, peripheral oxygenation saturation measures <96% should be reviewed by an investigator and referral for medical attention made at the discretion of the investigator.

See appendix/appendices for any additional elements needed for the targeted exam.

Post entry, see section 8.3 for collection requirements for pregnancy.

<u>Concomitant Medications</u>
Post entry, the following new and discontinued concomitant medications must be recorded:

- high blood pressure medications
- steroids or other immunosuppressive or immunomodulatory medication
- non-steroidal anti-inflammatory drugs (NSAIDS)
- chemotherapy
- antibiotics, antifungals, antiparasitics, and antivirals (including antiretrovirals)
- anticoagulants
- antiplatelets
- any approved or investigational agent felt to have potential COVID-19 activity (including hydroxychloroquine, chloroquine, ivermectin, HIV protease inhibitors, and SARS-CoV-2 vaccines)
- inhalers
- medications for symptoms of COVID-19, including aspirin, ibuprofen, acetaminophen, zinc, dietary supplements, herbal remedies, decongestants, cough suppressants, and antihistamines.

Assessment for Adverse Events
Beginning at entry, participants will be assessed at every visit (remote or in-person) for any new signs or symptoms and the relationship to study treatment.

Investigational Agent Modifications
Post entry, record any initial dose of treatment, modification to treatment, treatment interruption, and permanent discontinuation of treatment, and the reason for the modification, interruption, or discontinuation.

6.3.7 Collect/Update Secondary Contacts

Sites will capture contact information for at least two individuals that the site can contact if the participant cannot be reached (e.g., spouse, friend, neighbor). Sites will also request health care provider contact information and hospital(s) that the participant is likely to go to if they get sick.

Contact information for secondary contacts or health care provider will not be recorded on any eCRF.

At study entry only, sites will record the participant's home address in site records (it will not be reported on an eCRF).

6.3.8 Vital Status Check

If a participant cannot be reached after two attempts 24 hours apart, then their listed secondary contact person(s) or health care provider will be contacted for a check of the participant's vital status and study endpoints. In addition, for participants who prematurely discontinue for reasons other than withdrawal of consent or non-initiation of investigational product, or at any time the site becomes aware of a potential hospitalization or death after the participant discontinued study, site personnel should attempt to obtain information on the vital status of the participant and study endpoints as outlined in the MOPs.

Vital status contacts and other reported information should be recorded on the eCRFs.

6.3.9 Investigational Agent Administered

See relevant appendix/appendices for dispensing/administration details.

6.3.10 Study Kit Dispensed

The kit will include:
- copy of informed consent
- information about the study
- instructions on study procedures
- pocket/wallet card with site staff contact information
- instructions on what to do if participants have worsening symptoms/become hospitalized
- swabs for self-collected anterior nasal swabs with storage and transport materials
- study diary (see below)

6.3.11 Study Diary

Participant-Completed Study Diary
Participants will be asked to keep a log of symptoms, medications they are taking for COVID-19 symptoms, and major events such as urgent visit to an emergency room or clinic and hospitalization in their study diary. This log will be completed on paper or electronically, if appropriate electronic systems are available.

At study entry, participants will complete the study diary with site staff prior to initiating investigational agent/placebo. Participants will be asked to complete subsequent entries per the SOE. The diary should be completed at approximately the same time every day.

If the day 28 visit occurs on study day 28, then the day 28 study diary may be completed with the site staff during the day 28 visit, otherwise it should be completed by the participant on study day 28.

Study Diary Reminder and Staff Review of Study Diary
Participant will be contacted every day on days 1-28 and reminded to complete their study diary. This reminder may be by telephone, text message, email, or other method for which the participant provides permission. A direct response from the participant is not required.

The study diary will be reviewed by study staff in person or remotely with each participant according to the schedule in Tables 6.1-1 and 6.1-2. If an appropriate electronic system is available, the participant's diary entries will automatically be captured in the eCRF. If such a system is not available, the study staff will record the participant's answers on the study diary eCRF. If the participant uses a paper diary and it is feasible, prior to or during the remote study visits, sites will ask the participant to send images of each of their study diary entries to be reviewed at the next study contact. See MOPS for requirements for timely eCRF entry of diary data.

Participants who report worsening symptoms from any cause during the trial may be referred to their health care provider or closest emergency room. Such instances will be recorded at the time of the notification, and during follow-up to assess study endpoints, i.e., hospitalization or death.

Retrieval of Study Diary
If the participant uses a paper diary, the study diary should be collected following the current Diary Completion Guidelines on the A5401 PSWP. See MOPS for additional instructions on retrieval of Study Diary.

6.3.12 Household Infection and Linkage Report

At Study Entry/Day 0, participants will be asked if anyone who resides in their household, defined as sharing indoor living space or housekeeping space (i.e., kitchen, dining area, or bathroom) has been diagnosed with SARS-CoV-2 infection or are also enrolled in the study, and the response recorded on the eCRF. If a household member is enrolled in the study, the participant ID for the first household member enrolled into the study will be recorded.

Post entry, participants will be asked if any new household members have been diagnosed with SARS-CoV-2 infection, and the response recorded on the eCRF.

6.3.13 Virologic Studies

Anterior nasal and NP swabs and plasma will be collected for quantitative SARS-CoV-2 RNA, performed in near real-time.

Influenza and other respiratory viral testing may be performed on stored NP swabs.

Additional information can be found in the MOP and the LPC.

Self-Collected Anterior Nasal Swabs (Phase II and III)
Participants will self-collect anterior nasal swabs. Participants will be instructed by study staff and will obtain the day 0 swab while observed by study staff. This swab should be collected prior to the first dose of investigational agent.

After Day 0, in phase II, on days when an in-person visit occurs, the swab will be self-collected at the clinic on that day. On days without an in-person visit, the swabs will be self-collected by the participant on their own, when completing the study diary. Participants will record the time they collect their swab each day. Participants will turn in their self-collected (remote) swabs at their next in-person visit.

After Day 0, in phase III, nasal swabs will be self-collected by the participant on their own. Remote-collected nasal swabs will be stored at home as per the MOP.

Retrieval of Self-Collected Nasal Swabs (Phase II and III)
Site staff will retrieve the nasal swabs collected by the participants at home as per the MOP and LPC. The swabs will be processed, stored, and shipped to the central laboratory as per the LPC.
Staff-Collected NP Swab (Phase II only)
NP swabs will be collected during in-person visits after the self-collected nasal swab. At study entry, the sample should be collected prior to the first dose of investigational agent.

Blood Plasma for SARS-CoV-2 RNA (Phase II and III)
Blood plasma will be collected during in-person visits. At study entry, the sample should be collected prior to the first dose of investigational agent.

6.3.14 Laboratory Evaluations

The following laboratory evaluations are for all investigational agents. If additional measures are needed, these are detailed in the relevant investigational agent appendix.

Refer to the LPC for details of collection, processing, and shipping.
At screening, entry, and post-entry, all laboratory values must be recorded unless otherwise specified in the relevant appendix/appendices.

At study entry, blood samples should be collected prior to initiation of the investigational agent.

Blood can be collected outside of a clinic setting (e.g., home).

Inflammatory Markers
Lactate dehydrogenase, C-reactive protein, ferritin, and D-dimer will be performed.

Coagulation Markers

PT, PTT, INR, and fibrinogen will be performed.

Hematology
See relevant appendix/appendices for testing requirements.

Chemistry
See relevant appendix/appendices for testing requirements.

Pregnancy Testing
See relevant appendix/appendices for testing requirements.

6.3.15 Pharmacokinetics

Pharmacokinetic sampling will be performed per the relevant appendix/appendices.

6.3.16 Stored Samples

Collected plasma, sera, or PBMC will be used to assess SARS-CoV-2 virologic and immune responses. All Entry/Day 0 samples should be collected prior to the first dose of investigational agent/placebo. Additional samples will be collected for agent-specific evaluations per the relevant appendix/appendices.

Stored Plasma
Blood plasma will be collected and stored for future testing, including:

- immunologic studies including markers linked to systemic inflammation (IL-6, TNF-a), inflammasome activation (IL-1beta, IL-18), interferon pathways (IP-10, type I interferon), neutrophil activation (MPO), monocyte activation (sCD14), as well as markers associated with coagulation or endothelial cell dysfunction (VWF, P-selectin, tissue factor)
- SARS-CoV-2 seroconversion and antibody titers (among seroconverters)
- full viral genome sequencing will be performed from select samples that are detectable for SARS-CoV-2 RNA to assess for signs of viral evolution and resistance to the investigational agent or immune responses. If sequence analysis suggests viral escape from the investigational agent (e.g. mutations in putative binding regions or epitopes), then phenotypic analyses may be pursued.

Stored Serum
Blood sera will be collected and stored for future testing, including:

- total and neutralizing antibody assays

Stored Peripheral Blood Mononuclear Cells (PBMCs)
PBMCs will be collected only at select sites. PBMC processing must be done in an IQA-approved lab. PBMCs will be stored for future testing, which may include the following:

- cellular immune responses between treatment and control samples, including assessment of T-cell responses to SARS-Cov-2 protein (phase II: days 0, 7, 28, and week 24)
- cellular activation/exhaustion phenotypes among innate or adaptive immune cells (phase II: days 0, 7, 28, and week 24)
- host genetics

7.0ADVERSE EVENTS AND STUDY MONITORING

See relevant appendix/appendices for any modifications to recording of AEs and study monitoring.

See the MOPS for further instructions on AE reporting.

7.1 Definitions of Adverse Events

Adverse Event
An adverse event (AE) is any unfavorable and unintended sign (including an abnormal laboratory finding), symptom, or diagnosis that occurs in a study participant during the conduct of the study REGARDLESS of the attribution (i.e., relationship of event to medical treatment/investigational agent/device or procedure/intervention). This includes
any occurrence that is new in onset or aggravated in severity or frequency from the baseline condition.

The scale used in the Study Diary for participant symptoms does NOT equate to the AE grading as found in the Division of AIDS Table for Grading the Severity of Adult and Pediatric Adverse Events (DAIDS AE Grading Table), corrected Version 2.1, July 2017.

Sites should grade participant symptoms as they normally would according to the DAIDS AE Grading Table.

Serious Adverse Events (SAEs)
An SAE is defined as any untoward medical occurrence that results in any of the following outcomes:

- results in death
- is life-threatening
- requires inpatient hospitalization or prolongation of existing hospitalization
- results in persistent or significant disability/incapacity
- is a congenital anomaly/birth defect.
- is an important medical event that may not be immediately life threatening or result in death or hospitalization but may jeopardize the participant or may require intervention to prevent one of the other outcomes listed in the definition above).

Adverse Events of Special Interest
An adverse event of special interest (AESI) (serious or nonserious) is defined as an AE or SAE of scientific and medical concern specific to the investigational agent, for which ongoing monitoring and rapid communication by the investigator to the sponsor could be appropriate.

See appendix/appendices for AESIs related to specific investigational agents.

Suspected Unexpected Adverse Events
A Suspected Unexpected Serious Adverse Reaction (SUSAR) is defined as a serious adverse reaction, the nature or severity of which is not consistent with the applicable product information (e.g., Investigator's Brochure for an unapproved investigational product).

7.2 Eliciting and Documenting Adverse Events

Adverse events will be assessed beginning at Entry/Day 0 and through study completion or discontinuation.

If the investigator learns of any SAE, including a death, at any time after a participant has been discharged from the study, and he/she considers the event to be reasonably related to the investigational agent or study participation, the investigator must promptly notify the sponsor.

Serious AEs that occur after study completion or discontinuation need not be reported unless the investigator considers them related to the investigational product.

At every study visit, participants will be asked a standard nonleading question to elicit any medically related changes in their well-being. They will also be asked if they have been hospitalized, had any accidents, used any new medications, or changed concomitant medication regimens (both prescription and OTC medications).

In addition to participant observations, AEs identified from any study data (e.g., laboratory values, physical examination findings, or identified from review of other documents [e.g., participant diaries]) that are relevant to participant safety will be documented on the AE page in the eCRF.

7.2.1 Assessment of Severity

The severity, or intensity, of an AE refers to the extent to which an AE affects the participant's daily activities.

All AEs that are reported must have their severity graded. To grade AEs, sites must refer to the Division of AIDS Table for Grading the Severity of Adult and Pediatric Adverse Events (DAIDS AE Grading Table), corrected Version 2.1, July 2017, which can be found on the DAIDS RSC website at: https://rsc.niaid.nih.gov/clinical-research-sites/daids-adverse-event-grading-tables.

7.2.2 Assessment of Causality

If there is any doubt as to whether a clinical observation is an AE, the event should be reported.

The relationship or association of the investigational agent/placebo in causing or contributing to the AE will be characterized using the following classification and criteria:

- Unrelated: There is no association between the investigational agent/placebo and the reported event.
- Related: A causal relationship exists between administration of the investigational agent/placebo and the AE, and other conditions (concurrent illness, progression/expression of disease state, or concurrent medication reaction) do not appear to explain the event.

7.3 Recording Adverse Events

Post entry, the following must be recorded on the eCRFs within 72 hours:

- Grade ≥2 AEs
- AEs that led to a change in study treatment/intervention regardless of grade

Post entry, the following must be recorded on the eCRFs within 24 hours:

- AEs meeting SAE definition
- AESIs

Information to be collected includes the following:
study product group (investigational agent/placebo)
route of administration
dose
event term
time of onset

investigator-specified assessment of severity and relationship to the investigational product
time of resolution of the event
seriousness
any required treatment or evaluations
outcome

Adverse events resulting from concurrent illnesses, reactions to concurrent illnesses, reactions to concurrent medications, or progression of disease states must also be reported. All AEs will be followed to adequate resolution. The MedDRA will be used to code all AEs.

Any medical condition that is present at the time that the participant is screened but does not deteriorate should not be reported as an AE. However, if it deteriorates at any time during the study, it should be recorded as an AE with a descriptive modifier (e.g., “Exacerbation of,” “Worsening of,” “Deterioration of”) the event.

7.3.1Reporting Serious Adverse Events

Any AE that meets SAE criteria must be reported to PPD, Inc., immediately (i.e., within 24 hours of the time that the site personnel first learn about the event) by indicating on the Adverse Event eCRF within the Electronic Data Capture (EDC) system that seriousness criteria is met and providing initial relatedness/causality.

In the event the EDC electronic submission is not possible, a completed SAE/AESI report form along with written description of the serious adverse experience must be sent to PPD PVG by facsimile within 1 business day after awareness of the event (see regional Fax numbers below). Please note, the event must be entered into EDC once access has been corrected.

PPD Safety Reporting Fax Number
NA: +1 888 529 3580
LA: +55 11 4504 4802
EMEA/APAC: +44 (0)1223 374102

The following contact information is to be used for inquiries to determine if an event is reportable as an SAE:

PPD Safety Hotline Phone Number
NA (RTP): +1 888 483 7729
LA: +55 11 4504 4801
EMEA/APAC: +44 (0)1223 374240

The sponsor has a legal responsibility to notify the US FDA and other regulatory agencies about the safety of an investigational product under clinical investigation. The sponsor will comply with country-specific regulatory requirements relating to safety reporting to the regulatory authority, institutional review board/independent ethics committee (IRB/IEC), and investigators.

An investigator who receives an investigator safety report or memorandum describing an SAE or other specific safety information from the sponsor will review and then file it as appropriate and will notify the IRB/IEC and local regulatory agencies, if appropriate according to local requirements.

7.3.2 Reporting Adverse Events of Special Interest

Any AE that meets AESI criteria (section 7.1) must be reported immediately (i.e., within 24 hours of the time that the site personnel first learn about the event) by indicating on the Adverse Event eCRF that AESI criteria are met. If electronic submission is not possible it can be submitted in the same manner as the back-up manual SAE/AESI reporting process (section 7.3.1).

Contact the PPD Safety Hotline Phone Number with any questions on reportability.

7.3.3 Reporting Suspected Unexpected Serious Adverse Reactions

The sponsor will promptly evaluate all SUSARs and nonserious AEs of special interest (defined in section 7.1) against cumulative product experience to identify and expeditiously communicate possible new safety findings to investigators, IRBs/IECs, and applicable health authorities based on applicable legislation.

To determine reporting requirements for single AE cases, the sponsor will assess the expectedness of these events using the investigational agent Investigator’s Brochure.

The sponsor will compare the severity of each SUSAR and the cumulative event frequency reported for the study with the severity and frequency reported in the applicable reference document.

Reporting requirements will also be based on the investigator's assessment of causality and seriousness, with allowance for upgrading by the sponsor as needed.

7.4 Follow-up of Participants Reporting Adverse Events

All AEs must be reported in detail on the appropriate page in the eCRF and followed to satisfactory resolution, until the investigator deems the event to be chronic or not clinically significant, the event is considered to be stable, or the participant is lost to follow-up.

7.5 Study Monitoring

The protocol team will monitor the conduct and safety of the study via regular summaries of accrual, study discontinuation, data completeness, and adverse events.

The DAIDS Clinical Representative will review and assess select AE reports for potential impact on the study participant safety and protocol conduct as per DAIDS policies, guidance documents, and SOPs as applicable.

The DSMB will conduct interim reviews for safety. Enrollment will pause and the DSMB will review any death that occurs on study that is deemed related to study product as determined by the site investigator. A pause in enrollment for that study product group (investigational agent/placebo) will also occur and the

DSMB will review if two participants experience a Grade 4 AE that is deemed related to study product as determined by the site investigator.

See section 10.0 for statistical and other considerations related to interim monitoring.

Detailed plans for study monitoring are outlined in a Safety Management Plan.
See relevant appendix/appendices for additional monitoring procedures.

8.0CLINICAL MANAGEMENT ISSUES

The following guidance pertains to all investigational agents; however, additional guidance for particular agents are included in the appendix relevant for each investigational agent.

8.1 Toxicity

Criteria for participant management, dose adjustments and discontinuation, or changes in treatment will be described only for toxicities attributable to the investigational agents, when applicable, and are included in the appendix/appendices.

The grading system for drug toxicities is located in the Division of AIDS Table for Grading the Severity of Adult and Pediatric Adverse Events (DAIDS AE Grading Table), corrected Version 2.1, July 2017, which can be found on the DAIDS RSC website at https://rsc.niaid.nih.gov/clinical-research-sites/daids-adverse-event-grading-tables.

NOTE: The protocol team must be notified within 72 hours regarding toxicities that result in a change in study regimen (follow the directions described in the Study Management section).

For all agents evaluated in this trial, if a participant develops a Grade 4 AE that is related to the study product as determined by the site investigator, no further doses of the study treatment should be administered.

It is possible that some participants will experience transient or prolonged AEs during the study. As some of the visits will be conducted remotely, AEs will often be assessed remotely and unplanned study visits scheduled if deemed necessary by the site investigator. For any concerning AEs that are felt to require clinical intervention, participants should be instructed to contact their health care provider or seek urgent or emergent care, or 911 should be called, as appropriate.

Treatment may be discontinued without contacting the protocol team in advance, but the protocol team should be notified within 24 hours of parenteral and 72 hours of oral treatment discontinuation (follow the directions described in the Study Management section). This includes an interruption in administration for single-dosed agents.

8.2 Management of Side Effects

See relevant appendix/appendices for additional details on the management of side effects.

8.2.1Overdose

An overdose is any dose of study treatment given to a participant or taken by a participant that exceeds the dose described in the protocol.

Any overdose must be reported to the PPD Drug Safety Center within 24 hours (follow the directions described in the Study Management section). The overdose itself is not to be reported as an AE. However, any AEs associated with the overdose are to be reported on relevant AE/SAE sections in the eCRF.

In the event of an overdose, the site investigator should:

1. Contact the protocol team immediately (follow the directions described in the Study Management section).
2. Closely monitor the participant for any AE/SAE and laboratory abnormalities.
3. Obtain a plasma sample for PK analysis within 3 days from the date of the last dose of investigational agent/placebo if requested by the medical monitor.
4. Document the quantity of the excess dose as well as the duration of the overdose in the eCRF.

Decisions regarding dose interruptions or modifications will be made by the site investigator in consultation with the medical monitor based on the clinical evaluation of the participant.

8.3 Pregnancy

The use of investigational agents in pregnancy will vary depending upon agent. The ability to continue or need to discontinue investigational agent in event of pregnancy is outlined in the relevant appendix/appendices.

8.4 Breastfeeding

The use of investigational agent in breastfeeding participants who meet inclusion criteria for the study will vary depending upon agent and is outlined in the relevant appendix/appendices.

9.0CRITERIA FOR DISCONTINUATION

Participants may discontinue from the investigational product or withdraw from the study at any time and for any reason without prejudice to their future medical care by the investigator or at the study site. Every effort should be made to keep participants in the study. The reasons for participants discontinuing the investigational product and/or withdrawing from the study will be recorded on an eCRF.

9.1 Permanent and Premature Treatment Discontinuation

- Drug-related toxicity mandating discontinuation (see appendix/appendices).
- Participant experiencing an SAE that is considered related to investigational agent.
- Requirement for prohibited concomitant medications (see section 5.4 and relevant appendix/appendices).
- Request by participant to terminate treatment.
  NOTE: The reason for treatment discontinuation should be documented (e.g., concern for AE, lack of efficacy, or other reason).
- Clinical reasons believed life threatening by site clinical staff, even if not addressed in the Toxicity section of the protocol.
- Any additional indications are outlined in the relevant appendix/appendices.

9.2 Premature Study Discontinuation

- Failure to initiate investigational agent.

- Request by the participant to withdraw consent.
- Request of the health care provider if they think the study is no longer in the best interest of the participant.
- At the discretion of the IRB/EC, FDA, NIAID, ACTG, Office for Human Research Protections (OHRP), other government agencies as part of their duties, investigator, or industry supporter.
- Any additional indications are outlined in the relevant appendix/appendices.

In the event that a participant prematurely discontinues from the study, unless they have withdrawn consent or never initiated investigational agent/placebo, sites will attempt to obtain information regarding vital status (including date last seen alive, hospitalization, date of death, and primary cause of death) from other sources (e.g., family members, other designated secondary contacts, or clinic records). See the MOP for further guidance.

10.0 STATISTICAL CONSIDERATIONS

10.1General Design Issues

There are two major benefits of the proposed trial design. First, the platform trial aspect of the design allows for efficient evaluation of multiple investigational agents compared to concurrently randomized participants (who were eligible for a particular agent) in a combined placebo control group. Second, for infused agents, the transition from phase II evaluation to phase III evaluation for graduating investigational agents provides for more rapid evaluation of an investigational agent than having separate phase II and phase III trials. In both phase II and phase III evaluation, the intent is to focus on comparisons between each investigational agent and the placebo control, and not on comparisons among investigational agents. Control of Type I error rate will be undertaken separately for each investigational agent rather than across all investigational agents (so not the experiment-wise or family-wise error rate).

There is very little data available for ambulatory persons with COVID-19 and so this section provides information about the general approach that will be pursued with initial agents evaluated in this study. However, it is expected that this study will rapidly provide key information about clinical and virologic outcomes and their inter-relationships, and so the study design may be modified as this information accumulates. In particular, based on this accumulating information, a Bayesian framework will be developed to improve the process for deciding which infused agents graduate from phase II to phase III evaluation. This information will also be important for re-evaluation of sample size requirements for both phases. The Bayesian analytical framework will be described in a Graduation Rules Statistical Analysis Plan

It is expected the study will need to undergo a significant protocol amendment if an agent is shown to be effective in reducing hospitalization/death in the phase III evaluation or a new standard of care for the outpatient population is established outside of this study. Therefore, this possibility is not considered in this section.

10.2 Outcome Measures

Primary and secondary outcome measures listed below will be addressed in the study’s primary Statistical Analysis Plan, which will define the content of the Primary Analysis Report of outcomes through day 28 of follow-up and a Secondary Analysis Report of further outcomes through to week 24. These reports will form the basis for the main study manuscript(s) and results reporting to ClinicalTrials.gov.

10.2.1 Phase II: Primary Outcome Measures

10.2.1.1 Clinical (Symptom Duration): Duration of targeted COVID-19 associated symptoms from start of investigational agent (day 0) based on self-assessment. Duration defined as the first of two consecutive days when any symptoms scored as moderate or severe at study entry (pre-treatment) are scored as mild or absent, AND any symptoms scored as mild or absent at study entry (pre-treatment) are scored as absent The targeted symptoms are feeling feverish, cough, shortness of breath or difficulty breathing, sore throat, body pain or muscle pain or aches, fatigue (low energy), headache, chills, nasal obstruction or congestion (stuffy nose), nasal discharge (runny nose), nausea, vomiting, and diarrhea. Each symptom is scored daily by the participant as absent (score 0), mild (1) moderate (2) and severe (3).

10.2.1.2 Virologic: At each of days 3, 7, 14, and 28, quantification (<LLoQ versus ≥LLoQ) of SARS-CoV-2 RNA from site-collected NP swabs.

10.2.1.3 Safety: New Grade 3 or higher AE through 28 days.

10.2.2 Phase III: Primary Outcome Measures

10.2.2.1 Efficacy: Death from any cause or hospitalization during the 28-day period from and including the day of the first dose of investigational agent or placebo. Hospitalization is defined as ≥24 hours of acute care, in a hospital or similar acute care facility, including Emergency Rooms or temporary facilities instituted to address medical needs of those with severe COVID-19 during the COVID-19 pandemic.

10.2.2.2 Safety: New Grade 3 or higher AE through 28 days.

10.2.3 Secondary Outcome Measures

The clinical primary outcome measure in phase II (symptom duration) will also be assessed in phase III as a secondary outcome measure.

The primary outcome measure in phase III (death from any cause or hospitalization through 28 days) will also be assessed in phase II as a secondary outcome measure, including for non-infused agents

The following secondary outcome measures will also be assessed:

10.2.3.1 Phases II and III: Quantification (<LLoQ versus ≥LLoQ) and level of SARS-CoV-2 RNA from participant-collected nasal swabs through day 28.

10.2.3.2 Phases II and III: COVID-19 severity ranking based on symptom severity scores over time during the 28-day period from and including the day of the first dose of investigational agent or placebo, hospitalization, and death. For participants who are alive at 28 days and not previously hospitalized, the severity ranking will be based on their area under the curve AUC of the daily total symptom score associated with COVID-19 over time (through 28 days counting day 0 as the first day) where the total symptom score on a given day is defined as the sum of scores for the targeted symptoms in the participant’s study diary (each individual symptom is scored from

0 to 3). Participants who are hospitalized or who die during follow-up through 28 days will be ranked as worse than those alive and never hospitalized as follows (in worsening rank order): alive and not hospitalized at 28 days; hospitalized but alive at 28 days; and died at or before 28 days.

10.2.3.3 Phases II and III: Progression through day 28 of one or more COVID-19-associated symptoms to a worse status than recorded in the study diary at study entry, prior to start of investigational agent or placebo.

10.2.3.4 Phases II and III: Time to self-reported return to usual (pre-COVID-19) health as recorded in a participant's study diary on two consecutive days through day 28.

10.2.3.5 Phases II and III: Death from any cause or hospitalization during the 24-week period from and including the day of the first dose of investigational agent.

10.2.3.6 Phase II only: Oxygen saturation (i.e., pulse oximeter measures) as a quantitative measure and categorized as <96 versus ≥96% through day 28.

10.2.3.7 Phase II only: Area under the curve and above the assay lower limit of quantification of quantitative SARS-CoV-2 RNA over time from site-collected NP swabs at days 0, 3, 7, 14, and 28 and from self-collected nasal swabs daily at days 0-14 and at day 28.

10.2.3.8 Phase II only: Level (quantitative) of SARS-CoV-2 RNA from site-collected NP swabs at days 3, 7, 14, and 28.

10.2.3.9 Phase II only: New Grade 2 or higher AE through 28 days, and through week 24.

10.2.3.10 Phase III only: New Grade 3 or higher AE through week 24.

10.2.3.11 Phase II only: Pharmacokinetic measures will be defined in the agent-specific appendices.

10.2.4 Other Outcome Measures

10.2.4.1 Phases II and III: Worst clinical status assessed using ordinal scale among participants who become hospitalized. Ordinal scale defined as:

- death
- hospitalized, on invasive mechanical ventilation or ECMO;
- hospitalized, on non-invasive ventilation or high flow oxygen devices;
- hospitalized, requiring supplemental oxygen;
- hospitalized, not requiring supplemental oxygen (COVID-19 related or otherwise)

10.2.4.2 Phases II and III: Duration of hospital stay among participants who become hospitalized.

10.2.4.3 Phases II and III: ICU admission (yes versus no) among participants who become hospitalized.

10.2.4.4 Phases II and III: Duration of ICU admission among participants who are admitted to the ICU.

10.2.4.5 Phases II and III: New SARS-CoV-2 positivity among household contacts through to 28 days and through to 24 weeks from start of investigational agent or placebo.

10.2.4.6 Phases II: Quantification (<LLoQ versus ≥LLoQ) and level of SARS-CoV-2 RNA in blood.

10.2.4.7 Phase II only: Area under the curve and above the assay lower limit of quantification of quantitative SARS-CoV-2 RNA over time in blood.

10.2.4.8 Phases II and III: Hematology, chemistry, coagulation, and inflammatory markers through 28 days from start of investigational agent.

10.2.4.9 Phases II and III: Plasma markers of inflammation and antibody responses to SARS-CoV-2 infections, measured in blood in all phase II participants and in a subset of phase III participants per relevant appendix.

10.2.4.10 Phase II and III: Viral resistance (to be defined at the time of laboratory analysis).

10.2.4.11 Phase II only: Immune cell phenotypes and T and B cell responses to SARS-CoV-2 measured in PBMCs (to be defined at the time of laboratory analysis).

10.3 Randomization and Stratification

At any time that enrollment is ongoing, participants will be randomized in two steps with the ultimate intent of having approximately equal numbers of concurrently randomized participants on a given investigational agent and on the placebo control group for that agent (i.e., combining participants who were eligible to receive the agent but who were randomized to any of the available placebos). The requirement that a participant in the placebo control group had to have been eligible to receive the given investigational agent also means that, for infused agents, all participants in the placebo control group will be in the higher risk stratum for progression to severe COVID-19. Participants may be randomized to agents that are in phase II evaluation and to infused agents that are in the phase III evaluation.

To allow for the possibility that each agent may have a matching placebo for blinding, the randomization will be undertaken in two steps (see example in Figure 3.0-3). First, participants at a site will be randomized in approximately equal numbers to groups corresponding to the investigational agents that they are eligible to receive which are under study at that site. For example, when enrollment is ongoing for Agents A, B, and C at a given site, participants will be randomized to Groups A, B, and C if they are eligible to receive any of Agents A, B, and C. Participants who are only eligible to receive two of the three agents (e.g., Agents A and B) would only be randomized to the two respective groups (e.g., Groups A and B). Participants who are only eligible for one agent (e.g., Agent A) would be assigned to the respective group (e.g., Group A).

Immediately following the first randomization, participants will be randomized within their assigned group to receive the interventional agent or the matching placebo for that agent. For example, in Group A, participants would be randomized to receive Agent A or the placebo for Agent A. In this second randomization, the ratio of assignment to interventional agent or placebo will be r:1 where r is the number of agents in the same phase of evaluation that a given participant is eligible to receive. The dependence of

the ratio on the phase of evaluation of the agent is necessary because of phase III evaluation involves a lesser set of evaluations than phase II evaluation and hence participants randomized to a Group in phase III evaluation cannot contribute placebo recipients to the evaluation of an agent in phase II evaluation.

As an example, consider the situation in which randomization is ongoing to three agents A, B, and C with agents A and B in phase III evaluation and agent C in phase II evaluation, and consider participants who are eligible to receive any of the agents (A, B, or C). In the first randomization, a 1:1:1 ratio would be used to assign individuals to Agent Groups A, B and C. In the second randomization, participants in Group A will be randomized in the ratio 2:1 to active Agent A and Placebo for A (as two agents are in phase III evaluation). Participants in Group B will also be randomized in the ratio 2:1 to active Agent B and Placebo for B. However participants in Group C will be randomized in a 1:1 ratio to active Agent C or Placebo for C (as only one agent is in phase II evaluation). Participants assigned to Placebo for A or to Placebo for B will contribute to the placebo control group for evaluating both Agent A and Agent B.

This two-step randomization process will achieve approximately equal numbers being assigned to an investigational agent and its concurrent placebo control group (comprised of all concurrently enrolled placebo arms combined, restricted to participants who were eligible to receive that agent).

For non-infused agents, both randomization steps will be stratified (using blocked randomization) by time from symptom onset (≤ versus >5 days) and “higher” versus “lower” risk of progression to severe COVID-19, as defined in the Schema, Stratification. For infused agents, both randomization steps will only be stratified by time from symptom onset, as only “higher” risk participants are eligible for infused agents. There will therefore be four strata for non-infused agents as both “lower” and “higher” risk participants are eligible, and two strata for infused agents, eligibility for those agents is restricted to “higher” risk participants.

10.4 Sample Size

10.4.1 Phase II

The sample size for phase II is justified by standard (frequentist) power calculations in which the true difference between an interventional agent and placebo is assumed to be the targeted difference in the Bayesian probability statement for the graduation rules. As data become available concerning the distribution of outcomes in the study population, the sample size and power considerations may also be evaluated to address power to graduate for given true differences between randomized groups based on the Bayesian probability statements.

The phase II evaluation of an investigational agent involves the comparison of two primary outcomes (quantifiable SARS-CoV-2 RNA at days 3, 7, 14, and 28; and symptom duration) among participants randomized to that agent versus participants concurrently randomized to the placebo. This evaluation will involve approximately 110 participants randomized to the investigational agent and approximately 110 participants concurrently randomized to the control group for that agent (combined across one or more concurrently randomized placebo arms). The choice of sample size has been chosen to give high power to identify an active agent based on the primary virologic outcome so we describe that first. The phase II study is not specifically designed to have a high level of power for the symptom duration outcome, but we illustrate the anticipated power to detect a range of reductions in median symptom duration. As this is the phase II component of the study and hence there will be further evaluation of an agent that graduates to phase III, no adjustment is made for the multiplicity of outcomes being assessed for a given investigational agent (or across investigational agents).

Virologic Outcome
The percentage of participants with quantifiable SARS-CoV-2 RNA in NP swabs will be compared between an investigational agent and placebo control at each of days 3, 7 14, and 28. It is uncertain what might be the percentage <LLoQ at each of these times in the population being studied, and this percentage is likely to depend on the time since onset of symptoms at which participants are enrolled. However, a 20% absolute increase in percentage of participants with SARS-CoV-2 RNA <LLoQ is thought to be relevant. For example, in a clinical trial comparing the combination of interferon beta-1b, ribavirin, lopinavir/ritonavir (n=86) to lopinavir/ritonavir alone (n=41) in hospitalized COVID-19 patients in China, there was both a difference in clinical outcomes and more than a 20% reduction in undetectable virus at about 7 days (with the caveat that this does not establish that a difference in virologic outcome is a surrogate for a difference in clinical outcome) [8]. The median time to undetectable virus was 7 versus 14 days in this trial (based on daily NP swabs obtainable in the hospitalized setting), indicating that 50% of participants were undetectable at 7 and 14 days in the two groups.

With a phase II sample size of 110 participants assigned to an investigational agent and a similar number concurrently assigned to placebo, we assume that about 100 participants in each group will have NP swabs available at a scheduled measurement time. Table 10.4.1-1 shows the power to detect a 20% absolute increase in percentage of participants with unquantifiable virus for a range of percentages with unquantifiable virus in the placebo arm. The power was calculated for the comparison of two proportions using a normal approximation to the binomial distribution and unpooled variance, with two-sided Type I error rate of 5%. A power of over 82% is achieved regardless of the percentage of participants with unquantifiable virus in the control group. A sample size of 100 per group with NP swabs would also provide reasonable precision in estimating the absolute difference between groups in percentage with unquantifiable virus: for example, the width of a two-sided 95% confidence interval would be no more than ±13.6% around the observed difference, and the width of a two-sided 90% confidence interval would be no more than ±11.4%.

Table 10.4.1-1: Power to Detect a 20% Absolute Increase in % with SARS-CoV-2 RNA <LLoQ for Various Percentages Unquantifiable in Control Group (calculated in PASS15 software)

Control Group: Number with NP Swabs	Investigational Group: Number with NP Swabs	Percentage Unquantifiable in Investigational Arm	Percentage Unquantifiable in Placebo Arm	Power (%)
100	100	30	10	95.5
100	100	40	20	88.5
100	100	50	30	83.9
100	100	60	40	82.3
100	100	70	50	83.9
100	100	80	60	88.5
100	100	90	70	95.5

The duration of symptoms from the start of investigational agent through 28 days of follow-up will be compared between an investigational agent and placebo control.

To evaluate power and precision for this comparison, an estimate of the variability in durations is needed. We use data from the placebo arm of a US study (n=60), in which the median duration of COVID-19 symptoms (defined as time to first day with symptoms absent) was 8 days and the inter-quartile range (IQR) was 4 to 15 days [7]. For the purposes of calculating

sample size, we assume that the relative variability of durations among participants will be the same for this study's symptom duration outcome measure as in this recent data (recognizing that this study is using a different definition for symptom duration, which does not require all symptoms to be absent but conversely requires two consecutive days of sufficient symptom improvement from day 0 scores). To proceed with an assessment of power, we make the simplifying assumption that the $\log_{10}$-transformed symptom duration will be approximately normally distributed and use this normality assumption to infer a standard deviation based on the above IQR, specifically that the standard deviation equals $[\log_{10}(15) - \log_{10}(4)]/1.35 = 0.425$.

Division by 1.35 in this expression arises because the IQR for a normal distribution has width 1.35 times its standard deviation. For simplicity, we also ignore the fact that symptom durations will be measured in integer days rather than as continuous measurements, and assume that the symptom durations will be observed for all participants by day 28 (i.e., no censoring of symptom durations at 28 days).

Assuming that 100 of the 110 participants in each of the investigational agent and placebo control groups will provide study diary data, and continuing to assume a normal distribution for $\log_{10}$ durations with standard deviation of 0.425, then the phase II component of the study will have about 81% power to show a one-third (33%) relative reduction in median duration of symptoms from the start of investigational agent (e.g., 12 days to 8 days). This calculation is based on using a Wilcoxon rank sum test to compare groups using a two-sided significance level of 0.05. The power to detect smaller relative reductions will be lower: For example, it would be only 52% to detect a one-quarter (25%) relative reduction in median duration symptoms (e.g., 12 days to 9 days).

10.4.2 Phase III – Infused Agents

For infused agents, the phase III aspect of the study is designed to evaluate the efficacy of an investigational agent to reduce the proportion of participants hospitalized or dying by 28 days after starting investigational agent in outpatient adults diagnosed with COVID-19 compared to those receiving placebo. The primary analysis will focus on comparing the ratio of proportions because of the uncertainty in knowing what the hospitalization/death proportion will be.

For each infused agent that graduates to phase III, a total of approximately 421 participants will be randomized to receive that agent and approximately 421 participants will be concurrently randomized as the placebo control. This sample size includes the enrollment that occurred during the phase II evaluation. With 842 participants, the study has 90% power to detect a relative reduction of 50% in the proportion of participants hospitalized/dying between the study groups (investigational agent versus placebo), using a two-sided Type I error rate of 5%, using the following assumptions:

- Proportion hospitalized/dying in the placebo arm is 15%. This proportion is based on that observed in preliminary data in a similar higher risk outpatient population in the BLAZE-1 trial [15].
- Targeted 50% reduction is plausible based on the observed effect seen in the BLAZE-1 trial for both a single mAb and for a dual combination mAb [15]. Three interim analyses and one final analysis, equally spaced, with stopping guideline for efficacy of an agent versus placebo determined using the Lan-DeMets spending function approach with an O'Brien and Fleming boundary.
- Non-binding stopping guideline for futility using a moderately aggressive Type II error

spending function, specifically a Gamma (-2) spending function [15], implemented using the Lan-DeMets spending function approach. Further details about these stopping guidelines are in section 10.5.

- Allowance for 5% of participants to be lost-to-follow-up prior to being hospitalized or dying.

10.5 Data and Safety Monitoring

10.5.1 Phase II Period

Monitoring of safety during the time an investigational agent is in phase II evaluation is described in section 7.5. This includes the possibility that an independent NIAID-appointed DSMB may be asked to undertake an unblinded review of adverse events.

For non-infused agents, there will be interim analyses of safety data for review by the DSMB approximately each month (or on a schedule recommended by the DSMB) with the first review approximately six weeks after enrollment to an agent starts. Details regarding DSMB review of phase II results for non-infused agents, when all participants have completed day 28 of follow-up, will be described in a future version of the protocol that describes the phase III evaluation for these agents.

For infused agents, as described in section 3.0, there will also be interim analyses of safety data for review by the DSMB approximately each month (or on a schedule recommended by the DSMB) with the first review approximately six weeks after enrollment to an agent starts. If there are no safety concerns, then the DSMB may recommend continued enrollment of participants into phase III once phase II enrollment is complete with monthly (or as otherwise recommended by the DSMB) safety reviews pending interim analyses of phase II efficacy data.

For infused agents, the first interim analysis of phase II efficacy data will be undertaken when approximately 55 participants on an investigational agent (and approximately 55 in the placebo group for evaluating the agent) have viral shedding data in NP swabs through to day 7. If graduation criteria for viral shedding at day 3 and/or day 7 are met in this interim analysis, and/or graduation criteria are met for hospitalization/death based on all available data at the time of that interim analysis, then phase III enrollment will continue pending the day 28 graduation analysis including data from all phase II participants; otherwise enrollment to the investigational agent will pause after phase II enrollment of 220 participants is complete (if this interim analysis occurs before phase II is fully enrolled), or as soon as possible (if phase III enrollment has already begun on the basis of safety data) pending the day 28 graduation analysis.

For infused agents, the DSMB will also review results from complete phase II follow-up through day 28. If these results indicate that the graduation criteria have been met and there are no safety, resistance, or other concerns, then the DSMB may recommend continuation of the study for the full phase III period of evaluation. It is not generally intended to stop the phase II period of evaluation early for futility.

Only infused agents may enter into phase III; subsequent protocol version will address phase III design for non-infused agents.

10.5.2 Phase III Period – Infused Agents

A NIAID-appointed DSMB will undertake reviews of interim data from the study to help ensure the safety of participants in the study, and to recommend changes to the study including

termination or modification for safety reasons or if there is persuasive evidence of efficacy or lack of efficacy of an investigational agent versus placebo in preventing hospitalizations and deaths. It is not intended, however, to terminate evaluation of an agent early for efficacy based on symptom outcome measures. The DSMB may also recommend termination or modification of the study if it appears futile on statistical or operational
grounds to continue the study as designed. The operation of the DSMB is governed by the NIAID DSMB Charter.

At each interim review of an investigational agent, the DSMB will review summaries of data by randomized treatment arm for the primary outcome of hospitalization/death, the secondary outcome of death, losses to follow-up, and adverse events (including early discontinuation of investigational agent). By-stratum summaries will also be reviewed.

Stopping Guideline for Efficacy and Timing of Interim Efficacy Analyses
Unless otherwise recommended by the DSMB, it is intended that the DSMB review three interim analyses of safety and efficacy data for an investigational agent versus placebo at completion of day 28 follow-up for phase II participants, and after about 50% and 75% of the expected maximal efficacy (hospitalization/death) information in the trial is obtained. Note that the first interim analysis is approximately at 25% of maximal information. As a stopping guideline for greater efficacy of an investigational agent compared with placebo, the O'Brien and Fleming boundary will be used. The stopping guideline will be implemented using the Lan-DeMets spending function approach to allow for the possibility of changes in the timing of interim analyses and/or additional (or fewer) interim analyses if recommended by the DSMB.

With regard to the timing of interim analyses, the expected maximal efficacy information is approximately proportional to the expected number of hospitalizations/deaths under the assumed design parameters, i.e., assuming a proportion hospitalized/dying of 15% in the placebo control group and a relative reduction of 50% giving a proportion hospitalized/dying for the investigational agent of 7.5%, and a sample size of 421 in each group. This gives a total number of participants hospitalized/dying across the two groups combined of 95. Unless otherwise recommended by the DSMB, interim analyses will be undertaken at the following times:

1. The first interim analysis for Phase III will be when 220 participants from the two groups combined have been followed for the primary outcome assessed at day 28 (this will likely then be the same hospitalization/death information as used in the phase II graduation analysis), or when approximately 24 participants in the two groups combined have been hospitalized or have died;
2. The earlier of when approximately 421 participants from the two groups combined have been followed for the primary outcome assessed at day 28, or when approximately 48 participants in the two groups combined have been hospitalized or have died; and
3. The earlier of when approximately 632 participants from the two groups combined have been followed for the primary outcome assessed at day 28, or when approximately 72 participants in the two groups combined have been hospitalized or have died.

Formal details of the expected maximal information and calculation of information time will be provided in the Statistical Analysis Plan.

For infused agents, because phase III enrollment may be allowed to proceed pending phase II efficacy results, it is recognized that if enrollment is fast then the analyses of phase II virology and symptom efficacy data may not be completed until after one or more of the phase III interim analyses have been undertaken. If this occurs, it is intended that the phase III stopping guidelines for efficacy and futility take precedence over enrollment pause/no pause and graduation criteria

based on these analyses of phase II virology and symptom data. For example, if phase III criteria for futility are met but phase II virology efficacy data suggest that enrollment continue without pause, then the phase III criteria for futility take precedence and the DSMB may recommend termination of enrollment into the study.

In considering possible modifications to the study or termination of the study for efficacy, the DSMB may consider interim results for the secondary outcome of death. For example, the DSMB might make recommendations based on a high level of evidence for a difference between randomized groups in the proportion dying. In these contexts, a “high level of evidence” might be based on application of the O’Brien and Fleming stopping guideline to the death outcome. In these circumstances, consideration should also be given to the increased risk of a Type I error.

There is the possibility that differences between the treatment groups may be observed early in follow-up. However, the overall goal of the study is to prevent hospitalization and deaths regardless of the timing, and therefore the focus of the treatment group comparisons will be at day 28.

Stopping Enrollment to an Investigational Agent Because of Lack of Effect

If enrollment to the study is fast, there may be limited opportunity to stop enrollment to a specific investigational agent before the target of 421 participants randomized to that agent is complete (because it will take time to achieve follow-up of participants and additional time to analyze and review results). However, if the rate of enrollment allows for potential discontinuation of randomization to a specific investigational agent, then the following provides non-binding guidance on how this might be approached:

- an agent may be discontinued for statistical futility based on evidence of lack of effect or very limited effect compared with placebo. For the purposes of evaluating this, a moderately aggressive Type II error spending function will be used, specifically the Gamma (-2) spending function implemented using the Lan-DeMets spending function approach [16].

Figure 10.5.2-1 illustrates the stopping guidelines for both efficacy and futility assuming four equally spaced analyses (noting that the first interim analysis is only approximately at 26% of maximal information). The left panel shows the stopping guidelines in terms of critical values for a z-test statistic comparing an agent to placebo for the four analyses. The right panel shows the stopping guidelines in terms of observed differences in proportions for the scenario when the observed proportion in the placebo control arm is 0.15 (i.e., 15%). In both panels, greater negative values favor greater effects of an investigational agent versus placebo, and values in the blue area suggest stopping for efficacy whereas values in the pink area suggest stopping for futility. As an example, focusing on the right-hand panel, if the observed proportion for placebo was 0.15 (i.e., 15%) at the first interim analysis, an absolute difference in proportions of 0.025 or larger (i.e. favoring placebo by 2.5%) at the first interim analysis would suggest stopping for futility. At the second interim analysis, an absolute difference of -0.011 (i.e., -1.1%) or smaller (i.e. negative but closer to zero than -1.1%, or positive hence favoring placebo) would suggest stopping for futility.

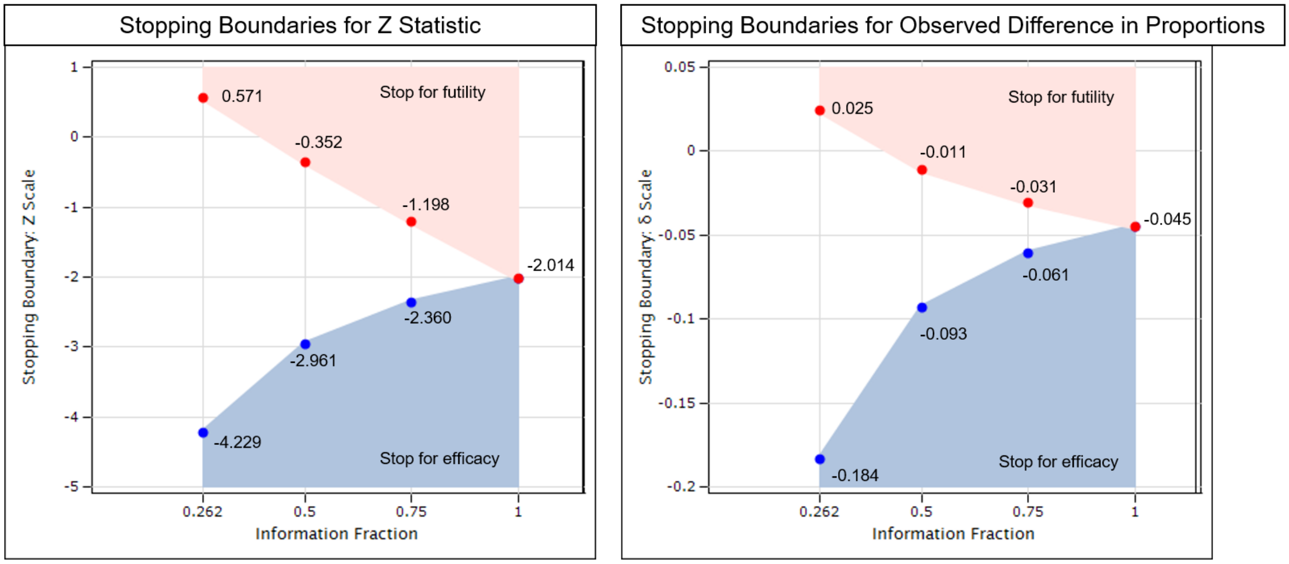

Figure 10.5.2-1: Stopping Boundaries for Efficacy and Futility

Modifying or Stopping the Study for Operational Futility
The DSMB will also monitor operational futility. With respect to operational futility, the DSMB may recommend modification or termination of the study if the proportion hospitalized/dying in the control group is much lower than expected in designing the trial. In addition, the DSMB will monitor the loss to follow-up (LTFU) rate. As a benchmark, an overall LTFU rate of more than 10% would be cause for concern.

10.6 Analyses

A Statistical Analysis Plan (SAP) will be developed that describes, in detail, the analyses to address the study's primary and secondary objectives in both phase II and phase III. The following provides an outline of the methods for the main comparisons between randomized groups, particularly for the primary outcome measures in each of phase II and phase III.

All analyses involving randomized comparisons will include all randomized participants who started an investigational agent or the concurrent placebo, according to a modified intention-to-treat approach. This should not introduce bias into the randomized comparison because of the use of a placebo. However, if evaluation of an investigational agent involves combining different placebos (i.e., because the study is partially blinded, with different placebos for different investigational agents), then consideration of the sensitivity of results to the possibility of different outcomes according to type of placebo taken will be considered; details will be provided in the SAP.

A general principle in all analyses is that outcomes among participants randomized to receive a specific investigational agent will be compared to outcomes among participants who were eligible to have been randomized (in the two-step randomization process) to the investigational agent but who were randomized instead to receive any of the placebos available at the time. This ensures that the comparison is restricted to concurrently randomized participants eligible to have taken the investigational agent of interest.

10.6.1 Primary Outcome Measures for Phase II

For evaluating the graduation criteria described in section 3.0, a Bayesian framework will be used to calculate the posterior probability that the difference in outcome for an investigational agent versus placebo exceeds the desired target. Initially non-informative prior distributions for relevant parameters will be used (details will be provided in a Graduation Rules Statistical Analysis Plan). The choice of prior distributions may be updated as data accrue in the study; this will be described in an amendment to the protocol or in the appendix specific to an agent.

Virologic Outcome: Unquantifiable SARS-CoV-2 RNA in NP Swabs

Descriptive statistics will be used to describe the proportion of participants with RNA <LLoQ at each scheduled measurement time. Because of uncertainty about whether hospitalization might be driven by immunologic factors rather than virologic factors, the main analysis will not impute virologic outcome if results are not available because of hospitalization (though the sensitivity of this issue will be explored by considering an imputation of having quantifiable virus during hospitalization). For (frequentist) inference in presenting results, a repeated measures analysis will be undertaken across the scheduled measurement times using a binary regression model fitted using the generalized estimating equation approach with an independence working correlation structure, and two-sided 5% Type I error rate applied to a Wald-type test of the treatment by time interaction term (time included with indicator variables for each evaluation time).

Clinical Outcome: Symptom Duration

Symptom durations will be compared between study groups using a two-sided Wilcoxon test with a 5% Type I error rate taking account of censoring, with descriptive summaries of the distribution of symptoms durations among participants.

Safety and Tolerability: Grade 3 or Higher AE

Safety and tolerability will be evaluated by estimating the proportion of participants with new Grade 3 or higher AE(s) by study day 28, and will be compared between groups using binary regression.

10.6.2 Primary Outcome Measures for Phase III

Hospitalization/Death

The cumulative proportion of participants hospitalized or dying during the first 28 days of follow-up will be estimated for each randomized group using Kaplan-Meier methods to take account of losses to follow-up. The difference between randomized groups in the estimated log cumulative proportion will be calculated and the variance for this difference will be obtained using Greenwood’s formula. Two-sided 95% confidence intervals (adjusted for multiple interim analyses) and associated p-value for the test of no difference between groups will then be obtained.

Participants who prematurely discontinue the study, who are not able to be contacted by the site to ascertain outcomes after discontinuation, will have follow up censored at the date of last known status.

The above analysis assumes that losses to follow-up are non-informative. As a sensitivity analysis of this assumption, causal inference methods, specifically inverse probability of censoring, may be used.

Safety and Tolerability: Grade 3 or Higher AE
Safety and tolerability will be evaluated by estimating the proportion of participants with new Grade 3 or higher AE(s) by study day 28, and will be compared between groups using binary regression.

10.6.3 Secondary Outcomes

The cumulative proportion of participants dying during the first 28 days of follow-up, and through to 24 weeks, and the cumulative proportion hospitalized/dying through to 24 weeks will be analyzed in a similar manner to the phase III primary outcome.
Analysis of the proportion of participants with new Grade 2 or higher AE(s) by day 28 in phase II, and new Grade 3 or higher AE(s) by week 24 in phase III, and the proportion with progression of symptoms, will be undertaken using the same approach as for the primary safety analysis.

The duration of fever, and duration of time to self-reported return to usual health will be analyzed using similar methods as for the analysis of symptom durations.

The AUC virologic outcome, COVID-19 severity ranking, will be compared between arms using a Wilcoxon test, with descriptive summaries of the distribution of these outcome measures among participants.

Levels of SARS-CoV2 RNA on days 3, 7, 14, and 28 will be compared between arms using non-parametric Wilcoxon rank-sum tests and descriptive statistics, separately at each scheduled measurement time (considering RNA results below assay limit as the lowest rank). A repeated measures analysis will also be undertaken using non-parametric methods.

In phase III, the large sample size will enable exploration of differences in symptom duration across strata defined by age, co-morbidities, and time from symptom onset to start of investigational agent using statistical methods for personalized/stratified medicine.

Descriptive summaries of clinical outcomes among those hospitalized will be provided by arm, recognizing that this would not be a randomized comparison, if restricted to participants who were hospitalized.

10.7 Unblinding

Unblinding requests will follow PPD procedures.

In general, participants who become hospitalized at any time during the study period of 24 weeks can have their individual study treatment unblinded if essential for their future treatment management or if necessary for enrollment into a COVID-19 treatment clinical trial. This determination should be made by the Investigator of Record at the trial site and documented on the eCRF.

If treatment assignment is unblinded, this information should only be shared with the physicians responsible for the management of the participant on a need-to-know basis. Treatment assignment should not be shared with others. This includes not sharing treatment assignment with the study team.

11.0 PHARMACOLOGY PLAN

The phase II pharmacology objective is to determine the pharmacokinetics of the investigational agent. For phases II and III, the pharmacology objective is to explore relationships between dose and concentration of

investigational agent with virology, symptoms, and oxygenation. Samples for quantification of concentrations of the investigational agent will be obtained using a collection schedule appropriate for that agent and phase of evaluation, taking into consideration known pharmacokinetic characteristics (e.g., elimination half-life). Pharmacokinetic data analysis will use conventional and accepted approaches such as non-compartmental analysis, compartmental analysis, and population approaches. Usual parameters of interest are area under the concentration-time curve (AUC), total or apparent body clearance (CL), elimination half-life ($T_{1/2}$), and maximum and minimum concentrations (C_{max}, C_{min}). Exploration of relationships between dose and concentration of investigational agent with virology, symptoms, and oxygenation will be approached using conventional and accepted methods for pharmacokinetic/pharmacodynamic (PK/PD) data analyses. Such methods might include the E_{max} or sigmoid E_{max} model or structurally linked PK/PD models to explore exposure-response relationships. Exposure-response relationships will be performed in conjunction with the protocol statisticians.

See relevant appendix/appendices for details of the agent-specific pharmacology plan.

12.0 DATA COLLECTION AND MONITORING

12.1 Data Quality Assurance

This study will be conducted according to the ICH E6(R2) risk and quality processes described in the applicable procedural documents. The quality management approach to be implemented in this study will be documented and will comply with the current ICH guidance on quality and risk management. The sponsor assumes accountability for actions delegated to other individuals (e.g., Contract Research Organizations).

12.2 Records to Be Kept

Electronic case report form (eCRF) screens will be made available to sites for data entry. Participants must not be identified by name on any data submitted to the DMC. Participants will be identified by the subject number provided by the Clinical Data Management System (CDMS) upon enrollment.

12.3 Role of Data Management

As part of the responsibilities assumed by participating in the study, the investigator agrees to maintain adequate case histories for the participants treated as part of the research under this protocol. The investigator agrees to maintain accurate eCRFs and source documentation as part of the case histories.

All eCRF information is to be filled in. If an item is not available or is not applicable, this fact should be indicated. Blank spaces should not be present unless otherwise directed.

Investigative site personnel will enter participant data into CDMS. The analysis data sets will be a combination of these data and data from other sources (e.g., laboratory data).

Clinical data management will be performed in accordance with applicable DAIDS and PPD standards and data cleaning procedures to ensure the integrity of the data, for example, removing errors and inconsistencies in the data. Adverse event terms will be coded using MedDRA, an internal validated medical dictionary, and concomitant medications will be coded using WHODRUG.

12.4 Clinical Site Monitoring and Record Availability

12.4.1 Site monitors under contract to the NIAID will visit participating clinical research sites to review the individual participant records, including consent forms, eCRFs, supporting data, laboratory specimen records, and medical records (physicians' progress notes, nurses' notes, individuals' hospital charts), to ensure protection of study participants, compliance with the protocol, and accuracy and completeness of records. The monitors also will inspect sites' regulatory files to ensure that regulatory requirements are being followed and sites' pharmacies to review product storage and management.

Monitoring visits may be conducted on-site or remotely. Remote visits may include remote source document verification using methods specified for this purpose by NIAID. Remote monitoring visits may be performed in place of, or in addition to onsite visits to ensure the safety of study participants and data integrity [17]. The site will make available study documents for site monitors to review utilizing a secure platform that is HIPAA and 21 CFR Part 11 compliant. Potential platform options include: Veeva SiteVault, site-controlled SharePoint or cloud-based portal, direct access to Electronic Medical Record (EMR), and Medidata Rave Imaging Solution. Other secure platforms that are 21 CFR Part 11 compliant may be utilized, as allowed by the DAIDS Office of Clinical Site Oversight (OCSO).

12.4.2 The site investigator will make study documents (e.g., consent forms, drug distribution forms, eCRFs) and pertinent hospital or clinic records readily available for inspection by the local IRB/IEC, the site monitors, the FDA, the NIAID, the ACTG, the OHRP, the industry supporter(s) or designee (as appropriate), other local, US, and international regulatory authorities/entities for confirmation of the study data.

13.0 PARTICIPANTS

13.1 Institutional Review Board (IRB) Review and Informed Consent

Federal regulations and the ICH guidelines require that approval be obtained from an IRB/IEC before human subjects participate in research studies. Before study onset, the protocol, informed consent, advertisements to be used for the recruitment of study participants, and any other written information regarding this study to be provided to the participant or the participant's legal guardian must be approved by the IRB/IEC. Documentation of all IRB/IEC approvals and of the IRB/IEC compliance with ICH harmonized tripartite guideline E6(R2). GCP will be maintained by the site and will be available for review by the sponsor or its designee.

All IRB/IEC approvals should be signed by the IRB/IEC chair or designee and must identify the IRB/IEC name and address, the clinical protocol by title or protocol number or both, and the date approval or a favorable opinion was granted.

The investigator is responsible for providing written summaries of the progress and status of the study at intervals not exceeding 1 year or otherwise specified by the IRB/IEC. The investigator must promptly supply the sponsor or its designee, the IRB/IEC, and, where applicable, the institution, with written reports on any changes significantly affecting the conduct of the study or increasing the risk to participants.

13.2 Ethical Conduct of Study

The study will be performed in accordance with the ethical principles that have their origin in the Declaration of Helsinki, ICH GCP, and all applicable regulations.

13.3 Participant Information and Consent

Informed consent in compliance with US Title 21 CFR Part 50 and US Title 45 CFR Part 46 shall be obtained from each participant before entering the study or performing any unusual or nonroutine procedure that involves risk to the participant. An informed consent template may be provided by the sponsor to investigative sites. If any institution-specific modifications to study-related procedures are proposed or made by the site, the consent should be reviewed by the sponsor or its designee or both before IRB/IEC submission. Once reviewed, the consent will be submitted by the investigator to his or her IRB/IEC for review and approval before the start of the study. If the consent for the phase and investigational agent a participant is enrolled in is revised during the course of the study, participants will be reconsented according to requirements of their IRB.

Before recruitment and enrollment, each prospective participant or his or her legal guardian will be given a full explanation of the study, be allowed to read the approved ICF, and have any questions answered. Once the investigator is assured that the participant/legal guardian understands the implications of participating in the study, the participant/legal guardian will be asked to give consent to participate in the study. A witness may be used for the informed consent process if remote consent is performed and it is not possible to obtain a copy of the signed consent form from the participant (or legal guardian or person with power of attorney for participants who cannot consent for themselves).

13.4 Participant Confidentiality

All laboratory specimens, evaluation forms, reports, and other records that leave the site will be identified by coded number only to maintain participant confidentiality. All records will be kept locked. All computer entry and networking programs will be done with coded numbers only. Clinical information will not be released without written permission of the participant, except as necessary for monitoring by the ACTG, IRB/EC, FDA, NIAID, OHRP, other local, US, and international regulatory authorities/entities as part of their duties, or the industry supporter(s) or designee.

13.5 Study Discontinuation

The study may be discontinued at any time by the ACTG, IRB/EC, FDA, NIAID, OHRP, other country-specific government agencies as part of their duties to ensure that research participants are protected (as appropriate), or the industry supporter(s).

14.0 PUBLICATION OF RESEARCH FINDINGS

Publication of the results of this trial will be governed by ACTG policies. Any presentation, abstract, or manuscript will be made available for review by the industry supporter(s) prior to submission.

15.0 BIOHAZARD CONTAINMENT

As the transmission of SARS-CoV-2 and other pathogens can occur through contact with contaminated needles, respiratory secretions, blood, and blood products, appropriate blood and secretion precautions will be employed by all personnel in the drawing of blood and shipping and handling of all specimens for this study, as currently recommended by the CDC and the National Institutes of Health.

All dangerous goods and materials, including diagnostic specimens and infectious substances, must be transported using packaging mandated by CFR 42 Part 72. Please refer to instructions detailed in the International Air Transport Association (IATA) Dangerous Goods Regulations.

16.0 REFERENCES

1. Tay MZ, Poh CM, Renia L, MacAry PA, Ng LFP. The trinity of COVID-19: immunity, inflammation and intervention. Nat Rev Immunol 2020;20:363-74. PMID: 32346093.
2. COVID-19 Dashboard by the Center for Systems Science and Engineering (CSSE) at Johns Hopkins University (JHU) [Educational]; 2020 [updated 2020/05/03/]. Available from: http://coronavirusstatistics.org/.
3. ESRI StoryMaps Team. Mapping the Wuhan coronavirus outbreak [February 18, 2020]. Available from: https://storymaps.arcgis.com/stories/4fdc0d03d3a34aa485de1fb0d2650ee0.
4. Sheahan TP, Sims AC, Graham RL, et al. Broad-spectrum antiviral GS-5734 inhibits both epidemic and zoonotic coronaviruses. Sci Transl Med 2017;9(396):eaal3653.
5. Beigel JH, Tomashek KM, Dodd LE, et al.; ACTT-1 Study Group Members. Remdesivir for the treatment of Covid-19 -- final report. N Engl J Med 2020;383:1813-26.
6. Chang D, Mo G, Yuan X, et al. Time kinetics of viral clearance and resolution of symptoms in novel coronavirus infection. Am J Respir Crit Care Med 2020;201(9):1150-2.
7. Jagannathan P, Andrews JR, Bonilla H, et al. Peginterferon Lambda-1a for treatment of outpatients with uncomplicated COVID-19: a randomized placebo-controlled trial. Medixrv preprint posted November 23, 2020. https://doi.org/10.1101/2020.11.18.20234161.
8. Hung IF, Lung KC, Tso EY, et al. Triple combination of interferon beta-1b, lopinavir-ritonavir, and ribavirin in the treatment of patients admitted to hospital with COVID-19: an open-label, randomised, phase 2 trial. Lancet 2020;395(10238):P1695-1704.
9. FDA News Release. Coronavirus (COVID-19) Update: FDA Authorizes Monoclonal Antibody for Treatment of COVID-19. November 09, 2020. Available from: https://www.fda.gov/news-events/press-announcements/coronavirus-covid-19-update-fda-authorizes-monoclonal-antibody-treatment-covid-19
10. FDA News Release. Coronavirus (COVID-19) Update: FDA Authorizes Monoclonal Antibodies for Treatment of COVID-19. November 21, 2020. Available from: https://www.fda.gov/news-events/press-announcements/coronavirus-covid-19-update-fda-authorizes-monoclonal-antibodies-treatment-covid-19#:~:text=The%20EUA%20was%20issued%20to%20Regeneron%20Pharmaceuticals%20Inc.,biological%20products%20for%20human%20use,%20and%20medical%20devices
11. VECKLURY® (remdesivir) Package Insert. Reference ID 4690158. Issued 10/2020. Gilead, Foster City, CA. https://www.accessdata.fda.gov/drugsatfda_docs/label/2020/214787Orig1s000lbl.pdf
12. Coronavirus Disease 2019 (COVID-19) Treatment Guidelines [Government]; 2020. Available from: https://www.covid19treatmentguidelines.nih.gov/introduction/
13. The Adaptive Platform Trials Coalition. Adaptive platform trials: definition, design, conduct and reporting considerations. Nat Rev Drug Discov 2019;18:797-807.
14. NIH to launch public-private partnership to speed COVID-19 vaccine and treatment options: NIH 2020 [updated 2020/04/16/T16:30:35-04:00; cited 2020 05/03]. Available from: https://www.nih.gov/news-events/news-releases/nih-launch-public-private-partnership-speed-covid-19-vaccine-treatment-options.
15. Hwang IK, Shih WJ, DeCani JS. Group sequential designs using a family of type I error probability spending functions. Stat Med 1990;9:1439-45.
16. East 6.5 User Manual (Software Version 6.5). Cytel Inc., Waltham, MA, USA; 2018.
17. **FDA Guidance on Conduct of Clinical Trials of Medical Products During the COVID-19 Public Health Emergency: Guidance for Industry, Investigators, and Institutional Review Boards, March 2020, Updated on January 27, 2021. Accessed at: https://www.fda.gov/media/136238/download**

APPENDIX I: SAMPLE INFORMED CONSENT – MAIN PROTOCOL

DIVISION OF AIDS
AIDS CLINICAL TRIALS GROUP (ACTG) SAMPLE INFORMED CONSENT
FOR PROTOCOL: ACTIV-2 / A5401

Adaptive Platform Treatment Trial for Outpatients with COVID-19, FINAL Version 5.0

SHORT TITLE FOR THE STUDY: Adapt Out COVID

SUMMARY

PURPOSE	This is a research study and your participation in this study is voluntary. The purpose of this study is to evaluate the ability of various drugs to improve health outcomes for people with COVID-19. We also want to see if these drugs are safe, and if these drugs can stop the disease process and prevent hospitalization. This study is designed to quickly identify safe and effective drugs that can treat COVID-19.
STUDY DRUG	Study drug will be either an active drug or a placebo. A placebo looks like a “real” drug, but it does not have any active medication in it.<br><br>As drugs are recommended for the treatment of COVID-19 symptoms, some of them will be selected for testing in this study. Therefore, there may be different drugs being used as part of the study at different times. You will receive information about specific drugs being tested at this time in a separate consent form. Regardless of how many study drugs are being tested, you will only receive one study drug (or placebo).<br><br>If, during the course of the study, a standard treatment for COVID-19 is identified, that treatment will be substituted for placebo.
NUMBER OF PARTICIPANTS	For each drug being tested, a minimum of 110 people will receive that drug and an equal or smaller number will receive placebo. If a drug appears to be safe and effective when 110 people have received it, then more people will be enrolled so that up to 1000 receive that drug. Again, an equal or smaller number will receive placebo.
LENGTH OF STUDY	Your participation in this study will last between 24 weeks (6 months) and 72 weeks (18 months), depending on which study drug you receive.
REQUIRED ACTIVITIES	If you are in this study, the following study procedures are required:<br>• you will record your symptoms<br>• you will provide blood samples<br>• you will provide self-collected nasal swab samples<br>• you may have nasopharyngeal swabs (i.e., deep nasal swabs) collected by a study staff person

RISKS	There are some risks that are specific to the study drug that you might receive. We will tell you about those risks in the second part of this consent process.
BENEFITS	If you take part in this study, there may be a direct benefit to you, but no guarantee can be made. It is also possible that you will receive no benefit from being in this study. Information learned from this study may help others who have COVID-19.
OTHER CHOICES	Instead of being in this study, you have the option of:<br>• treatment with prescription drugs available to you through your health care provider<br>• treatment with other experimental drugs, if you qualify<br>• no treatment

INTRODUCTION

You are being asked to take part in this research study because you have been diagnosed with SARS-CoV-2 and have symptoms of the disease it causes, which is commonly known as COVID-19. This study is sponsored by the National Institutes of Health (NIH). The doctor in charge of this study at this site is: (insert name of Principal Investigator). Before you decide if you want to be a part of this study, we want you to know about the study.

This is a consent form. It gives you information about this study. The study staff will talk with you about this information. You are free to ask questions about this study at any time. If you agree to take part in this study, you will be asked to sign this consent form. You will get a copy to keep.

WHY IS THIS STUDY BEING DONE?

SARS-CoV-2 is a new virus that has caused a widespread outbreak of an illness called COVID-19. In most people, it causes a mild to moderate symptoms, like a “cold”. In others, this virus can cause a pneumonia (an inflammation of the lungs), which can be serious and life threatening. There is no proven treatment for COVID-19 for people who are not sick enough to be hospitalized.

For each drug that is tested in this study, there could be two study parts. In the first part, we will see if the drug is safe. We will also see if it can decrease how long people have COVID-19 symptoms and if it can help get rid of SARS-CoV-2 virus more than the placebo. Drugs that appear to be safe and to work better than the placebo in the first part of the study will be tested in the second part of the study.

In the second part of the study, we will continue to test how safe the drug is. We will also continue to compare it to a placebo to see if it can reduce the number of people who have to go into the hospital or who die from COVID-19.

You will be told which part of the study is open for enrollment during this consent process. At each stage, new study drugs may be added (in other words, multiple study drugs may be studied at one time).

The study is designed to rapidly evaluate new therapies for COVID-19. This could mean that the study finds that a drug that you were started on will not be studied further. If this happens, we will tell you. If you agree we would like you to continue to participate in the study and have all of the study visits, but this is your choice. We will not ask you to stay on the study drug if early results suggest that the study drug is not safe.

If you are randomized to an active drug in the first part of the study that is selected to be tested in the second part of the study, you will not be notified of this decision.

WHAT DO I HAVE TO DO IF I AM IN THIS STUDY?

Location of Study Visits
Your study visits will take place in person or remotely. You and the staff at your site will discuss the location for each visit.

- In-person visits will take place at the clinic, at your home, or at another non-clinic location
- Remote visits will take place over the phone or via telemedicine systems approved for use at your site

Information Collected at Screening
There is some information that we collect on everyone who is screened for this study. As part of your screening visit, some demographic (for example, age, gender, race), clinical (for example, disease condition, diagnosis), and laboratory values will be collected from you.

We will collect this information even if you do not enroll in this study. This information is collected so that researchers may determine whether there are patterns and/or common reasons why people do not join a study.

Blood Drawn
The site staff can tell you how much blood will be collected at any particular visit. At most visits, the amount will be no more than *XX mL (x tablespoons)* of blood collected. At a few visits, up to *XX-XX mL (x-x tablespoons)* will be collected.

Screening Visit
If you would like to be in this study, after you have read and signed this consent form, you will have a screening visit to make sure you meet the requirements for joining the study. This visit will take about 1 hour.
At this visit:

- study staff will review your history and confirm that you have tested positive for SARS-CoV-2 infection.
- you will be asked about symptoms you are experiencing.
- study staff will ask you about any health conditions you have and questions about your health in general.
- study staff will ask you about your medication history and any medications you are taking.
- you may have a brief physical exam if your screening visit takes place in person.

Entry Visit
If you qualify for the study, you will have an entry visit. This visit might occur on the same day as your screening visit. At this visit, you will be randomly assigned (like flipping a coin or rolling dice) to a study group. You and the study staff will not be able to choose which treatment group you are in. You will not know whether you are receiving active drug or placebo. We will tell you more about the treatment groups that you might be in during the second part of this consent process.

Also at the Entry visit:

- you will have a physical exam and answer questions about your medical history and any medications you are taking or have taken in the past.
- you will be asked about symptoms you are experiencing.
- you will be asked about your smoking status and history.
- the study staff will ask if anyone else in your household has been diagnosed with SARS-CoV-2 infection.
- you will be asked to provide your home address.

- you will be asked to provide contact information for people the study staff could contact in case we cannot reach you for a study visit. You will need to tell these people that you are in the study, and that they could receive a call from study staff. If study staff cannot reach you after two tries (separated by 24 hours), they will call one of the people you have identified.
- you will be asked to provide your health care provider contact information, like your physician or commonly used clinic and hospital.
- you will receive a kit that includes information about the study, instructions and supplies for self-collection of certain samples, a diary in which you will record how you are feeling, instructions on what to do if you have worsening symptoms, and contact information for the study staff.
- you will complete your first entry in the study diary with the study staff to make sure that you understand how to complete the diary.
- a swab will be collected from your nose. This swab is used to detect viruses. You will place a swab in each nostril and rotate the swab several times. Study staff will provide you with further instructions about the nose swabs.
- you will have blood drawn. This blood will be used for the following tests:
    - to find out the levels of SARS-CoV-2 virus, inflammation markers, and clotting factors in your blood
    - for future protocol-required testing
- you will start study drug. Details of this are provided in the next part of the consent.

If you participate in the first part of the study:

- you will have a second swab collected from your nose. For this swab, the site staff will insert a different kind of swab into your nostril. The swab will be placed deep towards to the back of your throat. The swab will be left in place for several seconds and then slowly removed. This procedure is uncomfortable and it might make you gag or make your nose bleed.

Study Visits

After the Entry visit, your study visits and evaluations will be different depending on whether you are in the first part of the study or the second part of the study.

IF YOU ARE IN THE FIRST PART OF THE STUDY:

Daily on Days 1-14

You will collect a nose swab every day on days 1-14. On some of these days, you will collect the swab on your own and save it at home. You will record the time you collected your nose swab. You will be given instructions for how and when to return the swabs to the study staff.

Daily on Days 1-28

You will record your symptoms in your study diary at about the same time every day. If you are not feeling well, someone can help you by writing the responses down for you, but the responses should come from you.

You will receive a reminder every day on days 1-28 to complete your study diary. This reminder may be by telephone, text message, email, or other method that you give permission for.

Study Visits on Days 3, 7, 14, 28

At these visits:

- you will have a brief physical exam and answer questions about any medications you are taking.
- the study staff will ask you if there are any updates to the contact information for the people you have identified.
- you will review the entries in your study diary with study staff. On day 28, the study staff will collect your

diary.

- the study staff will ask you if anyone else in your household has been diagnosed with SARS-CoV-2 infection. (Day 28)
- you may have blood drawn. This blood will be used for the following tests:
  - to find out the levels of SARS-CoV-2 virus, inflammation markers, and clotting factors in your blood
  - for future protocol-required testing
- the site staff will collect a nasal swab as described above.
- you will also collect your own nasal swab.

Study Visits at Weeks 12 and 24

At these visits:

- you will have a brief physical exam (week 24)
- you will answer questions about any medications you are taking.
- at week 12, the study staff will ask you if there are any updates to the contact information for the people you have identified.
- the study staff will ask you if anyone else in your household has been diagnosed with SARS-CoV-2 infection.
- you will answer questions about any potential COVID-19 related symptoms or conditions you have experienced.
- at week 24, you will have blood drawn. This blood will be used for the following tests:
  - to find out the levels of inflammation markers and clotting factors in your blood
  - for future protocol-required testing

Additional Study Visits

Study visits may be required after week 24. This will depend on the study drug/placebo you received. Details are listed in the consent which discusses the study drug you might receive.

Genetic Testing *[sites remove this section if PBMCs are not collected at your site]*

Your body, like all living things, is made up of cells. Cells contain deoxyribonucleic acid, also known as “DNA”. DNA is like a string of information put together in a certain order. Parts of the string make up “genes”. Genes contain instructions on how to make your body work and fight disease. Differences or changes in DNA explain some of the physical differences among people. These differences partly explain why some people get diseases like cancer or diabetes while others do not. Genetic testing looks at the differences in people’s DNA. This testing also looks at how differences affect health and the body’s response to disease and treatment.

If you agree, some of your blood that is collected will be used to study whether there are genetic differences in how sick people get when they are infected with SARS-CoV-2 or how they respond to study drugs. This genetic testing might include might include whole genome sequencing (WGS). “Sequencing” is looking at the order of a person’s genes to see how this order is different from the order of most people.

You do not have to agree to participate in this genetic testing. Even if you do not agree, you can still participate in the rest of the study.

Please put your initials below to indicate your choice:

________ (initials) I understand and I agree to this use of my samples

OR

_________ (initials) I understand but I do not agree to this use of my samples

IF YOU ARE IN THE SECOND PART OF THE STUDY:

Days 3, 7, and 14
You will collect a nose swab on each of these days. You will collect the swabs on your own and save them at home. You will be given instructions for how and when to return the swabs to the study staff.

Daily on Days 1-28
You will record your symptoms in your study diary at about the same time every day. If you are not feeling well, someone can help you by writing the responses down for you, but the responses should come from you.

You will receive a reminder every day on days 1-28 to complete your study diary. This reminder may be by telephone, text message, email, or other method that you give permission for.

Study Visits on Days 3, 7, and 14
At these visits:
- you will answer questions about how you are feeling and any medications you are taking.
- the study staff will ask you if there are any updates to the contact information for the people you have identified.
- you will review the entries in your study diary with study staff.

Study Visit on Day 28
At this visit:
- you will have a brief physical exam and answer questions about any medications you are taking.
- the study staff will ask you if there are any updates to the contact information for the people you have identified.
- you will review the entries in your study diary with study staff and the study staff will collect your diary.
- the study staff will ask you if anyone else in your household has been diagnosed with SARS-CoV-2 infection.
- you will collect a swab from your nose as described above.
- you will have blood drawn. This blood will be used for the following tests:
  - to find out the levels of inflammation and clotting factors are in your blood
  - for future protocol-required testing

Study Visits at Weeks 12 and 24
At these visits:
- you will have a brief physical exam (week 24)
- you will answer questions about any medications you are taking.
- at week 12, the study staff will ask you if there are any updates to the contact information for the people you have identified.
- the study staff will ask you if anyone else in your household has been diagnosed with SARS-CoV-2 infection.
- you will answer questions about any potential COVID-19 related symptoms or conditions you have experienced.
- at week 24, you will have blood drawn. This blood will be used for the following tests:
  - for future protocol-required testing.

Additional Study Visits
Study visits may be required after week 24. This will depend on the study drug/placebo you received. Details are listed in the consent which discusses the study drug you might receive.

Early Discontinuation
If at any point in the study you want to stop participating in the study, you must contact the site immediately. The study doctor may ask you to continue to be part of the study and return for some study visits and procedures.

If you have not withdrawn consent but must discontinue participation in the study after starting study drug, the site will attempt to obtain information regarding vital status (whether you are living or have died) from other sources, such as family members, other secondary contacts that you have provided, or clinical records.

WILL I RECEIVE THE RESULTS OF ANY TESTS?

Some of the blood that is collected from you will be stored and tested later. Some of these tests will be done after you are done with the study, and other tests are not yet approved by the FDA and are still considered "research" tests. For these reasons, you will not receive the results of the tests to:
- check levels of SARS-CoV-2 in your blood and nasal swabs
- check how well your blood clots
- check the level of inflammation markers in your blood
- check if your body developed antibodies to SARS-CoV-2

You will be told of any new information learned during the course of the study that might cause you to change your mind about staying in the study. At the end of the study, you will be told when study results may be available and how to learn about them. As with all studies, if we find out important information that may affect your care, you will be provided with those results.

HOW MANY PEOPLE WILL TAKE PART IN THIS STUDY?

In the first part of the study, 110 people will receive each study drug and a similar number of people will receive placebo. If the study proceeds to the second part for a particular study drug, up to 1000 participants will receive that study drug and a similar number will receive placebo.

HOW LONG WILL I BE IN THIS STUDY?

You will be in this study between 24 weeks (6 months) and 72 weeks (18 months), depending on which study drug you receive

WHY WOULD THE DOCTOR TAKE ME OFF THIS STUDY EARLY?

The study doctor may need to take you off the study early without your permission if:
- the study is stopped or cancelled.
- your health care provider requests that you stop participating in the study.
- you do not receive the first dose of study drug when you start the study.

The study doctor may also need to take you off the study drug without your permission if:
- you are taking other medications that should not be taken with the study drug.
- continuing the study drug may be harmful to you.

If you must stop taking the study drug before you are finished with the study, the study doctor will ask you to continue to be part of the study and return for study visits and procedures.

WHAT HAPPENS IF I DECIDE TO PERMANENTLY STOP TAKING STUDY-PROVIDED MEDICATIONS?

If you must permanently stop taking study drug before your study participation is over, the study staff will discuss other options that may be of benefit to you.

WHAT HAPPENS WHEN I FINISH THE STUDY?

After you have completed your study participation, the study will not be able to continue to provide you with the study drug you received on the study. If continuing to take these or similar drugs/agents would be of benefit to you, the study staff will discuss how you may be able to obtain them.

WHAT ARE THE RISKS OF THE STUDY?

Risks of Study Drug
There are risks to taking part in any research study. The effectiveness of the study drug is not known. One risk is that the study drug may not stop you from becoming sicker, being hospitalized, or dying from SARS-CoV-2.

There is a risk of serious and/or life-threatening side effects when non-study medications are taken with the study drug. For your safety, you must tell the study doctor or nurse about all medications you are taking before you start the study.

There are some risks that are specific to the study drug that you might be assigned to. We will tell you about those risks in the second part of this consent process.

Risks of Blood Draw
Having blood drawn may cause some discomfort, bleeding, bruising, and/or swelling where the needle enters the body, and in rare cases it may result in fainting. There is a small risk of infection.

Risks of Nose Swabs
Nose swabs might make you gag or sneeze. They may also cause discomfort or cause your nose to bleed.

Effect on Future Vaccination
Vaccines against the virus that causes COVID-19 are becoming available. It is currently unknown how long people should wait to receive a COVID-19 vaccine after having COVID-19, since the body's own immune response may offer protection for several months. We also do not know how your body’s immune response to COVID-19 vaccines may be affected by the drugs being evaluated in this study. If there are potential effects and recommendations for a given study drug, they will be reviewed with you.

ARE THERE RISKS RELATED TO PREGNANCY AND BREASTFEEDING?

In the second part of the consent process we will tell you about the specific drugs that you might receive and whether they have any risks related to pregnancy and breastfeeding.

If you become pregnant while on study, the study staff would like to obtain information from you about the outcome of the pregnancy (even if it is after your participation in the study ends).

ARE THERE BENEFITS TO TAKING PART IN THIS STUDY?

If you take part in this study, there may be a direct benefit to you, but no guarantee can be made. It is also possible that you may receive no benefit from being in this study. Information learned from this study may help others who have COVID-19.

WHAT OTHER CHOICES DO I HAVE BESIDES THIS STUDY?

Instead of being in this study you have the choice of:

- treatment with prescription drugs available to you from your health care provider
- treatment with other experimental drugs, if you qualify
- no treatment
- There may be a COVID treatment available to you through a US FDA Emergency Use Authorization (EUA). Under an EUA, the FDA may allow unapproved medical products to be used in an emergency to diagnose, treat, or prevent serious or life-threatening diseases or conditions. Your site will tell you about any COVID treatments that might be available to you through an EUA.

Please talk to your doctor about these and other choices available to you. Your doctor will explain the risks and benefits of these choices.

WHAT ABOUT CONFIDENTIALITY?

For sites in the US

We will do everything we can to protect your privacy. In addition to the efforts of the study staff to help keep your personal information private, we have gotten a Certificate of Confidentiality from the US Federal Government. This certificate means that researchers cannot be forced to tell people who are not connected with this study, such as the court system, about your participation. Any publication of this study will not use your name or identify you personally.

Your records may be reviewed by the US Food and Drug Administration (FDA), the ACTG, the US Office for Human Research Protections (OHRP), or other local, US, and international regulatory entities as part of their duties, (insert name of site) institutional review board (IRB) (a committee that protects the rights and safety of participants in research), National Institutes of Health (NIH), study staff, study monitors, drug companies supporting this study, and their designees. Having a Certificate of Confidentiality does not prevent you from releasing information about yourself and your participation in the study.

Even with the Certificate of Confidentiality, if the study staff learns of possible child abuse and/or neglect or a risk of harm to yourself or others, we will be required to tell the proper authorities.

A description of this clinical trial will be available on ClinicalTrials.gov, as required by US law. This website will not include information that can identify you. At most, the website will include a summary of the results. You can search this website at any time.

For sites outside the US

Efforts will be made to keep your personal information confidential. We cannot guarantee absolute confidentiality. Your personal information may be disclosed if required by law. Any publication of this study will not use your name or identify you personally.

Your records may be reviewed by the US Food and Drug Administration (FDA), the ACTG, the US Office for Human Research Protections (OHRP), or other local, US, and international regulatory entities as part of their duties (insert name of site) institutional review board (IRB) or Ethics Committee (a committee that protects the rights and safety of participants in research), National Institutes of Health (NIH), study staff, study monitors, drug companies supporting this study, and their designees.

A description of this clinical trial will be available on ClinicalTrials.gov, as required by US law. This website will not include information that can identify you. At most, the website will include a summary of the results. You can search this website at any time.

All information collected about you as part of the study will be sent securely to the ACTG statistical and data management center in the United States for combining with information from other study participants and statistical analysis of study results. Your name and other personal identifiers will not be sent. Your research site is responsible for sending your information in accordance with the laws, regulations and policies of your country and research site.

WHAT IF THE SITE CAN NO LONGER REACH ME DURING THE STUDY?

If you cannot be reached after two attempts to contact you (with 24 hours between attempts), study staff may try to contact you through the family, friends, or acquaintances you provided at screening and updated at each visit.

If you are still unable to be reached, we will attempt to obtain information about your status (whether you are living or have died) by contacting your health care provider (if you agree) or by accessing publicly available records (you do not have to give your permission for us to access these records).

WHAT ARE THE COSTS TO ME?

There will be no cost to you for study-related visits or procedures. If you require medical care as a result of taking study drug, it is possible that your insurance company will not pay for these costs because you are taking part in a research study. Costs related to acute care/hospitalization will not be covered by the study.

WILL I RECEIVE ANY PAYMENT?

[Insert site-specific information on compensation to study participants.]

WHAT HAPPENS IF I AM INJURED?

If you are injured as a result of being in this study, you will be given immediate treatment for your injuries.

[Sites: Please modify (if necessary) and insert one of these two statements, as appropriate to your site. If your site is required to carry CTI, this must be indicated in the informed consent.

- this site has clinical trials insurance. This insurance will allow the site to provide you with monetary compensation if you suffer harm as a result of participating in this research study.
  OR
- the cost for this treatment will be charged to you or your insurance company. There is no program for compensation either through this institution or the NIH.]

The US federal government has a program that may provide compensation to you or your family if you experience serious physical injuries or death and these costs are not covered by other payors. To find out more

about this "Countermeasures Injury Compensation Program" go to https://www.hrsa.gov/cicp/about/index.html or call 1-855-266-2427.

Due to the coronavirus public health crisis, the US federal government has issued an order that may limit your right to sue and recover for losses if you are injured or harmed while participating in this COVID-19 clinical study. If the order applies, it limits your right to sue and recover for losses from the researchers, healthcare providers, any study sponsor or manufacturer or distributor involved with the study. However, the order does not limit your right to seek compensation for injuries that result from conduct or activities of the researchers, health care providers, study sponsors, manufacturers, and distributors that is unrelated to the study.

You will not be giving up any of your legal rights by signing this consent form.

WHAT ARE MY RIGHTS AS A RESEARCH PARTICIPANT?

Taking part in this study is completely voluntary. You may choose not to take part in this study or leave this study at any time. Your decision will not have any impact on your participation in other studies and will not result in any penalty or loss of benefits to which you are otherwise entitled.

We will tell you about new information from this or other studies that may affect your health, welfare, or willingness to stay in this study. If you want the results of the study, let the study staff know.

WHAT DO I DO IF I HAVE QUESTIONS OR PROBLEMS?

For questions about this study or a research-related injury, contact:

- name of the investigator or other study staff
- telephone number of above

For questions about your rights as a research participant, contact:

- name or title of person on the Institutional Review Board (IRB) or other organization appropriate for the site
- telephone number of above

Contacting Your Health Care Provider

[Sites modify per local requirements for obtaining health care records.]

With your permission, for which you would need to sign a waiver, study staff may contact your health care provider or hospital(s) where you might receive care to determine if you have been hospitalized or died while in the study, and the cause of death. You can still participate in this study even if you do not give us permission to contact your health care provider or hospital(s).

Will you allow us to contact your health care provider or hospital(s) to obtain this information?

________ YES

________NO

________Initials

If you said Yes, please list the names of your health care provider and the hospitals you would likely be admitted to, below:

____
____
____
____

SIGNATURE PAGE

If you have read this consent form (or had it explained to you), all your questions have been answered and you agree to take part in this study, please sign your name below.

Participant's Name (print)	Participant's Signature and Date
Participant's Legally Authorized Representative (As appropriate)Signature and Date	Legally Authorized Representative (print)
Study Staff Conducting	Study Staff's Signature and Date Consent Discussion (print)
Witness's Name (print)	Witness's Signature and Date (As appropriate)

ATTACHMENT A: CONSENT FOR USE OF EXTRA SAMPLES

When samples are no longer needed for this study, the ACTG may want to use them in other studies and share them with other researchers. These samples are called “extra samples.” The ACTG will only allow your extra samples to be used in other studies if you agree to this. If you have any questions, please ask.

Identifiers will be removed from your samples and from any private information that has been collected about you. This means that no one looking at the labels or at other information will be able to know that the samples or information came from you.

Extra samples are stored in a secure central place called a repository. Your samples will be stored in the ACTG repository located in the United States.

There is no limit on how long your extra samples will be stored. *[Site: Revise the previous sentence to insert limits if your regulatory authority imposes them.]*

When a researcher wants to use your samples and information, the research plan must be approved by the ACTG. Also, the researcher’s institutional review board (IRB) or ethics committee (EC) will review the plan. *[Site: If review by your institution’s IRB/EC/RE is also required, insert a sentence stating this.]* IRBs/ECs protect the rights and well-being of people in research. If the research plan is approved, the ACTG will send your samples to the researcher’s location. This means that researchers who are not part of the protocol team may use your samples without asking you again for your consent.

You will not be paid for your samples. Also, a researcher may make a new scientific discovery or product based on the use of your samples. If this happens, there is no plan to share any money with you.

You may withdraw your consent for research on your extra samples at any time and the specimens will be discarded.

Please choose the response that matches what you want by putting your initials in the space provided. Please ask the staff any questions that you have before you indicate your selection.

<u>Research without Human Genetic Testing</u>
If you agree, your extra samples may be stored (with usual protection of your identity) and used for ACTG-approved research that does not include human genetic testing.

________ (initials) I understand and I agree to this storage and possible use of my samples.

OR
________ (initials) I understand but I do not agree to this storage and possible use of my samples.

APPENDIX VI: INVESTIGATIONAL AGENT AZD7442 INTRAVENOUS ADMINISTRATION

Information/evaluations noted in this agent-specific appendix are IN ADDITION to those presented in the master protocol. Section numbering aligns with the master protocol.

SCHEMA

DURATION: 72 weeks

2.0INTRODUCTION

2.2Rationale

Monoclonal Antibodies (mAbs)
Sera obtained from persons or animals who recovered from a particular infection has shown prophylactic and therapeutic potential for a variety of infections, and Emil von Behring won the Nobel Prize in 1893 for his work on use of immune serum from the blood of infected animals to provide immunity to diphtheria [1]. Currently, hyperimmune human sera immunoglobulin is still used to treat many viral infections including cytomegalovirus (CMV), respiratory syncytial virus (RSV), hepatitis A virus (HAV), hepatitis B virus (HBV), and rabies [2].

Unfortunately, heterologous sera were associated with a variety of complications including serum sickness and hypersensitivity, which significantly limited its usefulness clinically [3]. Given the long history of use of antibodies for infectious diseases, monoclonal antibodies (mAbs) were developed. Improved purification techniques and the ability to engineer humanized mAbs allowed for the development of broadly reactive and potent mAbs, which helped reduce some of the issues that hampered the utility of heterologous sera [3, 4]. In fact, current technology allows mAbs to be produced requiring only tissue culture or microbial expression systems, thus the potential toxicity of humanized mAbs is comparable to antibiotics [2, 4].

Engineered humanized and human mAbs have shown considerable efficacy for viral infections. The first was palivizumab in 1998, which is used for RSV [5]. Monoclonal antibodies have also been quickly developed for emerging infections such as Ebola [6]. As a part of the massive scientific effort to stop COVID-19, mAbs have been developed for treatment of COVID-19. These agents now need to be evaluated in rigorous randomized clinical trials.

The limitations of mAbs continue to be cost and that these antibodies are perishable, require refrigeration, and must be administered parenterally [4]; however, their use may still be useful in the outpatient setting, as one dose often stays in the therapeutic range ‘for months [5], potentially allowing an entire treatment course with a single administration.
A number of viral infectious diseases have been successfully treated with mAbs, including RSV and HIV. Some of these mAbs were derived from persons who were infected with these viruses and mounted neutralizing humoral responses.

An investigational agent to be evaluated in this trial will be the mAb AZD7442 delivered by IV infusion and made by AstraZeneca Pharmaceuticals LP for the treatment of early, symptomatic SARS-CoV-2 infection.

Investigational Agent
Background
AZD7442 is a combination of two human mAbs, AZD8895 and AZD1061. Both were cloned from B-cells isolated from peripheral blood mononuclear cells (PBMCs) obtained from COVID-19 convalescent

patients. These mAbs bind to unique, non-overlapping epitopes at the human angiotensin-converting enzyme 2 (hACE2) interface of the receptor binding domain (RBD) of the Spike (S) protein of SARS-CoV-2, preventing viral entry into human cells and its subsequent viral replication. The two antibodies in the combination contain modifications in their Fc regions that extend their anticipated half-life up to 70-130 days [3-6] and reduce the risk of antibody dependent enhancement (ADE), by limiting binding to cellular Fc gamma receptors [7]. The combination of two mAbs with differing binding sites on the RBD is intended to reduce the probability of viral mutations that would confer antibody resistance, and to provide synergy in their virus neutralizing activity.

AZD7442 is expected to result in a clinically important decrease of viral replication, mitigating the severity of COVID-19 in persons with the infection in whom ongoing viral replication is the primary driver of pathophysiology. The potential reduction in viral replication may also decrease a treated person's extent and duration of viral shedding and transmission, thus potentially positively impacting public health.

Non-Clinical Studies: Pharmacokinetics (PK)

Nonclinical studies of AZD7442 have been performed in mice and non-human primates (NHPs). In human Fcn transgenic Tg32 mice, peak serum concentrations at 28 days post intravenous administration of AZD7442 components remained well above the EC50s and EC99s determined in cellular infection assays [8]. The toxicokinetic profile of AZD7442 (AZD8895 and AZD1061) following IV or IM administration is being evaluated in cynomolgus monkeys as part of a GLP toxicology study. In this GLP toxicology study for AZD7442 high exposures were achieved and were very consistent across animals and between males and females for both AZD8895 and AZD1061, for both the 300 mg/kg IV dose and the 75 mg/kg IM dose of each antibody [8]. Based on the data available over the first 2 weeks after dosing, the safety exposure margin for the clinical 300 mg IV AZD7442 dose against the IV NOAEL of 600 mg/kg AZD7442 is 103-fold and 150-fold for $AUC_{(0\text{-}4\ weeks)}$ and C_{max}, respectively [8].

Non-Clinical Studies: Antiviral Effects

Murine models of SARS-CoV-2 have been performed to study the prophylactic and post-exposure antiviral activity of the AZD7442. In these models, the parenteral formulations of AZD7442 components were studied: COV2-2196 and COV2-2130, the respective parental antibodies of AZD8895 and AZD1061. COV2-2196 and COV2-2130 lack the Fc region modifications but are expected to retain antiviral activity. In a mouse-adapted-SARS-CoV-2 model, BALB/c mice were inoculated via intranasal route with 10^5 fluorescent focus units (FFU) of MA-SARS-CoV-2 and COV2-2196 and COV2-2130 (1:1 cocktail) administered intravenously 12 hours after. Viral burden in the lungs was measured 2 days post infection (dpi) after viral challenge using RT-qPCR or plaque assay. Mice were monitored daily for body weight change. Administration of the mAbs 12 hours post infection in Ad5-hACE2 transduced mice resulted in neutralization of infectious virus in the lungs. The mAbs were also evaluated for in vivo efficacy in an immuno-competent model using a mouse-adapted-SARS-CoV-2 virus. In this model there was significant viral replication in the lungs but little or no clinical disease [9].

The parental mAb formulations were also assessed in a NHP model [10, 11]. Rhesus macaques received one 50 mg/kg dose of COV2-2196 or isotype control antibody intravenously 3 days prior to intranasal and intratracheal challenge with a total dose of 10,000 PFU SARS-CoV-2. Virus replication was quantitated by RT-qPCR for viral sgmRNA, which measures replicative viral RNA intermediates. Animals that received isotype control antibody showed a median peak of 7.53 log10 sgmRNA copies/swab in nasal swab and a median peak of 4.97 log10 sgmRNA copies/mL in bronchoalveolar lavage. In contrast, viral sgmRNA was not detected in either nasal or bronchoalveolar lavage samples from animals that received COV2-2196. A PK analysis revealed similar concentrations of circulating human mAbs in animals from both groups.

Human Clinical Studies

The first in-human clinical studies of AZD7442 began enrolling in August 2020. (NCT04507256). Both IV

(300 mg, 1000 mg, and 3000 mg), sequentially and co-administered, and IM (300 mg) administration have been studied in this phase I, single-dose, dose-escalating trial among healthy adults. As of early December, **50** persons in this study have received the product at doses up to 3000 mg IV, and no safety concerns have emerged. **Specifically, there were no deaths, SAEs or discontinuation of study product due to AEs and no laboratory events of concern. Pharmacokinetic data from this phase I study also demonstrate the persistence of neutralizing antibody levels at 90 days post-infusion in all participants, with dose-dependent concentrations observed.** As described below, pharmacokinetic data have demonstrated relative levels achieved with IV and IM dosing.

The proposed adaptive Phase II/III trial is likely to be the first administration in persons with COVID-19 disease, although pre-exposure and post-exposure prophylaxis studies have started. **These include clinical studies PROVENT (pre-exposure), STORM CHASER (post-exposure) and TACKLE (treatment with 600 mg IM).** As of December 8,
2020, there was a single SAE in a pre-exposure prophylaxis participant who fainted following product administration (IM) and required evaluation at the hospital.

Choice of Study Dosing
Human efficacious doses for AZD7442 were evaluated using in vitro potency data (virus neutralizing activity of AZD7442 against SARS-CoV-2) and PK data. In addition, a viral-dynamic model was developed, which allowed for understanding of the pharmacodynamic effects of AZD7442 to inhibit a SARS-CoV-2 infection and the resulting immune response. The viral-dynamic model indicates that assuming a partition ratio ranging between 0.1- 1.0% for lung epithelial lining fluid-to-serum and assuming potency with an IC_{80} (inhibiting SARS-CoV-2 by 80%) of 40 ng/mL, the estimated effective concentration may be as low 4 µg/mL in serum. The dosing in Phase I studies therefore targeted 20-40 µg/m to assure sufficient levels in patients with an active SARS-CoV-2 infection; 300 mg AZD7442 IV administration before the time of peak viral load (on average ~ 7 days after day of infection is expected to result in reduction of the peak viral load and earlier eradication of the viral load. Administration of 300 mg IV AZD7442 after the peak viral load has been reached, is still expected to result in earlier viral load eradication compared to when drug is not present.

Figure 1 shows the preliminary serum AZD7442 concentration through 30 days post dose for the different single doses tested, as well as the predicted concentration time course for a single 600mg IM dose by multiplying the concentrations for the 300 mg IM dose by 2. The median time of maximum drug levels still needs to be defined when more data are available but initial data suggests that the T_{max} can be as late as 30 days post dose when administered in the ventrogluteal muscle.

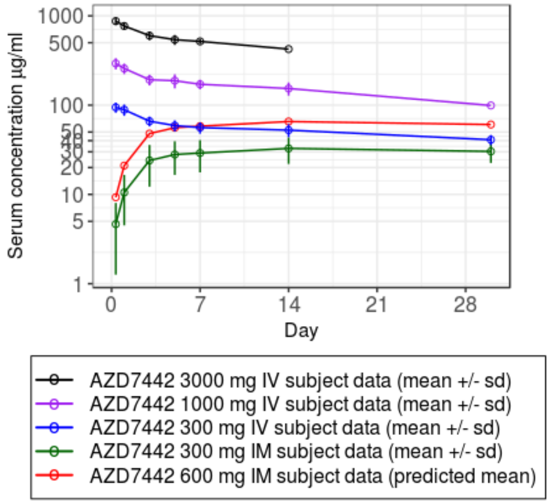

Figure 1: Observed Mean (SD) Serum AZD7442 concentrations over the first 30 days in Adult Healthy Volunteers in the Phase 1 Study

4.0SELECTION AND ENROLLMENT OF PARTICIPANTS

Participants must meet inclusion and exclusion criteria from the master protocol, as well as the appropriate inclusion and exclusion criteria for the investigational agent included below.

4.1General Eligibility Criteria

4.1.1 Inclusion Criteria

4.1.1.9 Meet the protocol definition of being at “higher” risk of progression to severe COVID-19 (see Schema, Population)

4.1.1.10 For participants who are of reproductive potential, negative serum or urine pregnancy test within 48 hours prior to study entry by any clinic or laboratory that has a CLIA certification or its equivalent, or by a point of care (POC)/CLIA-waived test.

Reproductive potential is defined as:

- participants who have reached menarche
- participants who have not been post-menopausal for at least 12 consecutive months with follicle-stimulating hormone (FSH) ≥40 IU/mL or 24 consecutive months if an FSH is not available
- participants who have not undergone surgical sterilization (e.g., hysterectomy, bilateral oophorectomy, bilateral tubal ligation, or bilateral salpingectomy)
- participants with no other clinical conditions (such as anorexia nervosa) that could induce amenorrhea
- participants not taking medications such as oral contraceptives, hormones, gonadotropin-releasing hormone, anti-estrogens, selective estrogen receptor modulators (SERMs) or chemotherapy that could induce amenorrhea
- For individuals with permanent infertility due to an alternate medical cause (e.g., Mullerian agenesis, androgen insensitivity), investigator discretion should be applied to determining study entry.

4.1.1.11 If participating in sexual activity that could lead to pregnancy, participants who are of reproductive potential must agree to use highly-effective contraception for 24 weeks after investigational agent is administered. Highly-effective contraception includes oral contraceptives, implanted contraceptives, and intrauterine devices.

NOTE: Participants not of reproductive potential are eligible without requiring the use of a contraceptive method. Participant-reported history is acceptable documentation of surgical sterilization and menopause, including vasectomy in a sole partner.

4.1.1.12 Participants that engage in sexual activity that may lead to pregnancy in their partner must agree to either remain abstinent or use male contraceptives. They are also strongly advised to inform their non-pregnant sexual partners of reproductive potential to use effective contraceptives for 24 weeks after investigational agent is administered.

Participants with pregnant partners should use condoms during vaginal intercourse through 24 weeks after investigational agent administration.

Participants should refrain from sperm donation for 24 weeks after investigational agent administration.

4.1.2 Exclusion Criteria

4.1.2.11 Currently pregnant or breastfeeding

5.0 INVESTIGATIONAL AGENTS

5.1 Regimen, Administration, and Duration

5.1.1 Regimen and Duration

Participants will be randomized to receive one of the following regimens:

Investigational Agent: AZD7442, 300 mg (AZD8895, 150 mg PLUS AZD1061, 150 mg) to be administered intravenously (IV) for one dose at study Entry/Day 0.

OR

Placebo for AZD7442: 0.9% Sodium Chloride Injection, USP, to be administered IV for one dose at study Entry/Day 0.

5.1.2 Administration

AZD7442/Placebo to be administered IV over approximately 15 minutes at a rate of 20 mg/minute.

Prior to administration, the infusion solution must be allowed to equilibrate to room temperature. An infusion set containing low protein binding 0.2 or 0.22 µm in-line filters must be attached and

primed per institutional procedures. The entire contents of the IV bag must be infused to the participant. After the entire contents of the IV bag have been administered, flush the catheter with 5 mL of 0.9% Sodium Chloride Injection, USP and flush the infusion line as per site requirements to ensure the full dose is administered. Infusion time does not include the final flush time.

5.2 Formulation, Storage, and Preparation

5.2.1Formulation and Storage

AZD7442 consists of two independent drug substances, AZD8895 and AZD1061, which are formulated separately. Both AZD8895 and AZD1061 are supplied as a 100 mg/mL aqueous solution with 150 mg (nominal) of active investigational product in 10R glass vials with a volume of 1.5 mL. The aqueous solutions are colorless to slightly yellow, clear to opalescent. AZD8895 and AZD1061 vials must be stored between 2°C to 8°C (refrigerated storage) until use. AZD7442 is described in further detail in AZD7442 Investigator’s Brochure.
Placebo for AZD7442 is 0.9% Sodium Chloride Injection, USP. The product must be locally sourced and stored according to the manufacturer’s recommendation.

5.2.2. Preparation

Pharmacists must follow appropriate aseptic technique and consider sterile preparation procedures/guidance as outlined in USP General Chapter <797> Pharmaceutical Compounding – Sterile Preparations. Pharmacists must also follow the requirements of their country, institution, and pharmacy regulatory authority regarding these procedures. The investigational agent and placebo should be prepared in a sterile environment, utilizing a biosafety cabinet/isolator. If a biosafety cabinet or isolator is not available, a laminar flow hood may be used. Local regulations and site institutional policies and procedures for use of personal protective equipment, such as gloves, gowns, face masks, and safety glasses, must be followed.

Any unused portion of investigational agent must not be used for another participant. Any empty vials, unused portion of entered vials, or unused solution which contains investigational agent should be discarded in a biohazard containment bag and incinerated or autoclaved in accordance with institutional or pharmacy policy.

5.2.2.1.1 AZD7442

1. Remove one (1) vial of AZD8895 and one (1) vial of AZD1061 from the refrigerator, and an appropriately sized IV bag of 0.9% Sodium Chloride Injection USP. The target volume of the 0.9% Sodium Chloride Injection, USP IV bag is 100 mL, however, a range of 50 to 250 mL volumes can be utilized if a 100 mL IV bag is not available.
2. Using an appropriately sized syringe and needle, withdraw 1.5 mL of AZD8895 from the AZD8895 vial and inject the contents into the IV bag with 0.9% Sodium Chloride Injection, USP. Gently mix the contents until visually uniform. When the stopper of the vial is punctured to start preparation, record this time as the investigational agent preparation time. Assign a 4 hour beyond use date and time from the preparation time if stored at room temperature or a 24 hour beyond use date and time from the preparation time if stored at refrigerated conditions.
3. Using an appropriately sized syringe and needle, withdraw 1.5 mL of

AZD1061 from the AZD1061 vial and inject the contents into the same IV bag with 0.9% Sodium Chloride Injection, USP and AZD8895 prepared in Step 2. Gently mix the contents until visually uniform.
4. Place an opaque cover over the IV bag.

5.2.2.1.2 Placebo for AZD7442

1. Remove an appropriately sized IV bag of 0.9% Sodium Chloride Injection, USP. The target volume of the 0.9% Sodium Chloride Injection, USP IV bag is 100 mL, however, a range of 50 to 250 mL volumes can be utilized if a 100 mL IV bag is not available. The IV bag used must be the same size as the IV bag used for the preparation of the investigational agent.
2. Remove a second container of 0.9% Sodium Chloride Injection, USP. Using an appropriately sized syringe and needle withdraw 3 mL of 0.9% Sodium Chloride Injection, USP from this container and inject the contents into the IV bag obtained in Step 1.
3. Assign a 4 hour beyond use date and time from the preparation time if stored at room temperature or a 24 hour beyond use date and time from the preparation time if stored at refrigerated conditions.
4. Place an opaque cover over the IV bag.

5.2.3Labeling of Investigational Agent and Placebo

Label the prepared IV bag with the following information:
a. Participant identifier(s)
b. Protocol number: ACTIV-2/A5401
c. Investigational agent names: AZD7442 300 mg or Placebo
d. Total volume: 100 mL (or appropriate size within range of 50 to 250 mL dependent on availability)
e. Route: IV
f. Infusion rate/time: 20 mg/minute over approximately 15 minutes
g. Preparation date and time
h. Beyond use date and time: 4 hours at room temperature conditions or 24 hours at refrigerated conditions after preparation
i. Any additional information required by jurisdiction

5.3Supply, Distribution, and Accountability

5.3.1Supply/Distribution

AZD7442 will be manufactured by Catalent for AstraZeneca and will be available through the NIAID Clinical Research Products Management Center (CRPMC). The site pharmacist will receive ordering instructions for AZD8895 and AZD1061 vials from the NIAID CRPMC.

0.9% Sodium Chloride Injection, USP, infusion sets, and any other ancillary supplies will be locally sourced by the site.

5.3.2 Accountability

The site pharmacist is required to maintain complete records of all investigational agents received from the NIAID CRPMC and subsequently dispensed. At US CRSs, all unused investigational agents must be returned to the NIAID CRPMC (or as otherwise directed by the sponsor) after the study is completed or terminated. At non-US CRSs, the site pharmacist must follow the instructions provided by the CRPMC for the destruction of unused investigational agents.

5.4 Concomitant Medications

Any pre-medications given will be documented as a concomitant medication. There are no known or expected drug-drug interactions with the investigational agent and therefore there no prohibited medications except as outlined in section 5.4 of the parent protocol.

6.0 CLINICAL AND LABORATORY EVALUATIONS

6.1 Schedule of Evaluations. The schedules of evaluations provided below include all the evaluations in the master protocol and additional evaluations for this investigational agent.

Table 6.1-1: Schedule of Evaluations Phase II

Phase II Evaluation	Screening	Study Entry/Day 0	Day 3	Day 7	Day 14	Day 28	Week 12	Week 24	Week 36	Week 48	Week 72	Premature Study D/C (Before Day 28 Visit)	Premature Study D/C (After Day 28 Visit)
Visit Window			+/-1 day	+/-2 days		+4 days	-7/+14 days						
Documentation of SARS-CoV-2 Infection	X												
COVID-19 Symptom Screen	X	X											
Medical/Medication History	X	X											
Smoking Status		X											
Clinical Assessments	X	X	X	X	X	X	X	X	X	X	X	X	X
Collect/Update Secondary Contacts		X	X	X	X	X	X						
Vital Status Check		If Participant Cannot be Reached per section 6.3.8											
Investigational Agent Administered		X											
Study Kit Dispensed		X											
Participant-Completed Study Diary		Every Day through Day 28											

Phase II Evaluation	Screening	Study Entry/Day 0	Day 3	Day 7	Day 14	Day 28	Week 12	Week 24	Week 36	Week 48	Week 72	Premature Study D/C (Before Day 28 Visit)	Premature Study D/C (After Day 28 Visit)
Visit Window			+/-1 day	+/-2 days		+4 days	-7/+14 days						
Study Diary Reminder		Days 1- 28											
Staff Review of Study Diary		X	X	X	X	X						X	
Retrieval of Study Diary						X						X	
Post-Acute COVID-19 Assessment							X	X	X	X	X		X
Household Infection and Linkage Report		X				X	X	X				X	X
Self-Collected Anterior Nasal Swab		Every Day through Day 14				X						X	
Retrieval of Self-Collected Anterior Nasal Swabs			Follow Instructions in MOP									X	
Staff-Collected NP Swab		X	X	X	X	X						X	
Blood Plasma for SARS-CoV-2 RNA		X		X								X	
Inflammatory Markers		X		X		X		X					
Coagulation Markers		X		X		X		X					
Hematology		X	X		X	X						X	
Chemistry		X	X		X	X						X	

Phase II Evaluation	Screening	Study Entry/Day 0	Day 3	Day 7	Day 14	Day 28	Week 12	Week 24	Week 36	Week 48	Week 72	Premature Study D/C (Before Day 28 Visit)	Premature Study D/C (After Day 28 Visit)
Visit Window			+/-1 day	+/-2 days		+4 days	-7/+14 days						
Pregnancy Testing	X		Whenever Pregnancy Suspected										
Stored Plasma		X		X		X		X				X	X
Stored Serum		X		X		X		X				X	X
Stored PBMCs (Selected Sites)		X		X		X		X				X	
PK Studies		X[1]	X	X	X	X	X	X				X	X
Antidrug Antibodies		X			X	X	X	X				X	X

[1] First PK serum sample to be obtained prior to infusion along with remainder of entry labs. A second PK sample to be obtained at the completion of the infusion

Table 6.1-2: Schedule of Evaluations Phase III

Phase III Evaluation	Screening	Study Entry/Day 0	Day 3	Day 7	Day 14	Day 28	Week 12	Week 24	Week 36	Week 48	Week 72	Premature Study D/C (Before Day 28)	Premature Study D/C (After Day 28)
Visit Window			+/-1 day	+/-2 days		+4 days	-7/+14 days						
Documentation of SARS-CoV-2 Infection	X												
COVID-19 Symptom Screen	X	X											
Medical/Medication History	X	X											
Smoking Status		X											
Clinical Assessments	X	X	X	X	X	X	X	X	X	X	X	X	X
Collect/Update Secondary Contacts		X	X	X	X	X	X						
Vital Status Check		If Participant Cannot be Reached per section 6.3.8											
Investigational Agent Administered		X											
Study Kit Dispensed		X											
Participant-Completed Study Diary		Every Day through Day 28											
Study Diary Reminder		Days 1- 28											
Staff Review of Study Diary		X	X	X	X	X						X	
Retrieval of Study Diary						X						X	

Phase III Evaluation	Screening	Study Entry/Day 0	Day 3	Day 7	Day 14	Day 28	Week 12	Week 24	Week 36	Week 48	Week 72	Premature Study D/C (Before Day 28)	Premature Study D/C (After Day 28)
Visit Window			+/-1 day	+/-2 days		+4 days	-7/+14 days						
Post-Acute COVID-19 Assessment							X	X	X	X	X		X
Household Infection and Linkage Report		X				X	X	X				X	X
Self-Collected Anterior Nasal Swab		X	X	X	X	X						X	
Retrieval of Self-Collected Anterior Nasal Swabs			Follow Instructions in MOP									X	
Blood Plasma for SARS-CoV-2 RNA		X										X	
Inflammatory Markers		X				X						X	
Coagulation Markers		X				X						X	
Hematology		X				X						X	
Chemistry		X				X						X	
Pregnancy Testing	X		Whenever Pregnancy Suspected										
Stored Plasma		X				X		X				X	X
Stored Serum		X				X		X				X	X
PK Studies		X[1]				X	X	X				X	X
Antidrug Antibodies		X				X	X	X				X	X

[1] First PK serum sample to be obtained prior to infusion along with remainder of entry labs. A second PK sample to be obtained at the completion of the infusion.

6.3 Instructions for Evaluations

6.3.9 Investigational Agent Administered

Pre-Medication

Pre-medication for infusions is not planned. However, if the participant has a medical history suggesting a potential benefit from pre-medication, the study investigator(s) should determine the appropriate pre-medication.

Any pre-medications given will be documented as a concomitant medication.

Before the Infusion

Vital signs (temperature, heart rate, respiratory rate, blood pressure, and SpO2).

During the Infusion

As the infusion should run over approximately 15 minutes, vital signs (temperature, heart rate, respiratory rate, blood pressure and SpO_2) will be measured at infusion end (± 3 minutes).

After Infusion

Vital signs (temperature, heart rate, respiratory rate, blood pressure, and SpO2) will be measured every 30 minutes (± 5 minutes) for 2 hours post infusion.

Only vital signs that meet AE reporting requirements will be recorded on an eCRF.

6.3.14 Laboratory Evaluations

Hematology

Participants will have blood drawn for complete blood cell count (CBC) with automated differential and platelet count.

At Entry/Day 0, blood should be drawn before study drug administration.

Chemistry

Participants will have blood drawn for liver function tests (ALT, ALP, AST, total bilirubin, direct bilirubin, and total protein), and renal function tests (albumin, BUN, creatinine, potassium, glucose, and sodium).

At Entry/Day 0, blood should be drawn before study drug administration.

Pregnancy Testing

For participants of reproductive potential: Serum or urine β-HCG. (Urine test must have a sensitivity of ≤25 mIU/mL).

Post-screening, pregnancy testing should be done any time pregnancy is suspected per the SOE.

In the event of pregnancy occurring during the study, record pregnancy and pregnancy outcome per section 8.3.

6.3.15 Pharmacokinetics

Serum will be collected and used to measure investigational agent levels.

At Entry/Day 0, the first serum sample should be collected along with the remainder of entry labs before the dose of investigational agent/placebo (up to 10 minutes before the start of infusion). A second PK sample should be obtained at the completion of the infusion (up to 15 minutes after completion of infusion) from an opposite limb and not the IV line/same site as the infusion.

Post-entry, serum should be collected as per the SOE for PK measurements. Date and time of collection should be recorded.

Samples will be analyzed at a laboratory approved by the sponsor and stored at a facility designated by the sponsor. Concentrations of the investigational agent will be assayed using a validated bioanalytical method. Analyses of samples collected from placebo-treated participants are not planned. Samples will be retained for up to 2-years after last patient visit. Remaining samples used for PK may be pooled and used for exploratory metabolism or bioanalytical method experiments as deemed appropriate.

6.3.17 Anti-Drug Antibodies

Serum will be collected to measure anti-drug antibodies (ADAs). At Entry/Day 0, serum should be collected before the dose of investigational agent/placebo.

Post-entry, serum should be collected as per the SOE for ADA measurement. Date and time of collection should be recorded.

Samples will be analyzed at a laboratory approved by the sponsor and stored at a facility designated by the sponsor.

7.0 ADVERSE EVENTS AND STUDY MONITORING

7.1 Definitions of Adverse Events

Adverse Events of Special Interest

The following are AESIs for the agent AZD7442 or placebo for AZD7442:

- ≥ Grade 1 infusion-related reactions within 12 hours of investigational agent/placebo administration (deemed related to study product as determined by the site investigator)
- ≥ Grade 1 allergic/hypersensitivity reactions within 12hrs of investigational agent/placebo administration (deemed related to study product as determined by the site investigator)

7.3 Recording Adverse Events

Post entry, the following non-lab AEs must be recorded on the eCRFs within 72 hours:

- Phase II and III: Grade 1 AEs

8.0 CLINICAL MANAGEMENT ISSUES

8.2 Management of Side Effects

8.2.1 Overdose

An overdose is defined as greater than the protocol indicated dose for either component of AZD7442 (>150 mg). There is no known antidote for AZD7442 overdose. In the event this occurs, the participant should be closely monitored for AE/SAE and laboratory abnormalities, and supportive care provided as indicated. If it is determined that an infusion contains more than the assigned dose, the infusion should be stopped immediately on recognition and the estimated dose received should be recorded.

8.2.2 Infusion-Related Reactions

All participants should be monitored closely, as there is a risk of infusion reaction (including anaphylaxis) with any biological agent.

Symptoms and signs that may occur as part of an infusion reaction include, but are not limited to fever, chills, nausea, headache, bronchospasm, hypotension, angioedema, throat irritation, rash including urticaria, pruritus, myalgia, and dizziness.

The severity of infusion-related reactions will be assessed and reported using the Division of AIDS Table for Grading the Severity of Adult and Pediatric Adverse Events (DAIDS AE Grading Table), corrected Version 2.1, July 2017, which can be found on the DAIDS RSC website at https://rsc.niaid.nih.gov/clinical-research-sites/daids-adverse-event-grading-tables.
The clinical site should have necessary equipment and medications for the management of any infusion reaction, which may include but is not limited to oxygen, IV fluid, epinephrine, acetaminophen and antihistamine.

Investigators should determine the severity of the infusion reaction and manage infusion reactions based on standard of care and their clinical judgment. If an infusion reaction occurs, then supportive care should be provided in accordance with the signs and symptoms.

Dosing can be modified, such as slowing infusion, for mild or moderate reactions (Grade 1 or Grade 2).

8.2.3 Hypersensitivity

Signs and symptoms of infusion-related immediate hypersensitivity reactions may include, but are not limited to anaphylaxis, angioedema, bronchospasm, chills, diarrhea, hypotension, itching, skin rash, shortness of breath, urticaria, tachycardia, and throat irritation or tightness [9].

Participants will be closely monitored for immediate hypersensitivity reactions.

Sites should have appropriately trained medical staff and appropriate medical equipment available when study participants are receiving AZD7442. Participants who experience a systemic hypersensitivity reaction should be treated per the local standard of care.

Dosing can be modified, such as slowing infusion, for mild or moderate reactions (Grade 1 or Grade 2).

8.3 Pregnancy

There are no data regarding the use of AZD7442 in participants who are pregnant, and therefore potential participants who are pregnant are not eligible during screening.

If a participant becomes pregnant during the study (post-entry), study follow up will continue for the duration of the study.

At the end of the pregnancy, outcome and adverse events for participant and infant will be recorded on the outcome eCRF.

8.4 Breastfeeding

Since there are no data regarding the use of AZD7442 in participants who are breastfeeding, participants who are breastfeeding are not eligible for the study.

9.0 CRITERIA FOR DISCONTINUATION

9.1 Permanent and Premature Treatment Discontinuation

A participant will stop investigational agent/placebo if a Grade ≥3 event occurs that is deemed related to the investigational agent/placebo.

10.0 STATISTICAL CONSIDERATIONS

10.2 Outcome Measures

Primary and secondary outcome measures listed below will be addressed in the AZD7442 IV specific appendix to the study’s primary Statistical Analysis Plan.

10.2.3 Secondary Outcome Measures

The following secondary outcome measures will also be assessed:

10.2.3.13 Phase II only: New Grade 2 or higher AE through week 48.

10.2.3.14 Phase III only: New Grade 3 or higher AE through week 48.

11.0 PHARMACOLOGY PLAN

11.1 Pharmacology Objectives

The phase II pharmacology objective is to determine the pharmacokinetics of AZD7442. For phases II and III, the pharmacology objective is to explore relationships between dose and concentration of AZD7442 with virology, symptoms, and oxygenation.

11.2 Pharmacology Study Design Overview

The Schedule of Evaluations shows the collection schedule for Phase II and for Phase III. AZD7442 has a long-elimination in preclinical animal studies, and is expected to be as long as 90 days in humans. The PK sample schedules are based on the long-elimination half-life of AZD7442 and are designed to meet the phase II objective of determination of AZD7442 pharmacokinetics and the phase III objective to explore dose/concentration-response relationships. By design, the sample collection schedules are different, with the phase II schedule being more intense to determine PK behavior, and the phase III schedule sparser to confirm PK behavior and support dose/concentration-response analyses.

11.3 Pharmacology Data Analysis and Modeling

Pharmacokinetic data analysis of phase II data will use conventional and accepted approaches such as non-compartmental analysis or compartmental analysis to determine the PK characteristics of AZD7442 and its components. Population pharmacokinetic approaches (e.g. nonlinear mix effects modeling such as implemented in NONMEM) may also be used. The usual parameters of interest are area under the concentration-time curve (AUC), total body clearance (CL), elimination half-life ($T_{1/2}$), and maximum and minimum concentrations (C_{max}, C_{min}). Exploration of relationships between dose and concentration of AZD7442 components with virology, symptoms, and oxygenation will be approached using conventional and accepted methods for pharmacokinetic/pharmacodynamic (PK/PD) data analyses. Such methods will include the E_{max} or sigmoid E_{max} model or structurally linked PK/PD models (as could be performed within NONMEM) to explore exposure-response relationships. Exposure-response relationships will be performed in conjunction with the protocol statisticians.

16.0 REFERENCES

1. Doherty M, Robertson MJ. Some early trends in immunology. Trends Immunol 2004;25:623-31.
2. Casadevall A, Dadachova E, Pirofski LA. Passive antibody therapy for infectious diseases. Nat Rev Microbiol 2004;2:695-703.
3. Saylor C, Dadachova E, Casadevall A. Monoclonal antibody-based therapies for microbial diseases. Vaccine 2009;27:G38-G46.
4. Casadevall A. The case for pathogen-specific therapy. Expert Opin Pharmacother 2009;10:1699-703.
5. IMpact-RSV Study Group. Palivizumab, a humanized respiratory syncytial virus monoclonal antibody, reduces hospitalization from respiratory syncytial virus infection in high-risk infants. Pediatrics 1998;102(3 Pt 1):531-7.
6. Fan P, Chi X, Liu L, et al. Potent neutralizing monoclonal antibodies against Ebola virus isolated from vaccinated donors. MAbs 2020;12:1742457.
7. Everds NE, Tarrant JM. Unexpected hematologic effects of biotherapeutics in nonclinical species and in humans. Toxicol Pathol 2013;41:280-302.
8. Investigator’s Brochure, AstraZeneca AZD7442 3.0; 08 December 2020.
9. Dinnon KH, 3rd, Leist SR, Schafer A, et al. A mouse-adapted model of SARS-CoV-2 to test COVID-19 countermeasures. Nature 2020 586:560-6.
10. Chandrashekar A, Liu J, Martinot AJ, et al. SARS-CoV-2 infection protects against rechallenge in rhesus macaques. Science 2020;369:812-17.
11. Yu J, Tostanoski LH, Peter L, et al. DNA vaccine protection against SARS-CoV-2 in rhesus macaques. Science 2020;369:806-11.
12. Zuidema J, Pieters FAJM, Duchateau GSMJE. Release and absorption rate aspects of intramuscularly injected pharmaceuticals. Int J Pharma 1988;47:1-12.
13. Scialli AR, Bailey G, Beyer BK, et al. Potential seminal transport of pharmaceuticals to the conceptus. Reprod Toxicol 2015;58:213-21.

APPENDIX VII: SAMPLE INFORMED CONSENT FOR STUDY DRUG AZD7442 ADMINISTERED VIA INTRAVENOUS INFUSION

One of the study drugs that you might be assigned to in this study is AZD7442 or the placebo for AZD7442.

AZD7442 is a type of drug called a monoclonal antibody. Many antibodies are naturally made by your body and help fight diseases. AZD7442 is made in a laboratory. It is a combination of two monoclonal antibodies, meaning many copies each of two antibodies designed to prevent SARS-CoV-2, the virus that causes COVID-19, from entering cells.

Your assignment is random, like the flip of a coin. You will be told about all the study drugs you may be assigned to in this study. If only one study drug is available, you will have an equal chance of receiving the study drug or placebo. If two study drugs are available, you will have a 2:1 chance of receiving a study drug or placebo. If three study drugs are available, you will have a 3:1 chance of receiving a study drug or placebo, and so forth. You will not be able to choose your group (study drug), and neither you, your study doctor, nor the study staff at your site will know whether you are receiving the study drug or placebo.

The United States Food and Drug Administration (FDA) has not approved AZD7442 for general use by the public. However, we have told the FDA about this study and they have given us permission to conduct this study.

ARE THERE ANY ADDITIONAL STUDY PROCEDURES IF I RECEIVE AZD7442 OR PLACEBO?

Screening Visit

- At your screening visit, if you can become pregnant, you will be asked to give blood (1 teaspoon) or a urine sample for a pregnancy test. You cannot receive AZD7442 or placebo if you are pregnant.

Entry Visit

- You will have blood drawn. This blood may be used for the following tests:
  - routine safety tests (liver and kidney tests and blood counts)
  - levels of the drug in your blood (you will have blood drawn before you receive the study drug and again after)
  - levels of antibodies to the drug (your body's immune response to the drug)
- You will have the infusion of each component of AZD7442 or placebo. The infusion will be given through a small plastic tube that will be placed into a vein in your arm. This is called an intravenous (IV) infusion. The infusion itself will take approximately 15 minutes. You will then be monitored for another 2 hours.

Study Visits

After the Entry visit, your study visits and evaluations will be different depending on whether you are in the first part of the study or the second part of the study.

IF YOU ARE IN THE FIRST PART OF THE STUDY:

Study Visits on Days 3, 7, 14, and 28

- You will have blood drawn. This blood will be used for the following tests:
  - Routine safety tests (liver and kidney tests and blood counts) (days 3, 14, and 28)
  - Levels of the drug and/or levels of antibodies to the drug (your body's immune response to the drug) (days 3, 7, 14 and 28)

Study Visits on Week 12 and 24

- You will have blood drawn. This blood will be used for the following tests:
  - levels of the drug and levels of antibodies to the drug (your body's immune response to the drug)
- You will be asked whether you have had any new symptoms or clinical events since your last visit.

Study Visits on Weeks 36, 48, and 72
- You will be contacted by phone by the study team to assess whether you have had any new symptoms or clinical events since your last visit
- You will answer questions about any potential COVID-19 related symptoms or conditions you have experienced

IF YOU ARE IN THE SECOND PART OF THE STUDY:

Study Visit on Day 28
- You will have blood drawn. This blood will be used for the following tests:
  - routine safety tests (liver and kidney tests and blood counts)
  - levels of the drug
  - levels of antibodies to the drug (your body's immune response to the drug)

Study Visit on Weeks 12 and 24
- You will have blood drawn. This blood will be used for the following tests:
  - levels of the drug
  - levels of antibodies to the drug (your body's immune response to the drug)
- You will be asked whether you have had any new symptoms or clinical events since your last visit.

Study Visits on Weeks 36, 48, and 72
- You will be contacted by phone by the study team to assess whether you have had any new symptoms or clinical events since your last visit
- You will answer questions about any potential COVID-19 related symptoms or conditions you have experienced

HOW LONG WILL I BE IN THIS STUDY?

If you are assigned to AZD7442 or placebo for AZD7442, you will be in this study for 72 weeks.

WHAT ARE THE RISKS OF AZD7442?

There is a risk of serious and/or life-threatening side effects when non-study medications are taken with the study drugs. For your safety, you must tell the study doctor or nurse about all medications you are taking before you start the study.

Another risk is that the study drug used in this study may have side effects, some of which are listed below. Additionally, the study drug tested in the study may have unknown side effects in persons with SARS-CoV-2 infection. In a research study, all of the risks or side effects may not be known before you start the study. You need to tell your doctor or a member of the study team immediately if you experience any side effects.

Please note that these lists do not include all the side effects seen with this study drug. These lists include the more serious or common side effects with a known or possible relationship to the study drug. If you have questions concerning the additional side effects, please ask the medical staff at your site.

Risks Associated with AZD7442

There is limited safety data on AZD7442 since it has not been given to a lot of people. As of December 8, 2020, there have been no serious unexpected effects reported by >100 healthy people taking AZD7442 or placebo to date. Most effects after taking AZD7442 or placebo have been mild or moderate and have either all gone away or are getting better. This study is likely to be the first study where this study drug is given to people with COVID-19 disease.

Administration of AZD7442 may result in allergic reactions. Signs and symptoms of these reactions include:

- chills
- skin rash
- itching
- hives
- swelling of the face or other soft tissues
- low blood pressure
- rapid heart rate
- throat irritation or tightness
- tightening of the muscles that line the airways
- shortness of breath
- loose stools

Administration of AZD7442 may induce release of chemicals called cytokines in the body. These chemicals may induce allergic reactions listed above as well as:

- fever
- muscle aches
- nausea
- vomiting
- headache
- dizziness

Some of these reactions may be serious or life-threatening including:

- skin rash
- swelling of the face or other soft tissues
- low blood pressure
- rapid heart rate
- throat irritation or tightness
- tightening of the muscles that line the airways
- shortness of breath

You will be monitored closely during administration of study drug. Medical personnel, equipment, and medication will be available to manage these reactions appropriately if they occur.

Administration of study drug may also cause the following risks and discomforts:

- development of proteins (antibodies) against AZD7442. This may cause your body to get rid of AZD7442 more quickly or change the effect of AZD7442 on the body. Your blood will be tested to find out whether your body made antibodies to AZD7442. The anticipated risk of this is low because AZD7442 is a fully human antibody.

Therefore, it is less likely to be seen as "foreign" by your body's immune system and your body is less likely to form antibodies against AZD7442.

- mixture of antibody and other chemicals in the body that may be deposited in tissues such as blood vessels and kidneys.
- unexpected increase in virus reproduction in your body. Although this has been observed with some viruses, this has not been observed with COVID-19 or with the use of serum-containing antibodies given to people with COVID-19. This risk of increased viral growth is perhaps greater when there are lower levels of antibodies in the blood in the presence of virus. To avoid this, AZD7442 will be given at a dose that is felt to be high enough to keep this from occurring.

Effect on Future Vaccination

The US Centers for Disease Control and Prevention (CDC) currently recommends that people wait at least 90 days after receiving antibody treatment before receiving a COVID-19 vaccine, because some antibodies remain in the body for about 90 days, and there is a chance that these antibodies could interfere with how your body responds to the vaccine during those 90 days. Some of the antibodies in this study including AZD7442 are designed to remain in the body for longer than 90 days. Although there is no further guidance available, there is a chance that these longer-lasting monoclonal antibodies could interfere with how your body responds to the vaccine even if you wait at least 90 days for the vaccine.

ARE THERE RISKS RELATED TO PREGNANCY AND BREASTFEEDING?

Pregnancy

Since there are no data regarding the use of this study drug in people who are pregnant, you are not eligible to receive this study drug if you are pregnant.

The study drug may involve risks to you (or to the embryo or fetus, if you or your partner become pregnant), which are currently unforeseen.

If you are engaging in sexual activity that could lead to pregnancy, you must agree to use effective contraception for 24 weeks after the study drugs are administered. Effective contraception includes oral contraceptives, implanted contraceptives, and intrauterine devices

If you are engaging in sexual activity that may lead to pregnancy in your partner, you must agree to either remain abstinent or use male contraceptives. You are also advised to inform your non-pregnant sexual partners that can become pregnant to use effective contraceptives for 24 weeks after the study drugs are administered to you.

If you have a pregnant partner you should use condoms during vaginal intercourse through 24 weeks after the study drugs administered.

If applicable, you should refrain from sperm donation for 24 weeks after study drug administration.

If at any point during the study you think you may be pregnant, you should let the staff at your site know so that a pregnancy test can be done.

Let your doctor know immediately if you become pregnant. If you become pregnant while on the study, you will be asked to continue to have study visits and the study staff would like to obtain information from you about the outcome of the pregnancy (even if it is after your participation in the study ends).

Breastfeeding

It is not known if this study drug is safe to use in people who are breastfeeding. You are not eligible to receive this

study drug if you are breastfeeding.

APPENDIX VIII: INVESTIGATIONAL AGENT AZD7442 INTRAMUSCULAR ADMINISTRATION

Information/evaluations noted in this agent-specific appendix are IN ADDITION to those presented in the master protocol. Section numbering aligns with the master protocol.

SCHEMA

DURATION: 72 weeks

2.0INTRODUCTION

2.2Rationale

Monoclonal Antibodies (mAbs)
Sera obtained from persons or animals who recovered from a particular infection has shown prophylactic and therapeutic potential for a variety of infections, and Emil von Behring won the Nobel Prize in 1893 for his work on use of immune serum from the blood of infected animals to provide immunity to diphtheria [1]. Currently, hyperimmune human sera immunoglobulin is still used to treat many viral infections including cytomegalovirus (CMV), respiratory syncytial virus (RSV), hepatitis A virus (HAV), hepatitis B virus (HBV), and rabies [2].

Unfortunately, heterologous sera were associated with a variety of complications including serum sickness and hypersensitivity, which significantly limited its usefulness clinically [3]. Given the long history of use of antibodies for infectious diseases, monoclonal antibodies were developed (mAbs). Improved purification techniques and the ability to engineer humanized mAbs allowed for the development of broadly reactive and potent mAbs, which helped reduce some of the issues that hampered the utility of heterologous sera [3, 4]. In fact, current technology allows mAbs to be produced requiring only tissue culture or microbial expression systems, thus the potential toxicity of humanized mAbs is comparable to antibiotics [2, 4].

Engineered humanized and human mAbs have shown considerable efficacy for viral infections. The first was palivizumab in 1998, which is used for RSV [5]. Monoclonal antibodies have also been quickly developed for emerging infections such as Ebola [6]. As a part of the massive scientific effort to stop COVID-19, mAbs have been developed for treatment of COVID-19. These agents now need to be evaluated in rigorous randomized clinical trials.

The limitations of mAbs continue to be cost and that these antibodies are perishable, require refrigeration, and must be administered parenterally [4]; however, their use may still be useful in the outpatient setting, as one dose often stays in the therapeutic range for months [5], potentially allowing an entire treatment course with a single administration.

A number of viral infectious diseases have been successfully treated with mAbs, including RSV and HIV. Some of these mAbs were derived from persons who were infected with these viruses and mounted neutralizing humoral responses.

An investigational agent to be evaluated in this trial will be the mAb AZD7442 delivered intramuscularly and made by AstraZeneca Pharmaceuticals LP for the treatment of early, symptomatic SARS-CoV-2 infection.

Investigational Agent

Background

AZD7442 is a combination of two human mAbs, AZD8895 and AZD1061. Both were cloned from B-cells isolated from peripheral blood mononuclear cells (PBMCs) obtained from COVID-19 convalescent patients. These mAbs bind to unique, non-overlapping epitopes at the human angiotensin-converting enzyme 2 (hACE2) interface of the receptor binding domain (RBD of the Spike (S) protein of SARS-CoV-2, preventing viral entry into human cells and its subsequent viral replication. The two antibodies in the combination contain modifications in their FC regions that extends their anticipated half-life up to 70-130 days [3-6] and reduces the risk of antibody disease enhancement (ADE), by limiting binding to cellular Fc gamma receptor [7]. The combination of two mAbs with differing binding sites on the RBD is intended to reduce the probability of viral mutations that would confer antibody resistance, and to provide synergy in their virus neutralizing activity.

AZD7442 is expected to result in a clinically important decrease of viral replication, mitigating the severity of COVID-19 in persons with the infection in whom ongoing viral replication is the primary driver of pathophysiology. The potential reduction in viral replication may also decrease a treated person's extent and duration of viral shedding and transmission, thus potentially positively impacting public health.

Non-Clinical Studies: Pharmacokinetics (PK)
Nonclinical studies of AZD7442 have been performed in mice and non-human primates (NHPs). In human Fcn transgenic Tg32 mice, peak serum concentrations at 28 days post intravenous administration of AZD7442 components remained well above the EC50s and EC99s determined in cellular infection assays [8]. The toxicokinetic profile of AZD7442 (AZD8895 and AZD1061) following IV or IM administration has been evaluated in cynomolgus monkeys as part of a GLP toxicology study. In this GLP toxicology study for AZD7442 high exposures were achieved and were very consistent across animals and between males and females for both AZD8895 and AZD1061, for both the 300 mg/kg IV dose and the 75 mg/kg IM dose of each antibody [8]. Based on the data available over the first 4 weeks after dosing, the predicted safety exposure margin for the clinical 600 mg IM AZD7442 dose against the IM NOAEL of 150 mg/kg AZD7442 dose is 21-fold and 28-fold for $AUC_{(0\text{-}4\ weeks)}$ and C_{max}, respectively [8]. Neither component of AZD7442 cross-reacts with human tissue targets, and neither was found to bind to any human reproductive tissues in pre-clinical testing, including the placenta [8].

Non-Clinical Studies: Antiviral Effects
Murine models of SARS-CoV-2 have been performed to study the prophylactic and post-exposure antiviral activity of the AZD7442. In these models, the parenteral formulations of AZD7442 components were studied: COV2-2196 and COV2-2130, the respective parental antibodies of AZD8895 and AZD1061. COV2-2196 and COV2-2130 lack the Fc region modifications but are expected to retain antiviral activity. In a mouse-adapted-SARS-CoV-2 model, BALB/c mice were inoculated via intranasal route with 10^5 fluorescent focus units (FFU) of MA-SARS-CoV-2 and COV2-2196 and COV2-2130 (1:1 cocktail) administered intravenously 12 hours after. Viral burden in the lungs was measured 2 days post infection (dpi) after viral challenge using RT-qPCR or plaque assay. Mice were monitored daily for body weight change. Administration of the mAbs 12 hours post infection in Ad5-hACE2 transduced mice resulted in neutralization of infectious virus in the lungs. The mAbs were also evaluated for in vivo efficacy in an immuno-competent model using a mouse-adapted-SARS-CoV-2 virus. In this model there was significant viral replication in the lungs but little or no clinical disease [9].

The parental mAb formulations were also assessed in a non-human primate (NHP) model [10, 11]. Rhesus macaques received one 50 mg/kg dose of COV2-2196 or isotype control antibody intravenously 3 days prior to intranasal and intratracheal challenge with a total dose of 10,000 PFU SARS-CoV-2. Virus replication was quantitated by RT-qPCR for viral sgmRNA, which measures replicative viral RNA intermediates. Animals that received isotype control antibody showed a median peak of 7.53 log10 sgmRNA copies/swab in nasal swab and a median peak of 4.97 log10 sgmRNA copies/mL in bronchoalveolar lavage. In contrast, viral sgmRNA was not detected in either nasal or bronchoalveolar

lavage samples from animals that received COV2-2196. A PK analysis revealed similar concentrations of circulating human mAbs in animals from both groups.

Human Clinical Studies
The first in-human clinical studies of AZD7442 began enrolling in August 2020. (NCT04507256). Both IV (300 mg, 1000 mg, and 3000 mg), sequentially and co-administered, and IM (300 mg) administration have been studied in this phase I, single-dose, dose-escalating trial among healthy adults. As of early December, **>100** persons in this study have received the product at doses up to 3000 mg IV, and no safety concerns have emerged. **Specifically, there were no deaths, SAEs or discontinuation of study product due to AEs and no laboratory events of concern. Pharmacokinetic data from this phase I study also demonstrate the persistence of neutralizing antibody levels at 90 days post-infusion in all participants, with dose-dependent concentrations observed. As described below, pharmacokinetic data have also demonstrated relative levels achieved with IV and IM dosing.**

The proposed adaptive Phase II/III trial is likely to be the first administration in persons with COVID-19 disease, although pre-exposure and post-exposure prophylaxis studies have started. **These include clinical studies PROVENT (pre-exposure), STORM CHASER (post-exposure) and TACKLE (treatment with 600 mg IM).** As of December 8, 2020, there was a single SAE in a pre-exposure prophylaxis participant who fainted following product administration (IM) and required evaluation at the hospital.

Choice of Study Dosing
Human efficacious doses for AZD7442 were evaluated using in vitro potency data (virus neutralizing activity of AZD7442 against SARS-CoV-2) and PK data. In addition, a viral-dynamic model was developed, which allowed for understanding of the pharmacodynamic effects of AZD7442 to inhibit a SARS-CoV-2 infection and the resulting immune response. The viral-dynamic model indicates that assuming a partition ratio ranging between 0.1-1.0% for lung epithelial lining fluid-to-serum and assuming potency with an IC_{80} (inhibiting SARS-CoV-2 by 80%) of 40 ng/mL, the estimated effective concentration may be as low as 4µg/mL in serum. The dosing in Phase I studies therefore targeted 20-40 µg/ml to assure sufficient levels in patients with an active SARS-CoV-2 infection; 600 mg AZD7442 IM administration before the time of peak viral load (on average ~7 days after day of infection) is expected to result in reduction of the peak viral load and earlier eradication of the viral load. Administration of 600 mg IM AZD7442 after the peak viral load has been reached is still expected to result in earlier viral load eradication compared to when drug is not present based on this same viral-dynamic model.

Figure 2.2-1 shows the preliminary serum AZD7442 concentration through 30 days post dose for the different single doses tested as well as the predicted concentration time course for a single 600 IM dose, estimated by multiplying the concentrations for the 300 mg IM dose by a factor of 2. The median time of maximum drug levels still needs to be defined when more data are available but initial data suggests that the T_{max} can be as late as 30 days post dose when administered in the ventrogluteal muscle. However, the crucial therapeutic window is likely to be within three days of administration, which means that the fasted possible attainment of target serum concentrations (4-40 µg/mL) should be prioritized over the C_{max} for therapeutic uses. Because the 600 mg dose will result in serum concentrations in the target range at an earlier time point compared to the 300 mg IM dose, 600 mg IM was selected, also given the maximum volume that can be administered intramuscularly with AZD7442 supplied at 100 mg/mL, as two separate site injections of 3 mL each. As both 300mg IV and 600mg IM are being tested together in separate appendices of this same ACTIV-2 protocol, the selection of these two separate doses and routes of administration will allow direct comparison of the PK between these groups.

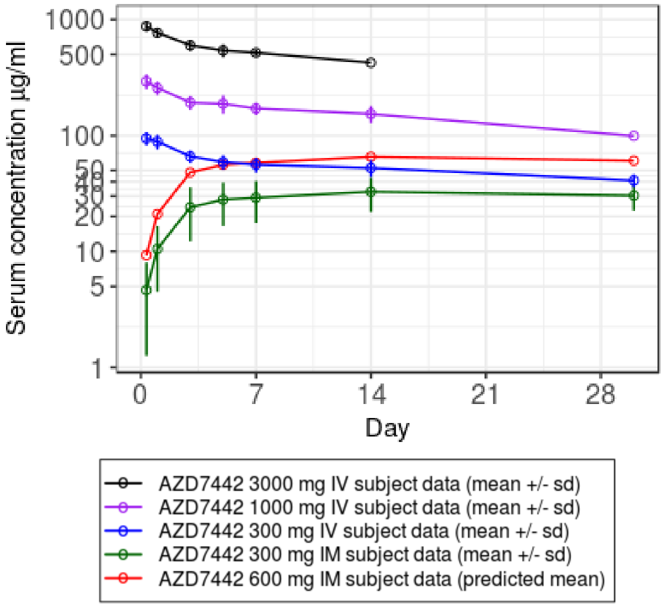

Figure 2.2-1: Observed Mean (SD) Serum AZD7442 concentrations over the first 30 days in Adult Healthy Volunteers in the Phase 1 Study

Rationale for Administration Site

The Phase I study tested 300 mg administered in two gluteal injections at a total injection volume of 1.5 mL per side. The dose selected for this trial is 600 mg IM, which will be delivered as two thigh injections of 300 mg each with a total volume of 3.0 mL per side (one injection in each thigh). Given the argument above that time to estimated effective concentration range is the most important parameter in treating COVID-19, we have chosen to administer IM AZD7442 to the vastus lateralis (lateral thigh). Compared to either dorsal or ventral gluteal sites, the thigh or deltoid have increased rate and decreased variability of absorption. The planned volume per injection exceeds that administered to the deltoid but falls within the accepted standard of care for the thigh. Gluteus medius absorption is slowest and most variable, with variability related to BMI, sex, and age, with slowest and impaired absorption in those with higher gluteal fat (Figure 2.2-2, Panel 2A), especially when administration is into the adipose layer and does not reach muscle (Figure 2.2-2 Panel 2B) [12].

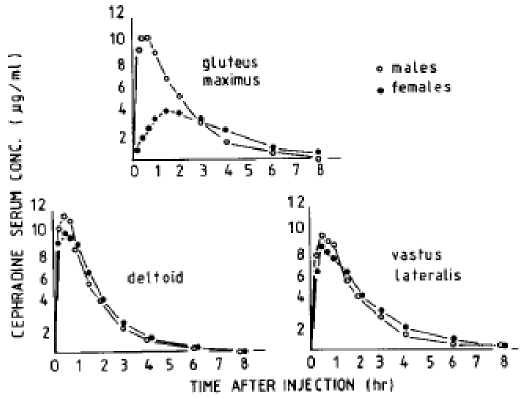

Fig. 2. Cefradine concentrations in serum after i.m. injections of 475 mg in healthy male and female subjects according to site.

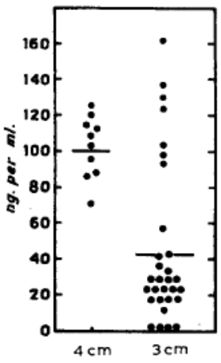

Figure 2.2-2: Panel 2A shows more consistent and rapid absorption of cefradine in women on par with men in the vastus lateralis, likely due to differential distribution of gluteal adipose tissue. This is further exemplified by Panel 2B, which shows impaired absorption of diazepam when administered to the gluteus with a shorter needle, which frequently results in administration to the adipose layer rather than a true intramuscular administration [12].

4.0SELECTION AND ENROLLMENT OF PARTICIPANTS

Participants must meet inclusion and exclusion criteria from the master protocol, as well as the appropriate inclusion and exclusion criteria for the investigational agent included below.

4.1General Eligibility Criteria

4.1.1Inclusion Criteria

All criteria within the master informed consent are applicable with the additional criteria as added below:

4.1.1.9 For participants who are of reproductive potential, negative serum or urine pregnancy test at within 48 hours prior to study entry by any clinic or laboratory that has a CLIA certification or its equivalent, or by a point of care (POC)/CLIA-waived test.

Reproductive potential is defined as:

- participants who have reached menarche
- participants who have not been post-menopausal for at least 12 consecutive months with follicle-stimulating hormone (FSH) ≥40 IU/mL or 24 consecutive months if an FSH is not available
- participants who have not undergone surgical sterilization (e.g., hysterectomy, bilateral oophorectomy, bilateral tubal ligation, or bilateral salpingectomy)
- participants with no other clinical conditions (such as anorexia nervosa) that could induce amenorrhea
- participants not taking medications such as oral contraceptives, hormones, gonadotropin-releasing hormone, anti-estrogens, selective estrogen receptor

modulators (SERMs) or chemotherapy that could induce amenorrhea

- For individuals with permanent infertility due to an alternate medical cause (e.g., Mullerian agenesis, androgen insensitivity), investigator discretion should be applied to determining study entry.

4.1.1.10 If participating in sexual activity that could lead to pregnancy, participants who are of reproductive potential must agree to use highly-effective contraception for 24 weeks after investigational agent is administered. This would include oral contraceptives, implanted contraceptives, and intrauterine devices.

NOTE: Participants not of reproductive potential are eligible without requiring the use of a contraceptive method. Participant-reported history is acceptable documentation of surgical sterilization and menopause, including vasectomy in a sole partner.

4.1.1.11 Participants that engage in sexual activity that may lead to pregnancy in their partner must agree to either remain abstinent or use male contraceptives. They are also strongly advised to inform their non-pregnant sexual partners of reproductive potential to use effective contraceptives for 24 weeks after investigational agent is administered.

Participants with pregnant partners should use condoms during vaginal intercourse through 24 weeks after investigational agent administration.

Participants should refrain from sperm donation for 24 weeks after investigational agent administration.

4.1.2 Exclusion Criteria

4.1.2.11 Currently pregnant or breastfeeding

4.1.2.12 Inflammatory skin conditions that compromise the safety of IM injections, or other overlying skin conditions or tattoos that would preclude the assessment of injection site reactions, per the discretion of the investigator

4.1.2.13 History of coagulopathy which, in the opinion of the investigator, would preclude IM injection, or use of oral or injectable anticoagulants (see prohibited medications, section 5.4).

5.0INVESTIGATIONAL AGENTS

5.1 Regimen, Administration, and Duration

5.1.1 Regimen and Duration

Participants will be randomized to receive one of the following regimens:

Investigational Agent: AZD7442, 600 mg, to be administered intramuscularly (IM), as two separate injections (AZD8895, 300 mg, and AZD1061, 300 mg), for one dose at study Entry/Day 0.

OR

Placebo for AZD7442: 0.9% Sodium Chloride Injection, USP, to be administered IM, as two separate injections, for one dose at study Entry/Day 0.

5.1.2 Administration

AZD8895/Placebo and AZD1061/Placebo to be administered IM as two separate injections, **one following the other in this order**, with a 22-25 gauge, 1-1.5 inch (25-38 mm) length needle each. The injections are to be administered using standard IM injection technique. Injections will be given in the lateral thigh (vastus lateralis, VL) site, one injection in each thigh. **No pause between the two injections is required. The time and site of each injection will be recorded on the eCRF.**

5.2 Formulation, Storage, and Preparation

5.2.1Formulation and Storage

AZD7442 consists of two independent drug substances, AZD8895 and AZD1061, which are formulated separately. Both AZD8895 and AZD1061 are supplied as a 100 mg/mL aqueous solution with 150 mg (nominal) of active investigational product in 10R glass vials with a volume of 1.5 mL. The aqueous solutions are colorless to slightly yellow, clear to opalescent.

AZD8895 and AZD1061 vials must be stored between 2°C to 8°C (refrigerated storage) until use. AZD7442 is described in further detail in AZD7442 Investigator's Brochure.

Placebo for AZD7442 will be 0.9% Sodium Chloride Injection, USP. The product must be locally sourced and stored according to the manufacturer's recommendation.

5.2.2 Preparation

Pharmacists must follow appropriate aseptic technique and consider sterile preparation procedures/guidance as outlined in USP General Chapter <797> Pharmaceutical Compounding – Sterile Preparations. Pharmacists must also follow the requirements of their country, institution, and pharmacy regulatory authority regarding these procedures. The investigational agent and placebo should be prepared in a sterile environment, utilizing a biosafety cabinet/isolator. If a biosafety cabinet or isolator is not available, a laminar flow hood may be used. Local regulations and site institutional policies and procedures for use of personal protective equipment, such as gloves, gowns, face masks and safety glasses, must be followed.

Any unused portion of investigational agent must not be used for another participant. Any empty vials, unused portion of entered vials, or unused solution which contains investigational agent should be discarded in a biohazard containment bag and incinerated or autoclaved in accordance with institutional or pharmacy policy.

5.2.2.1 AZD7442

1. Remove two (2) vials of AZD8895 and two (2) vials of AZD1061 from the refrigerator. Equilibrate the vials to room temperature prior to use.
2. Withdraw a total of 3 mL of AZD8895 from the vials obtained in Step 1, using an appropriately sized latex-free disposable syringe made of polycarbonate or polypropylene. When the stopper of the vial is punctured to start preparation, record this time as the investigational agent preparation time. Assign a 4 hour

beyond use date and time from the preparation time if stored at room temperature or a 24 hour beyond use date and time from the preparation time if stored at refrigerated conditions.

3. Using a new appropriately sized latex-free disposable syringe made of polycarbonate or polypropylene, withdraw a total of 3 mL of AZD1061 from the vials obtained in Step 1. Assign the same beyond use time given in Step 2.
4. Apply an overlay to each syringe to ensure blinding is maintained.

5.2.2.2 Placebo for AZD7442

1. Remove 0.9% Sodium Chloride Injection, USP from storage.
2. Withdraw a total of 3 mL of 0.9% Sodium Chloride Injection, USP, using an appropriately sized latex-free disposable syringe made of polycarbonate or polypropylene. When the stopper of the container is punctured to start preparation, record this time as the placebo preparation time. Assign a 4 hour beyond use date and time from the preparation time if stored at room temperature or a 24 hour beyond use date and time from the preparation time if stored at refrigerated conditions.
3. Using a new appropriately sized latex-free disposable syringe made of polycarbonate or polypropylene, withdraw a total of 3 mL of 0.9% Sodium Chloride Injection, USP. Assign the same beyond use time given in Step 2.
4. Apply an overlay to each syringe to ensure blinding is maintained.

5.2.3 Labeling of Investigational Agent and Placebo

Label each prepared IM syringe with the following information:

a. Participant identifier(s)
b. Protocol number: ACTIV-2/A5401
c. Investigational agent name:
    i. AZD8895 300 mg or placebo
    ii. AZD1061 300 mg or placebo
d. Describe sequential order of administration (Administer AZD8895/placebo, first, followed by AZD1061/placebo)
e. Total volume: 3 mL
f. Route: IM
g. Preparation date and time
h. Beyond use date and time: 4 hours after preparation if stored at room temperature conditions or 24 hours after preparation if stored at refrigerated conditions
i. Any additional information required by jurisdiction

5.3 Supply, Distribution, and Accountability

5.3.1 Supply/Distribution

AZD8895 and AZD1061 will be manufactured by Catalent for AstraZeneca and will be available through the NIAID Clinical Research Products Management Center (CRPMC). The site pharmacist will receive ordering instructions for AZD8895 and AZD1061 vials from the NIAID CRPMC.

0.9% Sodium Chloride Injection, USP, and any other ancillary supplies will be locally sourced by the site.

5.3.2 Accountability

The site pharmacist is required to maintain complete records of all investigational agents received from the NIAID CRPMC and subsequently dispensed. At US CRSs, all unused investigational agents must be returned to the NIAID CRPMC (or as otherwise directed by the sponsor) after the study is completed or terminated. At non-US CRSs, the site pharmacist must follow the instructions provided by the CRPMC for the destruction of unused investigational agents.

5.4 Concomitant Medications

Due to the IM route of administration, persons receiving therapeutic anticoagulation including warfarin, low-molecular-weight heparins, and Direct Oral Anti-Coagulants are excluded.

Any pre-medications given will be documented as a concomitant medication. There are no known or expected drug-drug interactions with the investigational agent, and there are no additional prohibited medications except as outlined in section 5.4 of the parent protocol.

6.0CLINICAL AND LABORATORY EVALUATIONS

6.1 Schedule of Evaluations. The schedules of evaluations provided below include all the evaluations in the master protocol and additional evaluations for this investigational agent.

Table 6.1-1: Schedule of Evaluations Phase II

Phase II Evaluation	Screening	Study Entry/Day 0	Day 1*	Day 3	Day 7	Day 14	Day 28	Week 12	Week 24	Week 36	Week 48	Week 72	Premature Study D/C (Before Day 28 Visit)	Premature Study D/C (After Day 28 Visit)
Visit Window				+/-1 day	+/-2 days		+4 days	-7/+14 days						
Documentation of SARS-CoV-2 Infection	X													
COVID-19 Symptom Screen	X	X												
Medical/Medication History	X	X												
Smoking Status		X												
Clinical Assessments	X	X		X	X	X	X	X	X	X	X	X	X	X
Collect/Update Secondary Contacts		X		X	X	X	X	X						
Vital Status Check		If Participant Cannot be Reached per section 6.3.8												
Investigational Agent Administered		X												
Study Kit Dispensed		X												
Participant-Completed Study Diary		Every Day through Day 28												

Phase II Evaluation	Screening	Study Entry/Day 0	Day 1*	Day 3	Day 7	Day 14	Day 28	Week 12	Week 24	Week 36	Week 48	Week 72	Premature Study D/C (Before Day 28 Visit)	Premature Study D/C (After Day 28 Visit)
Visit Window				+/-1 day	+/-2 days		+4 days	-7/+14 days						
Study Diary Reminder			Days 1- 28											
Staff Review of Study Diary		X		X	X	X	X						X	
Retrieval of Study Diary							X						X	
Post-Acute COVID-19 Assessment								X	X	X	X	X		X
Household Infection and Linkage Report		X					X	X	X				X	X
Self-Collected Anterior Nasal Swab		X	Every Day through Day 14				X						X	
Retrieval of Self-Collected Anterior Nasal Swabs			Follow Instructions in MOP										X	
Staff-Collected NP Swab		X		X	X	X	X						X	
Blood Plasma for SARS-CoV-2 RNA		X			X								X	
Inflammatory Markers		X			X		X		X					
Coagulation Markers		X			X		X		X					
Hematology		X		X		X	X						X	
Chemistry		X		X		X	X						X	

Phase II Evaluation	Screening	Study Entry/Day 0	Day 1*	Day 3	Day 7	Day 14	Day 28	Week 12	Week 24	Week 36	Week 48	Week 72	Premature Study D/C (Before Day 28 Visit)	Premature Study D/C (After Day 28 Visit)
Visit Window				+/-1 day	+/-2 days		+4 days	-7/+14 days						
Pregnancy Testing	X		Whenever pregnancy suspected											
PK Studies		X[1]	X**	X	X	X	X	X	X				X	X
Anti-Drug Antibodies		X				X	X	X	X				X	X
Stored Plasma		X			X		X		X				X	X
Stored Serum		X			X		X		X				X	X
Stored PBMCs (Selected Sites)		X			X		X		X				X	

**For approximately 40 participants at selected sites (see MOP and additional site-specific information)

[1] First PK serum sample to be obtained prior to investigational agent/placebo administration along with other entry labs. A second PK sample to be obtained 1 hour after IM administration.

6.3 Instructions for Evaluations

6.3.9 Investigational Agent Administered

Pre-Medication
Pre-medication for IM administration is not planned. However, if the participant has a medical history suggesting a potential benefit from pre-medication, the study investigator(s) should determine the appropriate pre-medication.

Any pre-medications given will be documented as a concomitant medication.

Before the IM Administration
Vital signs (temperature, heart rate, respiratory rate, blood pressure, and SpO2).

After IM Administration
Vital signs (temperature, heart rate, respiratory rate, blood pressure, and SpO2) will be measured every 30 minutes (± 5 minutes) for 2 hours post-administration.

Only vital signs that meet AE reporting requirements will be recorded on an eCRF.

6.3.14 Laboratory Evaluations

Hematology
Participants will have blood drawn for complete blood cell count (CBC) with automated differential and platelet count.

At Entry/Day 0, blood should be drawn before study drug administration.

Chemistry
Participants will have blood drawn for liver function tests (ALT, ALP, AST, total bilirubin, direct bilirubin, and total protein), and renal function tests (albumin, BUN, creatinine, potassium, glucose, and sodium).

At Entry/Day 0, blood should be drawn before study drug administration.

Pregnancy Testing
For participants of reproductive potential: Serum or urine β-HCG. (Urine test must have a sensitivity of ≤25 mIU/mL).

Post-screening, pregnancy testing should be done any time pregnancy is suspected.

In the event of pregnancy occurring during the study, record pregnancy and pregnancy outcome per section 8.3.

6.3.15 Pharmacokinetics

Serum will be collected and used to measure investigational agent levels.

At Entry/Day 0, the first serum sample should be collected along with the remainder of entry labs before the dose of investigational agent/placebo (up to 10 minutes before the start of administration). A second PK sample should be obtained one hour (± 10 minutes) after

administration of the IM injection.

Post-entry, serum should be collected for PK as per the SOE. Date and time of collection should be recorded.

Day 1 PK (Selected Sites): Approximately 40 Phase II participants at selected US sites will have a sample taken for PK at an additional Day 1 visit. The Day 1 PK is the only procedure performed at that visit for those selected participants; other participants do not have a Day 1 visit. The Day 1 PK sample should be collected 18-30 hours after administration of investigational agent/placebo. See MOPS and additional site-specific information for selection of participants for this additional Day 1 PK sample collection.

Samples will be analyzed at a laboratory approved by the sponsor and stored at a facility designated by the sponsor. Concentrations of the investigational agent will be assayed using a validated bioanalytical method. Analyses of samples collected from placebo-treated participants are not planned. Samples will be retained for up to 2 years after last patient visit. Remaining samples used for PK may be pooled and used for exploratory metabolism or bioanalytical method experiments as deemed appropriate.

6.3.17Anti-Drug Antibodies

Serum will be collected to measure anti-drug antibodies. At Entry/Day 0, the sample should be collected prior to the dose of investigational agent/placebo. Post entry, serum should be collected as per the SOE. Date and time of collection should be recorded.

Samples will be analyzed at a laboratory approved by the sponsor and stored at a facility designated by the sponsor.

7.0ADVERSE EVENTS AND STUDY MONITORING

7.1Definitions of Adverse Events

Adverse Events of Special Interest
The following are AESIs for the agent AZD7442 or placebo for AZD7442:

- Grade ≥3 injection-site reactions (ISRs) within 72 hours of investigational agent/placebo administration (deemed related to study product as determined by the site investigator)
- Grade ≥1 allergic/hypersensitivity reactions within 24 hours of investigational agent/placebo administration (deemed related to study product as determined by the site investigator)
- Grade ≥2 other systemic reactions, including cytokine release syndrome, within 24 hours of investigational agent/placebo administration (deemed related to study product as determined by the site investigator).

7.3Recording Adverse Events

Post entry, the following non-lab AEs must be recorded on the eCRFs within 72 hours:

- Phase II and III: Grade 1 AEs

8.0CLINICAL MANAGEMENT ISSUES

8.1Toxicity

The second IM injection should not be administered if the participant experiences a Grade 3 or higher AE after the first IM injection. For any other AE, following the first IM injection, the participant's clinical status should be assessed before proceeding with the second IM injection.

8.2 Management of Side Effects

8.2.1 Overdose

An overdose is defined as receiving >300 mg of either of the component monoclonal antibodies. There is no known antidote for AZD7442 overdose. In the event this occurs, the participant should be closely monitored for AE/SAE and laboratory abnormalities, and supportive care provided as indicated.

8.2.2 Systemic Reactions Related to Investigational Agent Administration

All participants should be monitored closely, as there is a risk of systemic reaction (including anaphylaxis) with any biological agent.

Symptoms and signs that may occur as part of an administration reaction include, but are not limited to fever, chills, nausea, headache, bronchospasm, hypotension, angioedema, throat irritation, rash including urticaria, pruritus, myalgia, and dizziness.

The severity of systemic reactions will be assessed and reported using the criteria for infusion-related reactions in the Division of AIDS Table for Grading the Severity of Adult and Pediatric Adverse Events (DAIDS AE Grading Table), corrected Version 2.1, July 2017, which can be found on the DAIDS RSC website at https://rsc.niaid.nih.gov/clinical-research-sites/daids-adverse-event-grading-tables.

The clinical site should have necessary equipment and medications for the management of any administration reaction, which may include but is not limited to oxygen, IV fluid, epinephrine, acetaminophen and antihistamine.

Investigators should determine the severity of the reaction and manage reactions based on standard of care and their clinical judgment. If an administration reaction occurs, then supportive care should be provided in accordance with the signs and symptoms.

8.2.3 Hypersensitivity

Signs and symptoms of administration-related immediate hypersensitivity reactions may include, but are not limited to anaphylaxis, angioedema, bronchospasm, chills, diarrhea, hypotension, itching, skin rash, shortness of breath, urticaria, tachycardia, and throat irritation or tightness [8].

Participants will be closely monitored for immediate hypersensitivity reactions.

Sites should have appropriately trained medical staff and appropriate medical equipment available when study participants are receiving AZD7442. It is recommended that participants who experience a systemic hypersensitivity reaction be treated per the local standard of care.

8.2.4 Injection-Site Reactions

Injection-site reactions (ISRs) will be differentiated from the above generalized hypersensitivity

reactions by definition as localized pain/tenderness, induration, erythema, and/or formation of an ulceration or infection at the injection site. ISRs will be graded per the DAIDS AE Grading Table), corrected Version 2.1, July 2017.

8.3 Pregnancy

There are no data regarding the use of AZD7442 in participants who are pregnant, and therefore potential participants who are pregnant are not eligible during screening.

If a participant becomes pregnant during the study (post-entry), study follow up will continue for the duration of the study.

At the end of the pregnancy, outcome and adverse events for participant and infant will be recorded on the outcome eCRF.

8.4Breastfeeding

Since there are no data regarding the use of AZD7442 in participants who are breastfeeding, participants who are breastfeeding are not eligible for the study.

10.0STATISTICAL CONSIDERATIONS

10.2Outcome Measures

Primary and secondary outcome measures listed below will be addressed in the AZD7442 IM specific appendix to the study's primary Statistical Analysis Plan.

10.2.3Secondary Outcome Measures

The following secondary outcome measures will also be assessed:

10.2.3.13 Phase II only: New Grade 2 or higher AE through week 48.

11.0PHARMACOLOGY PLAN

11.1Pharmacology Objectives

The phase II pharmacology objective is to determine the pharmacokinetics of AZD7442 administered via the intramuscular route. For phase II, the pharmacology objective is to explore relationships between dose and concentration of AZD7442 with virology, symptoms, and oxygenation. For phase II an additional objective is to define whether there is differential time to reach the calculated effective concentration by site of injection.

11.2Pharmacology Study Design Overview

The Schedule of Evaluations shows the collection schedule for Phase II. AZD7442 has a long-elimination in preclinical animal studies, and is expected to be as long as 26 weeks in humans. The PK sample schedules are based on the long-elimination half-life of AZD7442 and are designed to meet the phase II objective of determination of AZD7442 pharmacokinetics. Approximately 40 participants (~20 receiving investigational agent) will have an additional sample collected on Day 1 (24 hours after dosing) to further define time to calculated effective concentration. Participants contributing Day 1 samples will be recruited

from selected domestic sites and the PK data from these participants will be analyzed (see section 6.3.15) as soon as the last of these participants completes Day 7 on study.

11.3 Pharmacology Data Analysis and Modeling

Pharmacokinetic data analysis of phase II data will use conventional and accepted approaches such as non-compartmental analysis or compartmental analysis to determine the PK characteristics of AZD7442 and its components. Population pharmacokinetic approaches (e.g., nonlinear mix effects modeling such as implemented in NONMEM) may also be used. The usual parameters of interest are area under the concentration-time curve (AUC), total body clearance (CL), elimination half-life ($T_{1/2}$), and maximum and minimum concentrations (C_{max}, C_{min}). PK characteristics from AZD7442 given intramuscularly (test) will be compared with those when given intravenously (reference) by calculation of geometric mean ratios of primary PK parameters (e.g. C_{max}, AUC). Exploration of relationships between dose and concentration of AZD7442 components with virology, symptoms, and oxygenation will be approached using conventional and accepted methods for pharmacokinetic/pharmacodynamic (PK/PD) data analyses. Such methods will include the E_{max} or sigmoid E_{max} model or structurally linked PK/PD models (as could be performed within NONMEM) to explore exposure-response relationships. Exposure-response relationships will be performed in conjunction with the protocol statisticians.

16.0 REFERENCES

1. Doherty M, Robertson MJ. Some early trends in immunology. Trends Immunol 2004;25:623-31.
2. Casadevall A, Dadachova E, Pirofski LA. Passive antibody therapy for infectious diseases. Nat Rev Microbiol 2004;2:695-703.
3. Saylor C, Dadachova E, Casadevall A. Monoclonal antibody-based therapies for microbial diseases. Vaccine 2009;27:G38-G46.
4. Casadevall A. The case for pathogen-specific therapy. Expert Opin Pharmacother 2009;10:1699-703.
5. IMpact-RSV Study Group. Palivizumab, a humanized respiratory syncytial virus monoclonal antibody, reduces hospitalization from respiratory syncytial virus infection in high-risk infants. Pediatrics 1998;102(3 Pt 1):531-7.
6. Fan P, Chi X, Liu L, et al. Potent neutralizing monoclonal antibodies against Ebola virus isolated from vaccinated donors. MAbs 2020;12(1):1742457.
7. Everds NE, Tarrant JM. Unexpected hematologic effects of biotherapeutics in nonclinical species and in humans. Toxicol Pathol 2013;41:280-302.
8. Investigator's Brochure, AstraZeneca AZD7442 3.0; 08 December 2020.
9. Dinnon KH, 3rd, Leist SR, Schafer A, et al. A mouse-adapted model of SARS-CoV-2 to test COVID-19 countermeasures. Nature 2020;586:560-6.
10. Chandrashekar A, Liu J, Martinot AJ, McMahan K, Mercado NB, Peter L, et al. SARS-CoV-2 infection protects against rechallenge in rhesus macaques. Science 2020;369(6505):812-17.
11. Yu J, Tostanoski LH, Peter L, et al. DNA vaccine protection against SARS-CoV-2 in rhesus macaques. Science 2020;369(6505):806-11.
12. Zuidema J, Pieters FAJM, Duchateau GSMJE. Release and absorption rate aspects of intramuscularly injected pharmaceuticals. Int J Pharm 1988;47:1-3.
13. Scialli AR, Bailey G, Beyer BK, et al. Potential seminal transport of pharmaceuticals to the conceptus. Reprod Toxicol 2015;58213-21.

APPENDIX IX: SAMPLE INFORMED CONSENT FOR STUDY DRUG AZD7442 ADMINISTERED AS AN INTRAMUSCULAR INJECTION

One of the study drugs that you might be assigned to in this study is AZD7442 or the placebo for AZD7442.

AZD7442 is a type of drug called a monoclonal antibody. Many antibodies are naturally made by your body and help fight diseases. AZD7442 is made in a laboratory. It is a combination of two monoclonal antibodies, meaning many copies of two antibodies designed to prevent SARS-CoV-2, the virus that causes COVID-19, from entering cells.

Your assignment is random, like the flip of a coin. You will be told about all the study drugs you may be assigned to in this study. If only one study drug is available, you will have an equal chance of receiving the study drug or placebo. If two study drugs are available, you will have a 2:1 chance of receiving a study drug or placebo. If three study drugs are available, you will have a 3:1 chance of receiving a study drug or placebo, and so forth. You will not be able to choose your group (study drug), and neither you, your study doctor, nor the study staff at your site will know whether you are receiving the study drug or placebo.

The US Food and Drug Administration (FDA) has not approved AZD7442 for general use by the public. However, we have told the FDA about this study and they have given us permission to conduct this study.

At this time, participants assigned to this study drug will be in the first part of the study (phase II), as described in the main consent.

ARE THERE ANY ADDITIONAL STUDY PROCEDURES IF I RECEIVE AZD7442 OR PLACEBO?

<u>Screening Visit</u>

- At your screening visit, if you can become pregnant, you will be asked to give blood (1 teaspoon) or a urine sample for a pregnancy test. You cannot receive AZD7442 or placebo if you are pregnant.

<u>Entry Visit</u>

- You will have blood drawn. This blood will be used for the following tests:
  - routine safety tests (liver and kidney tests and blood counts)
  - levels of the drug in your blood (you will have blood drawn before you receive the study drug and again 1 hour after)
  - levels of antibodies to the drug (your body's immune response to the drug)
- You will receive two intramuscular injections of AZD7442 or placebo. The administration will consist of one injection into the outside of the thigh, one in each thigh. You will be monitored for 2 hours after the injection.

<u>Study Visits on Days 3, 7, 14, and 28</u>

- You will have blood drawn. This blood will be used for the following tests:
  - routine safety tests (liver and kidney tests and blood counts) (days 3, 14, and 28)
  - levels of the drug and/or levels of antibodies to the drug (your body's immune response to the drug)

<u>Study Visits on Weeks 12 and 24</u>

- You will have blood drawn. This blood will be used for the following tests:
  - levels of the drug and levels of antibodies to the drug (your body's immune response to the drug)
- You will be asked whether you have had any new symptoms or clinical events since your last visit

<u>Study Visits on Weeks 36, 48, and 72</u>

- You will be contacted by phone by the study team to assess whether you have had any new symptoms or clinical events since your last visit
- You will answer questions about any potential COVID-19 related symptoms or conditions you have experienced

Extra Visits
Approximately 40 participants will be asked to return about 24 hours (1 day) after the Entry visit for an additional blood draw to test levels of the drug. The site staff will tell you if you may be one of these 40 participants.

HOW LONG WILL I BE IN THIS STUDY?

If you are assigned to AZD7442 or placebo for AZD7442, you will be in this study for 72 weeks.

WHAT ARE THE RISKS OF AZD7442?

There is a risk of serious and/or life-threatening side effects when non-study medications are taken with the study drugs. For your safety, you must tell the study doctor or nurse about all medications you are taking before you start the study.

Another risk is that the study drug used in this study may have side effects, some of which are listed below. Additionally, the study drug tested in the study may have unknown side effects in persons with SARS-CoV-2 infection. In a research study, all of the risks or side effects may not be known before you start the study. You need to tell your doctor or a member of the study team immediately if you experience any side effects.

Please note that these lists do not include all the side effects seen with this study drug. These lists include the more serious or common side effects with a known or possible relationship to the study drug. If you have questions concerning the additional side effects, please ask the medical staff at your site.

Risks Associated with AZD7442
There is limited safety data on AZD7442 since it has not been given to a lot of people. As of 08 December 2020, there have been no serious unexpected effects reported by >100 healthy people taking AZD7442 or placebo to date. Most effects after taking AZD7442 or placebo have been mild or moderate and have either all gone away or are getting better. This study is likely to be the first study where this study drug is given to people with COVID-19 disease.

Administration of AZD7442 may result in allergic reactions. Signs and symptoms of these reactions include:
- chills
- skin rash
- itching
- hives
- swelling of the face or other soft tissues
- low blood pressure
- rapid heart rate
- throat irritation or tightness
- tightening of the muscles that line the airways
- shortness of breath
- loose stools

Administration of AZD7442 may induce release of chemicals called cytokines in the body. These chemicals may induce allergic reactions listed above as well as:

- fever
- muscle aches
- nausea
- vomiting
- headache
- dizziness

Intramuscular injections of any chemical can cause:

- Redness, pain, and/or swelling at the injection site
- Tenderness of the muscle group or soreness with movement
- Ulceration
- Infection

Some of these reactions may be serious or life-threatening including:

- skin rash
- swelling of the face or other soft tissues
- low blood pressure
- rapid heart rate
- throat irritation or tightness
- tightening of the muscles that line the airways
- shortness of breath

You will be monitored closely during and after administration of study drug. Medical personnel, equipment, and medication will be available to manage these reactions appropriately if they occur.

Administration of study drug may also cause the following risks and discomforts:

- development of proteins (antibodies) against AZD7442. This may cause your body to get rid of AZD7442 more quickly or change the effect of AZD7442 on the body. Your blood will be tested to find out whether your body made antibodies to AZD7442. The anticipated risk of this is low because AZD7442 is a fully human antibody. Therefore, it is less likely to be seen as “foreign” by your body’s immune system and your body is less likely to form antibodies against AZD7442.
- mixture of antibody and other chemicals in the body that may be deposited in tissues such as blood vessels and kidneys.
- unexpected increase in virus reproduction in your body. Although this has been observed with some viruses, this has not been observed with COVID-19 or with the use of serum or plasma containing antibodies given to people with COVID-19. This risk of increased viral growth is perhaps greater when there is lower levels of antibodies in the blood in the presence of virus. To avoid this, AZD7442 will be given at a dose that is felt to be high enough to keep this from occurring.

<u>Effect on Future Vaccination</u>

The US Centers for Disease Control and Prevention (CDC) currently recommends that people wait at least 90 days after receiving antibody treatment before receiving a COVID-19 vaccine, because some antibodies remain in the body for about 90 days, and there is a chance that these antibodies could interfere with how your body responds to the vaccine during those 90 days. Some of the antibodies in this study including AZD7442 are designed to remain in the body for longer than 90 days. Although there is no further guidance available, there is a chance that these longer-lasting monoclonal antibodies could interfere with how your body responds to the vaccine even if you wait at least 90 days for the vaccine.

ARE THERE RISKS RELATED TO PREGNANCY AND BREASTFEEDING?

Pregnancy
Since there are no data regarding the use of this study drug in people who are pregnant, you are not eligible to receive this study drug if you are pregnant.
The study drug may involve risks to you (or to the embryo or fetus, if you or your partner become pregnant), which are currently unforeseen.

If you are engaging in sexual activity that could lead to pregnancy, you must agree to use effective contraception for 24 weeks after the study drugs are administered. This would include oral contraceptives, implanted contraceptives, or intrauterine devices.

If you are engaging in sexual activity that may lead to pregnancy in your partner, you must agree to either remain abstinent or use male contraceptives. You are also advised to inform your non-pregnant sexual partners that can become pregnant to use effective contraceptives for 24 weeks after the study drugs are administered to you.

If you have a pregnant partner you should use condoms during vaginal intercourse through 24 weeks after the study drugs administered.

If applicable, you should refrain from sperm donation for 24 weeks after study drug administration.

If at any point during the study you think you may be pregnant, you should let the staff at your site know so that a pregnancy test can be done.

Let your doctor know immediately if you become pregnant. If you become pregnant while on the study, you will be asked to continue to have study visits and the study staff would like to obtain information from you about the outcome of the pregnancy (even if it is after your participation in the study ends).

Breastfeeding
It is not known if this study drug is safe to use in people who are breastfeeding. You are not eligible to receive this study drug if you are breastfeeding.

APPENDIX XVI**II:** SIGNATURE PAGE – STUDY DRUGS

Consent forms for the following study drugs were reviewed (initial if reviewed with you):

_____ (initials) BAMLANIVIMAB INTRAVENOUS ADMINISTRATION

_____ (initials) BRII-196 and BRII-198 INTRAVENOUS ADMINISTRATION

_____ (initials) AZD7442 INTRAVENOUS ADMINISTRATION

_____ (initials) AZD7442 INTRAMUSCULAR ADMINISTRATION

_____ (initials) SNG001 INHALATION ADMINISTRATION

_____ (initials) CAMOSTAT ORAL ADMINISTRATION

_____ (initials) SAB-185 INTRAVENOUS ADMINISTRATION

_____ (initials) **BMS-986414 and BMS-986413 SUBCUTANEOUS ADMINISTRATION**
[Sites should mark agents that do not apply to a participant with an "N/A" before the page is given to the participant to sign]

If you have read this consent form (or had it explained to you), all your questions have been answered and you agree to take part in this study, please sign your name below.

______________________________	______________________________
Participant's Name (print)	Participant's Signature and Date
______________________________	______________________________
Participant's Legally Authorized Representative (As appropriate)Signature and Date	Legally Authorized Representative (print)
______________________________	______________________________
Study Staff Conducting	Study Staff's Signature and Date Consent Discussion (print)
______________________________	______________________________
Witness's Name (print)<br>(As appropriate)	Witness's Signature and Date

Section 2. Enrollment Criteria and High Risk Criteria Changes from Version 3.0 to Version 6.0

ACTIV-2/A5401 Changes
Versions 3.0 through 6.0

Protocol Version, Document, and Date	Protocol Amendment and Sections Affected
Version 3.0 Protocol, dated 22Dec2020	Inclusion/exclusion criteria, sample size, outcome measures, recording of AEs, and data and safety monitoring updated with the introduction of non-infused agents.<br>Master protocol sections updated: Schema (Design, Stratification, Population, Sample Size, and Outcome Measures), Section 1.1.4, Section 2.2, Section 3.1, Section 3.2, Section 3.3, Section 7.3, Section 10.1, Section 10.2.3, Section 10.3, Section 10.4.2, Section 10.5.1, and Section 10.5.2.
	Outcome measures in the phase II evaluation and one of the virology-based graduation guidelines for an investigational agent to be eligible for phase III evaluation were changed to SARS-CoV-2 RNA below lower limit of quantification by nasopharyngeal (NP) swabs.<br>Master protocol sections updated: Schema (Outcome Measures), Section 1.1.3, Section 3.1- Phase II Period of Evaluation, Section 10.2.1.2, Section 10.2.3.1, Section 10.2.3.8, Section 10.2.4.6, and Section 10.6.1.
	Saliva collection removed.<br>Master protocol sections updated: Section 1.2.6, Section 1.3.13, Table 6.1-1, Section 6.3.13, Section 10.2.3.8, and Section 10.2.3.10.<br>Appendix IV (Brii agents) section updated: Table 6.1-1.
	Oxygen saturation-based graduation guideline removed.<br>Master protocol section updated: Section 3.2.
	Additional information regarding the use of placebos and sharing of placebo groups for evaluating multiple investigational agents added.<br>Master protocol sections updated: Figure 3.0-1, Section 3.3, and Section 10.3.
	Oxygen saturation criterion expanded to allow measurement while on standard home oxygen supplementation level.<br>Master protocol section updated: Section 4.1.1.6.
	Exclusion criteria updated to allow people previously infected with SARS-CoV-2 to enroll.<br>Master protocol section updated: Section 4.1.2.2.
	Exclusion criteria updated to exclude people who previously received antibody-based treatment or prophylaxis.<br>Master protocol section updated: Section 4.1.2.5.
	Participant-completed study diary and staff review of study diary removed from Week 12 and Week 24.<br>Master protocol sections updated: Table 6.1-1, Table 6.1-2, and Section 6.3.11.<br>Appendix IV (Brii agents) sections updated: Table 6.1-1 and Table 6.1-2.
	Timing of study completion evaluations revised to account for new agents.<br>Master protocol section updated: Section 6.2.3.
	Instructions for vital status check updated to include check on study endpoints and additional participant status categories.<br>Master protocol section updated: Section 6.3.8.
	Instruction to record a descriptive modifier for previously reported medication conditions, which deteriorate and are recorded as AEs, added.<br>Master protocol section updated: Section 7.3.

Protocol Version, Document, and Date	Protocol Amendment and Sections Affected
	Guidance for slowing or stopping infused agents for agent-related reactions and Grade ≥3 events added.<br>Appendix II (LY3819253 agent) and Appendix IV (Brii agents) sections updated: Section 8.2.2, Section 8.2.3, and Section 9.1.
	References to BRII-196 at a concentration of 50mg/mL removed.<br>Appendix IV (Brii agents) sections updated: Section 5.2.1 and Section 5.2.2.
	Reporting of AESIs updated to include occurrence within 12 hours of investigational agent/placebo administration.<br>Appendix IV (Brii agents) section updated: Section 7.1.
	AZD7442 Intravenous Administration added as a study agent.<br>Appendix VI and Appendix VII added.
	AZD7442 Intramuscular Administration added as a study agent.<br>Appendix VIII and Appendix IX added.
	Inhaled Interferon-β1a (SNG001) added as a study agent.<br>Appendix X and Appendix XI added.
	Camostat added as a study agent.<br>Appendix XII and Appendix XIII added.
Version 3.0, LOA #1, dated 29Jan2021	Acceptable tests for documentation of presence of SARS-CoV-2 virus clarified to molecular (nucleic acid) or antigen test.<br>Master protocol sections updated: Schema (Population) and Section 4.1.1.3.
	Symptom duration for eligibility reduced to ≤8 days or 192 hours.<br>Master protocol sections updated: Schema (Population), Section 4.1.1.4, and Section 6.2.2.
	Collection of zinc and vitamin D levels removed.<br>Master protocol sections updated: Table 6.1-1, Table 6.1-2, and Section 6.3.14.<br>Appendix IV (Brii agents) and Appendix VI (AZ IV agent) sections updated: Table 6.1-1 and Table 6.1-2.<br>Appendix VIII (AZ IM agent), Appendix X (SNG001 agent), Appendix XII (Camostat agent) section updated: Table 6.1-1.
	Post-acute COVID-19 assessment added to visits after Day 28.<br>Master protocol sections updated: Table 6.1-1, Table 6.1-2, and Section 6.3.2.<br>Appendix IV (Brii agents) and Appendix VI (AZ IV agent) sections updated: Table 6.1-1 and Table 6.1-2.<br>Appendix VIII (AZ IM agent), Appendix X (SNG001 agent), and Appendix XII (Camostat agent) section updated: Table 6.1-1.
	Medical history instructions updated to require complete history for the 120 days prior to screening and entry.<br>Master protocol section updated: Section 6.3.3.
	Phase II and Phase III visits requiring targeted physical exam and time points for recording supplemental oxygen clarified.<br>Master protocol section updated: Section 6.3.6.
	Part way through the study, enrollment in phase 2 studies was restricted to individuals at “lower” risk of progression to severe COVID-19 as EUA mAbs became standard of care for higher-risk participants (V6.0). Phase II primary outcome measures updated to correct the symptom outcome definition.<br>Master protocol sections updated: Section 10.2.1.1 and Section 10.4.1.

Protocol Version, Document, and Date	Protocol Amendment and Sections Affected
	Phase III secondary outcome measures updated to remove duration of fever.<br>Master protocol section updated: Section 10.2.3.
	BRII-196 and BRII-198 concentration of 50 mg/mL restored.<br>Appendix IV (Brii agents) sections updated: Section 5.2.1 and Section 5.2.2.
	Instructions for collection vital signs during investigational agent infusion or IM administration and collection of PK samples related to agent dosing clarified.<br>Appendix IV (Brii agents), Appendix VI (AZ IV), and Appendix VIII (AZ IM) sections updated: Section 6.3.9 and Section 6.3.16 (Appendix IV) or Section 6.3.15 (Appendix VI and Appendix VIII).
	Post-entry non-lab Grade 1 AEs that are deemed related to study product by the site investigator added to AEs recorded on eCRFs within 72 hours.<br>Appendix IV (Brii agents), Appendix VI (AZ IV), and Appendix VIII (AZ IM) sections updated: Section 7.3.<br>Appendix X (SNG001 agent) section updated: Section 7.3.
	Week 36, Week 48, and Week 72 phone visits for assessment of safety and post-acute COVID added.<br>Appendix X (SNG001 agent) and Appendix XII (Camostat agent) section updated: Table 6.1-1.
	SNG001 agent administration instructions updated to allow first dose to be taken at the clinic and PK instructions updated to allow analyses on select stored samples.<br>Appendix X (SNG001 agent) sections updated: Section 5.1.2 and Section 6.3.15.
Version 3.0, LOA #2, dated 10Feb2021	Transition into a larger phase III evaluation without pause in enrollment, if safety data are acceptable as determined by the study's Data and Safety Monitoring Board (DSMB), added for infused agents.<br>Master protocol sections updated: Section 3.1, Figure 3.0-1, Section 3.2, Section 10.5.1, Section 10.5.2, and Figure 10.5.2-1.
Version 3.0, CM #1, dated 17Mar2021	Post-entry non-lab AEs recorded on eCRFs within 72 hours clarified to Grade 1 AEs.<br>Master protocol section updated: Section 7.3.
Version 4.0 protocol, dated 22Feb2021	Higher risk definition updated to include no history of COVID-19 vaccination.<br>Master protocol section updated: Schema (Population).
	Smoker criterion for higher risk updated to cigarette smoking within the past 30 days and history of at least 100 lifetime cigarettes.<br>Master protocol section updated: Schema (Population).
	Restriction on vaccination prior to study entry removed.<br>Master protocol section updated: Section 4.1.2.
	Acceptable tests for presence of SARS-CoV-2 virus clarified to molecular (nucleic acid) or antigen tests.<br>Master protocol section updated: Section 4.1.2.2.
	Duration on study updated to Week 72 for SNG001 and camostat.<br>Appendix X (SNG001 agent) and Appendix XII (Camostat agent) section updated: Schema (Duration).
	SAB-185 added as a study agent.<br>Appendix XIV and Appendix XV added.

Protocol Version, Document, and Date	Protocol Amendment and Sections Affected
Version 5.0 protocol, dated 02Apr2021	Recent updates to the Investigator’s Brochures (IB) for the AZ agents added.<br>Appendix VI (AZ IV) and Appendix VIII (AZ IM) section updated: Section 2.2.
	Instructions for collection of vital signs during AZ IV agent infusion clarified to occur at end of infusion.<br>Appendix VI (AZ IV) section updated: Section 6.3.9.
	Order of administration of AZ IM agent injections and data recorded on eCRF clarified.<br>Appendix VIII (AZ IM) section updated: Section 5.1.2.
	Instructions for assessment of adherence for camostat agent clarified to days when doses are taken.<br>Appendix XII (Camostat agent) sections updated: Table 6.1-1 and Section 6.3.17.
	Additional PK data added to SAB agent rationale.<br>Appendix XIV (SAB agent) section updated: Section 2.2.
	PK study at Day 1 added to Phase II of SAB agent.<br>Appendix XIV (SAB agent) sections updated: Table 6.1-1 and Section 6.3.15.
	Instructions for management of hypersensitivity to SAB agent clarified.<br>Appendix XIV (SAB agent) section updated: Section 8.2.3.
	BMS-986414 and BMS-986413 added as study agents.<br>Appendix XVI and Appendix XVII added.

All participants in Tixagevimab/Cilgavimab (AZD7442) groups enrolled during protocol V3.0- V5.0, but have had had ongoing follow-up under a subsequent protocol version not impacting eligibility or the data presented in the primary analyses.

Changes to the definition of "High Risk" across ACTIV-2 Protocol Versions enrolling to the tixagevimab/cilgavimab groups

High Risk Condition	Protocol Version	
	**3.0**	**4.0, 5.0**[1]
**Age**	60 years and older	60 years and older
**Current smoker**[2]	Yes	Yes
**Exogenous or endogenous immunosuppression**[3]	Yes	Yes
**Chronic lung disease or asthma (* requiring daily prescribed therapy)**	Yes*	Yes*
**Obesity**	BMI >35	BMI >35
**Hypertension (* with at least one medication recommended or prescribed)**	Yes*	Yes*
**Cardiovascular disease**[4]	Yes	Yes
**Diabetes**	Yes	Yes
**Chronic kidney disease (*requiring hemodialysis or peritoneal dialysis)**	Yes*	Yes*
**Chronic liver disease (* with history of cirrhosis)**	Yes*	Yes*
**Active cancer, other than localized skin cancer**	Yes	Yes

1. Under protocol version 4.0 (and subsequent versions), participants with a history of SARS-CoV-2 vaccination were considered "low risk", regardless of other risk factors.
2. Under protocol version 3.0, current smoker was defined as 'any inhaled nicotine product'. Under protocol version 4.0 and 5.0, current smoker was defined as cigarette smoking within the past 30 days AND a history of at least 100 lifetime cigarettes
3. Exogenous or endogenous immunosuppression was defined as HIV infection with CD4 count <200 cells/mm^3, receiving corticosteroids equivalent to prednisone ≥20mg daily for at least 14 consecutive days within 30 days prior to study entry, or treatment with biologics (e.g., infliximab, abalizumab, ustekinumab, etc.), immunomodulators (e.g., methotrexate, 6MP, azathioprine, etc.).
4. Cardiovascular disease was defined as history of any of the following: myocardial infarction, stroke, transient ischemic attack, heart failure, angina with prescribed nitroglycerin, coronary artery bypass grafts, percutaneous coronary intervention (PCI), carotid endarterectomy, and aortic bypass).

Section 3. Statistical Analysis Plan 7.0

ACTIV-2/ACTG A5401
Primary Statistical Analysis Plan
Phase II/III Placebo-Controlled and Phase III Active-Controlled
Study Components
Version 7.0

Adaptive Platform Treatment Trial for Outpatients with COVID-19
(Adapt Out COVID)

Based on Protocol Version 7.0 and
Letter of Amendment #1 to Protocol v7.0
(Applies to Agents Entered into ACTIV-2 in Protocol Versions 2.0, 3.0, 4.0 & 5.0)
ClinicalTrials.gov Identifier: NCT04518410

October 24, 2021
Created by:
<*Authors Redacted*>
Harvard T.H. Chan School of Public Health

Table of Contents

VERSION HISTORY 3
GLOSSARY OF TERMS 4
1 INTRODUCTION 6
1.1 Purpose 6
1.2 Version History of this SAP 6
2 STUDY OVERVIEW 6
2.1 Study Design 6
2.2 Randomization Process 7
2.3 Study Objectives 8
2.3.1 Primary Objectives 8
2.3.2 Secondary Objectives 8
2.3.3 Exploratory Objectives 9
2.4 Overview of Sample Size Considerations 9
2.4.1 Phase II – Placebo-Controlled Superiority 9
2.4.2 Phase III – Placebo-Controlled Superiority Trial 10
2.4.3 Phase III – Active-Controlled Non-Inferiority Trial 10
2.5 Overview of Formal Interim Monitoring 13
2.5.1 Phase II – Placebo-Controlled Superiority 13
2.5.2 Phase III – Placebo-Controlled Superiority 13
2.5.3 Phase III – Active-Controlled Non-Inferiority 14
2.6 Graduation to Phase III 15
3 OUTCOME MEASURES 15
3.1 Primary Outcome Measures: Phase III 15
3.2 Primary Outcome Measures: Phase II 16
3.3 Secondary Outcome Measures 16
3.4 Other Outcome Measures 18
4 STATISTICAL PRINCIPLES 19
4.1 General Considerations 19
5 ANALYSIS APPROACHES 21
5.1 Analyses of the Primary Objectives 21
5.1.2 Phase III Primary Objective for Efficacy: Placebo-Controlled Superiority Evaluation .. 21
5.1.2 Phase III Primary Objective for Efficacy: Active-Controlled Non-Inferiority Evaluation 24
5.1.2 Primary Safety (Phase II and III) 25
5.1.3 Primary Clinical Symptoms (Phase II) 26
5.1.4 Primary Virologic (Phase II) 28
5.2 Analyses of Secondary Objectives 30
5.2.1 Secondary Clinical Symptoms 30
5.2.2 Secondary Virology 35
5.3 Exploratory Analyses 38
5.3.1 New SARS-CoV-2 among Household Contacts 38
5.3.2 Hospitalization Course 38
5.3.3 Resistance Mutations 38
5.4 Interim Analysis Considerations 38
6 APPENDIX 1: ALGORITHM FOR HANDLING MISSING SYMPTOM EVALUATIONS FOR THE PRIMARY PHASE II SYMPTOM OUTCOME MEASURE. 39
7 APPENDIX 2: STATISTICAL CONSIDERATIONS FOR BRII-198 + BRII-196 42
7.1 Randomization Details 42
7.2 Secondary Outcome Measures 42
7.3 Analysis Approaches 42
8 APPENDIX 3: STATISTICAL CONSIDERATIONS FOR AZD7442 IV 43
8.1 Secondary Outcome Measures 43
8.2 Analysis Approaches 43
9 APPENDIX 4: STATISTICAL CONSIDERATIONS FOR AZD7742 IM 44

9.1 Secondary Outcome Measures 44
9.2 Analysis Approaches 44
10 APPENDIX 5: STATISTICAL CONSIDERATIONS FOR SNG001 ERROR! BOOKMARK NOT DEFINED.
10.1 Objectives .... Error! Bookmark not defined.
10.1.1 Secondary Objectives .... Error! Bookmark not defined.
10.1.2 Exploratory Objectives .... Error! Bookmark not defined.
10.2 Outcome Measures .... Error! Bookmark not defined.
10.2.1 Secondary Outcome Measures .... Error! Bookmark not defined.
10.2.2 Other Outcome Measures .... Error! Bookmark not defined.
10.3 Analysis Approaches .... Error! Bookmark not defined.
11 APPENDIX 6: STATISTICAL CONSIDERATIONS FOR CAMOSTAT. ERROR! BOOKMARK NOT DEFINED.
11.1 Objectives .... Error! Bookmark not defined.
11.1.1 Secondary Objectives .... Error! Bookmark not defined.
11.1.2 Exploratory Objectives .... Error! Bookmark not defined.
11.2 Outcome Measures .... Error! Bookmark not defined.
11.2.1 Secondary Outcome Measures .... Error! Bookmark not defined.
11.3 Analysis Approaches .... Error! Bookmark not defined.
12 APPENDIX 7: STATISTICAL CONSIDERATIONS FOR SAB-185 ERROR! BOOKMARK NOT DEFINED.
13 APPENDIX 8: STATISTICAL CONSIDERATIONS FOR BMS-986414 + BMS-986413. ERROR! BOOKMARK NOT DEFINED.
13.1 Secondary Outcome Measures .... Error! Bookmark not defined.
13.2 Analysis Approaches .... Error! Bookmark not defined.

Version History

Version	Changes Made	Date Finalized
1.0	Original Version	July 29, 2020
2.0	Updated SAP to address the following:<br>- Changes to the protocol based on CMs and LOAs<br>- Typos/errors found after finalization of version 1.0<br>- Revised handing of missing symptom, virology, and oxygen saturation data in analysis<br>- Clarifying imputation of virology data<br>- Add analyses of resistance mutations<br>- Added LY3819253-specific appendix to address LOA #3	Jan 19, 2021
3.0	New SAP to describe analysis plans for agents introduced in protocol versions 2.0, 3.0 and 4.0	April 2, 2021
4.0	Updated SAP to address the following:<br>- Added appendix for BMS-986414 + BMS-986413 agent introduced in protocol version 5.0<br>- Added AUC outcome in phase III for SARS-CoV-2 RNA from nasal swabs<br>- Added visit and analysis windows for new study weeks (36, 48, 72)<br>- Clarified aspects of imputation for symptoms duration outcome<br>- Updated plans for determining interim stopping boundaries (using EAST software)	April 15, 2021
5.0	Update SAP to address changed implemented in protocol version 6.0. Specifically:<br>- Updated SAP to reflect changes to study design, randomization, study objectives, interim monitoring, and outcome measures	June 24, 2021

	- Clarified that although phase II enrollment was restricted to those at 'lower' risk of progression, all participants who enrolled under prior versions of the protocol would be included in all analyses (i.e., 'higher' risk participants)<br>- Removed risk stratification subgroup analyses for phase III outcome measures as all phase III is restricted to those at 'higher risk'<br>- Clarified that risk stratification subgroup analyses for phase II outcome measures will depend on the number of 'higher' risk participants enrolled<br>- Added supportive and sensitivity analyses for phase II primary symptom outcome measure per FDA recommendation<br>- Removed exploratory virology analyses<br>- Edited typographical errors	
6.0	Update SAP to address changed implemented in protocol version 7.0 and Letter of Amendment 1. Specifically:<br>- Updated SAP to reflect changes in study objectives, schedules for some evaluations, and associated outcome measures.<br>- Updated SAP to reflect changes in interim monitoring, mainly related to changes in graduation criteria.<br>- Updated this SAP to focus on the placebo-controlled phase III evaluation. (Note that a separate *revision to the* SAP will be prepared for the active-controlled phase III evaluation introduced in protocol version 7.0). [Note that the italicized text was added in version 7.0 of the SAP].	September 13, 2021
7.0	Updated SAP with the following major changes:<br>- To add details that are specific to the active-controlled phase 3 evaluation of agents based on protocol version 7.0 and Letter of Amendment 1.<br>- To edit some text to provide clarity concerning the analysis approaches which are the same regardless of whether a placebo control or active control is involved. In part, to achieve this, the terminology "comparator intervention" is often used.<br>- To replace the previous section 5.4 concerning interim analysis considerations for the placebo-controlled phase III trial (which have been completed) with interim analysis considerations for the active-controlled phase III trial.<br>- To indicate exclusion from analysis of viral shedding results from samples labelled as "Thawed', 'Destroyed', 'Quantity Not Sufficient' or "Invalid Specimen' as approved by the trial sponsor.<br>- To focus subgroup analysis by country on analyses for participants enrolled at U.S. versus non-U.S. sites, and to add a subgroup analysis by SARS-CoV-2 variants.	October 24, 2021

Glossary of Terms

ACTIV Accelerating COVID-19 Therapeutic Interventions and Vaccines
AEAdverse Event
AUC Area Under the Curve
CMClarification Memo
COVID-19 Coronavirus Disease 2019
DSMB Data and Safety Monitoring Board
ECMOExtracorporeal Membrane Oxygenation
FDAFood and Drug Administration

GEEGeneralized Estimating Equations
ICUIntensive Care Unit
IPCWInverse Probability of Censoring Weights
LOALetter of Amendment
LoDLimit of Detection
LLoQLower Limit of Quantification
LTFULoss to Follow Up
MCARMissing Completely at Random
mITTModified Intent-to-Treat
NIAIDNational Institute of Allergy and Infectious Diseases
NPNasopharyngeal
SAP Statistical Analysis Plan
SARS-CoV-2 Severe Acute Respiratory Syndrome Coronavirus 2
SOESchedule of Evaluations
TOCTrial Oversight Committee
ULoQUpper Limit of Quantification

Introduction

Purpose

This Primary Statistical Analysis Plan (referred to as “SAP” in this document) describes the general framework for the interim and key statistical analyses of the phase II and phase III placebo-controlled investigations of ACTIV-2/A5401, as well as the phase III active-controlled investigation introduced in protocol version 7.0. This SAP addresses the primary and secondary objectives and associated outcome measures, as well as a subset of exploratory objectives and associated outcome measures that may be included in primary manuscripts of the study. Hence, it also describes the primary and secondary outcome measures for which results will be posted on ClinicalTrials.gov. This SAP outlines the general statistical approaches that will be used in the analysis of the study and has been developed to facilitate discussion of the statistical analysis components among the study team, industry collaborators, and study sponsor; and to provide agreement between the study team and statisticians regarding the statistical analyses to be performed and presented. Given the design of the study and that, multiple investigational agents will be studied; separate analysis reports may be generated for each investigational agent and each study phase. Analysis considerations that are specific to a given investigational agent are provided in agent-specific appendices to this SAP.

Version History of this SAP

ACTIV-2 is a platform trial designed to evaluate multiple agents under a master protocol. Versions 1.0 and 2.0 of the SAP, which were based on protocol version 1.0, were developed with the idea that they would be applied to all agents included in the study. However, there were sufficient changes between protocol version 1.0 and subsequent versions of the protocol that versions 1.0 and 2.0 of the SAP were limited to analyses of data evaluating the first agent in ACTIV-2, referred to as LY3819253.

Version 3.0 of the SAP was developed for agents entering under protocol versions 2.0, 3.0 and 4.0, and was not used to describe analyses of data for LY3819253. Because version 3.0 of the SAP applied to different agents from version 2.0 of the SAP, changes between version 2.0 and version 3.0 of the SAP are not detailed here. Analyses that are only for a specific agent or agents are described in agent-specific supplements to the SAP. SAP version 4.0 was developed to address changes in protocol version 5.0 and to make adjustments noted in the version history table.

SAP version 5.0 was developed to address changes made to the protocol in version 6.0. Protocol version 6.0 stated that enrollment to all agents (except BRII-196+BRII-198 which was already in phase III), will stop after the phase II enrollment is completed and there will be no enrollment to a placebo-controlled phase III evaluation of these agents. SAP version 5.0 therefore described planned statistical analyses for both the phase II and the phase III evaluations of BRII-196+BRII-198 versus placebo, and for the phase II evaluations of all other agents that entered the study under protocol versions 3.0, 4.0 and 5.0 and which are also being compared to a placebo. In addition, SAP version 5.0 addressed some small changes to the schedule of evaluations and outcome measures introduced in protocol version 6.0.

Protocol version 7.0 introduced an open-label non-inferiority phase III design to compare investigational agents to an active-comparator among persons at higher risk of progression to hospitalization or death. This phase III evaluation is separate from the phase II superiority evaluation of agents compared to placebo among persons at lower risk for progression to hospitalization or death. Changes introduced in SAP version 6.0 focused on changes made under protocol version 7.0 (and letter of amendment #1) that related to the placebo-controlled superiority phase II/III design (note: BRII-196+BRII-198 is the only agent that enrolled in the placebo-controlled phase III design). Changes introduced in SAP version 7.0 address the introduction of the active-controlled non-inferiority phase III trial in protocol version 7.0 (and letter of amendment #1). SAP version 7.0 also introduces the exclusion from statistical analysis of results generated from problematic virologic samples based on a decision made by the DAIDS and study team. In addition, section 5.4 concerning interim analysis considerations was revised to replace considerations for the placebo-controlled phase III trial for which DSMB monitoring has been completed with considerations for the active-controlled phase III trial. Finally, adjustments were made to focus subgroup analysis by country on analyses for participants enrolled at U.S. versus non-U.S. sites, and to add a subgroup analysis by SARS-CoV-2 variants.

Study Overview

Study Design

The study design described in this section reflects details in protocol version 7.0 and letter of amendment 1.

ACTIV-2/A5401 is a master protocol to evaluate the safety and efficacy of investigational agents for the treatment of symptomatic non-hospitalized adults with COVID-19. The study is designed to evaluate both infused and non-infused investigational agents.

The trial has a randomized controlled adaptive platform study design that allows agents to be added or dropped during the course of the study for efficient phase II and phase III testing of new agents within the same trial infrastructure.

Version 7.0 of the protocol provides for a blinded phase II evaluation of an investigational agent compared to placebo among participants at lower risk for progression to hospitalization or death, regardless of the mode of administration of the agent; for some agents, enrollment to higher risk participants in phase II was allowed under earlier protocol versions. Agents that graduate to phase III (after initiation of this protocol version) will be evaluated in persons at higher risk for progression to hospitalization or death for non-inferiority to an active comparator, the monoclonal antibody combination of casirivimab plus imdevimab (REGEN-COV, Regeneron), which has been shown to be effective in this population in preventing hospitalization or death. Protocol version 7.0 also provides for continued follow-up of participants enrolled into a placebo-controlled phase III trial evaluating the combination monoclonal antibody agent, BRII-196 + BRII-198.

When two or more agents are being evaluated in the same phase of the study, the trial design includes sharing of the control group (placebo in phase II and active comparator in phase III) for efficient evaluation of each agent. Note that enrollment to BRII-196+BRII-198 did not coincide with enrollment to other agents in phase III.

Eligible participants will have intensive follow-up through day 28, followed by limited follow up through at least week 72 weeks in phase II and phase III.

The study population consists of adults (≥ 18 years of age) with documented positive SARS-CoV-2 molecular test results collected within 240 hours (10 days) prior to study entry with no more than 7 days of symptoms of COVID-19 prior to study entry (this criterion allowed up to 10 days in protocol version 3.0 and earlier, and up to 8 days in protocol versions 4.0 and 5.0) previous versions of the protocol), and with presence of select symptoms within 24 hours of study entry.

Randomization Process

The phase II trial and the active-controlled phase III trial involve different populations and have separate randomizations. However, the structure of the randomization process is the same for each of the two trials, as described in the following.

The randomization process is designed to be flexible for this adaptive platform study, in which participants may be eligible for randomization to different investigational agents, and investigational agents can be added or dropped during the course of the study. The ultimate intent is to have a similar number of concurrently randomized participants on a given investigational agent and on the comparator group for that agent (i.e. combining participants who were eligible to receive the agent but who were randomized to any of the available placebos in phase II or to the active comparator in phase III).

To achieve having a similar number of participants on the active arm and in the pooled comparator group for a given investigational agent, the randomization occurs in two steps within each trial.

The first randomization is to *Agent Group*. For a given participant, the first randomization assigns a participant with equal probability among the n agents in the trial (e.g., a 1:1 ratio for two agents, 1:1:1 ratio for three agents, etc.) that the participant is eligible to receive (based on protocol eligibility criteria and the set of agents available at the clinical site at which the participant is being enrolled). Trial phase for an agent is accounted for in the participant eligibility (i.e. by the classification of their risk for hospitalization/death as lower or higher). In the event that a participant is only eligible for one investigational agent (n=1), then they are assigned to the one appropriate Agent Group.

Immediately following the first randomization, participants are randomized within an Agent Group in the second randomization to the (active) investigational agent or appropriate comparator (the matching placebo for agents in phase II, or the active comparator for agents in phase III). For a given participant, the probability of assignment to the active agent or comparator in the second randomization depends on the number of agents currently under investigation that the participant was eligible to receive, as phase II and phase III have distinct populations (phase II is restricted to those at lower risk of progression to hospitalization and death, and phase III is restricted to those at higher risk).

Both the first and second randomizations involve blocked stratified randomization. In phase II, both the first and second randomizations are stratified by time from symptom onset (≤5 days vs >5 days), however, in previous versions of the protocol, in which both ‘higher’ and ‘lower’ risk participants could be

randomized to agents in phase II evaluation, both the first and second randomizations were also stratified by risk group (‘higher’ vs ‘lower’). In the active-controlled phase III trial introduced in protocol version 7.0, both randomization steps are stratified by country. Under previous versions of the protocol for the placebo-controlled phase III, both randomization steps were only stratified by time from symptom onset (≤ 5 days vs > 5 days). Additional details on randomization are provided in protocol section 10.3.

Study Objectives

The following sections list the primary, secondary and exploratory objectives from protocol version 7.0 (and letter of amendment #1); corresponding protocol numbering is shown in brackets. This Primary SAP addresses all of the primary and secondary objectives shown below, with the exception of the secondary PK objectives in phase 2, which will be addressed outside of this SAP. In addition, exploratory objectives 1 and 4 will also be addressed in this SAP; however, other exploratory objectives will be addressed separately.

Primary Objectives

1) Phases II and III: To evaluate safety of the investigational agent [Protocol Objective 1.1.1].

2) Phase II: To determine efficacy of the investigational agent to reduce the duration of COVID-19 symptoms through study day 28 [Protocol Objective 1.1.2].

3) Phase II: To determine the efficacy of the investigational agent to increase the proportion of participants with nasopharyngeal (NP) SARS-CoV-2 RNA below the lower limit of quantification (LLoQ) at study days 3, 7, and 14 [Protocol Objective 1.1.3].

4) Phase III: To determine if the investigational agent will prevent the composite endpoint of either hospitalization due to any cause or death due to any cause through study day 28. Hospitalization is defined as ≥24 hours of acute care, in a hospital or similar acute care facility, including Emergency Rooms or temporary facilities instituted to address medical needs of those with severe COVID-19 during the COVID-19 pandemic [Protocol Objective 1.1.4].

Secondary Objectives

1) Phases II and III: To determine whether the investigational agent reduces a COVID-19 severity ranking scale based on COVID-19-associated symptom burden (severity and duration), hospitalization, and death, through study day 28 [Protocol Objective 1.2.1].

2) Phases II and III: To determine whether the investigational agent reduces the progression of COVID-19-associated symptoms [Protocol Objective 1.2.2].

3) Phases II and III: To determine if the investigational agent reduces levels of SARS-CoV-2 RNA in NP swabs [Protocol Objective 1.2.3].

4) Phase III: To determine the efficacy of the investigational agent to increase the proportion of participants with NP SARS-CoV-2 RNA below the LLoQ at study day 3 [Protocol Objective 1.2.4]

5) Phase II: To determine the pharmacokinetics of the investigational agent [Protocol Objective 1.2.5].

6) Phase II: To determine efficacy of the investigational agent to obtain pulse oximetry measurement of ≥ 96% through day 28 [Protocol Objective 1.2.6].

7) Phase III: To determine if the investigational agent will prevent the composite endpoint of either hospitalization due to any cause or death due to any cause through study week 72 [Protocol Objective 1.2.7].

8) Phase III: To evaluate if the investigational agent reduces the time to sustained symptom resolution through study day 28 [Protocol Objective 1.2.8].

9) Phase III: To determine if the investigational agent will prevent the composite endpoint of hospitalization or death through study day 28, excluding hospitalizations that are determined to be unrelated to COVID-19 [Protocol Objective 1.2.9 (introduced in letter of amendment 1 to protocol version 7.0)].

Exploratory Objectives

1) Phases II and III: To explore the impact of the investigational agent on participant-reported rates of SARS-CoV-2 positivity of household contacts [Protocol Objective 1.3.1].

2) Phases II and III: To explore if baseline and follow-up hematology, chemistry, coagulation, viral, and inflammatory biomarkers are associated with clinical and virologic outcomes in relation to investigational agent use [Protocol Objective 1.3.2].

3) Phases II and III: To explore possible predictors of outcomes and differences between investigational agent and control (placebo in phase II and active comparator in phase III) across the study population, notably sex, time from symptom onset to start of investigational agent, and race/ethnicity [Protocol Objective 1.3.3].

4) Phases II and III: To explore if the investigational agent changes the hospital course in those hospitalized [Protocol Objective 1.3.4].

5) Phases II and III: To explore and develop a model for the interrelationships between virologic outcomes, clinical symptoms, and, in phase III, hospitalization, and death in each study group [Protocol Objective 1.3.5].

6) Phases II and III: To explore the relationship between exposure to the investigational agent and SARS-CoV-2 innate, humoral or cellular response, including anti-drug antibodies, as appropriate per investigational agent [Protocol Objective 1.3.6].

7) Phases II and III: To explore baseline and emergent viral resistance to the investigational agent [Protocol Objective 1.3.7].

8) Phases II and III: To explore the association between viral genotypes and phenotypes, and clinical outcomes and response to agents [Protocol Objective 1.3.8].

9) Phases II and III: To explore the association between host genetics and clinical outcomes and response to agents [Protocol Objective 1.3.9]

10) Phases II and III: To explore relationships between dose and concentration of investigational agent with virology, symptoms, and oxygenation [Protocol Objective 1.3.10].

11) Phases II and III: To explore the prevalence, severity, and types of persistent symptoms and clinical sequelae in participants through end of study follow-up [Protocol Objective 1.3.11].

12) Phases II and III: To explore measures of psychological health, functional health, and health-related quality of life in participants through end of study follow-up [Protocol Objective 1.3.12].

Overview of Sample Size Considerations

The sample size for phase II was the same under protocol versions 2.0 to 7.0. The sample size for the placebo-controlled superiority phase III design was also the same under protocol versions 2.0 to 6.0 (it was originally defined in Appendix IV of protocol version 2.0 for the agent entered into the study under protocol version 2.0) and is currently detailed in Appendix V of protocol version 7.0 for the BRII-196+BRII-198 agent. Details on the sample size for the non-inferiority active-controlled phase III design are from protocol version 7.0 section 10.4.

Phase II – Placebo-Controlled Superiority

For each investigational agent in phase II, the proposed sample size is 220 participants, consisting of 110 participants who receive that agent and 110 participants who are concurrently randomized to placebo

control. Participants who are randomized but do not start their randomized investigational agent or placebo will not be followed.

This sample size is chosen to give high power to identify an active agent on the basis of the primary virology outcome, due to limited data on the variability of symptom duration in the outpatient COVID-19 population.

Assuming 100 participants in each group will have NP swabs available at a scheduled measurement time, there is at least 82% power to detect a 20% absolute increase in the percentage of participants with SARS-CoV-2 RNA < LLoQ in the investigational agent group vs concurrent placebo group, regardless of the assumed percent <LLoQ in the placebo group (range: 10-70%); calculated for the comparison of two proportions using a normal approximation to the binomial distribution, unpooled variance, and two-sided Type I error rate of 5%.

Phase III – Placebo-Controlled Superiority Trial

The proposed sample size is 842 participants consisting of 421 participants who receive the active agent and 421 participants who are concurrently randomized to placebo control. This sample size includes the enrollment that occurred during the phase II placebo-controlled evaluation of an agent. Participants who are randomized but do not start their randomized investigational agent or placebo will not be followed.

This sample size has been chosen to provide 90% power to detect a relative reduction of 50% in the proportion of participants hospitalized/dying between the study groups. This is based on the following assumptions:

- Proportion hospitalized/dying in the placebo group is 15%;
- Two-sided test of two proportions with 5% Type I error rate;
- Three interim analyses and one final analysis, approximately equally spaced, with stopping guideline for efficacy of an investigational agent versus concurrent placebo determined using the Lan-DeMets spending function approach with an O'Brien and Fleming boundary, and a non-binding stopping guidelines for futility using a Gamma(-2) Type II spending function also implemented using the Lan-DeMets spending function;
- Allowance for 5% of participants to be lost-to-follow-up prior to being hospitalized or dying, and non-informative loss-to-follow-up.

Phase III – Active-Controlled Non-Inferiority Trial

The active-controlled Phase III trial is focused on a non-inferiority comparison of the proportion of participants who are hospitalized or who die through to 28 days for an investigational agent versus an active comparator agent, specifically the monoclonal antibody combination of casirivimab plus imdevimab. The non-inferiority margin for the absolute difference in proportion hospitalized/dead is 3% (investigational agent minus active comparator agent); the rationale for this choice is described in Section 3.1 of protocol version 7.0. Non-inferiority will be considered established if a two-sided exact 95% confidence interval for the absolute difference is entirely below 3%. Details of the construction of the confidence interval are in section 10.6 pf the protocol and are included further below in this SAP.

The sample size differs between infused investigational agents (600 for the investigational agent and 600 for the concurrently randomized active comparator) and non-infused investigational agents (800 per arm instead of 600 per arm). The rationale for this is that there may be broader clinical utility for non-infused agents such that a slightly higher true hospitalization/death rate may be tolerated in clinical practice.

Sample Size Justification for Infused Investigational Agents

For the evaluation of a specific infused investigational agent, the sample size is 1200 participants including approximately 600 participants randomized to receive the infused investigational agent and approximately 600 participants (who were eligible to receive the infused investigational agent) concurrently randomized to receive the active comparator agent. This sample size has been chosen to provide close to 90% power to establish non-inferiority assuming that the true proportion hospitalized/dead for both the infused investigational agent and the active comparator agent is 2.3%. The rate of 2.3% is based on the observed proportion for casirivimab plus imdevimab combining across doses in the subpopulation of the Regeneron COV-2067 clinical trial who met the criteria for being at high risk of progression to hospitalization/death (FDA communication to DAIDS/NIAID, May 2021). No adjustment for loss to follow-up is made in the sample size as the primary analysis will be based on the observed number of hospitalizations divided by the number of participants who initiated study treatment. In addition, the impact of any loss to follow-up is expected to be minimal as there will be regular contact between research site staff and participants (or their secondary contacts) and previous experience in the study and other trials has shown that the large majority of hospitalizations/deaths occur early in follow-up (first two weeks of follow-up).

The potential power of the study was evaluated in two ways using the PASS version 15 sample size calculation software. Both used a non-inferiority hypothesis testing approach based on use of the Miettinen and Nurminen score test statistic (which is the basis for calculating the confidence interval used for analysis in this study). The first ignored interim monitoring but used a binomial enumeration method to calculate power and type I error rates. Use of the binomial enumeration method takes account of the discreteness of the binomial distribution (rather than using a normal approximation to the binomial distribution) which may be important in the setting of low hospitalization/death probabilities. Using this approach gave a power of 90.2%. The second approach did not use a binomial enumeration but took account of interim analyses using a standard implementation of four equally-spaced interim analyses using the O'Brien and Fleming stopping guideline. This used a simulation approach and gave a power of 90.0% (width of 95% confidence interval around this simulation-based value was 0.12%). Based on these two approaches, it is anticipated that the study will have close to 90% power to show non-inferiority for an infused investigational agent assuming that it truly has the same 2.3% hospitalization/death rate as the active comparator agent.

The PASS software was also used to illustrate how the power of the study might change for various scenarios which differ from the scenario assumed (see Table 2.4.3-1). This was undertaken using the first of the two approaches for evaluating prior mentioned above (i.e., using the binomial enumeration approach). Looking at the top part of the table in which both the infused investigational agent and the active comparator agent have the same underlying true hospitalization/ death rate, the power is decreased if the true rate is above the assumed 2.3%, but increased if the true rate is less than 2.3%. If the true rate is 3%, then the power is still above 80%, but if the true rate is 4% it is reduced to 73%.

The middle and lower parts of the table show scenarios in which the infused investigational agent has a true hospitalization/death rate of 0.5% or 1% worse than the active comparator agent, respectively. If the true rate for the active comparator agent is 2.3% and is 2.8% for the infused investigational agent (i.e., 0.5% worse), then the power is reduced to 73%. If the true rate for the active comparator agent is 2.3% and is 3.3% for the infused investigational agent (i.e., 1% worse), then the power is reduced to 50%

Table 2.4.3-1: Power for various scenarios based on non-inferiority hypothesis testing using the likelihood score test statistic (Miettinen and Nurminen method) with binomial enumeration of power and Type I error rate. All scenarios use a 3% non-inferiority margin and one-sided Type-I error rate of 0.025 with a sample size of 600 participants receiving an infused investigational agent and 600 participants receiving the active comparator agent. Power in practice will be slightly reduced from the values shown due to interim monitoring.

Same Underlying True Hospitalization/Death Rate For Active Comparator Agent and Infused Investigational Agent			
Power	True % Hosp/Died on Active Comparator Agent	True % Hosp/Died on Infused Investigational Agent	Actual Type I Error Rate*
99.4%	1%	1%	2.2%
97.3%	1.5%	1.5%	2.2%
93.2%	2%	2%	2.3%
90.2%	2.3%	2.3%	2.4%

Same Underlying True Hospitalization/Death Rate For Active Comparator Agent and Infused Investigational Agent			
Power	True % Hosp/Died on Active Comparator Agent	True % Hosp/Died on Infused Investigational Agent	Actual Type I Error Rate*
88.1%	2.5%	2.5%	2.4%
83.1%	3%	3%	2.4%
78.1%	3.5%	3.5%	2.4%
73.2%	4%	4%	2.4%
Infused Investigational Agent with Underlying True Hospitalization/Death Rate that is 0.5% Worse than Active Comparator Agent			
Power	True % Hosp/Died on Active Comparator Agent	True % Hosp/Died on Infused Investigational Agent	Actual Type I Error Rate*
92.5%	1%	1.5%	2.2%
85.2%	1.5%	2%	2.2%
77.4%	2%	2.5%	2.3%
73.1%	2.3%	2.8%	2.4%
70.5%	2.5%	3%	2.4%
64.8%	3%	3.5%	2.4%
59.6%	3.5%	4%	2.4%
55.0%	4%	4.5%	2.4%
Infused Investigational Agent with Underlying True Hospitalization/Death Rate that is 1% Worse than Active Comparator Agent			
Power	True % Hosp/Died on Active Comparator Agent	True % Hosp/Died on Infused Investigational Agent	Actual Type I Error Rate*
71.3%	1%	2%	2.2%
61.7%	1.5%	2.5%	2.2%
54.0%	2%	3%	2.3%
50.4%	2.3%	3.3%	2.4%
48.4%	2.5%	3.5%	2.4%
44.0%	3%	4%	2.4%
40.0%	3.5%	4.5%	2.4%
36.7%	4%	5%	2.4%
*Actual type I error rate is slightly lower than assumed rate of 2.5% because of discreteness of the binomial distribution.			

Sample Size Justification for Non-infused Investigational Agents

For the evaluation of a specific non-infused investigational agent, the sample size will include approximately 800 participants randomized to receive the non-infused investigational agent and approximately 800 participants (who were eligible to receive the non-infused investigational agent) concurrently randomized to receive the active comparator agent. This sample size has been chosen to provide very high power (approximately 96%) to establish non-inferiority assuming that the true proportion hospitalized/dead for both the non-infused investigational agent and the active comparator agent is 2.3%, while also providing high power (approximately 85%) assuming that the true proportion hospitalized/dead for the non-infused investigational agent is 0.5% worse, i.e., 2.8%, than the active comparator agent. The rationale for the 2.3% rate for the active comparator agent and for having no adjustment for loss to follow-up is the same as described above in justifying the sample size for infused investigational agents.

The potential power of the study for non-infused agents was evaluated in the same two ways as described above for infused investigational agents using the PASS version 15 sample size calculation software. Use of the binomial enumeration method not taking account of interim analyses gave a power of 96.6% if the non-infused investigational agent and active comparator agent had the same true rates of

hospitalization/death (2.3%), and 85.2% power if the non-infused investigational agent had a slightly lower true rate than the active comparator agent (2.8% versus 2.3%). The second (simulation-based) approach did not use a binomial enumeration but taking account of four equally-spaced interim analyses using the O'Brien and Fleming stopping guideline. This used a simulation approach and gave a power of 96.2% (width of 95% confidence interval around this simulation-based value was 0.08%) if the non-infused investigational agent and the active comparator agent has the same true rate of hospitalization/death (2.3%), and 84.2% power (width 0.15%) if the non-infused investigational agent had a slightly lower true rate than the active comparator agent (2.8% versus 2.3%).

Overview of Formal Interim Monitoring

During the course of the study (phase II and phase III), an independent NIAID-appointed Data and Safety Monitoring Board (DSMB) will undertake reviews of interim data from the study. The following sections outline plans for interim monitoring of the placebo-controlled phase II, the placebo-controlled phase III and the active-controlled phase III. Additional details on phase II monitoring can be found in protocol version 7.0 section 10.5, and in protocol version 7.0 Appendix V for placebo-controlled phase III monitoring. Details on active-controlled phase III monitoring are taken for protocol version 7.0 section 10.5.2 as amended in letter of amendment 1 to protocol version 7.0. Statistical considerations for interim monitoring are shown in section 5.4 of this SAP.

Regardless of study phase, in the event that there is any death deemed related to study product or if two participants experience a Grade 4 AE deemed related to study product, enrollment to the study product group will be paused and the DSMB will review interim safety data.

Phase II – Placebo-Controlled Superiority

During phase II, the DSMB will review interim data to ensure the safety of participants in the study, and to evaluate the activity of each investigational agent in order to provide graduation recommendations to the Trial Oversight Committee (TOC) via NIAID. The DSMB may recommend early termination of randomization to a particular investigational agent if there are safety concerns, but it is not intended to stop for futility in the phase II evaluation period.

For each investigational agent, there will be interim analyses of safety data by the DSMB approximately monthly (or on a schedule recommended by the DSMB) with the first review occurring approximately 6 weeks after enrollment to a given agent begins.

Phase III – Placebo-Controlled Superiority

During phase III, the DSMB will review interim data to help ensure the safety of participants in the study, and to recommend changes to the study. The DSMB may recommend termination or modification of the study for safety reasons, if there is persuasive evidence of efficacy or lack of efficacy of an investigational agent versus placebo in preventing hospitalizations and deaths, or on the basis of statistical or operational futility. At each interim review, the DSMB will review summaries of data by unblinded randomized arms for the primary outcome of hospitalization/death, the secondary outcome of death, losses to follow-up, and adverse events (including early discontinuation of the investigational agent).

For monitoring the primary efficacy outcome, the O'Brien Fleming boundary will be used as the stopping guideline, implemented using the Lan-DeMets spending function to allow for changes in the timing or number of interim analyses if recommended by the DSMB.

Three interim efficacy analyses are planned during phase III. The first review is planned at the completion of day 28 of follow-up for phase II participants, and second and third reviews are planned for after about 50% and 75% of the expected maximal efficacy (hospitalization/death) information.

The expected maximal efficacy information available at the planned interim analyses is approximately proportional to the expected number of hospitalizations/deaths under design assumption parameters. Assuming 15% of participants will be hospitalized/die in the placebo control group and 7.5% will be hospitalized/die in the investigational agent group (i.e., relative reduction of 50%), with 421 participants per group, this corresponds to 95 participants hospitalized/died across both groups. Because of the uncertainty around the design assumptions, interim efficacy analyses will occur as follows (unless DSMB recommends otherwise):

- The first interim analysis for phase III will be when 220 participants from the two groups combined have been followed for the primary outcome assessed at day 28 (this will likely then be the same hospitalization/death information used in the phase II graduation analysis), or when approximately 24 participants in the two groups combined have been hospitalized or have died;

- The earlier of when approximately 421 participants from the two groups combined (50% of the 842) have been followed for the primary outcome assessed at day 28, or when approximately 48 participants in the two groups combined have been hospitalized or have died;
- The earlier of when approximately 632 participants from the two groups combined have been followed for the primary outcome assessed at day 28, or when approximately 72 participants in the two groups combined have been hospitalized of have died.

In considering possible modifications to the study or termination of the study for efficacy, the DSMB may also consider interim results for the secondary outcome of death. The DSMB may make recommendations based on a high level of evidence for a difference between randomized arms, which might be based on application of the O'Brien and Fleming stopping guideline to the death outcome. In these circumstances, consideration should be given to the increased risk of a Type I error.

There is the possibility that differences between the randomized arms may be observed at an early study time point (for example, cumulative proportion at day 6); however, the overall goal of the study is to prevent hospitalization and deaths regardless of the timing, and therefore the focus of the randomized arm comparisons will be at day 28.

The DSMB will monitor for statistical futility (i.e., stopping early for the absence of difference between groups). An investigational agent may be discontinued based on evidence of lack of effect or very limited effect compared with placebo control. For the purpose of evaluating statistical futility, a moderately aggressive Type II error spending function, Gamma (-2) spending function implemented using the Lan-DeMets spending function approach, will be used.

The DSMB will also monitor operational futility. With respect to operational futility, the DSMB may recommend modification or termination of the study if the proportion hospitalized/die in the control group is much lower than expected in designing the trial. For example, the DSMB might recommend restricting or closing enrollment to the low-risk stratum in favor or increasing enrollment to the high-risk stratum. In addition, the DSMB will monitor the loss to follow-up (LTFU) rate. As a benchmark, an overall LTFU rate of more than 10% would be cause for concern.

Phase III – Active-Controlled Non-Inferiority

The DSMB will undertake reviews of interim data from the study to help ensure the safety of participants in the study, and to recommend changes to the study including termination or modification for safety reasons or if there is persuasive evidence of non-inferiority (or superiority or inferiority) of an investigational agent versus the active comparator agent in its effect on the hospitalization/death outcome. It is not intended, however, to terminate evaluation of an agent early for efficacy based on symptom outcome measures. The DSMB may also recommend termination or modification of the study if it appears futile on statistical or operational grounds to continue an investigational agent in the study as designed.

Unless otherwise recommended by the DSMB, three interim analyses for DSMB review are planned for each investigational agent, after approximately 25%, 50% and 75% of the planned enrollment for an investigational agent has been completed and followed through to day 28. At each interim review of an investigational agent, the DSMB will review summaries of data by randomized treatment arm for the primary outcome of hospitalization/death, the secondary outcome of death, losses to follow-up, and adverse events (including early discontinuation of investigational agent).

Decision Guidelines for Efficacy or Lack of Efficacy

The general approach for decision-making with respect to efficacy is based on evaluating a two-sided 95% confidence interval (adjusted for interim analyses) for the absolute difference (investigational agent minus active comparator agent) in the proportion of participants hospitalized or dead by day 28, relative to thresholds defining non-inferiority, superiority or inferiority of the investigational agent as follows (in the order given):

- The DSMB may recommend releasing results evaluating the effect of an investigational agent when both non-inferiority and superiority of that agent is established based on the confidence interval being entirely below 0% (i.e., supportive of a lower true proportion being hospitalized or dying on the investigational agent than the active comparator agent). If this occurs, consideration will need to be given to the ongoing appropriateness of the active comparator agent as a control for evaluating other investigational agents in the study.
- Early stopping and/or release of results based on non-inferiority should be considered on an agent-by-agent basis. For non-infused agents, the DSMB may recommend releasing results evaluating the effect of an investigational agent when non-inferiority (but not superiority) of that agent is established based on the confidence interval being entirely below 3% (but not entirely below 0%).

However, in the interests of also having an adequate safety database for the investigational agent, it is not intended that this recommendation be made before approximately 400 participants have been randomized to receive the agent (or some other number of participants specified in the agent-specific appendix). In addition, the study may continue randomizing participants to the investigational agent in the interests of increasing precision in evaluating the agent; this decision will be made by the study team and sponsor on an agent-by-agent basis. For infused agents, early stopping and/or release of results for non-inferiority should not be considered.

- The DSMB may recommend releasing results and terminating randomization to an investigational agent if inferiority of that agent is established based on the confidence interval being entirely above 0% (i.e., suggesting a higher true proportion being hospitalized or dying on the investigational agent than the active comparator agent). Examples of how this criterion might be met when evaluating an infused agent and when the observed control rate is close to 2.3% include observing 18/150 versus 3/150 (observed difference 10.0%) at the first interim analysis; 23/300 versus 7/300 (observed difference 5.3%) at the second interim analysis; and 24/450 versus 10/450 at the third interim analysis (observed difference 3.1%). In these examples, all observed differences are higher than the non-inferiority margin of 3%, and are indicative also of the futility of continuing evaluation of the infused investigational agent to demonstrate non-inferiority.

Graduation to Phase III

The following applies to investigational agents that have been not assessed for graduation to phase III under prior versions of the protocol (version 1.0 to 6.0); note BRII-196+BRII-198 was previously assessed for graduation to phase III under protocol versions 2.0 and 3.0 (clinical sites were enrolling participants under both versions at the time of the graduation analysis).

Each investigational agent that is being considered for evaluation in phase III will be evaluated for safety, for activity in reducing COVID-19 symptoms and hospitalization/death, and for activity in reducing SARS-CoV-2 RNA shedding. An analysis to determine if an agent should graduate from phase II, and enter phase III, will be conducted when 220 participants assigned to the agent or concurrent placebo in phase II evaluation have completed their Day 7 evaluations and have the required data available in the database. Additional interim graduations may be assessed for some agents, see agent-specific appendices in protocol version 7.0 for details.

The DSMB will review unblinded data and make recommendations to NIAID (as trial sponsor) and to the TOC, indicating whether graduation criteria have been met. The recommendation for an agent to enter phase III evaluation will be made by the TOC in discussion with the collaborating company; the collaborating company that is responsible for the agent will decide whether or not to adopt the recommendation. The TOC and collaborating company will also consider which dose to recommend for evaluation in phase III, for investigational agents with more than one dose under evaluation in phase II. NIAID/DAIDS, as the sponsor of the study, will make the final determination regarding graduation of the study product.

The TOC may recommend an agent move directly into phase III, without evaluation in phase II in ACTIV-2, if there is sufficient safety and efficacy data supporting phase III evaluation available from outside of the trial. These agents will not undergo graduation analyses.

Graduation criteria and statistical considerations are discussed in the Graduation Rules SAP.

Outcome Measures

All outcome measures are copied from the protocol version 7.0 (including letter of amendment 1). Only outcome measures addressed in this SAP are included below. See protocol section 10.2 for additional outcome measures.

Primary Outcome Measures: Phase III

1) <u>Safety</u>: New Grade 3 or higher AE through 28 days. [For Primary Objective 1]
   New Grade 3 or higher AE is defined as: Grade 3 or higher event that was new in onset or aggravated in severity or frequency from the baseline condition (i.e., Grade 1 or 2 at baseline escalates to Grade 3 or higher, or Grade 3 at baseline escalates to Grade 4 or higher), following the start of study treatment.
2) <u>Efficacy:</u> Death due to any cause or hospitalization due to any cause during the 28-day period from and including the day of the first dose of investigational agent or comparator intervention. [For Primary Objective 4]

Hospitalization is defined as ≥24 hours of acute care, in a hospital or similar acute care facility, including Emergency Rooms or temporary facilities instituted to address medical needs of those with severe COVID-19 during the COVID-19 pandemic.

Primary Outcome Measures: Phase II

1) Safety: New Grade 3 or higher AE through 28 days. [For Primary Objective 1]
   New Grade 3 or higher AE is defined as: Grade 3 or higher event that was new in onset or aggravated in severity or frequency from the baseline condition (i.e., Grade 1 or 2 at baseline escalates to Grade 3 or higher, or Grade 3 at baseline escalates to Grade 4 or higher), following the start of study treatment.
2) Clinical (Symptom Duration): Duration of targeted COVID-19 associated symptoms from start of investigational agent (day 0) based on self-assessment. [For Primary Objective 2]
   Duration defined as the number of days from start of investigational treatment to the first of two consecutive days when any symptoms scored as moderate or severe at study entry (pre-treatment) are scored as mild or absent, and any symptoms scored as mild or absent at study entry (pre-treatment) are scored as absent. The targeted symptoms are fever or feeling feverish, cough, shortness of breath or difficulty breathing at rest or with activity, sore throat, body pain or muscle pain/aches, fatigue (low energy), headache, chills, nasal obstruction or congestion (stuffy nose), nasal discharge (runny nose), nausea, vomiting, and diarrhea. Each symptom is scored daily by the participant as absent (score 0), mild (1), moderate (2) and severe (3).
3) Virologic: At each of days 3, 7 and 14 quantification (< LLoQ versus ≥ LLoQ) of SARS-CoV-2 RNA from staff-collected NP swabs.
   [For Primary Objective 3]

Secondary Outcome Measures

Safety

1) Phase II only: New Grade 2 or higher AE through 28 days.
   [Supportive of Primary Objective 1]
   New Grade 2 or higher AE is defined as: Grade 2 or higher event that was new in onset or aggravated in severity or frequency from the baseline condition (i.e., Grade 1 at baseline escalates to Grade 2 or higher, or Grade 2 at baseline escalates to Grade 3 or higher, or Grade 3 at baseline escalates to Grade 4 or higher), following the start of study treatment.
2) Phase II only: New Grade 2 or higher AE through week 24.
   [Supportive of Primary Objective 1, with follow-up beyond day 28]
   New Grade 2 or higher AE is defined as: Grade 2 or higher event that was new in onset or aggravated in severity or frequency from the baseline condition (i.e., Grade 1 at baseline escalates to Grade 2 or higher, or Grade 2 at baseline escalates to Grade 3 or higher, or Grade 3 at baseline escalates to Grade 4 or higher), following the start of study treatment.
3) Phase III only: New Grade 3 or higher AE through week 24.
   [Supportive of Primary Objective 1, with follow-up beyond day 28]
   New Grade 3 or higher AE is defined as: Grade 3 or higher event that was new in onset or aggravated in severity or frequency from the baseline condition (i.e., Grade 1 or 2 at baseline escalates to Grade 3 or higher, or Grade 3 at baseline escalates to Grade 4 or higher), following the start of study treatment.

Clinical Symptoms

4) Phase III only: Duration of targeted COVID-19 associated symptoms from start of investigational agent (day 0) through day 28 based on self-assessment.
   [Supportive of Primary Objective 2]
   Duration defined as the same as the primary phase II clinical (symptom duration) outcome.
5) Phase II and III: Duration of targeted COVID-19 associated symptoms from start of investigational agent (day 0) through day 28 based on self-assessment.
   [Supportive of Primary Objective 2 and for Secondary Objective 8]
   Duration defined as the number of days from start of investigational treatment to the first of four consecutive days when all symptoms are scored as absent. Targeted symptoms as defined in the primary phase II clinical (symptom duration) outcome.
6) Phase II and III: Time to self-reported return to usual (pre-COVID-19) health as recorded in a participant's study diary through day 28.
   [Supportive of Primary Objective 2]

Time to self-reported return to usual health defined as the number of days from start of investigational treatment until the first of two consecutive days that a participant reported return to usual (pre-COVID) health.

7) Phase II and III: Time to self-reported return to usual (pre-COVID-19) health as recorded in a participant's study diary through day 28.
[Supportive of Primary Objective 2]

Time to self-reported return to usual health defined as the number of days from start of investigational treatment until the first of four consecutive days that a participant reported return to usual (pre-COVID) health.

8) Phase II and III: COVID-19 severity ranking based on symptom severity scores over time during the 28-day period from and including the day of the first dose of investigational agent or comparator intervention, hospitalization, and death. [For Secondary Objective 1].
Participants who are alive at 28 days and not previously hospitalized, the severity ranking will be based on their area under the curve (AUC) of the daily total symptom score associated with COVID-19 disease over time (through 28 days counting day 0 as the first day) where the total symptom score on a given day is defined as the sum of scores for the targeted symptoms in the participant's study diary (each individual symptom is scored as absent (score 0), mild (1) moderate (2) and severe (3)). Participants who are hospitalized or who die during follow-up through 28 days will be ranked as worse than those alive and never hospitalized as follows (in worsening rank order): alive and not hospitalized at 28 days; hospitalized but alive at 28 days; and died at or before 28 days.
9) Phase II and III: Progression through day 28 of one or more COVID-19-associated symptoms to a worse status than recorded in the study diary at study entry, prior to start of investigational agent or comparator intervention. [For Secondary Objective 2]

10) Phase II only: Oxygen saturation (i.e., pulse oximeter measure) categorized as <96 versus $\geq 96\%$ through day 28. [For Secondary Objective 6]

11) Phase II only: Level (quantitative) of oxygen saturation (i.e., pulse oximeter measure) through day 28. [Supportive of Secondary Objective 6]

<u>Virology</u>
12) Phase III (Active-Controlled) only: Quantification (< LLoQ versus ≥ LLoQ) of SARS-CoV-2 RNA from staff-collected NP swabs at day 3. [Support of Primary Objective 3]

13) Phase II and III: Level (quantitative) of SARS-CoV-2 RNA from staff-collected NP swabs at days 3, 7, and 14 in phase II and at day 3 in phase III.8.
[For Secondary Objective 3]

14) Phase II only: Area under the curve and above the assay lower limit of quantification of quantitative SARS-CoV-2 RNA over time from staff-collected NP swabs at days 0, 3, 7, and 14.
[Supportive of both Primary Objective 3 and Secondary Objective 3]
<u>Efficacy</u>
15) Phase II only: Death due to any cause or hospitalization due to any cause during the 28-day period from and including the day of the first dose of investigational agent or comparator intervention.
[Supportive of Primary Objective 4]
Hospitalization is defined as the same as the primary phase III outcome.
16) Phase II and III: Death due to any cause during the 28-day period from and including the day of the first dose of investigational agent or comparator intervention. [Supportive of Primary Objective 4]

17) Phase II and III: Death due to any cause or hospitalization due to any cause during the 24-week period from and including the day of the first dose of investigational agent or comparator intervention. [For Secondary Objective 8, with follow-up beyond day 28]
Hospitalization is defined as the same as the primary phase III outcome.
18) Phase II and III: Death due to any cause or hospitalization due to any cause during the 72-week period from and including the day of the first dose of investigational agent or comparator intervention. [For Secondary Objective 8, with follow-up beyond day 28]
Hospitalization is defined as the same as the primary phase III outcome.
19) Phase II and III: Death due to any cause during the 24-week period from and including the day of the first dose of investigational agent or comparator intervention.
[Supportive of Secondary Objective 8, with follow-up beyond day 28]

20) Phase II and III: Death due to any cause during the 72-week period from and including the day of the first dose of investigational agent or comparator intervention.
[Supportive of Secondary Objective 8, with follow-up beyond day 28]

21) Phase III: Death due to any cause or hospitalization due to any cause, excluding hospitalizations that are deemed unrelated to COVID-19, during the 28-day period from and including the day of the first dose of investigational agent or comparator intervention.
[For Secondary Objective 9]
Hospitalization is defined as ≥24 hours of acute care, in a hospital or similar acute care facility, including Emergency Rooms or temporary facilities instituted to address medical needs of those with severe COVID-19 during the COVID-19 pandemic.

Other Outcome Measures

1) Phase II and III: New SARS-CoV-2 positivity among household contacts through to 28 days from start of investigational agent or comparator intervention. [For Exploratory Objective 1]

2) Phase II and III: New SARS-CoV-2 positivity or COVID-19 symptoms among household contacts through to 28 days from start of investigational agent or comparator intervention. [Supportive of Exploratory Objective 1]

3) Phase II and III: New SARS-CoV-2 positivity among household contacts through to 24 weeks from start of investigational agent or comparator intervention. [For Exploratory Objective 1, with follow-up beyond day 28]

4) Phase II and III: New SARS-CoV-2 positivity or COVID-19 symptoms among household contacts through to 24 weeks from start of investigational agent or comparator intervention. [Supportive of Exploratory Objective 1, with follow-up beyond day 28]

5) Phase II and III: Worst clinical status assessed using ordinal scale among participants who become hospitalized through day 28. [For Exploratory Objective 4]
Ordinal scale defined as:
    Death
    Hospitalized, on invasive mechanical ventilation or ECMO;
    Hospitalized, on non-invasive ventilation or high flow oxygen devices;
    Hospitalized, requiring supplemental oxygen;
    Hospitalized, not requiring supplemental oxygen (COVID-19 related or otherwise).

6) Phase II and III: Duration of hospital stay among participants who become hospitalized through day 28. [For Exploratory Objective 4]

7) Phase II and III: ICU admission (yes versus no) among participants who become hospitalized through day 28. [For Exploratory Objective 4]

8) Phase II and III: Duration of ICU admission among participants who are admitted to the ICU through day 28. [For Exploratory Objective 4]

9) Phase II and III: Worst clinical status assessed using ordinal scale among participants who become hospitalized through week 24.
[For Exploratory Objective 4, with follow-up beyond day 28]

Ordinal scale defined as:
Death
Hospitalized, on invasive mechanical ventilation or ECMO;
Hospitalized, on non-invasive ventilation or high flow oxygen devices;
Hospitalized, requiring supplemental oxygen;
Hospitalized, not requiring supplemental oxygen (COVID-19 related or otherwise).

10) Phase II and III: Duration of hospital stay among participants who become hospitalized through week 24.
[For Exploratory Objective 4, with follow-up beyond day 28]

11) Phase II and III: ICU admission (yes versus no) among participants who become hospitalized through week 24. [For Exploratory Objective 4, with follow-up beyond day 28]

12) Phase II and III: Duration of ICU admission among participants who are admitted to the ICU through week 24. [For Exploratory Objective 4, with follow-up beyond day 28]

Statistical Principles

General Considerations

The following analysis populations are defined for a given investigational agent:

- Screened Population: All participants who were screened for enrollment into the study, between the time of screening of the first and last participants who were eligible to be randomized to the given Investigational Agent Group.

- Randomized Population: All participants who were enrolled and were eligible to be randomized to the given Investigational Agent Group, and were actually randomized either to the investigational agent or to its comparator intervention (placebo or active comparator, as appropriate for the agent and phase of evaluation).

- Treated Population: All participants in the Randomized Population who received any investigational agent or its comparator agent (this is a modified intent-to-treat [mITT] population).

In general, the Treated Population is the focus of randomized comparisons to evaluate the safety and efficacy outcomes of an investigational agent versus its comparator intervention. In all analyses of a given investigational agent, the comparison group will include all participants who were concurrently randomized to the comparator intervention, who were also eligible to have received the investigational agent of interest. For the placebo-controlled trials, the comparison group will pool across all relevant placebos (i.e. including the placebo for the agent of interest and the placebos for other agents). For the primary placebo-controlled analysis of a specific investigational agent, a supplemental analysis may be undertaken that restricts the comparison group to include only participants who received the placebo for that specific investigational agent.

Study visit windows for reporting are based on the Schedule of Evaluations (SOE) defined in the protocol (in person visits shown in the below table) and will be derived based on the evaluation/specimen date and study treatment initiation date (at interim analyses, if not available, study start date will be used). In the event that multiple results fall within the same analysis window, the one closest to the target time point will be prioritized, or if equidistant from the target time point, the earlier result will be prioritized. For interim analyses, if a result does not fall in an analysis window, the visit label will be used to identify the target time point.

SOE Visit	Protocol Range (Days)	Analysis Range (Days)	Analysis Window (Days)
Screening	-2, 0	-10, 0	-10, 0
Day 0*	0	-1, 0	-1, 0
Day 3	2, 4	1, 4	-2, +1
Day 7	5, 9	5, 10	-2, +3
Day 14	12, 16	11, 21	-3, +7
Day 28	28, 32	22, 38	-6, +10
Week 12	77, 91	56, 112	+/- 28
Week 24	161, 175	140, 196	+/- 28
Week 36	245, 266	224, 280	+/- 28
Week 48	329, 350	308. 364	+/- 28
Week 72	497, 518	476, 532	+/- 28

*The Day 0 analysis window is designed to capture data in scenarios where randomization occurs on the day prior to treatment initiation. Evaluations that occur on Day 0, post-treatment initiation (e.g., vital signs evaluations), will consider the time of the evaluation compared to the time of treatment administration (and will be presented as 'Day 0' with the relative time). Windows cited above do not apply to data with daily collections (i.e., diary cards or nasal swabs).

Key study visits are Entry (Day 0), day 28, week 24:

Entry (Day 0):	First dose of investigational agent/comparator intervention occurs. Baseline is defined as the last available measure prior to the initiation of investigational agent/placebo.
Day X:	Last day of investigational agent/comparator intervention. Value of X depends on agent: see protocol appendices for details for each specific investigational agents.
Day 28:	Last day primary outcome may occur.
Week 24:	Key visit for evaluating longer-term outcomes for all agents (note: some agents may have follow-up beyond week 24).
Week 72:	Key visit for evaluation longer-term efficacy and safety for some agents (see agent specific appendices).

Statistical comparison across randomized arms of baseline characteristics are not planned because the study is randomized; hence, any differences should reflect chance variation. In addition, comparisons between investigational agents are not planned. Control of the Type I error rate will be undertaken separately for each investigational agent, and not across all investigational agents (i.e., not for the experiment-wise or family-wise error rate of the study).

Analyses of primary and secondary outcomes will not adjust for multiple comparisons. Analyses of primary outcomes will adjust for the multiple interim reviews using group sequential methods.

Continuous variables will be summarized using mean, standard deviation, median, interquartile range (Q1 and Q3), 10th and 90th percentile, and min and max; categorical variables will be summarized using frequency and percentage.

NIH requires that the primary outcomes also be summarized by randomized arm by sex/gender and by race/ethnicity, and that treatment interactions with sex/gender and race/ethnicity be evaluated.

SARS-CoV-2 RNA results may be below the assay lower limit of quantification (LLoQ) or above the upper limit of quantification (ULoQ). Values below the LLoQ or above the ULoQ will generally be considered as censored observations in statistical analyses (with left censoring at the LLoQ and right censoring at the ULoQ, respectively). However, if necessary for any analyses (and for graphical presentations), values may be imputed in the following manner:

- Values below the LLoQ, but above the limit of detection (LoD) will be imputed as half the distance from the log-10 transformed LoD to the log-10 transformed LLoQ
- Values below the LLOQ and below the LoD will be imputed as half the distance from zero to the log-10 transformed LoD;
- Values above the ULoQ will be imputed as one unit higher than the log-10 transformed ULoQ; actual values obtained from assay reruns with dilution will be used instead, if available.

Virology results generated from specimens with the following conditions reported in the database will be excluded from analyses:

- Thawed

- Invalid Specimen
- Quantity Not Sufficient
- Destroyed

NOTE: Samples with the condition code 'NOT' were also to be excluded per the trial sponsor but this code indicates that the specimen was not tested. Thus, no result is expected and no exclusion is needed.

Analysis Approaches

All analyses addressing the primary and secondary objectives will include all randomized participants who started an investigational agent or the concurrent comparator intervention, according to a modified intent-to-treat (mITT) approach, i.e. using the Treated Population. Note that according to the protocol, participants who are randomized but do not start investigational agent or comparator intervention are not followed. Participants who have protocol violations, such as those who start investigational agent or comparator intervention outside of the protocol-defined study windows, or who are found to be ineligible, will be included in the analysis on the basis that they were considered part of the target population at the time of randomization and start of treatment.

For agents in phase II evaluation, participants who were at "higher" risk of progression to severe COVID-19 when eligible and enrolled under an earlier version of the protocol will be included in all analyses. Similarly, participants who were eligible and enrolled with longer than 7 days from symptom onset to study entry will be included in all analyses.

Analyses of the Primary Objectives

5.1.2 Phase III Primary Objective for Efficacy: Placebo-Controlled Superiority Evaluation

The following table summarizes the primary efficacy objective in phase III and the associated estimand under the placebo-controlled superiority design. Further details are provided after the table.

<table>
<tr><th colspan="3">Phase III Primary Objective for Efficacy—Placebo-Controlled Superiority Evaluation: To determine if the investigational agent will prevent the composite endpoint of either hospitalization due to any cause or death due to any cause through study day 28.</th></tr>
<tr><td>Estimand description</td><td colspan="2">Ratio (for investigational agent divided by placebo group) of cumulative probability of death or hospitalization through day 28, among adults with documented positive SARS-CoV-2 molecular test results collected within 240 hours (10 days) prior to study entry with no more than 10** days of symptoms of COVID-19 prior to study entry, and with presence of select symptoms within 24 hours of study entry.</td></tr>
<tr><td>Treatment</td><td colspan="2">Investigational agent or placebo.</td></tr>
<tr><td colspan="2">Target population</td><td>Analysis set (analysis population)</td></tr>
<tr><td colspan="2">Adults (≥ 18 years of age) with documented positive SARS-CoV-2 molecular test results collected within 240 hours (10 days) prior to study entry with no more than 10** days of symptoms of COVID-19 prior to study entry, and with presence of select symptoms within 24 hours of study entry</td><td>Treated Population</td></tr>
<tr><td colspan="2">Variable(s)</td><td>Outcome measure(s)</td></tr>
<tr><td colspan="2">Indicator variable for death due to any cause or hospitalization due to any cause during the 28-day period from and including the day of the first dose of investigational agent or placebo (coded as 1 if participant died or was hospitalized, and 0 otherwise).

To handle censoring due to loss to follow-up before 28 days in statistical analysis, a time variable for study</td><td>Death due any cause or hospitalization due to any cause during the 28-day period from and including the day of the first dose of investigational agent or placebo.</td></tr>
</table>

day of hospitalization/ death or censoring (earlier of 28 days or day of last contact with participant) is also needed.	
Handling of intercurrent events	Handling of missing data
None. A treatment policy strategy is being taken to evaluate treatment effects irrespective of intercurrent events (e.g. irrespective of whether a participant received the complete dose(s) of an agent/placebo).	Participants who discontinued follow-up before day 28 without previously dying or being hospitalized will be considered as (non-informatively) censored at the date last known to be alive.
Population-level summary measure	Analysis approach
Ratio (for investigational agent divided by placebo group) of cumulative probability of death or hospitalization over 28 days.	Ratio (for investigational agent divided by placebo group) of the cumulative proportion dying or being hospitalized at day 28 obtained using Kaplan-Meier estimation using the indicator variable for hospitalization/death and the time variable described above. See text for further details.
* * This was changed from 10 days under protocol version 2 and protocol version 3, to 8 days under LOA#1 to protocol version 3, (also applies to protocol version 4 and 5).	

Analysis Approach

The analysis of the primary efficacy outcome in phase III will compare the cumulative proportion of participants hospitalized or died (due to any cause), from day 0 through day 28, between randomized arms using a ratio of proportions; hospitalizations that begin on day 28 and deaths that occur on day 28 will be included. The cumulative proportion will be estimated for each randomized arm using Kaplan-Meier methods to account for losses to follow up (and differential follow-up at the interim reviews). For analysis purposes, the integer scale will be used as the time scale, where study day 0 is the day of start of investigational agent or placebo, study day 1 is considered day 1, and study day 28 is considered day 28; if an event occurs on day 0 then event time will be set to 0.5 for analysis. Participants will have follow-up censored at the date they were last known to be alive and not hospitalized through day 28. The primary analysis assumes non-informative censoring.

The absolute difference in the estimated log-cumulative proportion will be calculated between randomized arms; a 95% CI will be obtained for this difference in log-cumulative proportion calculated using a variance for this difference being the sum of the variances for each randomized arm obtained using Greenwood's formula. Results will be anti-logged to give the estimated ratio of cumulative proportions through day 28 (investigational agent vs placebo) and associated 95% CI. Two-sided 95% confidence intervals (CIs) and p-value (for the test of no difference between groups) will be obtained, which adjust for the interim analyses; a nominal 95% CI and p-value will also be provided.

It is possible, particularly at interim analyses for DSMB reviews, that the number of hospitalizations/deaths in an arm (investigational agent or placebo) will be very small and hence the asymptotic (large sample size) statistical theory underpinning the above statistical analyses may be questionable. To address this, using a standard rule of thumb, if there are fewer than 5 events (hospitalizations/deaths) in either arm, inference based on Fisher's exact test to compare arms will be adopted instead of using Greenwood's formula to calculate confidence intervals for the difference between arms and associated p-values. If there are zero events in both arms, then this will be stated and no formal statistical inferential analyses will be undertaken.

Sensitivity Analyses

The following sensitivity analyses are included to evaluate impact of different assumptions on the inference of the primary comparisons. The third sensitivity analysis is an exploratory analysis.

1) Evaluate the composite outcome of being hospitalized, dead, or loss-to-follow-up.
   Approach: Repeat the primary analysis, but assume all participants who prematurely discontinued study follow-up prior to day 28 and who were unable to be contacted by the site to ascertain outcomes after discontinuation, had a primary event at day 28. See sensitivity analysis number 3 below for evaluating the potential impact of differential loss to follow-up.
2) Evaluate the impact of participants enrolling from the same household.
   Approach: Repeat the primary analysis only including the first participant who enrolled from each household.

In the event that interpretation of results for the primary analysis differs substantially from the results from this sensitivity analysis, analysis methods that account for clustering will be considered, if feasible.

3) Exploratory: Evaluate the impact of differential loss-to-follow-up (LTFU).

Approach: In the event that interpretation of the results for the primary analysis differs substantially between the primary analysis and the first sensitivity analysis, the impact of participants being LTFU will be explored using IPCW potentially using both pre-treatment variables and variables after starting study treatment to determine weights. The primary analysis will be repeated but, within each group, participants who are not LTFU will be weighted using IPCW determined by baseline variables that predict LTFU.

Supportive Analyses

Secondary Outcome 16 is included as supportive to the primary efficacy outcome. The cumulative proportion of participants dead (due to any cause) by day 28 will be analyzed in the same manner as the primary outcome.

Secondary Outcomes 17,18, 19, 20 and 21, , evaluate the proportion of participants who are hospitalized or died through week 24, the proportion who are hospitalized or died through week 72, the proportion who died (due to any cause) through week 24, the proportion who died (due to any cause) through week 72, and the proportion who died or were hospitalized excluding hospitalizations deemed unrelated to COVID-19 though day 28. These outcomes will be analyzed in the same manner as the primary efficacy outcome. In these analyses, however, participants will have their follow-up censored at the date they were last known to be alive and not hospitalized (or date they were last known to be alive) through 168 days (i.e. 24 times 7 days) or through 504 days (i.e. 72 times 7 days).

Secondary outcome 15 is included to assess the phase III primary efficacy outcome of hospitalization or death during phase II. This outcome will be analyzed in the same manner as the primary efficacy outcome in phase III if there are 5 or more participants who died or were hospitalized in each arm. If not, the number of deaths and hospitalizations will be summarized and compared between arms using Fisher's exact test.

Subgroup Analyses

To evaluate the effect of the investigational agent in specific populations, the primary outcome will be assessed among different subgroups. The same approaches outlined for the primary analysis will be implemented for each subgroup. Within each subgroup, the difference between randomized arms in the log-proportion will be estimated, and compared between subgroups by constructing a test of interaction and 95% confidence interval. This will be implemented by determining the difference between subgroups of the differences between randomized arms, and the variance of the difference will be determined by summing the variance of the subgroup-specific variances. In the event that the number of events in a subgroup in either the investigational arm or placebo arm is low (less than 5), descriptive summaries of the number of hospitalizations and deaths by subgroup and arm will be provided. Pre-specified subgroups of interest include:

1) Sex (Male sex at birth, female sex at birth)
2) Race (white, non-white)
3) Ethnicity (Hispanic, non-Hispanic)
4) Age Group (<60, ≥ 60)
5) Calendar days from first symptom associated with COVID-19 to start of investigational agent/placebo Stratification ($\leq$ 5 days, $>$ 5 days)
6) Site (if applicable) or site location (if applicable)

   Subgroup analyses by site will be considered if there are a limited number of sites that contributed to enrollment. Otherwise, subgroup analyses by site location (e.g. by country or region) will be conducted if non-US sites contribute to enrollment.

5.1.2 Phase III Primary Objective for Efficacy: Active-Controlled Non-Inferiority Evaluation

The following table summarizes the primary efficacy objective in phase III and the associated estimand under the active-controlled non-inferiority design. Further details are provided after the table.

**Phase III Primary Objective for Efficacy—Active-Controlled Non-Inferiority Evaluation:** To determine if the investigational agent will prevent the composite endpoint of either hospitalization due to any cause or death due to any cause through study day 28.	
Estimand description	Difference (for investigational agent minus active comparator agent) of probability of death or hospitalization through day 28, among adults with documented positive SARS-CoV-2 molecular test results collected within 240 hours (10 days) prior to study entry with no more than 7 days of symptoms of COVID-19 prior to study entry, and with presence of select symptoms within 24 hours of study entry.
Treatment	Investigational agent or active comparator agent (casirivimab and imdevimab).
Target population	Analysis set (analysis population)
Adults (≥ 18 years of age) with documented positive SARS-CoV-2 molecular test results collected within 240 hours (10 days) prior to study entry with no more than 7 days of symptoms of COVID-19 prior to study entry, and with presence of select symptoms within 24 hours of study entry	Treated Population
Variable(s)	Outcome measure(s)
Indicator variable for death due to any cause or hospitalization due to any cause during the 28-day period from and including the day of the first dose of investigational agent or active comparator agent (coded as 1 if participant died or was hospitalized, and 0 otherwise).	Death due any cause or hospitalization due to any cause during the 28-day period from and including the day of the first dose of investigational agent or active comparator agent.
Handling of intercurrent events	Handling of missing data
None. A treatment policy strategy is being taken to evaluate treatment effects irrespective of intercurrent events (e.g. irrespective of whether a participant received the complete dose(s) of the agent to which they were randomized.	Participants who discontinued follow-up before day 28 without previously dying or being hospitalized will be considered as not having an event after the date last known to be alive.
Population-level summary measure	Analysis approach
Difference (for investigational agent minus active comparator agent) of probability of death or hospitalization over 28 days.	Difference (for investigational agent minus active comparator agent) of the proportion dying or being hospitalized at day 28. See text for further details.

<u>Analysis Approach</u>

The analysis of the primary efficacy outcome in phase III will evaluate the absolute difference in proportion of participants hospitalized (due to any cause) or died (due to any cause), from day 0 through day 28, between randomized arms; hospitalizations that begin on day 28 and deaths that occur on day 28 will be included.

Inference will be based on constructing a two-sided exact 95% confidence interval for the absolute difference in proportions (proportion for the investigational agent minus the proportion for the active comparator agent). If this confidence interval is entirely below the non-inferiority margin of 3%, then a conclusion of non-inferiority of the investigational agent compared with the active comparator agent will provide reasonable evidence that the investigational agent is effective against COVID-19.

The exact 95% confidence interval will be calculated using the method of Chan and Zhang [Biometrics 1999;55:1201-09] as implemented, for example, in StatXact PROC BINOMIAL for SAS [StatXact 12 PROCs for SAS Users Manual. Cytel Inc., Cambridge, MA; 2019]. This method inverts two one-sided hypothesis tests (with one-sided error rate of 0.025 each) to obtain the confidence interval so providing a confidence interval-based method which preserves the type I error rate in establishing non-inferiority to be 0.025. To preserve confidence interval coverage (and type I error rate for assessing non-inferiority) over multiple interim analyses, the confidence interval will be calculated using a “repeated” confidence interval

approach with spending of error rate at each interim analysis using the Land and DeMets approach with an O'Brien and Fleming spending function.

In essence, basing the comparison of treatment groups on the simple proportion of participants who were hospitalized or died assumes that participants who are lost to follow-up before 28 days without prior hospitalization were not hospitalized and did not die by 28 days. The decision to use the simple proportion for analysis rather than use, for example, a Kaplan-Meier estimate of the cumulative proportion of participants hospitalized or dying during the first 28 days of follow-up to account for losses to follow-up was taken for multiple reasons. First, in ACTIV-2 and other COVID-19 trials, most hospitalizations and deaths occur during the first two weeks of follow-up and the study has been designed to have regular contact with participants or their secondary contacts so as to maximize ascertainment of hospitalization and death information. Second, loss to follow-up has been low in the ACTIV-2 study: approximately 3% among higher risk participants. Third, with the very low rates of hospitalization/death expected (e.g., 2.3% for the active comparator agent), confidence interval coverage (and type I error rates) are better preserved at their desired levels through the use of exact statistical methods for analyzing proportions than is achieved using asymptotic statistical methods based on Wald-type analyses using Greenwood's formula to obtain standard errors for Kaplan-Meier estimates. To assess the potential impact of loss to follow-up (assumed to be non-informative) on the interpretation of results, the following sensitivity analyses will be undertaken, repeating the primary analysis repeated with:

(a) a comparison of the simple proportions using a Wald-based confidence interval; and

(b) a comparison of proportions estimated using Kaplan-Meier methods (with censoring of follow-up at the earlier of day 28 and the time that a participant was last known to be alive) using a Wald-based confidence interval with standard error based on Greenwood's formula.

Supportive Analyses

Secondary Outcome 16 is included as supportive to the primary efficacy outcome. The cumulative proportion of participants dead (due to any cause) by day 28 will be analyzed in the same manner as the primary outcome.

Secondary Outcomes 16, 17,18, 19, 20 and 21 evaluate the proportion of participants who die through to day 28, the proportion who are hospitalized or died through week 24, the proportion who are hospitalized or died through week 72, the proportion who died (due to any cause) through week 24, the proportion who died (due to any cause) through week 72, and the proportion who died or were hospitalized excluding hospitalizations deemed unrelated to COVID-19 though day 28. These outcomes will be analyzed in the same manner as the primary efficacy outcome. In the sensitivity analyses based on Kaplan-Meier estimates, however, participants will have their follow-up censored at the date they were last known to be alive and not hospitalized (or date they were last known to be alive) through 168 days (i.e. 24 times 7 days for outcomes through to 24 weeks) or through 504 days (i.e. 72 times 7 days for outcomes through to 72 weeks).

Subgroup Analyses

To evaluate the effect of the investigational agent in specific populations, the primary outcome will be assessed among different subgroups. The same approach outlined for the primary analysis will be implemented for each subgroup. However, these analyses are likely to involve small numbers of events in most or all subgroups and hence have very limited precision. Because of this, any assessment of treatment by subgroup interaction, if undertaken, will be considered exploratory. Pre-specified subgroups of interest include:

1) Sex (Male sex at birth, female sex at birth)
2) Race (white, non-white)
3) Ethnicity (Hispanic, non-Hispanic)
4) Age Group (<60, ≥60)
5) Calendar days from first symptom associated with COVID-19 to start of investigational agent/active comparator Stratification (≤ 5 days, > 5 days)
6) Country (U.S., non-U.S.)
7) SARS-CoV-2 Variant (if available: categories will be determined based on prevalence of variants identified in testing).

1.1.2 Primary Safety (Phase II and III)

Analysis Approaches

Occurrence of any new Grade 3 or higher AE through 28 days will be analyzed in the following manner. The proportion of participants who experienced a new Grade 3 or higher AE will be estimated and

compared between randomized arms using log-binomial regression, with log link, in order to obtain a risk ratio estimate; the model will include a main effect for randomized arm. A 95% confidence interval for the risk ratio and a two-sided p-value from a Wald test of the null hypothesis that the risk ratio is one will also be provided. In the event the log-binomial regression model fails to converge or has questionable convergence, a Poisson regression model with robust variance and log-link will be used instead.

In addition, the absolute difference in proportion of participants who experienced a new Grade 3 or higher AE (or new Grade 2 or higher AE) will be calculated, with associated 95% confidence interval (calculated using the normal approximation to the binomial distribution).

It is possible, particularly at interim analyses for DSMB reviews, that the number of Grade 3 or higher AEs in an arm (investigational agent or comparator intervention) will be very small and hence the asymptotic (large sample size) statistical theory underpinning the above statistical analyses may be questionable. To address this, using a standard rule of thumb, if there are fewer than 5 events in either arm, inference based on Fisher's exact test to compare proportion between arms will be adopted instead of using the log-binomial regression model and normal approximation to the binomial distribution to calculate confidence intervals for the relative and absolute differences between arms and associated p-values. If there are zero events in both arms, then this will be stated and no formal statistical inferential analyses will be undertaken.

<u>Sensitivity Analyses</u>

In placebo-controlled evaluations, because some agents may be administered using injections or infusions and others will not be, the primary safety analysis may be repeated on the subset of the Treated Population that received the investigational agent of interest or the placebo for that specific agent.

<u>Supportive Analyses</u>

Secondary Outcome 1 is included as supportive to the primary safety outcome in phase II. This outcome evaluates the occurrence of new Grade 2 or higher AEs through 28 days, and will be analyzed in the same manner as the primary outcome.

Secondary Outcomes 2 and 3, which are included in support of the primary safety objective, evaluate the occurrence of new Grade 2 or higher AEs (in phase II) and Grade 3 or higher AEs (in phase III) through week 24. These outcomes will be analyzed in the same manner as the primary safety outcomes.

Additional longer-term safety outcomes may be assessed, see agent-specific appendices for details.

Primary Clinical Symptoms (Phase II)

<u>Analysis Approaches</u>

The targeted symptoms considered in evaluating the primary symptom outcome are: feeling feverish, cough, shortness of breath or difficulty breathing at rest or with activity, sore throat, body pain or muscle pain/aches, fatigue (low energy), headache, chills, nasal obstruction or congestion (stuffy nose), nasal discharge (runny nose), nausea, vomiting, and diarrhea. Each of these symptoms is scored daily in a study diary by the participant as absent, mild, moderate or severe from day 0 (pre-treatment) through day 28.

The primary symptom outcome measure is the time to when all targeted symptoms are sufficiently improved or resolved for two consecutive days from their status at day 0 (pre-treatment). Specifically, it is defined as the number of days from start of investigational agent (day 0, pre-treatment) to the first of two consecutive days when all symptoms scored as moderate or severe at day 0 (pre-treatment) are scored as mild or absent, AND all symptoms scored as mild or absent at day 0 (pre-treatment) are scored as absent.

Statistically, this is a time-to-event (TTE) variable, potentially involving censoring due to loss-to- follow-up or if a participant did not meet the outcome criteria for symptoms sufficiently improved/resolved during the 28 days of completing the diary. Censoring of follow-up for the TTE outcome measure will occur on the last day that the TTE outcome measure could have been achieved. Specifically, as two consecutive days of symptoms meeting the outcome measure criteria are required, censoring would be on the day before the last day of completion of the diary card (e.g., this would be day 27 for participants with complete diaries through day 28, as meeting the criteria requires completion of the diary on both day 27 and day 28).

Descriptive analyses for this TTE outcome measure will be undertaken using Kaplan-Meier methods including "survival" functions and/or cumulative incidence plots, and associated summary statistics (median [quartiles] with 95% confidence interval; and estimated % not meeting outcome measure criteria by 28 days with a 95% confidence interval). Comparison of the distribution of the TTE outcome measure between investigational agent and comparator intervention arms will be undertaken using Wilcoxon's test adapted for handling censored data (the Gehan-Wilcoxon test) using a two-sided Type-I error rate of 5%.

For each participant, the symptom data that contribute to the calculation of the TTE outcome measure and the censoring time (and associated censoring indicator variable) can be described as a panel of evaluations (absent/mild/moderate/severe) for each of 13 targeted symptoms on each of 29 days (day 0 through day

28). The following general principles will be applied for the handling of deaths, hospitalizations, and missing data:

- **Deaths**. Participants who die without previously achieving the TTE outcome (i.e. without two consecutive days of symptoms improved/resolved), will be retained in the risk set for the TTE outcome, but without achieving the TTE outcome, from the day of death (or the day after death if the diary was completed on the day of death) through to and including study day 27. Retention in the risk set through to 27 days provides for appropriate estimation of the cumulative proportion of the Treated Population who had a good outcome, i.e. symptoms improved/resolved for two consecutive days, over time.

- **Hospitalizations.** Participants who are hospitalized without previously achieving the TTE outcome measure will be retained in the risk set for the TTE outcome, but without having the TTE outcome, from all days hospitalized (including day of admission if no diary was completed that day, and including day of discharge if no diary was completed that day). As the protocol does not expect that diaries are completed during hospitalization, diary evaluations that are completed from the day after admission to the day before discharge will be ignored. The underlying premise is that participants have not achieved symptom improvement/resolution while hospitalized.

- **Losses to Follow-up and Early Termination of Evaluation of Targeted Symptoms.** Participants who are lost to follow-up or who terminate providing evaluations of the targeted symptoms in their study diaries before day 28 for any reason have monotonic missing data (i.e. a sequence of missing values during follow-up through to and including day 28). For these participants, the TTE outcome measure will be censored at the last day that the relevant criterion for symptom improvement could have been met (this would be the day before the last diary entry for one or more targeted symptoms). For the special case of participants who have no evaluations of targeted symptoms in their study diaries from the day of hospital discharge through to day 28 for any reasons, the TTE outcome measure will be censored at the day before discharge. If the participant withdraws from the study while hospitalized and therefore no date of discharge is available, then the TTE outcome measure will be censored on the day before withdrawal from the study. These criteria for censoring assume that the censoring is non-informative about when the TTE outcome would have been met if diaries had been fully completed after the last diary entry for one or more targeted symptoms (or after hospitalization or after withdrawal from the study during hospitalization).

- **Intermittent Missingness**. Participants who have intermittent missing evaluations for a specific symptom (i.e. one or more successive evaluations with preceding and succeeding evaluations for the same symptom) will have the missing evaluation(s) imputed as the worst of the preceding and succeeding evaluations for the same symptom. There may be no impact of this on the TTE outcome if evaluations of other symptoms are completed and do not meet the TTE outcome during the period of missingness for the specific symptom. If there is an impact, it may be to move the TTE outcome earlier (than if the evaluations had been done) if both the preceding and succeeding evaluations for the specific symptom meet the criteria for improvement/ resolution; and, conversely, to move the TTE outcome later (than if the evaluations had been done), if both the preceding and succeeding evaluations for the specific symptom don't meet the criteria for improvement/resolution.

- **Missing Day 0 Evaluation.** If the evaluation at day 0 is missing for a given symptom and there is at least one evaluation provided for that same symptom during follow-up, then the missing evaluations at day 0 and subsequently through to the first evaluation will be imputed as "mild". The choice of imputation as "mild" is based on the fact that among early participants in ACTIV-2, the median evaluation given to any specific symptom at day 0 was "mild". This imputation means that the improvement/resolution criteria cannot be met based on these imputed data (as the criteria for a mild symptom at day requires resolution to absent). The impact of this may be to move the TTE outcome later (than if the evaluations had been done) if the true day 0 evaluation would have been "absent" or "mild"; and it may also move the TTE outcome later (than if the

evaluations had been done) if the true day 0 evaluation would have been "moderate" or "severe" as the imputed "mild" symptom at day 0 must resolve to absent whereas a true "moderate" or "severe" symptom only need to resolve to "mild".

Appendix 1 includes a detailed description of an algorithm for handling missing data following these general principles that can be implemented programmatically.

Supportive Analysis

The analysis will be repeated using the same approach as described above (including handling of deaths, hospitalizations and missing data) for a similar TTE outcome measure defined as time to (a) two consecutive days with resolution of all targeted symptoms to "absent", and (b) four consecutive days with resolution of all targeted symptoms to "absent" (i.e., secondary outcome measure 5). For these two outcomes, as for the primary symptom outcome measure, the first day that a participant may meet this outcome will be day 1 (i.e. if all targeted symptoms are "absent" on (a) both day 1 and day 2, or (b) on days 1, 2, 3 and 4).

Sensitivity Analysis

It is possible that a participant may meet the primary TTE symptom outcome measure and subsequently be hospitalized or die. To assess how sensitive the primary symptom outcome results might be to this form of improvement and then deterioration, the primary analysis maybe repeated with participants who are hospitalized or who die by day 28 kept in the risk set through to day 28 without meeting the improvement/resolution outcome (i.e. assuming that they did not achieve this outcome if they actually did). It is recognized that this adaptation means that the outcome measure being analyzed is not a true TTE outcome measure but this analysis does allow an assessment of the sensitivity of results to the handling of participants who are hospitalized or who die. [Note: this sensitivity analysis was suggested by the Food and Drug Administration].

No additional sensitivity analyses are currently specified for this outcome measure. In part, this is because the proportion of participants enrolled early in ACTIV-2 who were lost to follow-up or who had extensive missing diary evaluations has been very low, and not all loss to follow-up or missingness patterns affect the determination of the TTE outcome. If necessary, exploratory sensitivity analyses will be undertaken to explore sensitivity of interpretation of results for the comparison of investigational agent to comparator intervention to losses to follow-up and/or missing data but these may need specification based on the form of missingness identified.

Subgroup Analyses

To evaluate the effect of the investigational agent in specific populations, the primary symptom outcome will be assessed among different subgroups. The same approaches outlined for the primary analysis will be implemented within each subgroup; formal comparisons across subgroups will not be done in phase II analyses. Pre-specified subgroups of interest include:

1) Sex (Male sex at birth, female sex at birth)
2) Race (white, non-white)
3) Ethnicity (Hispanic, non-Hispanic)
4) 'Risk of Severe Disease' Stratification [this may not be pursued for agents which predominantly enrolled participants who were at 'lower' risk for severe COVID progression]
5) Age Group (<60, ≥60)
6) Co-morbidity Status (no comorbidities, at least one comorbidity) [this may not be pursued for agents which predominantly enrolled participants who were at 'lower' risk for severe COVID progression]
7) Calendar days from first symptom associated with COVID-19 to start of investigational agent/comparator intervention Stratification (≤ 5 days, > 5 days)
8) Country (U.S., non-U.S.)
9) SARS-CoV-2 Variant (if available: categories will be determined based on prevalence of variants identified in testing).

Primary Virologic (Phase II)

Analysis Methods

Descriptive statistics (number and percentage) will be used to describe the proportion of participants with SARS-CoV-2 RNA < LLoQ in NP swabs at each scheduled measurement time (entry and days 3, 7, and 14).

The proportion of participants with SARS-CoV-2 RNA < LLoQ will be compared between randomized arms using log-binomial regression for repeated binary measurements with log-link. This model will be fitted using generalized estimating equations (GEE) to handle the repeated measurements with an independence working correlation structure and robust standard errors. For each time point after starting treatment, the model will include a main effect for time (indicator variable for each evaluation time), an interaction between time and randomized arm to evaluate differences between arms, and will adjust for baseline (day 0) log-10 transformed SARS-CoV-2 RNA level. The estimated adjusted relative risk of having RNA < LLoQ (and associated 95% CI) will be obtained for each measurement time from the model by taking the exponential of the time*randomized arm interaction parameter estimate (and associated 95% CI and two-sided p-value) for that measurement time. In the event the log-binomial regression model fails to converge, a Poisson regression model with robust variance and log-link will be used instead.

In this analysis, baseline SARS-CoV-2 RNA values will be imputed if the level is < LLoQ as outlined in section 4.1. It is not expected that a high proportion of baseline results will be < LLoQ. However, in the event that there is a non-negligible proportion of baseline results <LLoQ (defined as 10% or more of baseline results < LLoQ), an additional variable will be added to the model that will indicate whether the baseline result was above or below the LLoQ (included programmatically as "0" if above LLoQ, and "1" if below LLoQ).

A joint test of randomized arm across the time points will also be assessed, with degrees of freedom determined by the number of time points included. With this model, the comparison between randomized arms will use a two-sided Wald test with 5% type I error rate. Time points with zero events in either arm will not be included in the model (as estimation for such a model may be problematic; however data for these time points will be included in a descriptive summary of results over time points).

Missing data are assumed to be missing completely at random (MCAR) and will be ignored in the primary analysis. Sensitivity analyses will address possible informative missingness (see below).

If there is a need to conduct analyses of interim data (e.g. if requested by the DSMB), then the primary statistical analysis described above may be sensitive to small numbers of participants with data available at some measurement times. Because of this, such interim analyses will be undertaken using log-binomial models fit separately at each time point. If at a given time point, the number of participants with SARS-CoV-2 RNA < LLoQ or, conversely the number with SARS-CoV-2 RNA ≥ LLoQ in an arm (investigational agent or comparator intervention) is small, the asymptotic (large sample size) statistical theory underpinning these model-based analyses may be questionable. To address this, using a standard rule of thumb, if there are fewer than 5 events in either arm, inference based on Fisher's exact test to compare arms will be adopted instead of using the log-binomial regression model. If there are no participants have SARS-CoV-2 RNA <LLoQ (or all participants have SARS-CoV-2 RNA ≥ LLoQ) in both arms, then this will be stated and no formal statistical inferential analyses will be undertaken.

<u>Sensitivity Analyses</u>

The following sensitivity analyses are included to evaluate impact of different assumptions on the inference of the virology outcomes.

1) Repeat primary analysis, but restrict analysis population to exclude those with SARS-CoV-2 RNA < LLoQ at Day 0. This model will adjust for baseline log-10 transformed SARS-CoV-2 RNA level.
2) Repeat primary analysis, but impute missing data in the following manner (ignores missingness due to hospitalization and death):
    - For non-monotonic missingness, participants with missing SARS-CoV-2 results will have their values imputed as < LLoQ if the preceding and succeeding results are < LLoQ, otherwise the results will be imputed as ≥ LLoQ.
    - For monotonic missingness, inverse probability weighted GEE will be used (as implemented in SAS PROC GEE [Lin G, Rodriguez RN. Weighted methods for analyzing missing data with the GEE procedure. Paper SAS166-2015. 2015.]; based on Robins and Rotnitzky. Journal of the American Statistical Association. 1995 Mar 1;90(429):122-9; Preisser, Lohman, and Rathouz. Statistics in Medicine. 2002 Oct 30;21(20):3035-54).
3) Repeat primary analysis, but impute missing data in the following manner (special considerations for missingness due to hospitalization and death):
    - For missingness due to hospitalization or death, participants with missing SARS-CoV-2 results will have their values imputed as ≥ LLoQ.

- For non-monotonic missingness, participants with missing SARS-CoV-2 results will have their values imputed as < LLoQ if the preceding and succeeding results are < LLoQ, otherwise the results will be imputed as ≥ LLoQ.
- For monotonic missingness, inverse probability weighted GEE will be used.

<u>Supportive Analysis</u>

The primary analysis will be repeated without adjustment for baseline (Day 0) SARS-CoV-2 RNA level. In addition, the absolute difference in proportion of participants with RNA < LLoQ will be calculated at each measurement time; with associated 95% confidence intervals (calculated using the normal approximation to the binomial distribution).

<u>Subgroup Analyses</u>

To evaluate the effect of the investigational agent in specific populations, the primary virology outcome will be assessed among different subgroups. The same approaches outlined for the primary analysis will be implemented within each subgroup; formal comparisons across subgroups will not be done in phase II analyses. Pre-specified subgroups of interest include:

1) Sex (Male sex at birth, female sex at birth)
2) Race (white, non-white)
3) Ethnicity (Hispanic, non-Hispanic)
4) 'Risk of Severe Disease' Stratification
   [this may not be pursued for agents which predominantly enrolled participants who were at 'lower' risk for severe COVID progression]
5) Age Group (<60, ≥60)
6) Co-morbidity Status (no comorbidities, at least one comorbidity) [this may not be pursued for agents which predominantly enrolled participants who were at 'lower' risk for severe COVID progression]
7) Calendar days from first symptom associated with COVID-19 to start of investigational agent/comparator intervention Stratification (≤ 5 days, > 5 days)
8) Country (U.S., non-U.S.)
9) SARS-CoV-2 Variant (if available: categories will be determined based on prevalence of variants identified in testing)

Analyses of Secondary Objectives

<u>Analysis Population</u>

The analyses of the COVID-19 symptoms will include all randomized participants who started an investigational agent or the concurrent comparator intervention, according to a modified intent-to-treat (mITT) approach (Treated Population). Participants who have protocol violations will be included in the analysis but the protocol violations will be documented and described.

Note: Participants who are randomized but do not start investigational agent or comparator intervention are, per protocol, not to be followed.

Secondary Clinical Symptoms

<u>Analyses Methods</u>

Duration of Clinical Symptoms

Duration of clinical symptoms in phase III will be analyzed in the same manner as the primary phase II clinical symptom outcome.

Progression of Symptoms

Progression of one or more COVID-19-associated symptoms to a worse status than recorded in the study diary on day 0 (pre-treatment) on or before day 28 (i.e., absent to at least mild, mild to at least moderate, or moderate to severe) will be analyzed in the following manner. The proportion of participants who progressed will be estimated and compared between randomized arms using log-binomial regression, with log link, in order to obtain a risk ratio estimate; the model will include a main effect for randomized arm. In the event the log-binomial regression model fails to converge, a Poisson regression model with robust variance and log-link will be used instead. Participants who do not report worsened symptoms in study diaries, but are hospitalized or die in the first 28 days will be counted as having progression of symptoms in this analysis. Missing symptom scores not due to hospitalization or death will be imputed in the same manner as the primary symptom duration outcome (see above).

Return to Usual Health

The study diary includes a question: "*Have you returned to your usual (pre-COVID) health today?*" which is answered each day with possible responses "*yes*" or "*no*". Duration of time without self-reported return

to usual health is defined as the number of days from start of treatment to the first of two consecutive days that self-reported return to usual health was indicated as "*yes*".

Analysis (including handling of hospitalizations, deaths and missing data) will follow the same approach as for the primary clinical symptom duration outcome measure as described above.

COVID-19 Severity Ranking

COVID-19 severity ranking will be summarized with descriptive statistics. Participant specific scores will be compared between randomized arms using a two-sided Wilcoxon test with a 5% type I error rate. In addition, Hodges-Lehmann estimate and associated 95% CI for the location shift between the two arms will be provided.

The symptoms considered in calculating symptom duration are: feeling feverish, cough, shortness of breath or difficulty breathing at rest or with activity, sore throat, body pain or muscle pain/aches, fatigue (low energy), headache, chills, nasal obstruction or congestion (stuffy nose), nasal discharge (runny nose), nausea, vomiting, and diarrhea. Each of these symptoms is scored daily in a study diary by the participant as absent (score 0), mild (1) moderate (2) and severe (3) from day 0 (pre-treatment) to day 28.

COVID-19 severity ranking is defined as the participant-specific AUC of the total symptom score associated with COVID-19 disease, over time (through 28 days counting day 0 as the first day). For participants who are alive and were never hospitalized on or before day 28, the total symptom score on a particular day is the sum of scores for the targeted symptoms in the participant's study diary for that day. The AUC will be calculated using the trapezoidal rule and is defined as the area below the line formed by joining total symptom scores on each daily diary card from day 0 through day 28. The AUCs will be rescaled by time by dividing by 28, corresponding to the number of trapezoids created from daily diary cards between day 0 and day 28, in order to provide results on a symptom scale from 0 to 39.

Special considerations are made for participants who are hospitalized or die on or before day 28. Participants who are hospitalized or who die during follow-up through day 28 will be ranked as worse (i.e., worse severity) than those alive and never hospitalized through day 28 as follows (in worsening rank order): alive and not hospitalized at day 28; alive but hospitalized at day 28; and died on or before day 28. Programmatically, participants who were hospitalized, but are alive and no longer hospitalized at day 28 will be assigned an AUC (severity score) of 40, participants who are alive but remain hospitalized at day 28 will be assigned an AUC (severity score) of 41, and participants who die (regardless of when the death occurred through day 28) will be assigned a severity score of 42.

Participants who have incomplete diary cards for reasons other than hospitalization or death, and who are not subsequently hospitalized and do not die through day 28, will be addressed in the following manner:

1) Participants who are missing day 0 total symptom scores (i.e., participants who failed to complete the diary card on Day 0 and have no scores for any symptoms) will have their total symptom score imputed as the mean day 0 total symptom score among participants who report a total symptom score on day 0;
2) Participants who have some symptom scores missing at Day 0 (i.e., completed the diary card but did not score all symptoms) will have their total symptom score calculated as the mean of the available symptoms scores at Day 0, multiplied by 13;
3) Participants who stop completing their symptom diaries before day 28 will have their last total symptom score carried forward through day 28, and their AUC calculation done as noted above;
4) Participants who have diary cards with some, but not all symptom scores reported, their missing symptoms scores will be linearly interpolated based on the preceding and succeeding available scores for a given symptom, and their AUC calculation done as noted above;
5) Participants who have intermittent days with no symptom scores reported (i.e., all scores missing), their missing scores will be ignored in the AUC calculation, which is analogous to interpolating the total symptom scores.

Methods such as multiple imputation or IPCW may be considered if more than 10% of participants in either group stop completing their diaries before day 28 for reasons other than death or hospitalization.

To programmatically implement the imputation of the missing diary cards in order to calculate the AUC for participants who are not hospitalized and do not die by day 28, the following steps will be followed. First, imputation of total symptom scores will be done according to (1), (2), and (3). Next, (4) intermittent missing symptom scores for particular symptoms will be imputed using linear interpolation (see below formula) of the preceding and succeeding scores. Note: no imputation done for (5).

X = (Succeeding Score – Preceding Score) ÷ (Succeeding Day – Preceding Day)

Score on 1st Day missing = 1*X + Preceding Score

Score on 2nd Day missing = 2*X + Preceding Score
…..
Score on Zth Day missing = Z*X + Preceding Score.

Oxygen Saturation

Participants who are on supplemental oxygen at day 0 (pre-treatment) will not be included in these analyses.

Oxygen saturation will be analyzed in the same manner as the virology outcomes.

Descriptive statistics (number and percentage) will be used to describe the proportion of participants with oxygen saturation ≥ 96% at each scheduled measurement time (day 0 [pre-treatment] and days 3, 7, 14, and 28).

The proportion of participants with any oxygen saturation values ≥ 96% will be compared between randomized arms using log-binominal regression for binary repeated measurements with log-link. This model will be fitted using generalized estimating equations (GEE) to handle the repeated measurements with an independence working correlation structure and robust standard errors. For each time point after starting treatment, the model will include a main effect for time (indicator variable for each evaluation time), and an interaction between time and randomized arm to evaluate differences between arms, and will adjust for baseline oxygen saturation level. The estimated adjusted relative risk of having oxygen saturation values ≥ 96% (and associated 95% CI) will be obtained for each measurement time from the model by taking the exponential of the time*randomized arm interaction parameter estimate (and associated 95% CI) for that measurement time. In the event the log-binomial regression model fails to converge, a Poisson regression model with robust variance and log-link will be used instead.

A joint test of randomized arm across the time points will also be assessed, with degrees of freedom determined by the number of time points included. With this model, the comparison between randomized arms will use a two-sided Wald-test with 5% type I error rate. Time points with zero events in either arm will not be included in the model (as estimation for such a model may be problematic; however data for these time points will be included in a descriptive summary of results over time points).

Missing data are assumed to be missing completely at random (MCAR) and will be ignored in this analysis. Sensitivity analyses will address possible informative missingness (see below).

Non-parametric Wilcoxon rank-sum tests with a 5% type I error rate will compare oxygen saturation levels (continuous) between randomized arms, separately at each post-entry study day. In addition, Hodges-Lehmann estimate and associated 95% CI for the location shift between the two arms will also be provided.

<u>Sensitivity Analyses</u>

The following sensitivity analyses are included to evaluate impact of different assumptions on the inference of the clinical symptoms outcomes.

Oxygen Saturation ≥ 96%

1) Repeat primary analysis, but impute missing data in the following manner (ignores missingness due to hospitalization and death):
    - For non-monotonic missingness, participants with missing oxygen saturation results will have their values imputed as ≥96% if the preceding and succeeding results are ≥96%, otherwise the results will be imputed as <96%.
    - For monotonic missingness, inverse probability weighted GEE will be used.
2) Repeat primary analysis, but impute missing data in the following manner (special considerations for missingness due to hospitalization and death):
    - For missingness due to hospitalization or death, participants with missing oxygen saturation results will have their values imputed as <96%.
    - For non-monotonic missingness, participants with missing oxygen saturation results will have their values imputed as ≥96% if the preceding and succeeding results are ≥96% , otherwise the results will be imputed as <96%.
    - For monotonic missingness, inverse probability weighted GEE will be used.

<u>Supportive Analyses</u>

Duration of Symptoms

In support of the symptom duration outcome in phase III, the analysis will be repeated using the same approach described in the primary symptom duration analysis for a similar TTE outcome measure defined as time to two consecutive days with resolution of all targeted symptoms to “absent.” To address secondary objective 8, and in supportive of the symptom duration outcome in phase III, a similar TTE

outcome measure will also be examined defined as time to four consecutive days with resolution of all targeted symptoms to “absent,” (i.e. secondary outcome measure 5).

Return to Usual Health

The analysis of return to usual health will be repeated using the same approach described above for a similar TTE outcome measures defined as the number of days from start of investigational treatment until the first of four consecutive days that a participant reported return to usual (pre-COVID) health.

COVID-19 Severity Ranking

To evaluate the effect of the investigational agent on COVID-19 symptom severity over different time-periods, analyses of COVID-19 severity ranking based on partial AUCs will also be examined. The time-periods considered include day 0 to day 7, day 0 to day 14, and day 0 to day 21. These analyses will compare participant specific AUCs between randomized arms using a two-sided Wilcoxon test with a 5% type I error rate. In addition, Hodges-Lehmann estimate and associated 95% CI for the location shift between the two arms will also be provided.

For each time period, for participants who are alive and were never hospitalized in that time period (i.e., as of 7 days, 14 days, and 21 days), the severity ranking will be based on their AUC of the symptom score associated with COVID-19 disease over time (through day 7, 14, 21, respectively, counting day 0 as the first day) assigned as the sum of scores for the targeted symptoms in the participant's study diary. The AUCs will be calculated using the trapezoidal rule and is defined as the area below the line formed by joining total symptom scores on each daily diary card from day 0 through day 7, 14, and 21, respectively. The AUCs will be rescaled by time in order to provide results on a symptom scale from 0 to 39. This will be done by dividing the AUC by 7, 14, or 21, respectively, corresponding to the number of trapezoids created from daily diary cards between day 0 and the last day considered in the calculation (i.e., day 7, day 14, and day 21).

Participants who die or are hospitalized in the time interval being considered (through day 7, day 14, or day 21, respectively) will be ranked as worse (i.e., worse severity) than those alive and never hospitalized in worsening rank order. Programmatically, participants who die in the time interval will be assigned an AUC (severity score) of 42 (worst rank) regardless of when the death occurred in the interval, participants who are alive but remain hospitalized at last day of the interval will be assigned an AUC (severity score) of 41 (second worst rank), and participants who are alive but are no longer hospitalized on the last day of the interval will be assigned an AUC (severity score) of 40 (the third worst rank).

Participants who have incomplete diary cards for reasons other than hospitalization or death will be addressed in the same manner as the analyses of COVID-19 severity through day 28, outlined in the above section of the SAP.

Oxygen Saturation

The primary analysis will be repeated without adjustment for baseline oxygen saturation level. In addition, the absolute difference in proportion of participants with oxygen saturation ≥ 96% will be calculated at each measurement time, with associated 95% confidence intervals (calculated using the normal approximation to the binomial distribution).

For analyses based on interim data (e.g. DSMB reviews), the proportion of participants with oxygen saturation ≥ 96% will also be compared using log-binomial models fit separately at each time point. If at a given time point there are zero events in either arm, a p-value from Fisher's exact test will be provided instead. If there are zero events in both arms, then this will be stated and no formal statistical inferential analyses will be undertaken.

<u>Subgroup Analyses</u>

Duration of Clinical Symptoms

In phase III, to evaluate the effect of the investigational agent on symptom duration in specific populations (address secondary objective 8), secondary outcome 4 will be assessed among different subgroups. These will also be conducted for the supportive outcome of time to two consecutive days of resolution of all symptoms to "absent". Descriptive analyses for the following subgroups will be considered. A separate analysis plan for multivariate/personalized-medicine type analyses across subgroups will be developed at a later time.

Pre-specified subgroups of interest include:

1) Sex (Male sex at birth, female sex at birth)
2) Race (white, non-white)
3) Ethnicity (Hispanic, non-Hispanic)
4) Age Group (<60, ≥60)
5) Calendar days from first symptom associated with COVID-19 to start of investigational agent/comparator intervention Stratification (≤ 5 days, > 5 days)
6) Country (U.S., non-U.S.)
7) SARS-CoV-2 Variant (if available: categories will be determined based on prevalence of variants identified in testing)

Secondary Virology

The schedule of evaluations in protocol version 7.0 indicates that only NP swabs will collected in both phase II and phase III, and therefore only analyses of SARS-CoV-2 RNA from NP swabs are outlined below. Some agents may have completed enrollment in phase II prior to implementing protocol version 7.0, and therefore may have additional specimens collected for SARS-CoV-2 RNA testing. If analyses of these additional specimens are pursued, then the approach will be as defined in the relevant previous version of the SAP.

Analysis Population

The analyses of the virology objectives will include all randomized participants who started an investigational agent or the concurrent comparator intervention, according to a modified intent-to-treat (mITT) approach (Treated Population). Participants who have protocol violations will be included in the analysis but the protocol violations will be documented and described.

Note: Participants who are randomized but do not start investigational agent or comparator intervention are, per protocol, not to be followed and will be replaced.

Analysis Methods

Quantification (< LLoQ versus ≥ LLoQ) of SARS-CoV-2 RNA at day 3 (this is a secondary outcome for the active-controlled phase 3 only)

Descriptive statistics (number and percentage) will be used to describe the proportion of participants with SARS-CoV-2 RNA < LLoQ from staff-collected NP swabs at entry and day 3.

The proportion of participants with SARS-CoV-2 RNA < LLoQ day 3 will be compared between randomized arms using log-binominal regression for repeated binary measurements with log-link. The model will include a main effect for treatment and will adjust for baseline (day 0) log-10 transformed SARS-CoV-2 RNA level. The estimated adjusted relative risk of having RNA < LLoQ (and associated 95% CI and two sided p-value) will be obtained by taking the exponential of the treatment parameter estimate (and associated 95% CI). In the event the log-binomial regression model fails to converge, a Poisson regression model with robust variance and log-link will be used instead.

In this analysis, baseline SARS-CoV-2 RNA values will be imputed if the level is < LLoQ as outlined in section 4.1. It is not expected that a high proportion of baseline results will be < LLoQ; however, in the event that there is a non-negligible proportion of baseline results < LLoQ (defined as 10% or more of baseline results < LLoQ), an additional variable will be added to the model that will indicate whether the baseline result was above or below the LLoQ (included programmatically as “0” if above LLoQ, and “1” if below LLoQ). Missing data are assumed to be missing completely at random (MCAR) and will be ignored in these analyses; however, sensitivity analyses will address possible informative missingness (see below).

Level (Quantitative) of SARS-CoV-2 RNA

Descriptive statistics will be used to describe the levels of SARS-CoV-2 RNA at each scheduled measurement time for staff-collected NP swabs.

Non-parametric Wilcoxon rank-sum tests with a 5% type I error rate will compare SARS-CoV-2 RNA level (continuous) between randomized arms, separately at each post-entry study day; results below the limit of detection will be imputed as the lowest rank and values above the limit of detection but below the LLoQ will be imputed as the second lowest rank. In addition, Hodges-Lehmann estimate and associated 95% CI for the location shift between the two arms will also be provided.

Missing data in analysis of continuous SARS-CoV-2 RNA levels are assumed to be missing completely at random (MCAR) and will be ignored in analysis.

AUC of SARS-CoV-2 RNA

In phase II only, levels of log-10 transformed SARS-CoV-2 RNA, measured from NP swabs will be analyzed using participant-specific AUCs. In this analysis, the AUC is defined as the area below the line formed by joining measured values at each successive measurement time and above the lower limit of quantification of the assay, calculated using trapezoidal rule. Programmatically, the trapezoidal rule will be applied to the following values: $\max[0, \log_{10}(\text{RNA})-\log_{10}(\text{LLoQ})]$, obtained at the scheduled measurement times between and including day 0 and day 14.

Missing values with preceding and succeeding values will be ignored, which is equivalent to linearly interpolating the RNA levels from preceding and succeeding values. Missing values with no succeeding values will be imputed using linear imputation assuming that the RNA level at day 14 equals the LLoQ (as it is anticipated that nearly everyone will clear virus over 14 days). If the day 0 result is missing then the participant will be excluded from analysis. The participant-specific AUCs will be compared between randomized arms using a two-sided Wilcoxon test with 5% type I error rate. In addition, Hodges-Lehmann estimate and associated 95% CI for the location shift between the two arms will also be provided.

Missing data in the AUC analysis of continuous SARS-CoV-2 RNA levels are assumed to be missing completely at random (MCAR) and will be ignored in analysis.

Sensitivity Analyses

The following sensitivity analyses are included to evaluate impact of different assumptions on the inference of the virology outcomes.

All Virology Outcomes

1) Repeat primary analysis, but restrict analysis population to exclude those with SARS-CoV-2 RNA < LLoQ at Day 0. This model will adjust for baseline log-10 transformed SARS-CoV-2 RNA level.

Dichotomous Virology Outcomes

1) Repeat primary analysis, but impute missing data in the following manner (ignores missingness due to hospitalization and death):

- For non-monotonic missingness, participants with missing SARS-CoV-2 results will have their values imputed as < LLoQ if the preceding and succeeding results are < LLoQ, otherwise the results will be imputed as ≥ LLoQ.
- For monotonic missingness, inverse probability weighted GEE will be used

2) Repeat primary analysis, but impute missing data in the following manner (special considerations for missingness due to hospitalization and death):
    - For missingness due hospitalization or death, participants with missing SARS-CoV-2 results will have their values imputed as ≥ LLoQ.
    - For non-monotonic missingness, participants with missing SARS-CoV-2 results will have their values imputed as < LLoQ if the preceding and succeeding results are < LLoQ, otherwise the results will be imputed as ≥ LLoQ.
    - For monotonic missingness, inverse probability weighted GEE will be used.

Supportive Analysis

The dichotomous virology analysis will be repeated without adjustment for baseline (Day 0) SARS-CoV-2 RNA level. In addition, the absolute difference in proportion of participants with RNA < LLoQ will be calculated at each measurement time; with associated 95% confidence intervals (calculated using the normal approximation to the binomial distribution).

Exploratory Analyses

New SARS-CoV-2 among Household Contacts

The analysis of household contacts will be restricted to the subset of randomized participants in the Treated Population who reported that they share indoor living space or housekeeping space with someone.

New SARS-CoV-2 positivity among household contacts through day 28 will be analyzed in the following manner. The proportion of participants with a household contact that tests positive for SARS-CoV-2 after the participant initiates study investigational agent or concurrent comparator intervention through day 28, will be estimated and compared between randomized arms using log-binomial regression, with log link, in order to obtain a risk ratio estimate; the model will include a main effect for randomized arm. In the event the log-binomial regression model fails to converge, a Poisson regression model with robust variance and log-link will be used instead. Missing data will be considered missing completely at random in analysis.

The same analysis approach will be used to compare the proportion of participants with a household contact that tests positive for SARS-CoV-2 or has COVID-19 symptoms after the participant initiates study investigational agent or concurrent comparator intervention through day 28.

Analysis of new SARS-CoV-2 positivity, and new SARS-CoV-2 positivity or COVID-19 symptoms, among household contacts through week 24 will be analyzed as in the same way as above for these outcomes through day 28.

Hospitalization Course

Analyses of clinical outcomes among those hospitalized will include all randomized participants who started an investigational agent or the concurrent comparator intervention who were also hospitalized. The analyses will be limited to descriptive summaries by randomized arm, as these analyses are restricted to participants who were hospitalized and so are not randomized comparisons.

Duration of hospitalization and duration of ICU admission will be summarized with continuous descriptive statistics. Duration of hospitalization/ICU through day 28 will be calculated as the difference between the date of discharge and the date of admission; the duration will be truncated at Day 28, if the participant is still hospitalized at Day 28. If data on discharge dates occurring after Day 28 are complete at the time of analysis of the Day 28 data, an additional descriptive analysis of durations for hospitalizations starting on or before Day 28 will be undertaken. The proportion of participants with ICU admission, among those hospitalized, will be summarized with frequencies and percentages. The worst clinical status (ordinal outcome) will be summarized with frequencies and percentages. Descriptive summaries of use of remdesivir and dexamethasone, and other approved medications for treatment of COVID-19 used during hospitalization will also be included.

This analysis will be done through day 28 and separately through week 72.

Resistance Mutations

Analyses addressing the emergence of new resistance mutations will be outlined for each investigational agent in agent-specific SAP appendices based on information about resistance available at the time of completion of sequencing.

Interim Analysis Considerations

Interim analyses of the placebo-controlled superiority phase III evaluation of an agent was finished at the time of finalization of SAP version 7.0. The following from protocol version 7.0 describes the interim analysis considerations for the active-controlled non-inferiority phase III evaluation of an agent.

The two-sided 95% confidence interval mentioned above [see section 2.5.3 of the SAP] will be adjusted for the multiple interim analyses to preserve the confidence interval coverage to at least 95% (this is also referred to as using "repeated" confidence intervals).

The standard Lan and DeMets approach will be used to achieve this, incorporating an O'Brien and Fleming spending function. For simplicity, the information scale for the spending function will be determined as the proportion of the planned enrollment randomized to the investigational agent being evaluated at the time of the interim analysis. As an example, if in practice, the analyses were after exactly 25%, 50%, 75% and 100% of the planned enrollment, then the nominal confidence intervals used to assess efficacy would have coverage 99.9985% at the first analysis, 99.70% at the second analysis, 98.17% at the third analysis and 95.60% at the fourth analysis (these were obtained from PASS software). However, as the O'Brien and

Fleming spending function is very conservative at early interim analyses, making stopping very difficult, for the assessment of inferiority of an investigational agent compared to the active comparator agent, an asymmetric approach will be used to reduce the level of evidence required for early stopping in the event that an investigational agent appears inferior to the active comparator agent. Specifically, if a nominal confidence interval with coverage of greater 99.9% at an early interim analysis is suggested by use of the O'Brien and Fleming spending function, then a nominal confidence interval with coverage of 99.9% will be used instead for assessing inferiority of the investigational agent.

The DSMB will also monitor the proportion hospitalized/dead in the active comparator arm as this key parameter, coupled with the non-inferiority margin, underpins the study design. The study is designed assuming that the underlying true proportion of participants on the active comparator agent is 2.3%. This is the proportion (32/1392) observed for high risk participants in the Regeneron COV-2067 trial for the agent (pooling across doses studied in that trial; FDA communication to DAIDS/NIAID). A 95% confidence interval for this proportion is (1.5%, 3.1%). An assessment of non-inferiority in this study would be more difficult if the proportion of participants on the active comparator agent in this study is somewhat different from that in the Regeneron COV-2067 (e.g., somewhat outside of the range suggested by the confidence interval).

For example, this might arise if variants of SARS-CoV-2 are present in the study population which the active comparator agent is less effective against. Such an issue would undermine the use of a 3% non-inferiority margin in this study. It may however be addressed by focusing the non-inferiority assessment on the subpopulation in this study without such variants (assuming these have been identified), or in establishing superiority of the investigational agent in the overall study population. This may require a larger sample size to maintain power.

Another potential reason for a somewhat different proportion hospitalized/dead on the active comparator agent in this study versus that in the Regeneron COV-2067 study is that this study is likely to enroll in a number of different countries, whereas the Regeneron COV-2067 enrolled primarily in the United States. Aside from possible differences in circulating variants among countries, differences among countries in clinical practice and/or in the availability of hospital care might lead to differences in hospitalization/death rates. The DSMB will monitor descriptive results by country and provide guidance about countries with notably low or high rates of hospitalization/death.

Appendix 1: Algorithm for Handling Missing Symptom Evaluations for the Primary Phase II Symptom Outcome Measure.

The following algorithmic approach will be used to handle hospitalizations and deaths, as well as missing data, in constructing the TTE symptom-based outcome measure. The steps of the algorithmic approach will be undertaken in the following order:

a. If a participant has none of the targeted symptoms evaluated at any time during follow-up (including if due to the diary never being returned):

i. If the participant died on or before study day 28, then the participant will be assumed not to have had symptoms improved/resolved prior to death but will be retained in the risk set through to 28 days (programmatically, this is achieved by considering the participant censored after 27 days). [The underlying premise is that (if evaluations had been available) the participant had targeted symptoms that did not improve/ resolve through to death. Retention in the risk set through to 27 days provides for appropriate estimation of the cumulative proportion of the Treated Population who had a good outcome, i.e. symptoms improved/resolved for two consecutive days].

ii. If the participant was hospitalized on or before study day 28, then the participant will be assumed not to have had symptoms improved/resolved through to the day of hospital discharge and their follow-up will be censored at the day before hospital discharge (or at day 27 if earlier). [The underlying premise is that (if evaluations had been available) the participant had symptoms that did not improve/resolve through to admission to hospital and during hospitalization. Censoring at the day before hospital discharge assumes that the participant's subsequent unobserved symptom course would have been the same as other participants who were still at risk on the study day that discharge occurred].

iii. If the participant was not known to have died or been hospitalized, then their follow-up will be censored at day 0. [Censoring at day 0 assumes that their subsequent unobserved symptom course would have been the same as other participants in the Treated Population].

b. **If a participant has one or more (but not all) targeted symptoms with no evaluations for all days from day 0 through day 28**:

The TTE outcome measure for this participant will be evaluated based on the remaining targeted symptoms with missing data handled for those targeted symptoms as described below in subsection c. [In essence, this is assuming that if the participant had evaluated the unscored symptoms that they would have shown improvement/resolution for two consecutive days as the same time, or earlier, as the symptoms that they did score. With this assumption, using the available symptom data is considered preferable to alternative strategies of censoring their TTE at day 0 or assuming that the unscored symptoms never improved/resolved throughout follow-up with censoring at day 27].

c. **If participant has an evaluation on day 0 and/or on days between day 1 and day 28 during follow-up on all targeted symptoms (or, per section b above, on a subset of targeted symptoms):**

For each symptom having an evaluation on at least one day between day 0 and day 28 inclusive, programmatically values will be imputed for unobserved evaluations after death, for days in hospital, and for missing values as follows:

i. For days after death (and the day of death if no diary was completed that day), set all symptoms to “severe”. This means that each symptom is never considered improved/ resolved unless this was achieved prior to death. For participants who did not achieve the event prior to death, the effect of this is to retain them in the risk set from death through to 28 days without meeting the symptom improvement/resolution criteria providing for appropriate estimation of the cumulative proportion of the Treated Population who had symptoms sufficiently improved/resolved throughout follow-up time.

ii. For days hospitalized (including day of admission if no diary was completed that day, and including the day of discharge if no diary was completed that day), set all symptoms to “severe” irrespective of whether or not the diary was completed. This means that each symptom is not considered improved/ resolved while a participant was hospitalized, but note that a participant could still have achieved the symptom outcome criteria prior to hospitalization.

iii. Impute a missing score for a symptom on day 0 as “mild”. If also missing on day 1 or for a sequence of consecutive days from day 1 but with at least one score during follow-up, impute the missing values on day 1 through to the first available score as “mild”. This means that the TTE criteria cannot be met during follow-up while a participant has a sequence of one or more missing values starting on day 0. The choice of imputing a missing value as “mild” on day 0 means that that symptom has to resolve to “absent” during follow-up before the TTE criteria can be met.

iv. For intermittent missingness during follow-up after day 0, impute a missing score for a symptom as the worst of (a) the last available value (actually provided by the participant or imputed due to hospitalization) before the missing value, and (b) the first available value (actually provided by the participant or imputed due to hospitalization) after the missing value, irrespective of the length of the sequence of missing values for the symptom. This gives potentially longer times until symptom improvement/resolution (compared with what might have occurred if the evaluations were available) if either of the preceding and succeeding values do not meet the criteria for improvement/ resolution, but potentially shorter times if both the preceding and succeeding values meet the criteria.

v. For monotonic missingness through to day 28 (i.e. a sequence of missing values during follow-up through to and including day 28 due to loss to follow-up, participant choice not to fully complete their diary, or an early day 28 clinic visit at which the diary is returned), censor the follow-up for this specific symptom at the last day that the relevant criterion for symptom improvement could have been met (this would be the day before the last diary entry for a given symptom, the day before the day of discharge, or the day before the day of withdrawal from the study during hospitalization). This assumes that the censoring is non-informative about when the criterion would have been met if diaries had been fully completed.

The TTE outcome is then calculated as the first of two successive days meeting the symptom improvement/resolution criteria using the combined observed and imputed data for all symptoms with one

or more evaluations observed during follow-up between day 0 and day 28, inclusive. In the event that the censoring due to monotonic missingness differs among targeted symptoms (e.g. because the participant stops completing the diary for one symptom earlier than for other symptoms), then the TTE outcome will be calculated using the available observed and imputed data, and censoring of the TTE outcome will be at the time of censoring of the symptom with the longest time to censoring.

Appendix 2: Statistical Considerations for BRII-198 + BRII-196

NOTE: Enrollment to BRII-198+BRII-196 started under protocol version 2 and continued through to protocol version 6.0. There were changes to the phase II primary virology and symptom outcomes measures in protocol version 3 from protocol version 2. No analyses comparing BRII-198+BRII-196 to placebo for the protocol version 2 phase II outcomes had been undertaken when protocol version 3 was implemented, and this SAP documents the intent that the phase II primary virology and symptom outcome measures in protocol version 3 (and continued in subsequent protocol versions) are primary using data from participants enrolled under all protocol versions. All participants enrolled to evaluate BRII-198+BRII-196 were randomized to active agent or placebo and so placebo is mentioned as a the comparator intervention throughout this appendix.

Randomization Details

Phase III is stratified by time from symptom onset (≤ 5 days versus > 5 days).

Secondary Outcome Measures

1) Phase II only:New Grade 2 or higher AE through week 72. [Supportive of Primary Objective 1] New Grade 2 or higher AE is defined as: Grade 2 or higher event that was new in onset or aggravated in severity or frequency from the baseline condition (i.e., Grade 1 at baseline escalates to Grade 2 or higher, or Grade 2 at baseline escalates to Grade 3 or higher, or Grade 3 at baseline escalates to Grade 4 or higher), following the start of study treatment
2) Phase III only:New Grade 3 or higher AE through week 72. [Supportive of Primary Objective 1] New Grade 3 or higher AE is defined as: Grade 3 or higher event that was new in onset or aggravated in severity or frequency from the baseline condition (i.e., Grade 1 or 2 at baseline escalates to Grade 3 or higher, or Grade 3 at baseline escalates to Grade 4 or higher), following the start of study treatment.

Analysis Approaches

The secondary safety outcome measures specified in this appendix will be analyzed in the same manner as the primary and secondary safety outcomes defined in the main text of the SAP. The same analysis population will be considered, however, the placebo control arm will be restricted to those who randomized to a placebo that included follow-up through at least week 72 (i.e. will be restricted the those who received placebo for BRII-196+BRII-198 or a placebo arm for an agent with follow up through to at least week 72).

Appendix 3: Statistical Considerations for AZD7442 IV

NOTE: AZD7442 IV is only being evaluated in this study in phase II with a placebo control.

Secondary Outcome Measures

1) Phase II only:New Grade 2 or higher AE through week 72. [Supportive of Primary Objective 1] New Grade 2 or higher AE is defined as: Grade 2 or higher event that was new in onset or aggravated in severity or frequency from the baseline condition (i.e., Grade 1 at baseline escalates to Grade 2 or higher, or Grade 2 at baseline escalates to Grade 3 or higher, or Grade 3 at baseline escalates to Grade 4 or higher), following the start of study treatment

Analysis Approaches

The secondary safety outcome measures specified in this appendix will be analyzed in the same manner as the primary and secondary safety outcomes defined in the main text of the SAP. The same analysis population will be considered, however, the placebo control arm will be restricted to those who were randomized to a placebo that included follow-up through at least week 72 (i.e. will be restricted the those who received placebo for AZD7442 IV or a placebo arm for an agent with follow up through to at least week 72).

Appendix 4: Statistical Considerations for AZD7742 IM

NOTE: AZD7442 IM is only being evaluated in this study in phase II with a placebo control.

Secondary Outcome Measures

1) Phase II only:New Grade 2 or higher AE through week 72. [Supportive of Primary Objective 1] New Grade 2 or higher AE is defined as: Grade 2 or higher event that was new in onset or aggravated in severity or frequency from the baseline condition (i.e., Grade 1 at baseline escalates to Grade 2 or higher, or Grade 2 at baseline escalates to Grade 3 or higher, or Grade 3 at baseline escalates to Grade 4 or higher), following the start of study treatment

Analysis Approaches

The secondary safety outcome measure specified in this appendix will be analyzed in the same manner as the primary and secondary safety outcomes defined in the main text of the SAP. The same analysis population will be considered, however, the placebo control arm will be restricted to those who were randomized to a placebo that included follow-up through at least week 72 (i.e. will be restricted the those who received placebo for AZD7442 IM or a placebo arm for an agent with follow up through to at least week 72).
