# Supplementary material for: Safety and Efficacy of Combined Tixagevimab and Cilgavimab Administered Intramuscularly or Intravenously in Nonhospitalized Patients With COVID-19: 2 Randomized Clinical Trials
Source: JAMA Netw Open. 2023 Apr 26;6(4):e2310039. doi: 10.1001/jamanetworkopen.2023.10039 (PMC10134004; doi:10.1001/jamanetworkopen.2023.10039)
Supplement: Supplement 2. — eMethods 1. Participant Enrollment and Study Analysis eFigure. CONSORT Diagram eTable 1. Clinical Outcomes Amongst Persons Randomized to Receive Tixagevimab-Cilgavimab, 600 mg IM or 300 mg IV, or Placebo eTable 2. Clinical Outcomes Stratified by Symptom Duration Prior to Randomization eTable 3. Number and Proportion of Participants With SARS-CoV-2 RNA Levels Below and at or above the LLOQ From NP Swabs by Duration of Symptoms Prior to Enrollment (≤ 5 or >5 d), Amongst Those in the Tixagevimab-Cilgavimab, 600 mg IM and 300 mg IV, Groups eTable 4. Change in NP RNA From Day 0 to Day 3 Among Those With NP RNA at Day 0 Greater Than LLOQ eTable 5. Number and Proportion of Participants With Unquantifiable SARS-CoV-2 RNA Levels From Participant Self-collected Anterior Nasal (AN) Swabs Amongst Persons in the Tixagevimab-Cilgavimab, 600 mg IM and 300 mg IV, Groups eTable 6. Number of Participants With Adverse Events Grade 2 or Higher Through Day 28 eTable 7. Variants Among Persons Randomized to Either IV or IM Tixagevimab-Cilgavimab or Placebo Groups eMethods 2. Site Acknowledgments [file jamanetwopen-e2310039-s002.pdf]

Supplementary Online Content

Bender Ignacia RA, Chew KW, Moser C, et al; Accelerating COVID-19 Therapeutic Interventions and Vaccines (ACTIV)–2/A5401 Study Team. Safety and efficacy of combined tixagevimab and cilgavimab administered intramuscularly or intravenously in nonhospitalized patients with COVID-19: 2 randomized clinical trials. *JAMA Netw Open*. 2023;6(4):e2310039. doi:10.1001/jamanetworkopen.2023.10039

eMethods 1. Participant Enrollment and Study Analysis

eFigure. CONSORT Diagram

eTable 1. Clinical Outcomes Amongst Persons Randomized to Receive Tixagevimab-Cilgavimab, 600 mg IM or 300 mg IV, or Placebo

eTable 2. Clinical Outcomes Stratified by Symptom Duration Prior to Randomization

eTable 3. Number and Proportion of Participants With SARS-CoV-2 RNA Levels Below and at or above the LLOQ From NP Swabs by Duration of Symptoms Prior to Enrollment (≤ 5 or >5 d), Amongst Those in the Tixagevimab-Cilgavimab, 600 mg IM and 300 mg IV, Groups

eTable 4. Change in NP RNA From Day 0 to Day 3 Among Those With NP RNA at Day 0 Greater Than LLOQ

eTable 5. Number and Proportion of Participants With Unquantifiable SARS-CoV-2 RNA Levels From Participant Self-collected Anterior Nasal (AN) Swabs Amongst Persons in the Tixagevimab-Cilgavimab, 600 mg IM and 300 mg IV, Groups

eTable 6. Number of Participants With Adverse Events Grade 2 or Higher Through Day 28

eTable 7. Variants Among Persons Randomized to Either IV or IM Tixagevimab-Cilgavimab or Placebo Groups

eMethods 2. Site Acknowledgments

This supplementary material has been provided by the authors to give readers additional information about their work.

© 2023 Bender Ignacio RA et al. *JAMA Network Open*.

eMethods. Participant Enrollment and Study Analysis

Participant Recruitment and Enrollment:

The ACTIV-2 study team, the AIDS Clinical Trials Group COVID-19 Community Advisory Board, and Klick Health Media co-produced a multifaceted community outreach cmpaign to increase awareness of the trial and facilitate enrollment of those newly diagnosed with COVID-19. At the center of this initiative was a dedicated study website (www.riseaboveCOVID.org), developed with Jumo Health, Inc, which in addition to containing detailed information about COVID-19, monoclonal antibodies, and the trial, included a toll-free number for a study call center. The call center was operational 24 hours a day and along with the website was available in English and Spanish. Call center staff used IRB-approved scripts to vet callers for basic eligibility and then connected them to the nearest participating research site for more in-depth screening.

Initiatives to direct traffic to the study website and call center included: search engine marketing (paid search), social media advertisements, and listing of the trial on US Department of Health and Human Services COVID-19 websites. Non-digital initiatives included newspaper advertisements, earned media (interviews with study and site leads with national and local media), and partnerships with COVID-19 testing operations to send text messaging or call those testing positive to provide information about the study. A barbershop and salon initiative was launched in select cities in conjunction with I Choose Life, a Los Angeles, CA community-based health advocacy organization that led trainings of shop staff so that they could discuss the trial and its importance with patrons. I Chose Life also engaged community political and religious leaders in these cities as part of a city-wide social mobilization effort to encourage minority recruitment into the trial.

Local sites developed their own outreach activities including COVID-19 testing events and local advertising,

All outreach and marketing activities were approved by the study IRB.

Individuals contacting a study site were screened for eligibility per the study protocol. Documentation of SARS-CoV-2 detection with an antigen or molecular test was required. Symptoms and symptom onset was self-reported. Informed consent was obtained following Good Clinical Practice (GCP) and may have been performed remotely (eConsent, with an institutionally approved FDA 21 CFR part 11 compliant platform) or in person. Conference calls

© 2023 Bender Ignacio RA et al. *JAMA Network Open*.

were held regularly with representatives from all sites to review study procedures, including any changes to eligibility or procedures.

Methods for nasopharyngeal and anterior nasal swab sample collection and quantitative SARS-CoV-2 RNA measurement

Nasopharyngeal (NP) and anterior nasal (AN) samples were collected using standardized swabs and collection procedures. AN swabs collected daily on days 0-14 by participants were stored at cool temperatures (refrigerated or in a study-provided cooler with a combination of refrigerated and frozen gel packs) and returned to the site and frozen at -80°C (-65°C to -95°C) within 7 days of collection. Site-collected NP swabs, as well as AN swabs collected on site, and EDTA plasma samples were frozen and stored at -80°C (-65°C to -95°C) on the day of collection. All samples were shipped on dry ice to a central laboratory (University of Washington) for quantitative SARS-CoV-2 RNA testing using the Abbott m2000sp/rt platform with a validated internal standard (1). The collection, storage, processing, and assay methods have previously been validated (1). The assay limit of detection (LoD) was 1.4 $\log_{10}$ copies/mL, lower limit of quantification (LLoQ) was 2 $\log_{10}$ copies/mL, and upper limit of quantification (UloQ) was 7 $\log_{10}$ copies/mL. For samples with RNA levels >UloQ, the assay was rerun with dilutions to obtain a quantitative value.

Methods for SARS-CoV-2 sequencing and variant analysis

S gene sequencing was performed on AN and NP swab samples for all participants with a SARS-CoV-2 RNA level $\geq$2 $\log_{10}$ SARS-CoV-2 RNA copies/mL at study entry or the earliest subsequent time point. Viral RNA extraction was performed on 1 mL of swab fluid by use of the TRIzol-LS™ Reagent (ThermoFisher), as previously described (2). cDNA synthesis was performed using Superscript IV reverse transcriptase (Invitrogen) and S gene amplification was performed using a nested PCR strategy with in-house designed primer sets targeting codons 1-814 of Spike (3). llumina library construction was performed using the Nextera XT Library Prep Kit (Illumina). Sequencing was performed on the Illumina MiSeq platform and deep sequencing data analysis was carried out using the Stanford Coronavirus Antiviral & Resistance Database (CoVDB) platform (4). The CodFreq pipeline (https://github.com/hivdb/codfreq) uses MiniMap2 version 2.22 to align the input FASTQ sequence with Wuhan-

© 2023 Bender Ignacio RA et al. *JAMA Network Open*.

Hu-1 reference. The output of MiniMap2, an aligned SAM file, is converted to a CodFreq file by an in-house written Python script using a PySam library (version: 0.18.0) and further analyzed with the Sierra SARS-CoV-2 program. Amino acid variants were then called at the codon level and used for resistance interpretation with a 1% limit of detection. SARS-COV-2 variant calling was done using 3 different variant calling platforms namely, CoVDB (5) (version id: 17d0d87), Scorpio call (6) (v0.3.16) & Nextclade (7) (v1.10.3). Wherever possible, final variant call was made based on the consensus of variant calls made across these 3 platforms, otherwise CoVDB based call was the final variant call.

Symptom diary questions and methods for severity ranking analysis

The ACTIV-2 symptom diary was completed by participants on the day of enrollment (Day 0) prior to receipt of investigational agent or placebo and then daily for 28 days. It included 13 targeted symptoms, which were each scored as absent, mild, moderate, or severe, and a question about whether they had returned to pre-COVID-19 health, in which the participant answered "yes or no" to the study diary question "Have you returned to your usual (pre-COVID) health today?". This diary was completed on paper or electronically and participants were asked to record their symptoms in the study diary at about the same time every day. Participants received a reminder by study staff every day on days 1-28 to complete the symptom diary. Symptoms included: feeling feverish, cough, shortness of breath or difficulty breathing at rest of with activity, sore throat, body pain or muscle pain/aches, fatigue (low energy), headache, chills, nasal obstruction or congestion (stuffy nose), nasal discharge (runny nose), nausea, vomiting, and diarrhea.

A COVID-19 severity ranking was based on symptom severity scores over time during the 28-day period from and including the day of the first dose of investigational agent or placebo, hospitalization, and death. For participants who were alive at 28 days and not previously hospitalized, the severity ranking was based on their area under the curve (AUC) of the daily total symptom score associated with COVID-19 over time (through 28 days counting day 0 as the first day) where the total symptom score on a given day is defined as the sum of scores for the targeted symptoms in the participant's study diary (each individual symptom is scored from 0 to 3). Participants who were hospitalized or who died during follow-up through 28 days were ranked as worse than those alive and never hospitalized as follows (in worsening rank order): alive and not hospitalized at 28 days; hospitalized but alive at 28 days; and died at or before 28 days.

© 2023 Bender Ignacio RA et al. *JAMA Network Open*.

Power and Sample Size (see also Section 5 Statistical Analysis Plan)

Each phase 2 evaluation was powered based on the primary virology outcome. The target sample size was 220 (110 each for T/C route and placebo). With this sample size, there was at least 82% power to detect a 20% absolute increase in the proportion with SARS-CoV-2 RNA <LLoQ on a given day in each T/C arm compared to its placebo using a two-sided 5% type I error rate. The study was not designed to have a high level of power for the phase 2 symptom duration outcome. Assuming that 100 of the 110 participants in each of the investigational agent and placebo control groups will provide study diary data, and assuming a normal distribution for log10 symptom durations with standard deviation of 0.425, then the phase II component of the study will have approximately 81% power to show a one-third (33%) relative reduction in median duration of symptoms from the start of investigational agent (e.g., 12 days to 8 days). This calculation is based on using a Wilcoxon rank sum test to compare groups using a two-sided significance level of 0.05.

© 2023 Bender Ignacio RA et al. *JAMA Network Open*.

eFigure. CONSORT Diagram

1A: CONSORT Diagram of Tixagevimab/Cilgavimab vs Placebo Intramuscular Administration

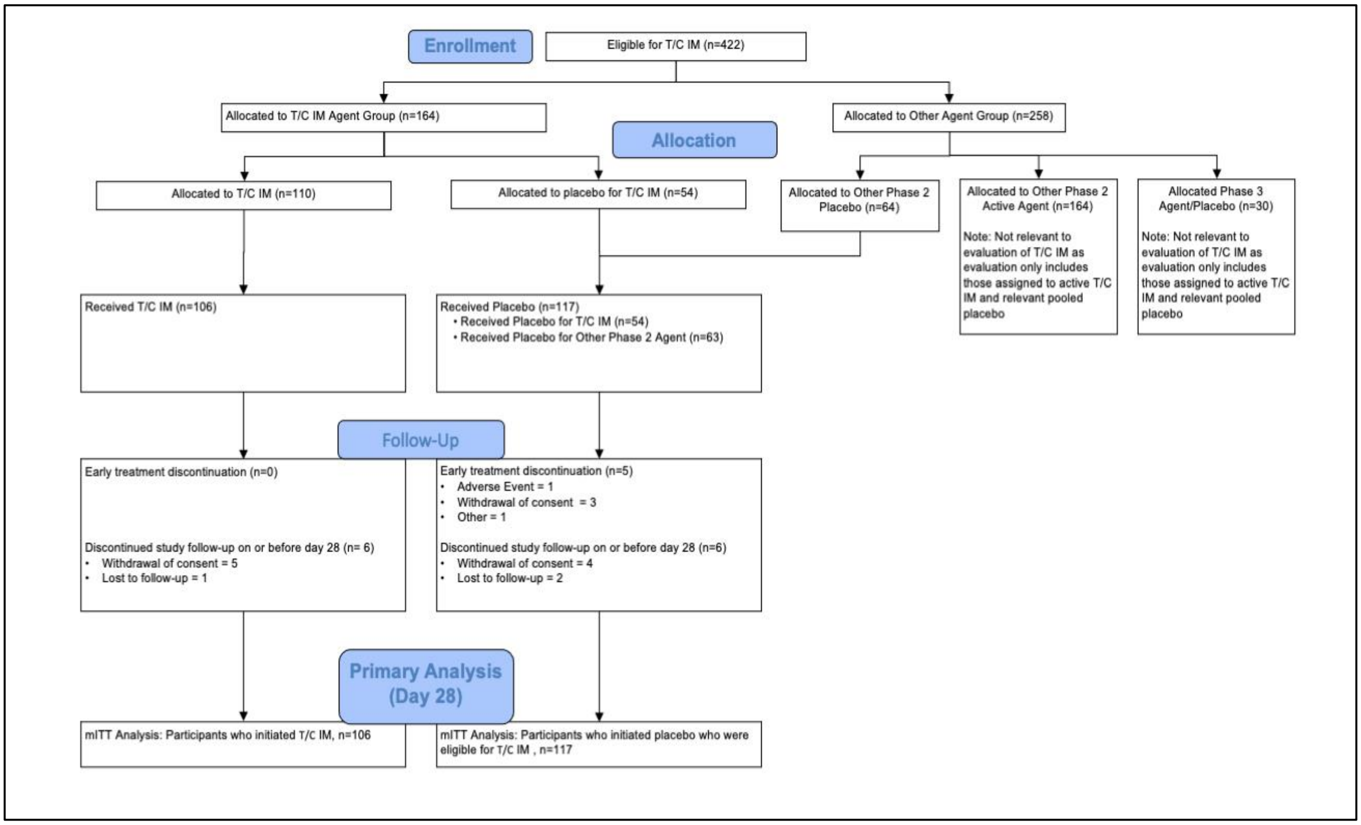

The primary modified intention-to-treat (mITT) population included all participants who began their assigned treatment. Four of 110 persons randomized to tixagevimab/cilgavimab IM never initiated treatment (n=106 included in mITT) and 1 of 118 persons allocated to pooled placebo did not initiate treatment (n=117 of these included in mITT).

Abbreviations: IM, intramuscular; T/C, tixagevimab/cilgavimab; mITT, modified intention-to-treat

© 2023 Bender Ignacio RA et al. *JAMA Network Open*.

1B: CONSORT Diagram of Tixagevimab/Cilgavimab vs Placebo Intravenous Administration

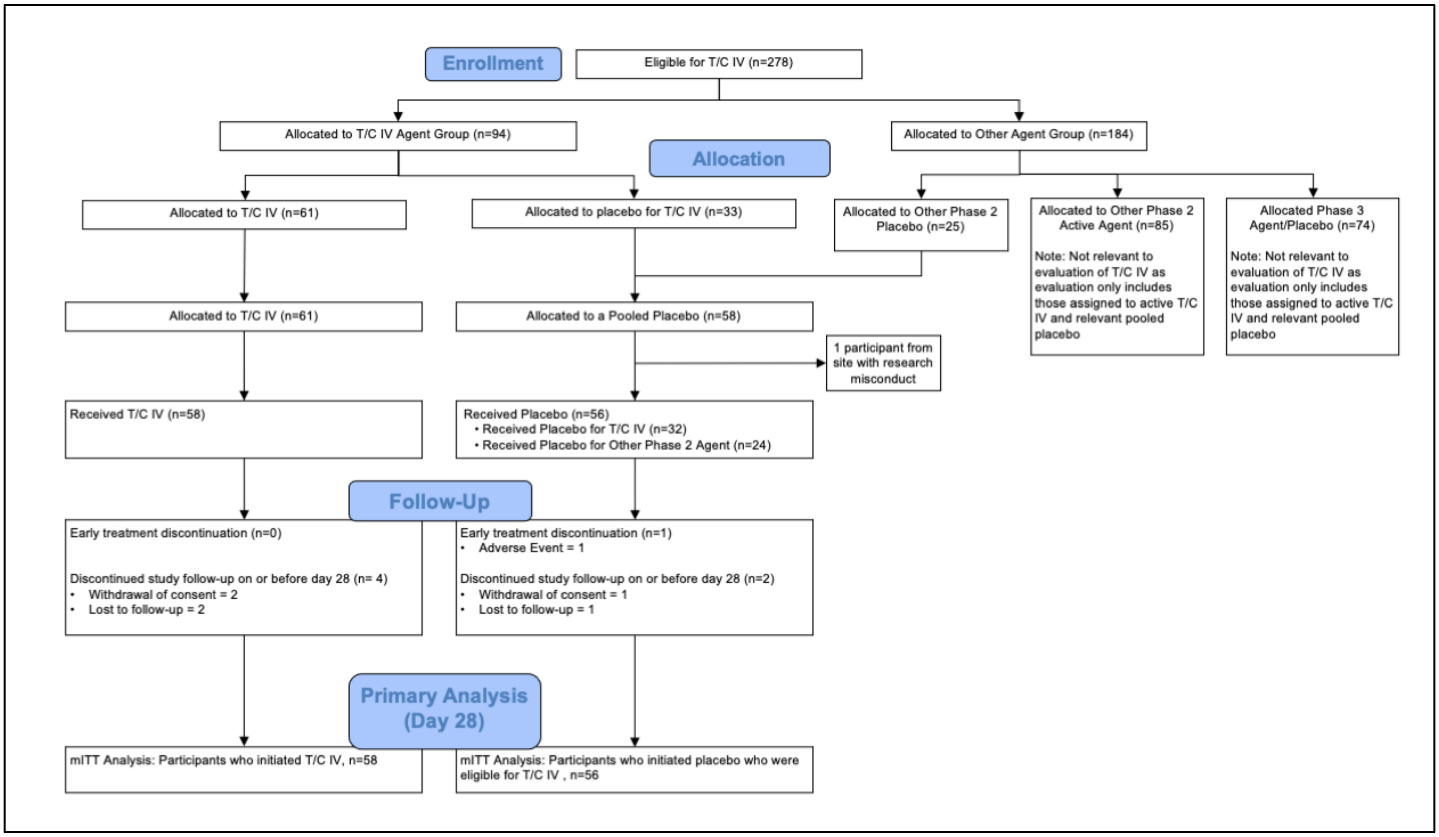

The primary modified intention-to-treat (mITT) population included all participants who began their assigned treatment. Three of 61 persons randomized to tixagevimab/cilgavimab IV never initiated treatment (n=58 included in mITT) and 1 of 58 persons allocated to pooled placebo did not initiate treatment. An additional pooled placebo participant was excluded from analysis due to research misconduct that precluded data reliability (n=56 of these included in mITT).

Abbreviations: IM, intramuscular; T/C, tixagevimab/cilgavimab; mITT, modified intention-to-treat

© 2023 Bender Ignacio RA et al. *JAMA Network Open*.

eTable 1. Clinical Outcomes Amongst Persons Randomized to Receive Tixagevimab-Cilgavimab, 600 mg IM or 300 mg IV, or Placebo

Outcome	T/C	Placebo	P-value<br>RR (95% CI)
**Tixagevimab/Cilgavimab 600mg IM (N=106) vs Placebo (N=117)**			
Symptom improvement for at least 2 days, median days to endpoint (95% CI) [PRIMARY]<br>n (%) meeting endpoint	8 (7, 12) days<br>81 (76%)	10 (8, 13) days<br>88 (75%)	0.35[A]
Symptom resolution lasting at least 2 days, median days to endpoint (95% CI)<br>n (%) meeting endpoint	14 (12, 16) days<br>72 (68%)	14 (10, 15) days<br>82 (70%)	0.62[A]
Return to usual pre-covid health lasting 2 days, median (95% CI)<br>n (%) meeting endpoint	14 (8, 16) days<br>67 (63%)	13 (11, 16) days<br>81 (69%)	0.79[A]
Time-averaged symptom scores through day 28 (AUC), Median (Q1, Q3)	2.2 (1.1, 4.3)	2.2 (1.1, 4.1)	0.87[B]
Progression of COVID-19-associated symptoms through day 28, n (%)	90 (85%)	100 (86%)	0.66[C]<br>0.98 (0.88, 1.08)[D]
Death or hospitalization through day 28[D], n (%)	4 (3.8%)	7 (6.0%)	0.54[E]
**Tixagevimab/Cilgavimab 300mg IV (N=58) vs Placebo (N=56)**			
Symptom improvement for at least 2 days, median days to endpoint (95% CI) [PRIMARY]<br>n (%) meeting endpoint	11 (9, 15) days<br>43 (74%)	10 (7, 15) days<br>44 (79%)	0.71[A]
Symptom resolution lasting at least 2 days, median days to endpoint (95% CI),)<br>n (%) meeting endpoint	15 (11, 23) days<br>36 (62%)	15 (8, 19) days<br>38 (68%)	0.59[A]
Return to usual pre-covid health lasting 2 days, median (95% CI)<br>n (%) meeting endpoint	12 (8, 16) days<br>39 (67%)	15 (9, 19) days<br>41 (73%)	0.51[A]
Time-averaged symptom scores through day 28 (AUC), Median (Q1, Q3)	2.6 (1.2, 4.2)	2.2 (1.3, 5.2)	1.00[B]
Progression of COVID-19-associated symptoms through day 28, n (%)	45 (78%)	47 (84%)	0.50[C]<br>0.94 (0.79, 1.12)[D]
Death or hospitalization through day 28[d], n (%)	0 (0%)	4 (7.1%)	0.06[E]

[A]Two-sided Gehan-Wilcoxon Test; [B]Two-sided Wilcoxon Rank-Sum test; [C]Wald test from Log-binomial regression; [D]No death events reported; [E]Fisher's Exact Test

Abbreviations: 95% CI= 95% Confidence Interval, Q1 = 1st Quartile; Q3 = 3rd Quartile; AUC = Area under the Curve

© 2023 Bender Ignacio RA et al. *JAMA Network Open*.

eTable 2. Clinical Outcomes Stratified by Symptom Duration Prior to Randomization

**Tixagevimab/Cilgavimab 600mg IM vs Placebo**				
**Outcome**	**≤ 5 Days**		**> 5 Days**	
	**T/C (n=48)**	**Placebo (n=53)**	**T/C (n=58)**	**Placebo (n=64)**
Symptom improvement for at least 2 days, median days to endpoint (95% CI)<br>n (%)	8 (6, 12) days<br>40 (83%)	10 (8, 14) days<br>41 (77%)	8 (7, 16) days<br>41 (71%)	10 (7, 13) days<br>47 (73%)
Death or hospitalization through day 28, n (%)	3 (6.3%)	2 (3.8%)	1 (1.7%)	5 (7.8%)
**Tixagevimab/Cilgavimab 300mg IV vs Placebo**				
**Outcome**	**≤ 5 Days**		**> 5 Days**	
	**T/C (n=24)**	**Placebo (n=23)**	**T/C (n=34)**	**Placebo (n=33)**
Symptom improvement for at least 2 days,<br>median days to endpoint (95% CI)<br>n (%)	10 (6, 16) days<br>18 (75%)	12 (6, 18) days<br>18 (78%)	13 (8, 16) days<br>25 (74%)	9 (6, 15) days<br>26 (79%)
Death or hospitalization through day 28, n (%)	0 (0%)	2 (8.7%)	0 (0%)	2 (6.1%)

© 2023 Bender Ignacio RA et al. *JAMA Network Open*.

eTable 3. Number and Proportion of Participants With SARS-CoV-2 RNA Levels Below and at or above the LLOQ From NP Swabs by Duration of Symptoms Prior to Enrollment (≤ 5 or >5 d), Amongst Those in the Tixagevimab-Cilgavimab, 600 mg IM and 300 mg IV, Groups

		**Tixagevimab/Cilgavimab IM**				**Tixagevimab/Cilgavimab IV**			
		**≤ 5 Days**		**> 5 Days**		**≤ 5 Days**		**> 5 Days**	
		**T/C IM (N=48)**	**Placebo (N=53)**	**T/C IM (N=58)**	**Placebo (N=64)**	**T/C IV (N=24)**	**Placebo (N=23)**	**T/C IV (N=34)**	**Placebo (N=33)**
Day 0	n	46	45	44	53	21	20	27	30
	< LLOQ, n (%)	6 (13)	9 (20)	9 (21)	13 (25)	2 (10)	6 (30)	12 (44)	13 (43)
	≥ LLOQ, n (%)	40 (87)	36 (80)	35 (80)	40 (76)	19 (90)	14 (70)	15 (56)	17 (57)
Day 3	n	39	42	46	50	19	18	31	28
	< LLOQ, n (%)	9 (23)	16 (38)	19 (41)	23 (46)	6 (32)	7 (39)	20 (65)	17 (61)
	≥ LLOQ, n (%)	30 (77)	26 (62)	27 (59)	27 (54)	13 (68)	11 (61)	11 (36)	11 (39)
Day 7	n	40	41	46	55	21	20	29	29
	< LLOQ, n (%)	30 (75)	23 (56)	39 (85)	39 (71)	13 (62)	9 (45)	24 (83)	26 (90)
	≥ LLOQ, n (%)	10 (25)	18 (44)	7 (15)	16 (29)	8 (38)	11 (55)	5 (17)	3 (10)
Day 14	n	38	44	45	51	20	21	27	28
	< LLOQ, n (%)	32 (84)	38 (86)	39 (87)	47 (92)	19 (95)	19 (91)	26 (96)	27 (96)
	≥ LLOQ, n (%)	6 (16)	6 (14)	6 (13)	4 (8)	1 (5)	2 (10)	1 (4)	1 (4)

Abbreviations: T/C, tixagevimab/cilgavimab; LLoQ = Lower Limit of Quantification (2.0 $\log_{10}$ copies/mL)

© 2023 Bender Ignacio RA et al. *JAMA Network Open*.

eTable 4. Change in NP RNA From Day 0 to Day 3 Among Those With NP RNA at Day 0 Greater Than LLOQ

	Total N (Total censored)	Mean difference* (log10 copies/mL)	95% CI	p-value
T/C IM vs Placebo	126 (32)	-0.147	[-0.718, 0.425]	0.61
T/C IV vs Placebo	55 (15)	-0.970	[-1.805, -0.134]	0.023

*Mean difference (T/C – Placebo) obtained from linear regression model for censored data adjusting for day 0 NP RNA.

Abbreviations: T/C, tixagevimab/cilgavimab; LLoQ = Lower Limit of Quantification (2.0 log_{10} copies/mL) ; LOD = Limit of Detection (1.4 log_{10} copies/mL)

© 2023 Bender Ignacio RA et al. *JAMA Network Open*.

eTable 5. Number and Proportion of Participants With Unquantifiable SARS-CoV-2 RNA Levels From Participant Self-collected Anterior Nasal (AN) Swabs Amongst Persons in the Tixagevimab-Cilgavimab, 600 mg IM and 300 mg IV, Groups

		**Tixagevimab/Cilgavimab IM**			**Tixagevimab/Cilgavimab IV**		
		**T/C IM (N=106)**	**Placebo (N=117)**	**aRR (95% CI)[A]**	**T/C IV (N=58)**	**Placebo (N=56)**	**aRR (95% CI)[A]**
Day 0	< LLOQ, n (%)	19 (21)	34 (33)	-	21 (41)	26 (50)	-
	≥ LLOQ, n (%)	73 (79)	69 (67)		30 (59)	26 (50)	
Day 1	< LLOQ, n (%)	25 (28)	39 (42)	0.73 (0.49, 1.10)	28 (54)	24 (50)	1.20 (0.86, 1.67)
	≥ LLOQ, n (%)	65 (72)	55 (59)		24 (46)	24 (50)	
Day 2	< LLOQ, n (%)	30 (33)	47 (50)	0.75 (0.53, 1.06)	27 (50)	26 (57)	0.96 (0.69, 1.32)
	≥ LLOQ, n (%)	60 (67)	48 (51)		27 (50)	20 (44)	
Day 3	< LLOQ, n (%)	43 (52)	48 (53)	1.12 (0.85, 1.47)	26 (55)	29 (62)	1.02 (0.76, 1.36)
	≥ LLOQ, n (%)	40 (48)	42 (47)		21 (45)	18 (38)	
Day 4	< LLOQ, n (%)	65 (74)	63 (66)	1.16 (0.97, 1.37)	35 (67)	30 (67)	1.10 (0.88, 1.38)
	≥ LLOQ, n (%)	23 (26)	33 (34)		17 (33)	15 (33)	
Day 5	< LLOQ, n (%)	**67 (82)**	**63 (68)**	**1.29 (1.10, 1.50)**	**42 (79)**	**33 (73)**	**1.21 (1.03, 1.42)**
	≥ LLOQ, n (%)	**15 (18)**	**30 (32)**		**11 (21)**	**12 (27)**	
Day 6	< LLOQ, n (%)	63 (77)	73 (79)	1.08 (0.93, 1.26)	**41 (82)**	**31 (72)**	**1.21 (1.01, 1.45)**
	≥ LLOQ, n (%)	19 (23)	19 (21)		**9 (18)**	**12 (28)**	
Day 7	< LLOQ, n (%)	**70 (89)**	**73 (78)**	**1.23 (1.08, 1.40)**	40 (91)	35 (80)	1.18 (1.00, 1.38)
	≥ LLOQ, n (%)	**9 (11)**	**21 (22)**		4 (9)	9 (21)	
Day 8	< LLOQ, n (%)	70 (89)	81 (88)	1.09 (0.97, 1.22)	43 (90)	38 (84)	1.10 (0.95, 1.27)
	≥ LLOQ, n (%)	9 (11)	11 (12)		5 (10)	7 (16)	
Day 9	< LLOQ, n (%)	72 (91)	83 (90)	1.09 (0.98, 1.21)	42 (89)	41 (89)	1.03 (0.90, 1.19)
	≥ LLOQ, n (%)	7 (9)	9 (10)		5 (11)	5 (11)	
Day 10	< LLOQ, n (%)	75 (93)	84 (91)	1.10 (1.00, 1.21)	46 (96)	40 (89)	1.13 (1.00, 1.26)
	≥ LLOQ, n (%)	6 (7)	8 (9)		2 (4)	5 (11)	
Day 11	< LLOQ, n (%)	74 (94)	85 (93)	1.11 (1.00, 1.22)	47 (96)	43 (96)	1.08 (0.96, 1.22)
	≥ LLOQ, n (%)	5 (6)	6 (7)		2 (4)	2 (4)	
Day 12	< LLOQ, n (%)	73 (92)	88 (96)	1.06 (0.96, 1.16)	46 (98)	44 (100)	1.06 (0.96, 1.18)
	≥ LLOQ, n (%)	6 (8)	4 (4)		1 (2)	0 (0)	
Day 13	< LLOQ, n (%)	76 (97)	85 (97)	1.09 (1.00, 1.18)	46 (96)	40 (95)	1.11 (0.98, 1.25)
	≥ LLOQ, n (%)	2 (3)	3 (3)		2 (4)	2 (5)	
Day 14	< LLOQ, n (%)	69 (97)	79 (98)	1.08 (1.00, 1.17)	43 (98)	35 (97)	1.07 (0.95, 1.20)
	≥ LLOQ, n (%)	2 (3)	2 (3)		1 (2)	1 (3)	
Overall P-value[B]		0.012			0.12		

[A]Adjusted Risk Ratio (aRR) comparing proportion of participants with SARS-CoV-2 RNA below LLoQ for Tixagevimab/Cilgavimab versus placebo, using a modified Poisson regression model adjusted for baseline log_{10}-SARS-CoV-2 RNA level, and an independent working correlation structure with robust standard errors for repeated measurements. Corresponding 95% CI for the risk ratio from the GEE fit.
[B]Two-sided Wald test

Abbreviations: T/C, tixagevimab/cilgavimab; LLoQ = Lower Limit of Quantification (2.0 log_{10} copies/mL) ; LOD = Limit of Detection (1.4 log_{10} copies/mL)

© 2023 Bender Ignacio RA et al. *JAMA Network Open*.

eTable 6. Number of Participants With Adverse Events Grade 2 or Higher Through Day 28

AE Category, n (%)	Tixagevimab/ Cilgavimab 600 mg IM		Tixagevimab/ Cilgavimab 300 mg IV	
	T/C IM (N = 106)	Placebo (N = 117)	T/C IV (N = 58)	Placebo (N = 56)
**Proportion with any AE of Grade ≥ 2**	**30 (28.3)**	**25 (21.4)**	**20 (34.5)**	**15 (26.8)**
**Investigations**	13 (12.3)	11 (9.4)	10 (17.2)	7 (12.5)
Alanine aminotransferase increased	3 (2.8)	2 (1.7)	2 (3.4)	1 (1.8)
Aspartate aminotransferase increased	0	1 (0.9)	0	0
Bilirubin conjugated increased	0	0	1 (1.7)	0
Blood bilirubin increased	1 (0.9)	0	1 (1.7)	0
Blood creatine phosphokinase increased	1 (0.9)	0	0	0
Blood creatinine increased	2 (1.9)	0	1 (1.7)	2 (3.6)
Blood glucose increased	6 (5.7)	5 (4.3)	4 (6.9)	1 (1.8)
Blood lactic acid increased	1 (0.9)	0	0	0
Blood potassium decreased	0	1 (0.9)	0	1 (1.8)
Blood potassium increased	1 (0.9)	0	0	0
Blood sodium decreased	0	0	1 (1.7)	1 (1.8)
Body temperature increased	0	1 (0.9)	0	0
Haemoglobin decreased	0	1 (0.9)	0	0
Hepatic enzyme increased	0	0	1 (1.7)	0
Lymphocyte count decreased	0	0	1 (1.7)	0
Neutrophil count decreased	0	1 (0.9)	0	1 (1.8)
Red blood cell sedimentation rate increased	1 (0.9)	0	0	0
Serum ferritin increased	1 (0.9)	0	0	0
Transferrin saturation decreased	1 (0.9)	0	0	0
**Infections and infestations**	8 (7.6)	10 (8.5)	1 (1.7)	6 (10.7)
Acute sinusitis	0	1 (0.9)	0	1 (1.8)
COVID-19 pneumonia	5 (4.7)	7 (6.0)	0	3 (5.4)
Diverticulitis	1 (0.9)	0	0	0
Gastroenteritis	0	1 (0.9)	0	0
Pneumonia	1 (0.9)	0	0	0
Pneumonia bacterial	0	0	0	1 (1.8)
Tooth infection	0	1 (0.9)	0	2 (3.6)
Upper respiratory tract infection	0	1 (0.9)	1 (1.7)	0
Vestibular neuronitis	1 (0.9)	0	0	0
**Respiratory, thoracic and mediastinal disorders**	12 (11.3)	4 (3.4)	3 (5.2)	2 (3.6)
Acute respiratory failure	1 (0.9)	2 (1.7)	0	1 (1.8)
Cough	4 (3.8)	0	1 (1.7)	0
Dyspnoea	4 (3.8)	2 (1.7)	1 (1.7)	0
Hypoxia	0	1 (0.9)	0	1 (1.8)
Nasal congestion	1 (0.9)	0	0	0
Nasal obstruction	2 (1.9)	1 (0.9)	0	0
Obstructive sleep apnoea syndrome	0	0	1 (1.7)	0
Oropharyngeal pain	2 (1.9)	0	0	0
Productive cough	1 (0.9)	0	0	0
Respiratory failure	1 (0.9)	0	0	0
Rhinorrhoea	2 (1.9)	1 (0.9)	1 (1.7)	0
Tachypnoea	0	1 (0.9)	0	0
**Metabolism and nutrition disorders**	3 (2.8)	3 (2.6)	1 (1.7)	3 (5.4)
Decreased appetite	0	1 (0.9)	0	0
Diabetes mellitus	1 (0.9)	0	0	0
Hyperglycaemia	2 (1.9)	2 (1.7)	0	2 (3.6)
Hyperkalaemia	0	0	0	2 (3.6)
Hypoglycaemia	0	0	0	1 (1.8)

© 2023 Bender Ignacio RA et al. *JAMA Network Open*.

AE Category, n (%)	Tixagevimab/ Cilgavimab 600 mg IM		Tixagevimab/ Cilgavimab 300 mg IV	
	T/C IM (N = 106)	Placebo (N = 117)	T/C IV (N = 58)	Placebo (N = 56)
Metabolic acidosis	0	0	0	1 (1.8)
Type 2 diabetes mellitus	0	0	1 (1.7)	0
**Gastrointestinal disorders**	3 (2.8)	3 (2.6)	1 (1.7)	2 (3.6)
Anal incontinence	1 (0.9)	0	0	0
Diarrhoea	0	1 (0.9)	0	0
Gastritis	1 (0.9)	0	0	0
Nausea	2 (1.9)	1 (0.9)	1 (1.7)	1 (1.8)
Toothache	0	1 (0.9)	0	1 (1.8)
Vomiting	0	0	0	1 (1.8)
**General disorders and administration site conditions**	1 (0.9)	3 (2.6)	1 (1.7)	3 (5.4)
Discomfort	1 (0.9)	0	0	0
Fatigue	0	1 (0.9)	1 (1.7)	2 (3.6)
Feeling abnormal	0	1 (0.9)	0	0
Pyrexia	0	1 (0.9)	0	1 (1.8)
**Nervous system disorders**	3 (2.8)	3 (2.6)	2 (3.4)	0
Dizziness	0	1 (0.9)	1 (1.7)	0
Dysaethesia	1 (0.9)	0	0	0
Headache	1 (0.9)	1 (0.9)	1 (1.7)	0
Paraesthesia	1 (0.9)	0	0	0
Syncope	0	1 (0.9)	0	0
**Vascular disorders**	4 (3.8)	1 (0.9)	1 (1.7)	1 (1.8)
Flushing	1 (0.9)	0	0	0
Hypertension	2 (1.9)	0	1 (1.7)	1 (1.8)
Hypotension	0	1 (0.9)	0	0
Thrombophlebitis	1 (0.9)	0	0	0
**Musculoskeletal and connective tissue disorders**	1 (0.9)	2 (1.7)	0	1 (1.8)
Arthralgia	0	2 (1.7)	0	1 (1.8)
Myalgia	1 (0.9)	1 (0.9)	0	0
**Psychiatric disorders**	0	3 (2.6)	0	1 (1.8)
Insomnia	0	3 (2.6)	0	1 (1.8)
**Blood and lymphatic system disorders**	2 (1.9)	0	1 (1.7)	0
Anaemia	1 (0.9)	0	1 (1.7)	0
Leukocytosis	1 (0.9)	0	0	0
**Injury, poisoning and procedural complications**	1 (0.9)	0	2 (3.4)	0
Infusion related reaction	0	0	2 (3.4)	0
Procedural vomiting	1 (0.9)	0	0	0
**Skin and subcutaneous tissue disorders**	1 (0.9)	1 (0.9)	1 (1.7)	0
Alopecia	0	0	1 (1.7)	0
Dermatitis contact	1 (0.9)	0	0	0
Dry skin	0	1 (0.9)	0	0
**Cardiac disorders**	0	0	0	1 (1.8)
Cardiac failure	0	0	0	1 (1.8)
**Immune system disorders**	1 (0.9)	0	0	0
Hypersensitivity	1 (0.9)	0	0	0

Preferred terms grouped by system organ class defined according to MedDRA coding (v24.0).

© 2023 Bender Ignacio RA et al. *JAMA Network Open*.

eTable 7. Variants Among Persons Randomized to Either IV or IM Tixagevimab-Cilgavimab or Placebo Groups

Of persons randomized to either IV or IM tixagevimab/cilgavimab or placebo groups, variant analysis of SARS-CoV-2 RNA from entry nasopharyngeal swab samples was available on 202 of 258 persons (78.3%).

Variant	Number	Percent of total analyzed
**Alpha**	115	56.9%
**Beta**	2	1.0%
**Gamma**	12	5.9%
**Delta**	1	0.5%
**Iota**	1	0.5%
**Epsilon**	23	11.4%
**Other (not variants of concern)**	35	17.3%
**Total**	202	

© 2023 Bender Ignacio RA et al. *JAMA Network Open*.

eMethods 2. Site Acknowledgments

Absolute Clinical Research, LLC, Phoenix, AZ, USA: James Beach (PI), Bianca Garcia, Priscilla Bustamante
Advance Medical Research Center, Miami, FL, USA: Ana Acosta (PI), Yenis Barbachan, Madelyn Ara
AIDS Research and Treatment Center of the Treasure Coast, Vero Beach, FL, USA: Gerald Pierone (PI), Juliana Elliott
Allegiance Research Specialists, Wauwatosa, WI, USA: Samuel Idarraga (PI), Andrew Kim
Allianz Research Institute Inc, Westminster, CA, USA: David N. Pham (PI), Wei-Hsin Kao
Allied Biomedical Research Institute, Miami, FL, USA: Michael M Pfeffer (PI), Miriam Batule Dominguez
Amber Clinical Research, LLC, Miami Shores, FL, USA: Francisco Patron (PI), Luz Guzman, Esnaldo Ferrer
Ascension St. John Clinical Research Institute, Tulsa, OK, USA: Anju Malik (PI), Anna Bryan, Melanie Arnold
Bozeman Health Deaconess Hospital, Bozeman, MT, USA: David Taylor (PI)
Bradenton Research Center Inc, Bradenton, FL, USA: Eric Folkens (PI)
Carolina Clinical Research, Charlotte, NC, USA: Emeka Eziri (PI)
Clinical Research Partners LLC, Richmond, VA, USA: Robert Call (PI), Leroy Vaughan
Clinical Trials Center of Middle Tennessee, Franklin, TN, USA: Aaron Milstone (PI), Jamie Alex Slandzicki, Jessica Wallan
Clinical Trials of America, LLC, Monroe, LA, USA: Clinton Guillory (PI), Nancy Andrews, Leslie Hughes
Clintheory, Orlando, FL, USA: Souheil Moussly (PI), LaShoun Sanders, Brittany Idlette
Community Research of South Florida, Hialeah, FL USA: Jose Carpio (PI), Nubia Concepcion
Cornell Clinical Trials Unit, New York Presbyterian Hospital-Weill Cornell Uptown, New York, NY, USA: Teresa Evering (PI), Jonathan Berardi, Celine Arar; additional grant support: UM1AI069419, UL1TR002384
Cullman Clinical Trials, Cullman, AL, USA: Randall Quinn (PI)
D&H National Research Centers, Miami, FL, USA: Jorge P. Amaya (PI)
Duke University Medical Center, Durham, NC, USA: Nwora Lance Okeke (PI), Folavan Morehead, Charles M. Burns
Fadi A. Haddad, MD, Inc, La Mesa, CA, USA: Fadi Haddad (PI), Victoria Haddad
Fairway Medical Clinic, Houston, TX, USA: Murtaza Mussaji (PI)
Family Clinical Trials, Pembroke Pines, FL, USA: Jeanne-Elyse Cedeno (PI), Lisbel Gonzalez, Lilliana Pestana
FOMAT Medical Research - FOMAT – HyperCore, Oxnard, CA, USA: Augusto Focil (PI), Griselda Rosas, Susana Moyano
Gonzalez MD & Aswad MD Health Services, Miami, FL, USA: Yaneicy Gonzalez Rojas (PI), Ahmad Aswad
IACT Health – Roswell – IACT – HyperCore, Columbus, GA, USA: Joseph Surber (PI), Jeffrey Kingsley, April Pixler
Infectious Disease Consultants of the Treasure Coast, Vero Beach, FL, USA: Laurie Welton (PI), Barbara Bromberg, Kelly Christina
Innovative Health Medical Center, Hollywood, FL, USA: Fernando Gonzalez Vergara (PI), Ana I. Gonzalez, Noemi Gonzalez
Inova Fairfax Medical Campus, Falls Church, VA, USA: Christopher deFilippi (PI), Allen Freiler, Brian Moore
James J Peters VA Medical Center - (CRS) – NAVREF, Bronx, NY, USA: Michael Gelman (PI), Olga Andriunas, Zarema Jagizarov; additional grant support: NIAID CRADA
Johns Hopkins University, Baltimore, MD, USA: Kelly Dooley (PI), Becky Becker, Adaliah Wilkins; additional grant support: additional grant support: UM1AI069465
Lakes Research, Miami Lakes, FL, USA: Jose Pérez (PI), Eloy Roman, Heriberto Fernández
Las Vegas Medical Research, Las Vegas, NV, USA: Bharat Mocherla (PI), Kelly Beck, Valarie Maldonado
Loma Linda University Health, Loma Linda, CA, USA: Jennifer Veltman (PI)
Massachusetts General Hospital, Boston, MA, USA: Rajesh Gandhi (PI), Katrina Shea, and Matthew Planchon; additional grant support: UM1AI069412
Miami Clinical Research, Miami, FL, USA: Keila Hoover (PI)
Moore Clinical Research, Inc. - Brandon – HyperCore, Brandon, FL, USA: George W. Monlux, Jr (PI), Elizabeth Juneja, Arthur Wernick
Northwestern University, Chicago, IL, USA: Babafemi O Taiwo (PI), Claudia Hawkins, Baiba Berzins; additional grant support: UM1AI069471 and UL1TR001422
Ohio State University Medical Center, Columbus, OH, USA: Carlos Malvestutto (PI), Heather Harber, Robyn Cicarella; additional grant support: UM1AI69494, UL1TR002733

© 2023 Bender Ignacio RA et al. *JAMA Network Open*.

Orlando Immunology Center Clinical Research Site, Orlando, FL, USA: Edwin DeJesus (PI), Charlotte-Paige Rolle
Pro Live Medical Research Corp, Miami, FL, USA: Rosa M. Suarez (PI), Ezequiel Socorro, Estefania Socorro
Puerto Rico AIDS Clinical Trials Unit, San Juan, Puerto Rico, USA: Jorge Santana-Bagur (PI), Sigrid Perez, Marielly Lopez; additional grant support: UM1AI69415, U54GM133807
Quantum Clinical Trials, Miami Beach, FL, USA: Gene Neytman (PI), Jack Herman, Craig Herman
Rush University Medical Center, Chicago, IL, USA: Mariam Aziz (PI), Joan Swiatek
Sanford USD Medical Center, Sioux Falls, SD, USA: Susan E. Hoover (PI), Allison Lutz, Jessica Just
Savin Medical Group LLC, Miami Lakes, FL, USA: Manuel Hernandez (PI), Yanly B Victoria, Gabriel Rodriguez
Snake River Research, PLLC, Idaho Falls, ID, USA: Martha Buitrago (PI)
Stanford University, Palo Alto, CA, USA: Upinder Singh (PI), Prasanna Jagannathan (PI), Divya Pathak
STAT Research, Springboro, OH, USA: Joshua J. Ordway (PI), Megan Heffner
The Miriam Hospital Clinical Research Site, Providence, RI: Karen Tashima (PI), Natasha Rybak, Helen Patterson; additional grant support: 3UM1AI068636-15S2
The University of Pittsburgh, Pittsburgh, PA, USA: Madhu Choudhary (PI), Jennifer Sullivano; additional grant support: UM1AI069494
Triple O Research Institute PA, West Palm Beach, FL, USA: Olayemi Osiyemi (PI), Myriam Izquierdo, Odelsey Torna
University at Buffalo, Emergency Medicine, Buffalo, NY, USA: Brian Clemency (PI), Renoj Varughese, Joshua Lynch; additional grant support: UL1TR001412
University of California Los Angeles, Los Angeles, CA, USA: Kara Chew (PI), Aleen Khodabakhshian, Samantha Fortier; additional grant support: UM1AI69424, UL1TR001881
University of California San Diego Antiviral Research Center, San Diego, CA, USA: Constance Benson (PI), Steven Hendrickx, Rosemarie Ramirez
University of Florida Jacksonville, Jacksonville, FL, USA: Mobeen Rathore (PI), Saniyyah Mahmoudi, Amna Riaz
University of Kansas Medical Center, Kansas City, KS, USA: Mario Castro (PI), Leslie Spikes, Chase Hall
University of North Carolina at Chapel Hill, Chapel Hill, NC, USA: David Wohl (PI), Jonathan Oakes, Amy James Loftis; additional grant support: UM1AI069423, UL1TR002489
University of Pennsylvania, Philadelphia, PA, USA: Pablo Tebas (PI), William Short; additional grant support: UM1AI069534
University of Washington AIDS Clinical Trials, Seattle, WA, USA: Rachel Bender Ignacio (PI), Sarah McGuffin, Chris Jonsson; additional grant support: UM1AI069841
UT Southwestern HIV/ID Clinical Trials Unit, Dallas, TX, USA: Mamta K. Jain (PI), Smruthi Senthil, Kimberly Turner-Gray
Vanderbilt Therapeutics Clinical Research Site, Vanderbilt Health-One Hundred Oaks, Nashville, TN, USA: David Haas (PI), Joan Gottesman, Beverly Woodward; additional grant support: UM1AI069439, UL1TR002243
Vida Clinical Studies, Dearborn, MI, USA: Derrick Williamson (PI), Hisham Atriss, Matthew Caloura
Zion Medical Center, San Diego, CA, USA: Adam Schwartz (PI), Donald P. Mebust, Clifford J. Swap

© 2023 Bender Ignacio RA et al. *JAMA Network Open*.
