# Supplementary material for: Safety and Efficacy of Combined Tixagevimab and Cilgavimab Administered Intramuscularly or Intravenously in Nonhospitalized Patients With COVID-19: 2 Randomized Clinical Trials
Source: JAMA Netw Open. 2023 Apr 26;6(4):e2310039. doi: 10.1001/jamanetworkopen.2023.10039 (PMC10134004; doi:10.1001/jamanetworkopen.2023.10039)
Supplement: Supplement 3. — Nonauthor Collaborators [file jamanetwopen-e2310039-s003.pdf]

Supplemental Online Content: Nonauthor Collaborators
*First name, last name, and suffix (if applicable) are required and will appear in PubMed.

*Group Name(s): The Accelerating COVID-19 Therapeutic Interventions and Vaccines (ACTIV)–2/A5401 Study Team							
***First Name and Middle Initial(s)**	***Last Name**	***Suffix (eg, Jr, III)**	Academic Degrees	Institution	Location (city, state/province, country)	Role or Contribution, eg, chair, principal investigator	Group (if more than 1 Group listed in the byline) and/or Subgroup (eg, Steering Committee)
Justin	Ritz		MS	Harvard T.H. Chan School of Public Health	Boston, MA, USA	statistician	
Lara	Hosey		MA	AIDS Clinical Trials Group Network Coordinating Cneter, Social and Scientific Systems, a DLH Company	Silver Spring, MD, USA	Clinical Trials Specialist	
Jhoanna	Roa		MD	AIDS Clinical Trials Group Network Coordinating Cneter, Social and Scientific Systems, a DLH Company	Silver Spring, MD, USA	Clinical Trials Specialist	
Nilam	Patel			AIDS Clinical Trials Group Network Coordinating Cneter, Social and Scientific Systems, a DLH Company	Silver Spring, MD, USA	Clinical Trials Specialist	
Kelly	Colsh		PharmD	NIH/DAIDS Pharmaceutical Affairs Branch	Rockville, MD, USA	DAIDS Pharmacist	
Irene	Rwakazina		PharmD	NIH/DAIDS Pharmaceutical Affairs Branch	Rockville, MD, USA	DAIDS Pharmacist	
Justine	Beck		PharmD	NIH/DAIDS Pharmaceutical Affairs Branch	Rockville, MD, USA	DAIDS Pharmacist	
Scott	Sieg		PhD	Case Western Reserve University	Cleveland, OH, USA	Protocol Immunologist	
Teresa	Evering		MD	Weill Cornell Medicine	New York, NY, USA	Protocol Investigator	
Sandra	Cardoso		MD, PhD	Fundação Oswaldo Cruz	Rio de Janeiro, Brazil	Protocol Investigator	
Katya	Corado		MD	Lundquist Institute at Harbor-UCLA Medical Center	Torrance, CA, USA	Protocol Investigator	

Supplemental Online Content: Nonauthor Collaborators
*First name, last name, and suffix (if applicable) are required and will appear in PubMed.

***First Name and Middle Initial(s)**	***Last Name**	***Suffix (eg, Jr, III)**	Academic Degrees	Institution	Location (city, state/province, country)	Role or Contribution, eg, chair, principal investigator	Group (if more than 1 Group listed in the byline) and/or Subgroup (eg, Steering Committee)
Prasanna	Jagannathan		MD	Stanford University School of Medicine	Palo Alto, CA, USA	Protocol Investigator	
Nikolaus	Jilg		MD, PhD	Massachusetts General Hospital, Harvard Medical School	Boston, MA, USA	Protocol Investigator	
Alan	Perelson		PhD	Los Alamos National Laboratory	Los Alamos, NM, USA	Protocol Investigator	
Sandy	Pillay		Mb, CHB	Enhancing Care Foundation	Durban, KwaZulu-Natal, South Africa	Protocol Investigator	
Cynthia	Riviere		MD	GHESKIO Center	Port-au-Prince, Haiti	Protocol Investigator	
Upinder	Singh		MD	Stanford University School of Medicine	Palo Alto, CA, USA	Protocol Investigator	
Babafenu	Taiwo		MBBS, MD	Northwestern University Feinberg School of Medicine	Chicago, IL, USA	Protocol Investigator	
Joan	Gottesman		BSN, RN, CCRP	Vanderbilt University Medical Center	Nashville, TN, USA	Field Representative	
Susan	Pedersen		BSN, RN	University of North Carolina at Chapel Hill School of Medicine	Chapel Hill, NC, USA	Field Representative	
Cheryl	Jennings		BS	Northwestern University	Chicago, IL, USA	Laboratory Technologist	
Brian	Greenfelder		BA	Ohio State University	Columbus, OH, USA	Laboratory Technologist	
William	Murtaugh		MPH	ACTG Laboratory Center, University of California Los Angeles	Los Angeles,, CA, USA	Laboratory Specialist	
Jan	Kosmyna		MIS, RN, CCRP	Case Western Reserve University	Cleveland, OH, USA	ACTG Community Scientific Subcommittee Representative	

Supplemental Online Content: Nonauthor Collaborators
*First name, last name, and suffix (if applicable) are required and will appear in PubMed.

***First Name and Middle Initial(s)**	***Last Name**	***Suffix (eg, Jr, III)**	Academic Degrees	Institution	Location (city, state/province, country)	Role or Contribution, eg, chair, principal investigator	Group (if more than 1 Group listed in the byline) and/or Subgroup (eg, Steering Committee)
Morgan	Gapara		MPH	AIDS Clinical Trials Group Network Coordinating Cneter, Social and Scientific Systems, a DLH Company	Durham, NC, USA	International Site Specialist	
Akbar	Shahkolahi		PhD	AIDS Clinical Trials Group Network Coordinating Cneter, Social and Scientific Systems, a DLH Company	Silver Spring, MD, USA	International Site Specialist	
Robert	Gasser	Jr	MD	Kelly Services, a Service Provider to AstraZeneca	Gaithersburg, MD, USA	Industry Representative	
