# Supplementary material for: Safety and Efficacy of Combined Tixagevimab and Cilgavimab Administered Intramuscularly or Intravenously in Nonhospitalized Patients With COVID-19: 2 Randomized Clinical Trials
Source: JAMA Netw Open. 2023 Apr 26;6(4):e2310039. doi: 10.1001/jamanetworkopen.2023.10039 (PMC10134004; doi:10.1001/jamanetworkopen.2023.10039)
Supplement: Supplement 4. — Data Sharing Statement [file jamanetwopen-e2310039-s004.pdf]

Data Sharing Statement

Bender Ignacio. Safety and Efficacy of Combined Tixagevimab and Cilgavimab Administered Intramuscularly or Intravenously in Nonhospitalized Patients With COVID-19. *JAMA Netw Open*. Published April 26, 2023. doi:10.1001/jamanetworkopen.2023.10039

Data
Data available: Yes
Data types: Deidentified participant data, Data (not involving human participants), Data dictionary
How to access data: The next-generation sequencing data generated in this study have been deposited on the NCBI Short Read Archive (SRA) under accession number PRJNA935778 and PRJNA935802. All other are available under restricted access due to ethical restrictions, with trial conduct ongoing. Access can be requested by submitting a data request at https://submit.mis.s-3.net/ and will require the written agreement of the AIDS Clinical Trials Group (ACTG) and the manufacturer of the investigational product. Requests will be addressed as per ACTG standard operating procedures. Completion of an ACTG Data Use Agreement may be required.
When available: With publication

Supporting Documents
Document types: Informed consent form, Other (please specify)
Additional Information: SAP, full protocol, and protocol eligibility revisions
How to access documents: Provided in submitted supplement
When available: With publication

Additional Information
Who can access the data: Data are available under restricted access due to ethical restrictions, with trial conduct ongoing. Access can be requested by submitting a data request at https://submit.mis.s-3.net/ and will require the written agreement of the AIDS Clinical Trials Group (ACTG) and the manufacturer of the investigational product. Requests will be addressed as per ACTG standard operating procedures. Completion of an ACTG Data Use Agreement may be required.
Types of analyses: Can be obtained for any purpose, but the concept requires ACTG approval for scientific merit and appropriate data usage.
Mechanisms of data availability: Data generally available without investigator support, but some mechanisms exist for funding new concepts with existing data within the ACTG. ACTG Data Use Agreements are generally required
